# Burden and Inattentive Responding in a 12-Month Intensive Longitudinal Study: Interview Study Among Young Adults

**DOI:** 10.2196/52165

**Published:** 2024-08-02

**Authors:** Shirlene D Wang, Lori Hatzinger, Jeremy Morales, Micaela Hewus, Stephen Intille, Genevieve F Dunton

**Affiliations:** 1 Department of Population and Public Health Sciences Keck School of Medicine University of Southern California Los Angeles, CA United States; 2 Bouvé College of Health Sciences Northeastern University Boston, MA United States; 3 Khoury College of Computer Sciences Northeastern University Boston, MA United States

**Keywords:** data quality, burden, exit interview, careless responding, ecological momentary assessment, intensive longitudinal data collection, mobile phone

## Abstract

**Background:**

Intensive longitudinal data (ILD) collection methods have gained popularity in social and behavioral research as a tool to better understand behavior and experiences over time with reduced recall bias. Engaging participants in these studies over multiple months and ensuring high data quality are crucial but challenging due to the potential burden of repeated measurements. It is suspected that participants may engage in inattentive responding (IR) behavior to combat burden, but the processes underlying this behavior are unclear as previous studies have focused on the barriers to compliance rather than the barriers to providing high-quality data.

**Objective:**

This study aims to broaden researchers’ knowledge about IR during ILD studies using qualitative analysis and uncover the underlying IR processes to aid future hypothesis generation.

**Methods:**

We explored the process of IR by conducting semistructured qualitative exit interviews with 31 young adult participants (aged 18-29 years) who completed a 12-month ILD health behavior study with daily evening smartphone-based ecological momentary assessment (EMA) surveys and 4-day waves of hourly EMA surveys. The interviews assessed participants’ motivations, the impact of time-varying contexts, changes in motivation and response patterns over time, and perceptions of attention check questions (ACQs) to understand participants’ response patterns and potential factors leading to IR.

**Results:**

Thematic analysis revealed 5 overarching themes on factors that influence participant engagement: (1) friends and family also had to tolerate the frequent surveys, (2) participants tried to respond to surveys quickly, (3) the repetitive nature of surveys led to neutral responses, (4) ACQs within the surveys helped to combat overly consistent response patterns, and (5) different motivations for answering the surveys may have led to different levels of data quality.

**Conclusions:**

This study aimed to examine participants’ perceptions of the quality of data provided in an ILD study to contribute to the field’s understanding of engagement. These findings provide insights into the complex process of IR and participant engagement in ILD studies with EMA. The study identified 5 factors influencing IR that could guide future research to improve EMA survey design. The identified themes offer practical implications for researchers and study designers, including the importance of considering social context, the consideration of dynamic motivations, and the potential benefit of including ACQs as a technique to reduce IR and leveraging the intrinsic motivators of participants. By incorporating these insights, researchers might maximize the scientific value of their multimonth ILD studies through better data collection protocols.

**International Registered Report Identifier (IRRID):**

RR2-10.2196/36666

## Introduction

### Background

Intensive longitudinal data (ILD) collection methods are becoming more popular in social and behavioral research due to the development of methodology, data analysis, and technology, which extends the feasibility of this approach to accurately understanding behaviors and experiences over time. A common ILD study design uses ecological momentary assessment (EMA), which involves collecting repeated self-report data in real time [1]. Unlike most other survey sampling methods, EMA reduces recall biases associated with cross-sectional surveys and has the benefit of examining time-varying (within-person or within-survey) factors [2]. However, despite these strengths, sustaining participant engagement, defined as motivation to complete study procedures, can be challenging, given the potential burden of completing repeated surveys. Contemporary EMA studies typically send participants multiple survey prompts per day on a personal mobile device that occur randomly throughout the day or on fixed schedules (ie, every 2 hours). The design of EMA studies depends on the data required to pursue the research question of interest, so studies can range from prompting once a day (daily diary) to several times a day, with some prompting schedules that are as dense as every 15 minutes. The length of studies also ranges from periods as short as a few hours to >1 year in length. Increased participant burden to complete study procedures may occur due to EMA study design or specific time-varying factors influencing the participant, such as the time of day or physical and social contexts. Beyond compliance and nonresponse, lack of engagement may also consist of careless or inattentive responding (IR), in which participants provide lower-quality data due to having low motivation to comply with survey instructions, correctly interpret item content, or provide accurate responses [3].

The accuracy of self-report data has been a long-standing concern and has been identified as a source of error [4,5]. While there are methods to ensure that response inaccuracy is not due to misunderstanding (eg, screening out participants who do not have strong language comprehension or cognitive difficulties that would influence participation), addressing inaccuracy due to cognitive nonengagement and low motivation has been more challenging for researchers. The prevalence of IR in EMA studies should be concerning because it leads to inaccurate data, threatens the reliability and validity of self-report data, and can lead to measurement errors. Previous estimates in emerging adult samples estimate that the median rate of IR in cross-sectional self-report methods is 10% to 12%, with some estimates of 50% to 72% on single items [6-8]. While statistical analysis methods such as multilevel modeling may be robust to missing data, they are not robust to false or deceitful data. Simulation studies have suggested that a small proportion of inattentive responders (5%-10%) is enough to alter study results and lead to different conclusions regarding hypotheses [9]. While random responding adds noise to the data set, inattentive responses introduce systematic bias [10]. These error variances reduce reliability estimates, measurement precision, and attenuate or inflate correlations [11-13]. Published studies based on the analysis of these “uncleaned” data sets may present misleading and unrepresentative conclusions. In addition, because EMA data are often used to interpret within-person effects, inattentive responses misattributed as individual differences could introduce error variances to results [14].

IR has rarely been studied in EMA studies. Solutions to support participants contributing meaningful and high-quality data have been evaluated [15,16]. Researchers commonly evaluate engagement with the EMA protocol through retrospective assessments of participants’ experiences in the study. Behavioral and affective aspects of participant engagement in EMA have been assessed through compliance (eg, performing the task of answering surveys) and acceptability (eg, assessing if participants enjoy the tasks), but there is limited research on cognitive engagement with EMA (eg, do participants spend time thinking about the tasks? Do participants engage in cognitive processing of the questions?) [17]. It has been proposed that participants may have less cognitive engagement in observational EMA data collection compared to intervention-related EMA data collection due to a lack of direct benefit from the data monitoring [18]. As a relatively novel approach, the cognitive challenges associated with EMA as a methodological technique must be further addressed. Currently, there is a lack of knowledge of the behavioral or psychological processes underlying why and in what situations individuals respond inattentively. Following Ajzen’s theory of planned behavior by Madden et al [19], the intention to engage in a behavior (eg, respond to an EMA survey attentively) could be thought of as a function of social norms (ie, acceptability of answering surveys in their physical or social context) and intrapersonal attitudes (ie, participant’s desire or motivation to satisfy researchers or earn compensation). To confirm the associations proposed in this theory, qualitative research methods could be used to directly ask participants to describe their process of responding to EMA surveys. Accordingly, this study was designed to broaden researchers’ knowledge about IR in studies that use EMA and begin to uncover the underlying processes of IR to aid with future hypothesis generation for further quantitative investigation of this phenomenon.

Qualitative interviews have previously been conducted with emerging adult participants in EMA studies to assess the acceptability of various EMA protocols and to understand better the experience behind completing surveys and the barriers to data collection for various health behaviors [20-25]. However, these studies have mainly focused on the barriers to compliance rather than the barriers to providing high-quality data. There have been 2 previous mixed methods studies assessing the accuracy of EMA data using brief qualitative interviews with a subset of participants that highlighted study design factors such as the timing of prompts that led to fatigue, which hindered engagement [26,27]. However, the EMA protocols in these studies were short (14 days), and results may highlight changes in the data quality that appear relatively early and miss slower or more long-term changes that could occur during longer EMA protocols.

### Objectives

To advance the field, we collected qualitative data from 31 young adult (aged 18-29 years) participants who completed a 12-month intensive longitudinal EMA study with daily evening EMA surveys and 4-day waves of hourly EMA surveys. Semistructured interviews and thematic analysis were conducted to understand participants’ response patterns and potential factors leading to IR. The goals were to understand (1) why participants joined the study and their motivation to continue, (2) the effects of time-varying contexts on IR, (3) changes to motivation or response patterns over time, and (4) perceptions of the attention check questions (ACQs). Through the interviews, rich descriptive data were captured about the potential burden of an intensive EMA study lasting 12 months, and through the analysis, we can begin to explain “why” participants may provide inattentive responses.

## Methods

### Design

This study was conducted using a subsample of participants enrolled in the larger Temporal Influences on Movement and Exercise (TIME) study [28]. The overall TIME study sample consisted of emerging adults aged between 18 and 29 years living in the United States who were recruited on the web. To be eligible for the study, the participant had to use a compatible Android-based smartphone and intend to engage in recommended levels of moderate to vigorous physical activity within the next 12 months. Participants were excluded for physical or cognitive disabilities that prevented participation and inability to wear the smartwatch or answer EMA surveys at locations where the participant spent a substantial amount of time (eg, >20% of the time). The TIME study is exceptional due to the long-term and demanding nature of data collection. The overarching objective of the 12-month study was to use real-time mobile technologies to collect ILD examining differences in the microtemporal processes underlying the adoption and maintenance of physical activity, low sedentary time, and sufficient sleep duration. Participants completed a daily diary smartphone EMA survey at the end of each day as well as hourly EMA surveys across 4-day measurement bursts every 2 weeks. Each hourly EMA survey started with an audio chime, a vibration lasting 11 seconds, and a persistent notification. EMA surveys included up to 27 back-to-back multiple choice questions (requiring 1-2 minutes to complete). If the participant did not complete the survey after the initial notification, the app repromoted it after 5 minutes; if 10 minutes elapsed and the study was still not completed, it disappeared and became inaccessible to the participant. Participants received US $10 for each EMA burst if they completed at least 8 surveys per day. In addition, if the participant answered >11 EMA burst surveys on a given day, they received a US $5 bonus for that day. The TIME app showed a persistent notification on the smartphone that displayed the number of answered versus surveyed EMA surveys on a given burst day to help participants monitor their daily compliance. To objectively measure data quality, 20% of the hourly EMA surveys included an ACQ that asked about obvious facts with clear, unambiguous answers (eg, what color is the sky? blue, green, red, sixteen, and pliers and which of these is an animal? polar bear, iPhone 7, chai tea latte, snowball, and microwave). Individuals who successfully completed the full year of data collection after April 22, 2022, were asked to participate in an end-of-study session with the study staff on Zoom (Zoom Video Communications) that included a 30-minute semistructured interview designed to elicit feedback on their experiences in the study and perceptions about their EMA survey responses. The data analyzed in this substudy were collected between April 22 and August 29, 2022.

This study used a qualitative research design to explore participants’ lived experiences, behaviors, feelings, perceptions, and interactions with the EMA study protocol. The semistructured interview style balanced the need for structure to elicit data for the study with a conversational approach to build rapport with the participants and allow for flexibility in responses. The guiding research question was, “How can we discover the process and sequence of decisions that individuals make that may contribute to low-quality data?”

### Ethical Considerations

The study was approved by the institutional review board at the University of Southern California (HS-18-00605). All data collected were deidentified. Participants provided informed consent to have their deidentified research data included in secondary analyses and published in journals. Participants were compensated for their participation in the main study [28], but there was no additional compensation provided for the interview session in this substudy.

### Interview Procedure

Participants were asked a series of predetermined questions developed by author SDW (Textbox 1). Additional probing questions were asked depending on their initial responses to gain further clarity about participants’ decision-making process. The interviews were guided by the primary research question but also allowed for new ideas and themes to be discussed. All interviews were recorded through Zoom, which generated an audio file and a video file. However, for the purposes of this study, only the audio file was kept and stored on a secure, shared drive for transcription, and the video was deleted.

**Interview guide used for semistructured interviews with participants to explore potential factors that may lead to inattentive responding.**

**Theme and questions**

- Motivation
  How did you learn about this study? (probe: what features of the study interested you to want to participate?)
  Can you describe what motivated you to continue to answer surveys in this study? (probe: if not discussed ask about the motivating level of money; motivation to help science; reflection; ease of answering; How important was the compensation from the study to you?)
  Can you describe the process of answering phone surveys on a typical burst day? (probe: how many phone surveys do you think you answered on a typical day? Did you have a goal for the number of surveys you were trying to reach? Did you track completion?)
  What would have made participation in the study more fun or rewarding? (probe: try to identify nonmonetary factors)
- Situations of increased burden
  How did you handle distractions when taking the survey?
  Were there situations in which your responses to the surveys may have been less accurate (that you answered without thinking through your responses)? (probe: how did your responses change if someone else was around? Depending on your location? Different times of the day?)
- Response accuracy
  Were there situations in which your responses to the surveys may have been less accurate (that you answered without thinking through your responses)? (probe: how did your responses change if someone else was around? Depending on your location? Different times of the day?)
  How do you think your motivation or accuracy changed as you were in the study longer? (probe: what made the study easier or harder over time?)
- Perceptions of attention check questions
  What did you think about the questions and messages that were not related to measuring health behaviors, routines, and mood on the phone? (probe: which ones were the most memorable? Any suggestions on how we can make them better?)

### Data Analysis

The collected qualitative data were systematically analyzed using an iterative process following recommendations by Charmaz [29]. The audio files were deidentified using the participants’ study ID and transcribed verbatim by an external transcription service provider (GoTranscript). Transcripts were revised to correct errors manually. Multimedia Appendix 1 contains the transcripts. First, author SDW independently reviewed the documents and summarized ideas through memos. An initial set of gerunds was created by extracting significant language and patterns and focused primarily on the research question and was centered around the habit of responding to surveys and reactions to the ACQs. From these, codes began to form after looking at the broader testimonies that participants provided. All codes were revised and condensed to create a total set of 13 thematic codes. Definitions and an example from the transcripts for each thematic code were provided in the codebook. Transcripts were then uploaded onto the qualitative data analysis software program ATLAS.ti (ATLAS.ti Scientific Software Development GmbH). Using the program, the thematic codes were applied to the transcripts to conduct a qualitative assessment of the factors that influenced the data quality of participants. Each transcript was independently coded by 3 different authors (SDW, LH, and JM) to ensure consistent application of codes. The team discussed and resolved disagreements using the ATLAS.ti software Intercoder Agreement Mode. After a high level of agreement was reached between coders (Krippendorff α-binary [global]>0.8), 5 overarching themes that aggregated the 13 codes emerged. These themes highlight a range of concepts that participants associated with their study participation and data quality. The distribution of themes and codes was checked across transcripts to ensure that they adequately covered the overall discussions.

## Results

### Participant Characteristics

A total of 31 participants were interviewed, 24 (77%) of whom were female participants. The largest portion of participants (14/31, 45%) self-identified as Asian, had graduated from college, and were employed. Demographics for these participants collected at baseline and 12-month web-based surveys are presented in Table 1. Our qualitative study subsample (n=31) differed demographically compared to the full sample of participants who completed the 12-month intensive longitudinal study (N=136) in 1-tailed *t* tests. Our subsample was predominantly female (24/31, 77% vs 57.4%, *t*_165_=–2.06, *P*=.02); included fewer Hispanic (7/31, 23% vs 32.4%, *t*_165_=1.06, *P*=.14), fewer White (9/31, 29% vs 48.1%, *t*_165_=1.93, *P*=.03), more Asian (14/31, 45% vs 37%, *t*_165_=–0.85, *P*=.20) individuals; had a higher proportion of college graduates (22/31, 71% vs 49.3%, *t*_165_=–2.19, *P*=.02); and had a high proportion of employed or self-employed individuals (20/31, 65% vs 56.3%, *t*_165_=–0.83, *P*=.20) compared to students (12/31, 39% vs 48.4%, *t*_165_=.97, *P*=.16). Multimedia Appendix 2 contains demographic information of the participants.

Interviews ranged from 12 to 37 (mean 21.30, SD 7.15) minutes in length, and the number of quotes coded per participant ranged from 6 to 63 (mean 25.87, SD 12.98). The 13 thematic codes that were constructed after analyzing all 31 transcripts are presented with the number of participants (N=31) who discussed each code (Table 2).

The 13 abovementioned codes were distilled into 5 overarching themes. The 5 themes presented do not encompass all participant experiences but aim to provide a general overview of them.

**Table 1:** Participant demographics for the study subsample that completed exit interviews (N=31).

Characteristics		Values
Age (y), mean (SD)		24.4 (3.1)
**Sex, n (%)**		
	Male	7 (23)
	Female	24 (77)
**Ethnicity, n (%)**		
	Non-Hispanic	24 (77)
	Hispanic	7 (23)
**Race, n (%)^a^**		
	Asian	14 (45)
	Black	2 (7)
	White	9 (29)
	Multiple races	5 (16)
	None indicated	1 (3)
**Education, n (%)**		
	High school	4 (13)
	Some college	5 (16)
	College graduate	22 (71)
**Work status, n (%)^a,b^**		
	Employed	20 (65)
	Self-employed	4 (13)
	Out of work	3 (10)
	Student	12 (39)
	Homemaker	2 (7)
**Marital status^a^, n (%)**		
	Never married	20 (65)
	Unmarried couple	7 (23)
	Married	4 (13)
**Financial status, n (%)**		
	Live comfortably	10 (32)
	Meet needs with a little left	15 (48)
	Just meet basic expenses	6 (19)

^a^Assessed at the end of the study (12-month survey).

^b^Participants could select more than one option.

**Table 2:** Thematic codes and description (n=31).

Thematic codes	Description	Frequency, n (%)
Interrupting social situations	Surveys interfering with social situations	29 (94)
Earning compensation	Earning payment for the study and the minimum threshold of surveys for payment	28 (90)
Habit of responding to surveys	Familiar routine around the surveys	24 (77)
Identifying the purpose of ACQ^a^	Recognizing researchers are testing their attention with the ACQs	23 (74)
Feeling internal motivation	Internal factors driving survey completion rather than money	22 (71)
Contemplating response	Thoughtfulness about responses (opposite of IR^b^) and discussing reactivity	22 (71)
Response speed	Responding to surveys quickly	19 (61)
Question difficulty	Cognitive difficulty of questions; not sure what question was asking	19 (61)
Reactivity to attention check question	Describing a change in response patterns due to ACQs	16 (52)
Contributing to science	External motivation outside of compensation (eg, want to help researchers)	15 (48)
Feeling judged by others	Others’ negative perceptions of the participant answering surveys	11 (35)
Disrupting screen time	Survey causing interference by displaying over other phone tasks	8 (26)
Receiving assistance answering surveys	Participant had others help answer the survey (eg, if the participant was busy driving)	6 (19)

^a^ACQ: attention check question.

^b^IR: inattentive responding.

### Theme 1: My Friends and Family Also Had to Tolerate the Frequent Surveys

All participants described how they fit the hourly EMA surveys (lasting 4 days every 2 weeks) into their routines and lifestyles, which was required to tolerate the disruptions, with many describing the process of responding as a habit. Given the young adult population, for many, their usual routines involved spending time with others, such as friends and family, who had to tolerate these surveys, as well. They commonly described the reaction of others and how answering the burst surveys became routine during their social interactions. Some participants’ social companions could tolerate the surveys better than others. For example:

But also, it really did become part of my routine. I remember, at the beginning, someone saying it kind of becomes part of your routine, and it really did. It was kind of just funny, like sometimes I’ve been playing games with friends, and I’ll be like, “Oh, we have to pause it’s survey time,” and they got it. They just understood, and it became part of our thing.

9252, female; aged 25 years

I think that piece of habit definitely played a role. I didn’t necessarily feel as motivated towards the end, but it was just a part of my daily routine. I guess for me, it was just easy because it was second nature, I guess, by that point. Maybe for friends and family, it was a little bit more frustrating just because they’re like, “He’s going to go do a survey.”

9310, male; aged 25 years

I think there are a couple or a few times when a buzz day would overlap with some social thing, and so I would tell whoever I was with, “Oh, I have this survey thing that comes every hour or so. Every hour, I need to quickly answer some questions on my phone.” I did have a friend who was like, “Oh, you’re being rude,” and blah, blah, blah. That was not pleasant, but I feel like that was more of my friend’s problem.

9314, female; aged 27 years

Because on occasion, it would be a little bit annoying because it interferes with social life a little bit, and when you’re talking with somebody it can be rude to just look at your watch or look at your phone or something. There’s a little bit of that, but that’s what you knew going in, so I knew this was going to happen. It’s just every once in a while, you’re just like, “This is annoying and inconvenient,” but other than that, it felt fine.

9284, male; aged 28 years

When hourly EMA surveys came in during social gatherings, the 2 main strategies described by participants involved either physically stepping away and leaving conversations to complete the survey with more focus or trying to multitask and take the survey in front of others. When completing an EMA survey in front of others, participants also encountered situations where they had to decide whether to provide a rationale for their phone use and how much of the study to explain:

[I]t did get complicated because having to stop, especially if you’re socializing with people and having to stop then, okay, do I either explain to them what I’m doing and then try to explain to them the whole study or just, “Give me a minute, let me do this real quick.”

9248, female; 29 years

It’s just some of those exposed scenarios when you’re out. For me personally, at least, you’re out at a dinner or there’s people around you or something, you cannot go by yourself or take your phone and focus on the survey. You’re just passively filling the survey out while you’re doing other things.

9282, male; aged 24 years

I would just say like, oh, can you gimme like two minutes and usually I try to multitask by explaining to them telling a story about it. Makes it more engaging with other people.

9271, female; aged 23 years

I think it was doing the actual survey on the phone just because in those scenarios where I am with friends, but there would be times where I’d be like, okay, well, we’re all hanging out, but it’s no big deal. Then every hour, I would just be bringing out my phone, like, “Sorry guys. I just have some stuff to do real quick.” Again, it only took a minute so no big deal, but I guess it’s disruptive.

9276, male; aged 28 years

Thus, for many participants, the friends and family they saw regularly began to understand the frequency of the surveys. However, these interruptions were typically recounted in interviews humorously:

Other times I would—I think my family also got used to it after one point that they knew, almost everyone knew that I’m doing this, so they would repeat it for me. [laughs]

9287, female; aged 29 years

Yes, especially the friends that I saw all the time, they all knew. They just like, “Okay, hold on survey time”...Sometimes they would hear the buzzing and be like, “Oh, it’s time for you to do a survey.” I’d be like, “Oh, thanks for telling me.”

9296, female; aged 25 years

The notification is disruptive, but it has to be in order to get my attention, I suppose. My brother and I were joking that we have PTSD from [mimics notification sound] I would be like, “Oh, gosh, it’s coming now.”

9245, female; aged 28 years

However, for other participants, the surveys sometimes interrupted sensitive moments with others:

I think the other times it really caused me major issues was typical marital stuff. If my husband and I are arguing or getting into it and my phone goes off, he got to know the notifications, so then he’s going, “Just deal with,” and I’m like, “It can wait, we need to deal with this. It’s more important.” That did happen a couple of times.

9248, female; aged 29 years

### Theme 2: I Answered the Surveys Quickly

Outside of not answering the survey, participants described a quicker response speed as the best way to manage the disruption of the survey. Due to the consistency of the survey items and responses, participants were able to answer the questions quickly to minimize the interruption burden while potentially providing accurate responses. This outcome was especially beneficial when the survey came at an inconvenient time:

I usually just try to do it as fast as possible, get it done with so it wouldn’t annoy me again.

9270, female; aged 26 years

Because the surveys would basically be the same questions, I can answer the surveys in 15, 20 seconds because it’s just the same answers and it’s the same questions. I’d be able to do it fast.

9294, male; aged 21 years

With the phone surveys, with the questions being in the same order, that helped a lot because at the start of it, I hear then get the notification go and I could run through my head real quick how I was doing since the last survey, over the last hour, so I could already have that in mind. It would allow me to, if needed, to split my attention, but still be able to accurately answer the phone questions.

9248, female; aged 28 years

Response speed is often used as an indicator of IR by researchers, and participants did discuss how responding quickly may have been an indicator that the responses were less accurate or a sign that the participant was distracted:

I feel like during the finals week answering surveys it’s not fun at all. That’s the time when I probably didn’t have as accurate answers because I was like, “I need to get through this real quick and keep working.”

9302, female; aged 18 years

I guess maybe sometimes. If I’m really busy, maybe I’ll just skim through it faster. Maybe like in the middle of the day, I’m working and I want to do it fast, I may not take as long. It’s more of a reflex of answering those questions.

9318, female; aged 23 years

However, fast response speed is not necessarily always a sign of poor data quality. Many participants also described beginning to respond more quickly over time as an expected response pattern due to becoming more familiar with the study procedures and the questions:

Usually, it took, I don’t know, maybe a minute to get through. After the first couple, you know what the questions are going to be so it’s easier to go through faster and, yes, just knock it out essentially and then wait for the next one... I feel like answering the questions probably got a little bit easier because I knew what they were and who gauge better, like in the beginning you think a little bit and you’re like, how am I feeling? Then it just becomes like second nature almost to think about it and to go through and you’re like, oh yes, I’m a little bit tired or, yes, I have been procrastinating today. Yes, since you’re thinking about it, you know the levels a little bit better, so you’re able to—I was able to, I think, answer more accurately the longer I was in it just because of that.

9329, female; aged 23 years

### Theme 3: The Repetition Made Me Start to Pick Neutral Responses

Being in a study with repeated surveys had some benefits, such as reducing burden due to the repetitive and consistent survey questions and habitual prompting schedule, but it also introduced fatigue for the study participants. One participant emphasized the double-edged nature of the study’s EMA protocol design:

The factors come from the study. I think I know that it’s repetitive. Sometimes it’s daunting because it’s repetitive but sometimes it’s efficient because it’s repetitive.

9320, female; aged 28 years

Participants sometimes felt like they did not have sufficient time or cognitive effort to process the questions, and later in the study, they may have stopped taking the time to fully process how they were feeling once the novelty of the study wore off. This may have resulted in a preference for selecting the middle or neutral response option:

Fatigue, energetic for me is not that hard, but some other questions can be more subtle, like “Am I really feeling relaxed or not?” You know those kind of things and I would not get enough time to really properly answer those because I’m not sure what the answer to those questions are. “Am I tense? Am I not tense?” I don’t know at certain times, especially when I have this deadline to respond right away.

9310, male; aged 27 years

I feel like when it would ask me, “Oh, are you feeling this, are you feeling that?” Oftentimes, I’m not really actively thinking about how stressed I am, how tense I am, so oftentimes, I would just click the middle option, because I’m not really strongly feeling anything.

9314, female; aged 27 years

Indicating a neutral response more regularly may have also been due to the length of the study. By the end of the 12-month period, study participants found themselves feeling neutral emotions more frequently in comparison to extreme positive and negative emotions that they may have been more likely to experience as the study progressed. Given the frequency of experiencing relatively neutral emotions, being able to indicate the feeling of strong emotion was refreshing:

I think there’s a degree to which almost everything is in the middle towards the last fourth maybe the last three months of just feeling almost everything I’m answering is within a certain little range...it felt everything sort of balanced each other out to just be moderate. I’m always feeling moderate or whatever. I don’t think it’s inaccurate though, but—There was a day where it was actually really nice to be like, “Yes, I am feeling extremely sad today.”

9331, female; aged 28 years

### Theme 4: I Fell Into a Consistent Rhythm, But the Attention Check Questions Helped

Over time, due to the habit of responding to burst surveys, sometimes participants got into a “flow” of tapping responses, likely the same response option repeatedly mindlessly, which may have resulted in poorer data quality. However, participants often spoke about being able to catch themselves in this pattern and trying to make sure the data were accurate:

I remember, for some time, I did not see the exercise and physical activity box. I did not click that for some time. Then at some point, I went through, and I was like, “Oh wow.”

9236, female; aged 24 years

You know, that has happened a few times when I would go back because I’m in the flow of hitting what I’m used to. I would go back, it’s like, “Wait, hang on.” I would say yes when I am distracted with work especially...I would call it 70% accurate instead of 100% accurate.

9287, female; aged 29 years

I guess if I was really busy but I had to answer a survey, then I would spend less time on each question. I don’t know how much that affected my responses because a lot of the times my responses were in the middle. If I felt like I answered a question incorrectly, I did go back and fix it. I did this every time. Even when I was doing it pretty fast, I think I would still go back for those.

9314, female; aged 27 years

I think I just got more used to the questions so less second-guessing “what are they asking for”...I know there were times when I caught myself like, “Wait, I didn’t read that, go back,” but I don’t think there was any rhyme or reason to that.

9265, female; aged 27 years

One participant made sure to emphasize that falling into an overly consistent rhythm was the opposite of her response pattern and described her process of thoughtfully selecting response options throughout the study:

I’d say mine were really accurate. I honestly probably put a little too much thought into it. I tend to be thoughtful and that’s probably why I avoided it more because I’m like, “Okay how am I really feeling?” I feel like a lot of my responses felt generally the same except when something did happen that day that was really different. Even though some of them felt contradictory, I’m like, “Oh, I’m a little focused, but I’m also a little fatigued.” I would say that I definitely didn’t just pick whatever, just to get all the way through. I would say they’re as accurate as I could possibly make them.

9270, female; aged 26 years

The ACQs that appeared randomly in 20% of burst surveys (eg, what color is the sky? blue, green, red, sixteen, and pliers) were mentioned as a good tool to break up this repetitiveness or throw off the participants’ usual pace. Participants were able to identify the purpose of these questions as a tool to verify if participants were paying attention, but they did not find them annoying and sometimes found them amusing:

I love them. Sometimes I had to go back and like, because I was anticipating a different question, I’m like, wait, no, that was not the question. I would go back and then select the correct one. [laughs]

9287, female; aged 29 years

It seemed like maybe they were there to keep people focused and not just like tapping through the questions, which was nice, but I did feel sometimes because I knew what the questions were going to be and like, I was going through them, especially on the burst periods, you get one like that and it breaks my routine going through it and I was like, oh, okay then you got to reset.

9329, female; aged 23 years

I thought they were semi-amusing. Like, “Oh are they trying to make sure I’m not just going through the motions?” They were mildly amusing I guess is how I would describe them. Not a big burden to have one more. “Okay, something different.”

9270, female; aged 26 years

### Theme 5: Different Motivations For Answering Surveys Could Result in Different Levels of Data Quality

Monetary compensation was described as the primary motivating factor for most participants to continue the study. They were particularly aware of the minimum response rate of 8 surveys per day for compensation and put in the effort to reach this threshold. It is possible that this drive may have led participants to take shortcuts to complete the surveys:

Sometimes I knew that minimum I have to answer eight. I used to make sure that in the morning when I’m at home, I’ll keep my phone with me just so that I can answer at least eight. If I’m home the whole day, sometimes I used to answer even more. After the eight were done, it at least mentally, I was like, “Okay, I’m done now”...I wouldn’t have to be consciously looking at my phone all the time.

9287, female; aged 29 years

If it was a day where there’s no way that I get anywhere close to the eight, I would just not bother. On the flip side, if there was a day when it’s really close to having those eight surveys, then I would try to make an effort to get from seven to eight.

9245, female; aged 28 years

Oh, definitely while cooking. There were a couple of times when I knew that I still needed to do a few more for the minimum eight surveys that I needed to answer. I would answer with half-greasy hands.

9314, female; aged 27 years

[T]here was a little stress trying to hit the eight surveys a day so I always wanted to make sure any opportunity I get I could try to answer them because I don’t know why I would just miss one or two, but I’d always be like one or two shy and I’d be in despair.

9271, female; aged 23 years

Beyond monetary compensation, some participants felt responsibility and external accountability to the study after the protocol became a habit. Others mentioned the study as a positive aspect of their lives and emotionally rewarding. These internal sources of motivation could have increased the quality of data collected:

I felt a responsibility towards the study to answer questions. Obviously, the money incentive is a thing too. I think that’s in the back of my head though. I didn’t say, “Oh, I’m going to not make money if I don’t do this.” I think for the most part, in reality, it was just a habit. Over time, it became a habit and a sense of responsibility to this study. I just felt like I wanted to, so I did.

9307, male; aged 25 years

I actually think that as the study wore on, I was more able to answer the questions and the survey properly because it was easier for me to gauge how I was feeling. I am thinking about certain things just because I was more practiced at it of like checking, doing the whole mental check-ins every hour of, am I actually stressed? Am I frustrated? Or am I nervous or am I tense? Distinguishing between those things was a nice skill, I guess. [chuckles]

9296, female; aged 25 years

It helped me to reflect on how I was feeling. I did actually like that aspect of it, making me stop and think and be a bit more mindful throughout the day of how I was doing. Also, it helped me to realize a lot more how active I actually am. That definitely has had a very positive impact.

9248, female; aged 29 years

I used to take a moment and think about what I am feeling in the moment to answer the questions correctly, which has been pretty helpful in tethering myself, very emotionally rewarding almost if it makes sense. Reminder to slow down.

9266, female; aged 21 years

I looked forward to something to do and also it was very simple and also in a small way, it was like having a buddy [chuckles] checking on you. How are you feeling? Are you planning to exercise? Do you sit? Do you eat? It reminded me to not just stay stationary. I think it just encouraged me to think about things I wouldn’t always think about.

9271, female; aged 23 years

## Discussion

### Principal Findings

Reducing IR in future intensive longitudinal EMA studies extending over several months will depend on a better understanding of participants’ experiences. It will be important to accept that participants are active contributors in research and, in turn, consider the complex nature of the relationships between participant and researcher in the design of EMA studies. Improving engagement is a universal concern across all digital health tools. A high rate of disengagement is frequently reported as a barrier to the uptake of mobile health tracking apps where high-quality user experience and adherence to app use often underlies the success of the interventions [30]. This study contributes to limited research examining participants’ perceptions of the quality of the data provided in an intensive longitudinal health behavior study. As one of the first studies that collected EMA data intensively over a 12-month period, the TIME study was a novel avenue to examine the prevalence of IR and the potential application of ACQs as both a method to validate momentary attention as well as a potential intervention to improve data quality.

Thematic analysis revealed 5 overarching themes that shed light on the factors influencing IR in ILD studies that use EMA. Findings suggest the importance of social context and the presence of understanding and accommodating individuals in their social circle, which could alleviate the perceived burden of participating in an intensive longitudinal EMA study over several months. Quick response times can be attributed to time constraints, distraction, or a desire to complete the surveys quickly to avoid the disruption of daily activities. The findings suggest that optimizing the survey length and minimizing disruptions could reduce IR. The third theme further highlighted the dynamic nature of participants’ response patterns over time. Participants reported external factors such as mood, stress, and daily routines influencing their willingness to respond accurately and thoughtfully. This insight underscores the importance of considering and potentially accommodating these fluctuations in study design and analysis. This could include items to measure potential confounders such as mood or stress or potentially allowing participants to snooze time-to-respond to reduce the burden of completing a survey at an inconvenient time. Furthermore, participants generally recognized the ACQs as a technique that was used to reduce IR; thus, reactivity to these questions could potentially be a tool for researchers. Moreover, monetary incentives alone were not the only driving force behind participants’ sustained engagement. Instead, participants expressed a sense of curiosity, personal interest, and a desire to contribute to scientific knowledge as important motivations for their continued participation in the EMA study. Understanding these intrinsic motivators can inform future study recruitment strategies and participant retention efforts. In addition, an effort can be made to cater to these intrinsic motivations rather than focusing solely on the monetary component, especially in future longitudinal studies using EMA.

The findings from this study align well with those of the previous quantitative studies examining factors underlying compliance to EMA studies. Our results indicate that the social context is an important factor in young adults’ routines, and the burden associated with an EMA study may need to expand to the disruption of others around study participants. A review by van Roekel et al [31] found that participants perceived surveys to be more inconvenient in public places. Participants’ descriptions of changes in response patterns toward quicker responses and decreased variability are consistent with previous research [32,33], but this study sheds light on reasons these patterns begin and suggests that these commonly assumed indicators of IR may actually be normal shifts over time. Many participants reported becoming more habituated to the measures as the study went on, which has been previously reported [34]. However, our previous analyses suggest that response speed declines across the study period were not significant. When examining the predictors of completion speed for the first 14 EMA surveys from this study, results of a linear mixed model indicated that response speed was slower for prompts completed in the evening (B=1.05, 95% CI 1.02-1.08) and reprompted surveys (B=1.22, 95% CI 1.19-1.26), but there was no effect of day in the study [35].

The results were also consistent with previous qualitative studies that examined accuracy in EMA studies. A 2-week EMA study conducted by van Berkel [27] found that participants attributed their current mental state as the biggest influence on the accuracy of answers, with tiredness, distraction, and low concentration as negative factors. In addition, Eisele [26] had participants in a 14-day study describe a stabilization effect, with some participants reporting how the initial excitement wore off and the questions were boring and others reporting becoming familiar with the routine of surveys. Participants in that study also reported changes in response patterns, with 56% reporting an increase in habitual survey responses over time, which included reports of learning the order of questions and increased familiarity with the questions over time, thus leading to easier and faster responses. In the same study, participants also reported higher awareness of their emotions over time due to repeated assessments, which may suggest reactivity. The researchers further matched participants’ interview responses with quantitative data that showed decreases in response variability during the study period. Participants in the study by Eisele [26] did not report changing their behavior or routines to avoid missing assessments, but this may have potentially been due to the shorter study duration of only 2 weeks. Given the similar theme of the qualitative data collected, a future direction may be to further examine the response variability of the TIME participants whom we interviewed.

A major contribution of this study is the feedback that participants provided on the ACQs demonstrating acceptability; none of the participants mentioned any major concerns about the items. In a recent paper that addresses IR in EMA studies, Welling et al [30] discuss that while ACQs have been effective in detecting IR in cross-sectional studies, participants in EMA studies answer repeated surveys and will begin to recognize the items over time and “possibly be annoyed or insulted by the apparent distrust in their responding behavior.” Similar reactivity to these types of questions has been proposed by researchers, where participants may start to look out for the items [30,36]. However, through the interviews, it seems there was positive reactivity reported where the attention checks made participants more aware that their accuracy was being monitored, which may have increased data quality. Due to the lack of expressed concerns and reported potential benefits, it is recommended that researchers include attention checks in future EMA studies, although this may increase survey length or response time. When designing an EMA study, researchers must make many decisions, and optimal EMA study design is highly dependent on the study research question, study population, and available research infrastructure. Given that EMA studies are burdensome for researchers in terms of cost and time, enhancing data quality should be a priority for researchers. Future studies may consider using phone use data and contextual information to explore ways to infer participants’ ability to answer an EMA survey. Given the participants’ discussion of the repetitive nature of the surveys, researchers should aim to diversify the types of answer options commonly used in EMA or randomize the order of questions. In addition, resources to reduce researcher burden in detecting IR should be developed. In an ideal study, researchers would monitor data integrity the same way that many studies currently check compliance rates. If programs that can automatically screen for IR can be developed, dashboards could be created for investigators to use, and emails or SMS text messages could be automatically sent to participants about responding to their surveys more thoughtfully. Finally, the orientation session is a critical element for researchers to use to build rapport with participants and discuss the purpose of the study. This time could be used to stress the importance of high data quantity and quality and discuss the problem of missing data and inattentive responses. This session could also be used as an opportunity to familiarize participants with the EMA items and response scales of the study and how to use them. Further research should be conducted to examine how participants’ use of response scales changes over time or in response to extreme events.

### Limitations

This study is not without limitations. First, there may have been sampling bias. Overall, there are likely personality and demographic differences between participants who consent to participate in a 12-month intensive longitudinal study and the general population. Our study may have had a lower prevalence of IR compared to other EMA studies due to participant motivation. Participants may have been more likely to skip surveys rather than answer inattentively. In addition, the qualitative sample of 31 participants who completed the study was significantly different from the overall study sample. In looking at the coding process, each code was not applicable to all participants and may have skewed the number of codes applied per transcript. Participants generally reported having a positive experience with the study, but interviews were only conducted with participants who were all able to finish an extremely intensive study. The participants who had more negative experiences likely withdrew from the study. There may also have been reporting bias, as some opinions or information may have been actively withheld or suppressed by the participant to present a favorable impression to researchers. Despite much discussion on the repetitiveness of the surveys and lack of variability in responses, surprisingly, none of the participants proposed in interviews that the study could be shorter or complained that hourly surveys were too frequent. Moreover, given the length of the parent study, participants may have experienced recall bias trying to recall feelings about aspects of the study across the year and trying to describe their own experiences. Anecdotally, some of the interviews were less informative because it was difficult for the interviewer to get participants to open up about their experiences, and some of the transcripts had brief responses. However, even with less informative interviews, existing sample size guidelines suggest that a range between 20 and 30 interviews is adequate for qualitative interview protocols such as the one used in this study [37]. Since our study population was well-defined, it can be assumed that the phenomenon of IR is likely homogenous across participants in our study, which resulted in reaching theoretical saturation (no new information is emerging in each category) for our focused research aims even with fewer participants.

### Conclusions

Overall, the results from this qualitative study provide insights into the complex process of IR in EMA studies. The identified themes offer practical implications for researchers, including the importance of social context and support, the consideration of dynamic motivations, the utility of ACQs, and the further exploration of potential intrinsic motivators for completing EMA. By incorporating these insights, researchers can improve participant engagement, enhance data quality, and maximize the scientific value of long-term EMA studies.

## Appendix

Multimedia Appendix 1 Transcripts from interviews.

Multimedia Appendix 2 Demographics data.

## Abbreviations

ACQ: attention check question
EMA: ecological momentary assessment
ILD: intensive longitudinal data
IR: inattentive responding
TIME: Temporal Influences on Movement and Exercise

## Data Availability

Multimedia Appendices 1 and 2 contain transcripts and demographic information of the participants. Additional participant questionnaire data from the Temporal Influences on Movement and Exercise (TIME) study can be requested from the corresponding author.
