# Supplementary material for: Burden and Inattentive Responding in a 12-Month Intensive Longitudinal Study: Interview Study Among Young Adults
Source: JMIR Form Res. 2024 Aug 2;8:e52165. doi: 10.2196/52165 (PMC11329843; doi:10.2196/52165)
Supplement: Multimedia Appendix 1 [file formative_v8i1e52165_app1.zip › Transcripts/unraveledlitterpowerably_audio_8.9.22.m4a.docx]

**Interviewer:** To start, can you provide me with some of your overall feedback regarding the study?

**Interviewee:** Overall it was good, it was just a bit annoying with it was a busy day and we had to do the first surveys. I understand that I had to answer them, so I did my best trying to do that but there was no problems with it or with using the app or the watch.

**Interviewer:** Okay. That's good. I'm going to ask more specific questions about your experience. If a question is ever unclear, please feel free to ask me for clarification. Let's start a little bit more about participating in the study in general. The first question I have is, how did you learn about the study? I know it's been a year, so it's been a while.

**Interviewee:** I used researchmatch.org and that's how I was able to find it.

**Interviewer:** Got it. Do you remember what aspects of the study interested you?

**Interviewee:** It was being a diabetic. Because it was like a sleep study. At the moment, I just wanted to just see whatever I could do to just help the diabetes community then.

**Interviewer:** That's great. Can you describe to us what motivated you to continue to answer surveys in the study?

**Interviewee:** Finishing the study was my main goal because I knew it's really, really long, a year-long study.

**Interviewer:** Yes, it's long.

**Interviewee:** Yes, and then I would get compensated as well.

**Interviewer:** It was important to have compensation in the study.

**Interviewee:** Yes. Right now because I'm not working, so that was whatever I could get.

**Interviewer:** Yes, definitely. Can you describe the process of answering phone surveys on a typical birthday? I know you mentioned those days were a little bit busy or it was hard if it was on a busy day, but can you tell me about the typical day of answering surveys on the first day?

**Interviewee:** When I first started the study, I was working, so I couldn't get to all to answer all the first surveys. Then afterwards I wasn't working, so I had more time to answer them. There's not much to say about them, but yes, I just tried my best to answer them.

**Interviewer:** Did you have a goal number of surveys that you tried to reach each day?

**Interviewee:** I don't think I did but I tried to answer as many as I can.

**Interviewer:** Let's see. Did you ever track completion? Ever look on the app to show you how many you've done so far for the day?

**Interviewee:** Yes. Oh, I did remember seeing I had to complete at least eight I believe. I try to go reach that goal or pass that.

**Interviewer:** What would've made participation in the study more fun or rewarding for you as a participant? Besides paying more? That would be more rewarding of course, but anything else?

**Interviewee:** I don't think there would be anything. It was great overall, I would say.

**Interviewer:** Okay. For this next section of questions, I want to learn a little bit more about situations of increased burden that the time study may have caused. We know participating in the study may not have been easy at times and so we want to learn a little bit about the challenges that you may have experienced while in the study. What were some situations in which it was particularly challenging to answer surveys?

**Interviewee:** Like I said in the beginning, like work-wise. Being at work, I do more hands-on things, so I was a chiropractor assistant, so I was always using a massage gun. I couldn't just let it go and then answer surveys or be on my watch at times. I would say that would be the only thing that would get in the way.

**Interviewer:** What part of the app or procedures of the study was most disruptive? Like was it the sound, was it the vibration? Was it taking time to do the survey?

**Interviewee:** No, the surveys were pretty quick, but no, I don't think I had a problem with me other than what I just mentioned.

**Interviewer:** Okay. Also, I'm sorry if you hear drilling outside. I don't know what they're doing out there, but if it's loud, I apologize.

**Interviewee:** No, that's fine.

**Interviewer:** You're much luckier to have a rooster in the background than whatever they're doing over there. What most frequently led you to be unable to or to miss answering phone surveys?

**Interviewee:** Work. I think that's the only thing, yes.

**Interviewer:** Did you ever prefer to dismiss a survey? If you saw it and you're like, eh, no.

**Interviewee:** There were times that I felt that way, but, no, I never did.

**Interviewer:** Never actually did it.

**Interviewee:** No.

**Interviewer:** That's dedication. That's pretty good. What did you typically tell friends or family or coworkers if they asked about the study?

**Interviewee:** My family and friends would ask about it because I would be with them sometimes and I would have to answer it or they think that I'm just on my phone not paying attention to them. I would tell them about the study and why I was doing it. It was a brief summary that I would just tell them that's first **[unintelligible 00:06:35]** study and all that.

**Interviewer:** Okay. For these last set of questions here I want to learn a little bit more about response accuracy. Besides not answering or being unable to answer, I'm curious if there were other ways that you dealt with maybe some challenges or burdens while being in this study. How did you typically handle distractions when taking a survey?

**Interviewee:** I was distracted at some times, so I would just put it aside for a minute, and knowing that I had in a few minutes, it would remind me again to take the survey, that was really helpful. I would finish up what I was doing and then go back to it once it sent me a notification again to complete the survey.

**Interviewer:** Yes. Were there situations in which your responses may have been less accurate? Like maybe you weren't totally thinking through your responses, you just answered them.

**Interviewee:** No, not at all.

**Interviewer:** Okay. Do you think your responses changed like morning versus evening or if you were around certain people?

**Interviewee:** They did change throughout the day and not around certain people, but throughout the day they would.

**Interviewer:** Yes. In what way?

**Interviewee:** I would be stressed like waking up in the morning or relaxed and then stressed by the end of the day or vice versa. So at the moment when the watch would ask me if I was like sleeping well or stressed or if I'm excited or happy, I would answer it, but then when the in-the-evening survey would come, it would ask me if I was sad or I forgot when then I would probably say not at all, even though it would just a few minutes of during the day that I would feel that type of way.

**Interviewer:** You base it more so for like the end of the day survey, like more so an average of the day, not like one specific moment.

**Interviewee:** Yes.

**Interviewer:** Okay. How do you think your motivation or accuracy changed as you were in the study longer?

**Interviewee:** I was more dedicated throughout, in the middle of the study and at the end because I was seeing how I was being compensated so that was a factor.

**Interviewer:** Absolutely. What made the study easier or harder the longer you were in it?

**Interviewee:** I wouldn't say it was easy but what made it easier was having the watch and so you could briefly answer the questions instead of like, if it was on a phone, take your phone out, unlocking it, and answering and stuff like that.

**Interviewer:** The convenience of the watch.

**Interviewee:** Yes.

**Interviewer:** Let's see. Okay, last question here. What did you think about the questions and messages that were not related to measuring either health behavior, routines, or moods that came up on the phone?

**Interviewee:** Oh, those were easy to answer.

**Interviewer:** Were any of them memorable?

**Interviewee:** Like which one of these is the president or something.

**Interviewer:** Yes. Do you have any suggestions for us on how to make those ones better?

**Interviewee:** I think those are fine. I wouldn't change them or anything. There was although one question that I did get confused on. I really didn't understand what it was trying to say. I was like, if I answered this wrong, they probably think I'm not paying attention or anything. That was that, it just happened once and throughout the whole study.

**Interviewer:** Do you remember what question it was?

**Interviewee:** I'm not sure.

**Interviewer:** There are so many that came up. Are there any other points that we didn't cover that you would like to discuss? Maybe something that came up.

**Interviewee:** No, well, I don't think there's anything wrong with it, but on the watch, it would ask, are you with friends or family? In the beginning, I didn't know what it was trying to say because it didn't completely finish the whole question as the [crosstalk]

**Interviewer:** It didn't show it all the way.

**Interviewee:** Yes. After a while, I did realize what it did say so it wasn't a problem afterwards but that same thing.

**Interviewer:** It got cut off on those ones. For some of the longer ones, they seem to get cut off. You still answered those then, even if it was cut off?

**Interviewee:** Yes.

**Interviewer:** Okay.

**[00:11:54] [END OF AUDIO]**
