# Supplementary material for: Burden and Inattentive Responding in a 12-Month Intensive Longitudinal Study: Interview Study Among Young Adults
Source: JMIR Form Res. 2024 Aug 2;8:e52165. doi: 10.2196/52165 (PMC11329843; doi:10.2196/52165)
Supplement: Multimedia Appendix 1 [file formative_v8i1e52165_app1.zip › Transcripts/persevereriseswoop_transcript_4.29.22.docx]

File: Interviewee:_transcript_4.29.22

---

Interviewer: For this first part, we kind of want to know a little bit about your intent to join the study, why you decided to join the study, and what motivated you, whether motivation in response patterns change throughout the study and so first we want to hear about how did you hear about some study.

Interviewee: I heard that the study I think through [ ]. I get like emails to my inbox about like studies and so I found it through that email list and I decided to do it because it was longer term and it just sounded a little bit more interesting and something that I could pretty easily do especially while working from home.

Interviewer: Can you describe to us what motivated you to continue answering surveys in the study, because we obviously know it was a lot. What kept you going in this study?

Interviewee: I think, of course, kind of an obvious one was the compensation. But also it really did become part of my routine and I remember at the beginning, someone saying like it kind of becomes part of your routine and it really did it was kind of just funny like sometimes like i've been playing games with friends and i'll be like Oh, we have to pause it's it's survey time and they got it. They just understood and it became part of our thing so part of me is like it's been a weird and i'm probably going to get phantom pings on my wrist. So I think that was probably what kept me motivated was it became part of my routine and then just feeling like kind of I guess gratified at the completion of okay completed that many surveys and stay are answered that many questions, etc.

Interviewer: You mentioned telling friends about the survey time. What did you tell like friends and family in the beginning when you like join the study.

Interviewee: I told them that I was joining a study and be asked questions like every day. And a lot of times they would ask what's the study about and like what they are studying and I'd be like, to be honest, I don't remember. I say just asked a lot about like mood and activity and stuff like that, and just about me and I tell them who doesn't love answering questions about themselves. A lot of them are like well how do I get involved, I was like well you all can't you have iphones.

Interviewer: I know it's hard. I wish we were able to do with iphones it would open it up to a lot more people. Can you tell me about like how easy or difficult was it to answer the surveys on the watch?

Interviewee: Yeah it was pretty easy for the most part, I would say, sometimes the questions on the watch would get a little bit cut off just because it's a smaller screen and interface, and so, sometimes I would think I know what the entire question is but sometimes there's like ones that were a little bit longer that were cut off. But for the most part they were easy to understand, especially in the last few months, I just know I can anticipate pretty easily what the next question is going to be and how i'm going to answer it. Yeah it became…it was pretty easy.

Interviewer: So, speaking of burst periods, can you tell me your process of answering the phone surveys on a typical burst day like how many do you think you answered on a typical day or like did you set a goal for it?

Interviewee: Yeah, I always set a goal for at least 11 and I pretty much always went above 11. I think the past couple months I just struggling a little bit meeting it. But yeah for the most part, I try to set a goal for at least 11.

Interviewer: And, can you tell me a little bit about why it's been a little bit harder to answer them in the past couple months?

Interviewee: It's kind of hard to figure out exactly why it was more difficult, I think some of it was I just didn't have my phone on me all the time and so it would feel like i'd be waiting and i'd be here, and then I go to the bathroom and come back and say I missed the survey and so that happened to me a lot when I wouldn't have my phone on me or our sometimes it would happen because maybe I slept in later than I expected to, and so I missed a couple of surveys because I wanted to wake up earlier and I didn't. Or a lot of times, if I was working….meeting with someone for my job…my meeting so i'm not really able to do on time.

Interviewer: Did you track your completion on the app? Do you ever go and look and see how many completed for the day or keep track of that?

Interviewee: Yeah, I was looking at the notification in the menu and see how many I completed today.

Interviewer: Do you know of anything that would have made participation in this study more fun or rewarding besides paying more?

Interviewee: I can't really think of anything I think around Christmas there was an email that was like happy holidays and had some info about how many people were in the study and how many completed it. That kind of information was nice because it was really just brief but it was also nice to see like “people are completing it and it is going to be over”.

Interviewer: You should be getting another one of those emails like today. When you finish the study we will still send you newsletters or anything that comes out.

Interviewer: Okay, so for this next section we want to talk about situations where it was an increased burden to you. So we know that obviously participating in this study is not easy, and of course we appreciate you participating. So we want to learn more about challenges that you may have experienced from being in a study for a year. And so, can you tell me about situations you kind of mentioned like if you left and you weren't able to be near your phone or if you were sleeping in but were there any other situations in which it was particularly challenging to answer the surveys?

Interviewee: When I was in a meeting or if I was like playing a game really intensely and couldn't leave I would actually give the phone to my fiancé and say can you help me ask these questions and the answers because my hands are busy. And of course, while driving if I was alone in the car, I will just ignore it, so that means I miss out on it, but if my fiancé was with me and he was in the passenger seat, I give him the phone and asked him to ask me the questions. Those are really kind of the only instances, I can think of. Or if I was working out and didn’t have my phone right on me or I went for a swim or something like that.

Interviewer: What part of the app, it can be the watch and the phone, was the most disruptive or challenging to deal with? Or any procedures that were part of the study.

Interviewee: I don't know if there's anything that was super disruptive. More frustrating, I think the only thing was just the longer questions on the watch screen like I mentioned getting cut off. Or the questions on the watch feeling like repetitive. Sometimes it just felt like it asked me the same question three or four times in a day and I’m like I don't know how to answer it anymore.

Interviewer: For this next section of questions, I want to know a little bit more about response accuracy. So besides not answering if you were busy, we're kind of curious if there are other ways that you dealt with some of the challenges or burdens and the accuracy of your responses during that time. So, how did you typically handle distractions when taking the survey. I know you mentioned like your fiance would answer them for you.

Interviewee: When I was in the middle of doing something it would sometimes be difficult to get back into whatever I was doing before and sometimes it was a welcome break like maybe I was like doing that kind of work and doing a ton of emails and then oh hey I have a little bit of a break from that to answer the survey, but especially days, where maybe I was already having a hard time focusing and just feels like I keep getting distracted.

Interviewer: And were there any situations where your responses to the surveys may have been a little bit less accurate like if you going through and not really thinking about the answers.

Interviewee: Maybe like situations where the survey was really interrupting or disturbing, and so I feel like I kind of want to like get through it. The Sunday surveys at least, they are so much longer now, it is always a little bit of a rough one. I think, just when the surveys were like longer.

Interviewer: And how did your responses change if someone else was around or if there was if it was like a different location or time of day.

Interviewee: Yeah if someone else was around I think I still answered honestly but I'd be extra vigilant that people don't look at my phone. And same thing for the times that I do travel to my work’s actual office. That felt a little weird because I don't want to seem like i'm just on my phone all the time. I think, for the most part, I was still able to answer pretty honestly irregardless of who I was around or, where I was.

Interviewer: Okay last big question, and this is related overall to both the watch and the phone. Did you notice any of the questions and messages that were not related to measuring health behaviors, routines, mood on the phone or the watch?

Interviewee: Yeah they're definitely sprinkled in there and I thought they were always kind of funny at the beginning. I wanted to answer them incorrectly to be funny… screen people to see that they're actually paying attention with questions. I shouldn't do that… I thought they were kind of funny.

Interviewer: Were there any that were more memorable to you, that stood out to you?

Interviewee: Like not specific ones i'm thinking off the top of my head … not really any specific ones kind of just any of the ones where it's like which one is not a US President and like a Bamboo tree or something right?

Interviewer: Before I switch totally topics here, are there any points or topics that we didn't discuss that you wanted to tell us about that maybe came up in the study?

Interviewee: I guess the only thing. especially in the beginning, answering questions about like mood and stuff and my answering those like big intake kind of series and six months surveyed like…. since I am some of the mental illness and so answering those on like wow I really hope you don't like or even like reach out… not even like a concern or something that stressed out or word, he is something that I thought was kind of funny like la like what are they gonna think about this, or like are you going to like reach out to me and recommend resources.

Interviewer: Last question for here, what are you going to miss most about study?

Interviewee: it's definitely going to feel weird…. that becomes so part of my daily routine and sometimes my hourly routine. I think it's going to be, you know interesting because my friends will be like will now be like away from my desk and they'll be like, are you answering survey i'm like you know, surprisingly, not. So it's gonna I think the only thing i'm going to kind of miss it's like using it as like I guess an excuse to get out of certain social situations like if you don't want to talk someone are going to stop conversation oh sorry I have…

Interviewer: Last question that I have for you um do you have any questions for me about the research that is being conducted.

Interviewee: I’m just curious about what all the data is going to be used for.

Interviewer: So the kind of the purpose of the study is we're trying to see how young adults so 18 to 30. How their health behaviors change over a year, naturally, so typically when you like do a survey, for instance. Typically, when you do a study you will maybe reflect back on like the past month, so you'll see like have you done physical activity or have you how did you feel during physical activity. You know, a month ago, or in the past month, and you will reflect your answers, there were here, as you know, we're asking things in real time and so we're trying to see if any if there are any changes over a year, naturally. You know most research shows big things like if you find physical activity rewarding and then, if that affects how much do. were kind of interested in like the day to day factors of it and look at seeing if there's any trends, obviously the pandemic kind of threw an interesting wrench in it all. You know there's that factor into it as well, because everyone was kind of forced to stay home for a while, or Walker and some States longer than others um. But yeah I mean that's kind of the overall what we want to do with the data we obviously have a lot of data we collect a lot of data so there'll be some really interesting papers that will come out of it, and we are giving you some of the research..results that we have found in this next newsletter but, at the end when everyone is done and we'll be able to hopefully share some more with you guys and we'll send out an email to you all.

Interviewee: On average, how many people I do a lot of people drop out after a certain period of time.

Interviewer: um, it is a year long so that is a long time for a lot of people in a lot of people did join at the beginning of the pandemic when they're like i'm home, I have nothing else to do and and they started going back to school and going back into jobs. And so we've actually had a really good retention rate, but you know, there are a lot that did drop out because of that, where it was like hey I got a job and I can't like in person and I can't be on my phone or I can't be looking at my watch every five seconds.
