# Supplementary material for: Burden and Inattentive Responding in a 12-Month Intensive Longitudinal Study: Interview Study Among Young Adults
Source: JMIR Form Res. 2024 Aug 2;8:e52165. doi: 10.2196/52165 (PMC11329843; doi:10.2196/52165)
Supplement: Multimedia Appendix 1 [file formative_v8i1e52165_app1.zip › Transcripts/copybrickcreative_audio_8.29.22.m4a.docx]

**Interviewer:** To start can you provide me with some of your overall general feedback?

**Interviewee:** Yes. I think it’s interesting because I started the study before I got into grad school or before I knew what that would look like. The first few months I was better at hitting the marks than I ended up being overall, and just like, “Oh, crap.” I was a little worried about that over time, but then as time progressed, because the consent form it seemed a little more rigid on the protocols on which I would continue being a part of the study. Really trying to meet those as best as possible.

Then just be like, “I don’t know. It’s six months in. I guess, I’m still in,” [laughs] even though I haven’t been as high as I’ve wanted or you all seemed to want in the beginning, and so sort of uncertainty of like did the benchmarks change? [crosstalk]

**Interviewer:** Honestly, you did great throughout the study. You really did. You were consistent throughout. It was not bad at all. Don’t worry.

**Interviewee:** Because there were some days I was like, “Crap, I forgot my charger or whatever.”

**Interviewer:** You did great.

**Interviewee:** Some days it was just like, “Too bad I got to study for this test.” Over time I did balance it out a little better because I am very rule oriented myself and so I was like, “I got to do the thing.” Then I was like, “No, okay.”

Today I just got to do this other thing and so watch is getting muted all day so be it. I think overall the burst periods weren’t too bad. It was frustrating because some days I was at work or school and then I had my phone on vibrate because I couldn’t have it make noise.

Then I didn’t always feel the vibration and so that was just annoying. Sometimes the time periods was frustrating because it was like, “Oh, my break is for classes at this time. If they were more consistent or maybe if I could click on it once per hour or something then I could have just still gotten one in for those three hours versus missing all three or even sometimes four.

That was harder. I also wonder. Sometimes I felt like the location surveys, I feel like I got very few of those and it’s probably just because I have my phone on mute most of the time, and so it’s like, “Wait, do I just not get a lot of those?” Or I assumed that once I put in where somewhere was it was recorded as, “Oh, they’re in this place now,” but then sometimes I would get asked again and so I was like, “Maybe.” There’s some thoughts about how it worked.

**Interviewer:** At the end of the questions I’ll share my screen with you, part of this exit interview. I’ll share the spots of your frequently visited spots or maps of your frequently spots. If there’s any that were missed, you’ll get the chance to redeem and tell us where they are. It’s fun if you could just see a trip on memory lane.

**Interviewee:** That’s also confusing because sometimes it was like, “Are you with friends?” On the survey, burst period survey it has an option for spouse or partner and so I designated my partner as that option, but then it never asked about that on here. Sometimes it was like, “Are you with friends?” I’m like, “No.” “Are you with family?” “No,” because you’re not married.

**Interviewer:** That’s hard.

**Interviewee:** Then other times it was like, “Oh, I went to therapy. Who were you last with? Who were you with over the hour?” I’m like, “Acquaint, friend. I don’t know.” Become like, “What are they? What do I call them?” Sometimes the categories didn’t, but the watch was annoying but I got used to it. Interestingly at the beginning I’m pretty sensitive physically and so I actually every few days had to switch wrists, because not the buzzing actually it seemed like whatever it does to track my stats made my arm feel weird. I would switch it over but then in the last seven months I have just kept it on my left arm.

**Interviewer:** Is it been okay? Have you noticed it?

**Interviewee:** Yes, no problems. It was just like I just had to get used to it. That was interesting. That’s overall.

**Interviewer:** If anything comes up again or if--

**Interviewee:** Probably will.

**Interviewer:** Please let me know. I’m going to ask a little bit more questions about your overall experience. First, I would like to know how did you learn about this study? Do you remember how you learned? I know it’s been hard.

**Interviewee:** Research match, I think.

**Interviewer:** Research match. Oh, go ahead.

**Interviewee:** It might have been a few months prior before I got into it that I heard about it through research match and I did the survey and then either I just didn’t hear anything or it said that I wasn’t qualified for it or I don’t know and then it ended up coming back around somehow. It was really cool because I know I was like 298 or something?

**Interviewer:** You’re-- [crosstalk]

**Interviewee:** One of the last few.

[laughter]

**Interviewer:** Do you remember what features about it made you want to join the study?

**Interviewee:** I do have to say it’s the only thing I’ve ever been asked about that was paid so that was definitely a component. It also just really interesting. It seemed pretty straightforward, like, “Oh, okay, you’re going to ask me questions” Cool. I do that.” I take surveys through research match fairly regularly and like, “Wait there’s this watch.” I don’t know. It did sound really interesting.

I’m not sure. It might have been my own self being like, “Oh, I didn’t qualify for that.” Because I don’t know if I do however many hours or minutes of exercise a week. I know that was one of the original qualifications in this study, and then over time I was like “Maybe I do, maybe I don’t. I have no idea.” [laughs]

**Interviewer:** I know some people have mentioned like did you find that being in the study made you more mindful of how much physical activity you did?

**Interviewee:** Somewhat, yes. It definitely played a part. It’s something I always strive to do more of and I wouldn’t say it went out of my way to do more of it. Maybe at the very beginning it was on my mind more and so it got me more like, “I’m going to take a walk.” It might have been a little more consistent, but then when grad school hit, it was like--

**Interviewer:** Yes, everything goes out the window in grad school. [laughs]

**Interviewee:** A little bit, yes. No, to be fair. I definitely at some point got into doing some exercises in the morning for a consistent period which I have been meaning to do for years. You know ebbs and flows and then I think it was always in the back of my mind being asked like, “Oh, what do you do?” Then also sometimes in the burst periods like the next hour I’d be like, “Wait, I did my stretches. I didn’t say I did physical activity.” That’s another piece of interpreting.

**Interviewer:** What count?

**Interviewee:** Unless I physically active I’m going to walk from my desk to the kitchen but I don’t think I would count that. That’s where making my own distinction on what counted was interesting over time as well.

**Interviewer:** Did you find that you stuck to what you chose in the beginning and that stuck to that throughout the study or did it change over time?

**Interviewee:** I’m mostly stuck to what I decided at the beginning, I think as far as I know.

**Interviewer:** What motivated you to continue answer surveys in the study? Obviously, grad school it’s hard. What kept you going?

**Interviewee:** I don’t know. I’m in grad school for counseling. I’m super into self-growth and self-awareness. I think that’s part of it was just being like, “Oh, yes, how am I feeling right now?” That was interesting to check in with having my own awareness increased by answering the surveys. Then also just the, I don’t think science and then obviously the money was helpful too. There’s always this like, “What are you guys going to do with all this?” I’m like, “What does this mean?” Just curious. Yes, it’s intriguing.

**Interviewer:** It’s a lot of data, obviously, a year’s worth of data, and the overall goal of most research has shown that big things like if you find physical activity rewarding, that’s going to affect how much you do. We’re looking at the little tiny day-to-day thing and how much that affects. That’s why hence all the questions that you’re getting asked, all the different emotions that you’re getting asked. Just how your health behaviors change over a year naturally, and being in grad school, that’s going to affect a lot of life things, and so changes naturally over a year. Yes, a lot of data.

**Interviewee:** Yes, **[unintelligible 00:10:51]** like, “I want to take a walk but I got to get this finished or whatever.” Definitely more sitting than I would like.

**Interviewer:** Can you describe the process of answering surveys on a typical burst day? What that was like for you?

**Interviewee:** Yes, most days the night before set my-- because I have my phone on airplane mode at night and also on silent, obviously. Setting it to turn off of silent and on to vibrate in the morning after, so it’s like, “Okay, I’m going to sleep for nine hours, cool.” Nine and a half hours after I go to sleep and have it turned back on vibrate automatically so I don’t forget. Then just going about my day and answering when they come, hopefully, I feel most of them or hear most of them. It was more difficult at work or at school.

At school I had to turn my phone silent and then at work, generally, I was more able to. I work in customer service, so some days it’s super slow and so it’s totally like, “Two minutes, great, no problem.” Other days it was harder to hit more of them, and it depended on-- [crosstalk]

**Interviewer:** Yes, very.

**Interviewee:** Super very. Then it was also frustrating because sometimes I would not hear or feel the buzz until the second prompt, like, “Hey, do the thing.” Then I would start it a little bit after that, and then- [crosstalk]

**Interviewer:** It would time out.

**Interviewee:** -half way through, I can be like, “No.” [laughs] It can be frustrating sometimes. Then also overtime figuring out there were some days that I changed the time that I was planning on going to bed in the app so that I could maybe squeeze another survey in to get that eight or 11. Because I know it was just one more and because it’s like, “Oh, I’m not going to bed for two hours, I’m going to be awake.” It’s not like it’s keeping me awake because it would cut off two hours before bed, sort of figuring that out, figuring the systems out sometimes.

**Interviewer:** Did you have a goal number then? I know you mentioned right now you try to get to eight or 11, but did you have a goal number that you generally try to answer each day?

**Interviewee:** Ideally 11, but that wasn’t always possible, obviously, because I didn’t get it every time. Sometimes that was just this mere fact. I sleep nine hours every night, and so that severely reduced. It’s like, “If I miss two, then that’s pretty much out the window.” Not always, though some days it ended up working out. Yes, that was just like, at first that was my like, “Yes, I got it.” Then at some point it reduced out of like, “Okay, I just have to get at least eight.” Some days I was like, “I only got four, but okay.” [crosstalk]

[laughter]

**Interviewer: [unintelligible 00:14:29]**

[laughter]

**Interviewer:** As the last one, though, it’s done.

**Interviewee:** Right. Yes. It was funny because it was both motivating and I had just started the semester.

**Interviewer:** For this next section of questions, I want to learn a little bit more about situations of increased burden. We know, obviously, the time study was not easy at times, and being in it could have been challenging, and so I want to learn a little bit more about those challenges. What were some situations in which it was particularly challenging? I know you mentioned being at work at times or in the classroom and whatnot. Is there anything that sticks out to you where it was like, this is just too hard to answer surveys?

**Interviewee:** I don’t know if this is actually an answer to your question, but I do remember one day that I was hiking, and my phone was in my pocket on vibrate, and I just didn’t feel it whatsoever because I was moving too much. I would check my phone and be like, “Oh, crap, I missed another one. How’s that happen?” Then that just consistently happened through the day. Then that was like, “Oh,” there was an unwillingness to have a noise interrupt me, interrupt my day.

That was something that prevented me from answering more that-- I maybe could have answered more had I been willing to have that noise. I almost never have anything on sound. Otherwise, really, the main ones are just, oh, okay-- I mentioned this in the type survey. Definitely work in school. Sometimes study days if I have a lot of reading or whatnot, that can be frustrating for my attention to be diverted for either phone or watch. Then there’s also, like, I cared less about the watch because I know you all weren’t-

**Interviewer:** Complicating **[unintelligible 00:16:47]**.

**Interviewee:** -expecting as much from that. I allowed myself to care less about that, which was nice, especially on days. That I feel is more disruptive because it’s more often, like when I’m reading or something, whereas it’s like, “Okay, time for the hourly survey.” That felt better. It was almost nice to take that break, be like, “Okay, right, there’s a world outside of this book.” [laughs] Oh, I’m hungry. I’m going to go get it back.

It’s actually nice to have that one. Whereas the watch ones, I actually found myself, there sometimes I would read it, press, and then be like, “Wait, what was the question?” That became my default. Often, I would be able to change it in time. It was probably maybe 3% of my answers because of habit rather than actual being sort of--

That was also, in general, it was interesting to see how the watch slipped in because even today there was a question it asked me and I answered it and then it disappeared. I was like, “Why didn’t it just ask me?” I don’t even know. I know I answered whatever was true, but it was just funny. [laughs]

**Interviewer:** Yes, it’s funny because every time I’ll ask if there’s a specific question that comes to mind, which I’ll ask you like, “Do any specific things come to mind?” Everyone’s always like, “I can’t think of any.” It’s been a whole year of answering questions, but it’s like they’ve gone in one year and out the other because it’s been so many questions asked.

You reminded me, though, and I forgot to mention this earlier, you can delete the app off of your phone and watch now. I had never been earlier, but I forgot to tell you so that way you won’t get prompted anymore and you’ll be done.

**Interviewee:** Okay.

[laughter]

**Interviewer:** Anyway, I’m sorry, didn’t mean to interrupt.

**Interviewee:** No, let’s see, any other times. Oh, and then I mentioned that this is done. I mentioned in the printed one of date nights and intimate times and--

**Interviewer: [inaudible 00:19:06]** [crosstalk]

**Interviewee:** It’s Sunday night, I don’t want to miss the week. The big week survey.

**Interviewer:** A long one.

**Interviewee:** Or I would turn my watch off on do not disturb, but that sort of thing.

**Interviewer:** Yes, that’s real, I think. Can you remember a time where you preferred to just dismiss a survey? If you saw it, you’re like, “No, dismiss not now,” instead of answering it.

**Interviewee:** Maybe if I happened to look at my phone for some other reason and notice that during class and noticed there was a survey, that would be one, but that’s the only thing I could-- or yes, school or work, noticing it during a time when I just can’t answer it. I don’t think there were any other ones. The watch felt less intrusive in work and school. I turned it off for school, but work like that, like nobody easy. Yes, nobody really cares, really knows what I’m doing.

**Interviewer:** Did you typically tell coworkers or family and friends? What would you typically tell them if they asked about this stuff?

**Interviewee:** I think I mostly did that at the beginning and mostly about burst periods because it didn’t take enough time. The surveys themselves didn’t take very long and so I would just do them when I was hanging out with someone.

I would tell my family, on a family trip, I had a burst period, just like, “Oh, hey, by the way, I’m doing this study. It’s pretty interesting. It’s about feelings and about movement and about tracking my stats and stuff and getting paid for it. It’s pretty **[unintelligible 00:21:26]** that was cool watch. It asked me surveys every hour for four periods and other days it asked me things on the watch.” They’re like, “Oh, okay, cool.”

**Interviewer:** They’re like, “Dang, would you want to be paid **[unintelligible 00:21:42]** survey?”

[laughter]

**Interviewee:** Fairly recently some of my friends were visiting from out of town and I explained it to them and they seem like, “That’s a lot.” They didn’t say that, but they seemed like, “Not really. I don’t know.”

**Interviewer:** It’s up to you, I guess.

**Interviewee:** Yes. I actually have a protocol. My partner and I have a protocol of showering when we get home from being at work or something because of COVID.

**Interviewer:** COVID, yes.

**Interviewee:** That was when I charged my watch, but on bust days I would turn my phone on sound and just be like, “Hey, it’s a bust day. Can you listen for my phone and answer the questions?” Oh, actually on a road trip too with a friend, it was during a burst period, so it was like, “Oh, here. Ask me the questions so you can answer them for me.”

**Interviewer:** That’s a good idea.

**Interviewee:** It was fun. It was interesting because it felt more vulnerable answering the questions. **[inaudible 00:22:46]** [crosstalk]

**Interviewer:** Totally.

**Interviewee:** Then it was like, “Oh, don’t read too much.”

[laughter]

**Interviewer:** That’s true.

**Interviewee:** That’s always a little bit frustrating it turns out.

[laughter]

**Interviewer:** We all are. Don’t worry. [laughs] That’s a good point though, for sure. For this last section of questions, I want to learn a little bit about response accuracy. Besides not answering, we’re curious if there are other ways that you dealt with some challenges. Sorry, she will not stop barking. Hopefully, you can’t hear.

**Interviewee:** That’s fine.

**Interviewer:** I know you mentioned sometimes just default you would answer sort of. Asking questions similar to that. How did you typically handle distractions when taking a survey?

**Interviewee:** With the watch, it’s pretty straightforward of like, “Oh, maybe I had read it,” and then like, “Oh, wait. It is just redirecting.” Then sometimes it would be doing an undo. Sometimes I learned to press with my nose because like, “Oh, my hand’s busy Sorry.”

**Interviewer:** Doing dishes or something. [laughs]

**Interviewee:** What’s that?

**Interviewer:** Everyone always says when they’re doing dishes, they always do that because your hands are wet.

**Interviewee:** Yes. My partner has mostly taken over the dishes since grad school.

**Interviewer:** Good. That’s so nice.

**Interviewee:** That sort of thing and they’re like-- [laughs] Let’s see. What was the question?

**Interviewer:** How did you typically handle distractions spontaneously?

**Interviewee:** Oh, distractions. Then just on the survey, if I’m at work and somebody comes up generally pausing mid-survey and then hoping I can get to the rest of it. I feel like that’s true in general either pausing, doing whatever, and coming back to it or occasionally having more split attention. I can get carsick and so if I’m reading while I’m riding in a car. Maybe reading one and answering and then looking up and then-- It was never a big problem doing that but maybe something like that. That split attention, yes.

**Interviewer:** Were there any situations in which your responses may have been less accurate thinking about morning versus night or if you were with a certain person or a group of people or different locations?

**Interviewee:** No, I don’t think so. Maybe sometimes some evenings that could be true of just feeling more drained and so everything, feeling a little more dull or numb or just like, I’m just tired. I don’t know. I feel like it’s still accurate. It’s just different.

That’s I think part of it too, sometimes it would be like, “Oh, how sad are you?” I’m like, “Now that you mention it, I guess I’m a little sad.” That was hard. The not at all was intense. It was hard for me to hit not at all sometimes. Sometimes it was like, not at all. Other days it was just like--

**Interviewer:** It seemed too extreme?

**Interviewee:** Yes. Sometimes. That’s another piece of some of the things you’re like, “Oh, do you go ice skating or whatever,” and sometimes it would happen never, and sometimes the bottom one be would be rarely and that was frustrating for me, because like, “Oh, yes.” I don’t sometimes go skiing. I rarely go skiing, but I do it every few years, but never was the only, “Wow.” That didn’t feel accurate because those options changed. It’s like, “Oh, I want the other options.”

I guess there was sometimes with the watch, actually, back to the question about if I ever just looked at it and said, “No.” There were actually a few times where it just didn’t feel answerable given the options. It was like, “This wouldn’t be accurate if I answered it,” and so just--

**Interviewer:** Let it go.

**Interviewee:** Yes. Then similarly, sometimes if I had had it on like, “Do not disturb for a few hours, then turned it back off.” It would sometimes be like, “Oh, were you physically active 115 minutes ago?” I’m like, “No.”

**Interviewer:** What is that? [crosstalk] An hour, two hours?

[laughter]

**Interviewee:** I’m like, “I don’t know.” I would ignore those. Occasionally, I remember one instance where I had parked my car, run into a store, wandered slowly through the store, and then checked out, and then on my way to the car it buzzed and like, “Oh, were you physically active 15 minutes ago?” I was like, “I ran into the store, but was that 15 minutes ago? I have no idea how long I was in the building.” At one point I was like, “Is this actually testing my memory?”

[laughter]

Are they going to judge me at the end of this?

[laughter]

Like, “I don’t think so.”

**Interviewer:** It was actually 16 minutes ago. [laughs]

**Interviewee:** That was another instance where just like, “I have no idea.” Those are the more specific ones, which is like, “I don’t know.” We may have been on a different question then I backtracked you a different way.

**Interviewer:** No. You answered it. No, that was perfect. Thank you. How do you think your motivation or accuracy changed as you were in the study longer? Obviously, you weren’t in grad school at the beginning compared to now. [crosstalk]

**Interviewee:** It was only the first month that I wasn’t in grad school to be fair. Let’s see. My motivation, and what was the other one? [crosstalk]

**Interviewer:** Accuracy, even.

**Interviewee:** Oh, accuracy.

**[pause 00:29:50]**

**Interviewee:** I think my motivation was a little bit more money focused at the beginning. Wanting to get that max amount every month, and I never hit that.

[laughter]

It did actually fade over time, and so there was this point where I was like, “Oh, that’s actually unrealistic for me.” Okay, and so accepting that and then just started doing the best I can. Then it shifted more into just wanting to be as accurate as possible, and get as much as I can done.

**[pause 00:30:33]**

I think there’s a degree to which it’s almost everything hidden in the middle line towards the last fourth maybe of like last three months of just feeling most everything I’m answering is within a certain little range. That’s fine, but just it felt it had everything sort of balanced each other out to just be in this little moderate. I’m always feeling moderate or whatever. It’s not actually an answer, but just noticing that. I don’t think it’s inaccurate though, but--

**Interviewer:** That’s what you’re feeling?

**Interviewee:** That’s just something that seemed true. There was a day where it was actually really nice to be like, “Yes, I am feeling extremely sad today.” I’m just like, “Cool.” [crosstalk]

**Interviewer: [unintelligible 00:31:58]**

**Interviewee:** Now I actually know what that continuum is for myself of like, “Okay.” Because it feels like most of the time it fits in the middle three and there’s not a whole lot of distinction there. I do like seven-point scale is better than a five-point scale for a reason.

[laughter]

I like my accuracy.

**Interviewer:** Yes? That’s good. [laughs]

**Interviewee:** Yes. It’s more complicated.

**Interviewer:** Last question that does tie into these questions, but doesn’t. What did you think about the questions and messages that were not related to measuring either health activities, health behaviors, or **[unintelligible 00:32:37]**?

**Interviewee:** I don’t know. I mentioned one about ice skating. I didn’t actually get them. Those ones were fun. I looked forward to those because it felt like opportunities to share a little bit something else about myself than just the same old, same old, so that was always what I looked forward to those. The ones about tech being harmful or whatever-

[laughter]

**Interviewer:** That one always comes up. That’s so funny.

**Interviewee:** -yes, those ones were tricky. Again, those are hard. [crosstalk]

**Interviewer:** That’s an existential question, like how do I answer that? [crosstalk]

**Interviewee:** You’re right. How do I answer that in two seconds?

[laughter]

I need some time to think about this. Of course, I never thought about it long enough for the next time, but yes, so I did keep it interesting. At month three, the little blurbs at the end of the surveys, it’s like, “That’s the same thing I’ve already answered,” I just stopped reading them. Same thing with-- Sometimes it would be like, they didn’t ask how I felt, but on the watch, it would be like, “Were you sedentary 20 minutes ago?” I’d be like, “Yes.” Then like, “Bravo,” and I was like, “Not sure.”

[laughter]

Sometimes misaligned with like, “I don’t think you want me to be sad right now, or to be sedentary or to be, you know,” so that was interesting.

**Interviewer:** Are you frustrated? Yes. Bravo. [laughs]

**Interviewee:** Right. That was funny, because I-- Those sorts of things were-- I just totally ignored them at some point.

**Interviewer:** Thank you for answering those questions. I know those were a lot and revisiting the past years. Thank you.

**[00:34:47] [END OF AUDIO]**
