# Supplementary material for: Burden and Inattentive Responding in a 12-Month Intensive Longitudinal Study: Interview Study Among Young Adults
Source: JMIR Form Res. 2024 Aug 2;8:e52165. doi: 10.2196/52165 (PMC11329843; doi:10.2196/52165)
Supplement: Multimedia Appendix 1 [file formative_v8i1e52165_app1.zip › Transcripts/erasuresafeguardravishing_audio_4.22.22.m4a.docx]

**Interviewer:** To get started-- [chuckles] First question for you, is there anything about the time study that you're going to miss?

**Interviewee:** I'm not sure I miss it, but I think it was somewhat valuable during the first periods, I used the questions as mental check-ins for myself to think like how am I feeling right now? Am I procrastinating right now? The answer is almost always yes.

**Interviewer:** [laughs] You and I, both.

**Interviewee: [unintelligible 00:00:36]** elp me to course correct or just be more reflective about my mental state throughout the day. I don't think I need to reflect every hour though.

**Interviewer:** A lot of reflecting.

**Interviewee:** I'd be prompted to do it with a buzz. I think I might miss the extra external encouragement to reflect on my mental state. Then also miss a few extra dollars every month that I could just spend on whatever.

**Interviewer:** Definitely. Okay. I want to first learn a little bit about your experience participating in the study in general. Can you tell me a little bit about how you learned about the study in the first place?

**Interviewee:** I don't recall-

**Interviewer:** It's been a year I know.

**Interviewee:** -exactly how I learned about the study. I might have heard about the study through one of those websites where-- clinical trials or research studies.

**Interviewer:** ResearchMatch.

**Interviewee:** ResearchMatch, Yes. That I receive emails from ResearchMatch and I generally like participating in research studies. The projects that I work on for my dissertation have to do with physical activity. I figured it'd be cool to be a participant in one especially as I think through potential projects that I might want to do for my dissertation. I now understand the participant burden of doing ecological time movement data.

**Interviewer:** Yes. You're familiar with that then with that aspect of research. That's very interesting to hear your perspective on that. On the other side of it.

**Interviewee:** I understand now where the missing data is coming from.

**Interviewer:** [laughs] A year's a long time. Did that factor in at all when you chose to participate in the study? Did you think about the year or any of that?

**Interviewee:** I didn't think about the year in terms of how long it would take me to complete the study. I think a limitation in a lot of physical function, physical activity studies are that they use measures of activity that ask participants to recall their activity over various periods of time, be it a week, a month, or the last year. There could be a measurement error in that.

**Interviewer:** Yes, a bias.

**Interviewee:** It's cool to see the implementation of hopefully a more objective measure of activity. I'm imagining that you all will probably publish a paper one day looking at the concordance or discordance between health report recall and what's actually objectively measured. I think when I enrolled in the study too was near peak pandemic and I was at home most of all the time. When I initially enrolled, I didn't realize how much of a burden it could be to have to do this type of intensive data collection. I think if I had been recruited today or if I was looking at the study today, just understanding more about the daily activities that I have to do and like physically go out and meet people. I'm not sure if I would've enrolled or even passed that initial trial period where you had us tested out for a bit.

**Interviewer:** It's very different from when you started to now, just the world is very different. That's interesting to hear that perspective. Can you describe to us what motivated you to continue answering the surveys on your phone and the watch?

**Interviewee:** I think being a researcher myself was a motivation. I think deriving some value from the mental check-ins. My brother is also participating in the survey and so we just like doing it together.

**Interviewer:** Yes. You can commiserate together too.

**Interviewee:** Yes. That was a bit of a motivation. Then, I don't know. It's my current understanding of how little free living through physical activity research there is out there. I want to be part of that wearables change.

**Interviewer:** Well, we'll be sending out a new newsletter again soon. Even though you finish, we'll keep you on it. Hopefully, we'll be sending you guys some of our papers once everyone's done in this study. Keep you up to date with the research that we are doing because I know, from your perspective being a Ph.D. student and interested in this topic, it would be fascinating to learn not only the research but research that you've been a part of. We'll keep you updated on things with it. You also have access to it as a student, so it'd be good to see it. Talking about compensation, you mentioned it was nice to have a couple of extra dollars each month. How important was the compensation for you and was it motivating?

**Interviewee:** It was important. I don't think I would've enrolled in the study had there not been any compensation. However, when I think about the text messages I had received from the research coordinator saying, "Try and push to get at least eight surveys during the first period in order to get the higher level of compensation." I don't think in my mind the extra money that I would've gotten, that to me wasn't worth it enough to put in the extra effort to answer additional birth period surveys particularly if the trade-off in my mind was having more hours where I can concentrate without any disruption versus whatever the extra incentive was. I'm not sure where that tipping point would've been. If the reward was 500 more dollars then okay, maybe, but--

**Interviewer:** [laughs] Be like, sign me up. Okay.

**Interviewee:** Yes. I've learned that I value quiet time. [chuckles]

**Interviewer:** Definitely, it's important for sure. Can you describe to me the process of answering phone surveys on a typical birthday?

**Interviewee:** Sure. I guess it first begins with waking up. Something I think the study has also made me realize is just how disordered my sleep pattern is. I appreciate that we were able to enter our approximate sleep and wake times and that's used to set when is prompted the next day. I think my ability to plan for when I would wake up was just not reflective of reality. My intentions and what actually happened very rarely aligned. I'd say starting the day on a typical **[unintelligible 00:08:23]** is usually a disruption. Either I was sleeping or I had woken up really early in order to do something. In order to have heads downtime, I'd mute my phone so that I didn't have to deal with it.

After that, I think if this was a day when it wasn't a particular disruption and I was at home or relatively alone for most of the day, I'd try to use the hourly alerts as like a Pomodoro method of sorts to help me break up my time.

**Interviewer:** That's interesting.

**Interviewee:** Again, I'm not sure my ability to plan around it, I felt I had to try to plan my life around or my work pattern around the alerts but I wasn't very successful in doing that. Then my next step would be to mute my phone. Answering the questions, the questions were not particularly invasive or offensive or anything like that. There was one question, the one about I felt like I could not resist doing things that were bad for me or something along those lines. I probably for the first month or so, just was skimming the question and didn't quite realize what it was asking. I think my initial answers made me seem like I have very little, self-control **[unintelligible 00:10:01]**

**Interviewer:** That changed.

**Interviewee:** This is not what the question is asking you, so answer it differently. [crosstalk]

**Interviewer:** Go ahead. Sorry. Were you pretty consistent then in answering that one after you realized what the question was?

**Interviewee:** Yes.

**Interviewer:** Okay.

**Interviewee:** What was the next thing I was going to say? They're decent enough questions. Is there anything more specific that you want to know?

**Interviewer:** Did you ever track how many you were doing? Did that ever matter to you about-- do you have a goal number of surveys you were trying to reach each day or was it like what you were saying kind of based on how busy the day it was?

**Interviewee:** Yes, it was more based on how busy the day was. I did see that permanent banner in my notifications that would tell me how many I had for the day. [chuckles] If it was a day where there's no way that I get anywhere close to the eight, I would just not bother. On the flip side, if there was a day when it's really close to having those eight surveys, then I would try to make an effort to, get from seven to eight. Yes.

**Interviewer:** Okay. What would have made participation in this study more fun or rewarding for you as a participant? Besides paying $500 because that'd be pretty motivating. A month [chuckles]

**Interviewee:** More motivating. On the one hand, this would probably change the nature and quality of data that you all collected, but if you're able to share results in real-time, like tracking my mood over the last week or last month. I think that probably could have incentivized me more to fill it out regularly but at the same time, I understand that by seeing trends in my mood, I might take steps to alter my daily habits or just alter how I'm responding. That could introduce bias like information bias into your study. As a participant, I want to see my data. As a researcher, I understand why I can't see my data.

**Interviewer:** Do you want to join the research lab? Fascinating. [laughs]

**Interviewee:** Yes. **[unintelligible 00:12:52]**

**Interviewer:** I love talking to people that are interested in research or work in research. It's just fascinating. As participants, I should say, because you kind of have that background, that understanding of it also. Okay, let me skip ahead to here. I'm going to ask some questions about increased burden with the survey. I know at times in the time study, it was not easy, and of course, we really appreciate you participating, so we want to know a little bit more about the challenges that you faced in the study. What were some situations in which it was particularly challenging to answer the surveys, both watch or phone surveys?

**Interviewee:** Yes. Anytime that I needed to concentrate, which is almost all the time.

**Interviewer:** All the time. Yes.

**Interviewee:** Yes, if I'm doing readings or working on an analysis or having a meeting, teaching a class, or if I'm in class. I think the questions were not difficult questions. The individual survey, it doesn't take particularly long at all, but I recognize that I was doing a lot of task-shifting. Even by shifting off task for a very short period of time, it means coming back on task to whatever I'm doing, really hard. Yes, so I think the times when I was most responsive were times when task-shifting wasn't a problem. Let's say I'm doing chores around the house or just binging on Netflix or something, where there's no primary task for me to focus on.

**Interviewer:** What about the app or the watch was more disruptive? Was it doing the actual survey itself? Was it the notification, either vibration or sound? What was more disruptive?

**Interviewee:** I think it was the-- so the disruption was disruptive. The notification is disruptive, but it has to be in order to get my attention, I suppose. My brother and I were joking that we have PTSD from [mimics notification sound] I would be like, "Oh, gosh, it's coming now."

**Interviewer:** Oh, no, it's the freaking time study. [laughs] It's going to be a while, like a couple of weeks after you're done, where it's going to be like, "Okay, **[unintelligible 00:15:36]** [chuckles]

**Interviewee:** I think the most distracting aspect of it is that whatever task that I was working on was very, very different from what the time survey is asking me about. If, for example, I'm working on a statistical analysis and then one of my classmates shoots me an email, "Elizabeth, I don't understand how to do XYZ and state." It's relatively easy for me to switch to address that interruption because it's in the same topic. This was going from "think about study design" to "tell me about your emotions." That, I think, was the most disruptive part. I'm also not particularly attached to my phone, so all other apps on my phone are muted. I don't get notifications from them.

For a good portion of the day, I have restrictions on the people whose text messages will even ring. I'll get a text message, but I've set my phone to not vibrate to tell me that random Joe Schmo has texted me. Only my family members' calls and texts will immediately come through. Joining the study where I'm constantly being pained, just that too was a tension shift for me, a difference for me, that was a little bit challenging.

**Interviewer:** Yes. Let's see. Can you describe an instance, a specific instance that comes to mind when you prefer dismissing a survey on your phone rather than actually answering it?

**Interviewee:** Yes. Oh, my goodness. Friday--

**Interviewer:** Or a couple of instances. [chuckles]

**Interviewee:** I teach a class. I'm in class, and then I teach a class back to back on Fridays, and to get from point A to point B I'm riding my bike. I would leave from that class, ride my bike, I hear the phone vibrating because it wants to know where I am now, and it's time for a survey. I'm like, "Okay, I don't have time for this while I'm on my bike." Get to class, sweaty, trying to change quickly. I hear it ringing again, like, "All right, I know what you want, but I'm busy. I need to set up my classroom." Then invariably a student has some question for me before class starts so I'm not able to get to my phone in order to respond to it.

My phone is in the bottom of my bag, so I'm not trying to dig it out because there are a million and one things on top of it. When finally class starts, and I was like, "I need to turn this thing to do not disturb because I have something else to do now."

**Interviewer:** Yes, I teach. Yes.

**Interviewee:** Did you? That happened very regularly.

**Interviewer:** Birth surveys are always on a Friday or on a weekend day. That happened a lot, probably.

**Interviewee:** Is that session and then also, I guess also at the weekend, a bit. My weekends are the days where I sleep in and I'm not going to answer that.

**Interviewer:** Okay, so what did you-- I know your brother is obviously in this too. How frequently do you guys interact? That was the question I was going to ask last time.

**Interviewee:** In-person or--

**Interviewer:** Either way. In-person, over the phone, text messages.

**Interviewee:** Pretty regularly. I'd say at least five or six times per week if we're in different cities. He lives in another state from me. Then if we were both home for holidays or something, we spend a lot of time together. [crosstalk]

**Interviewer:** No, no, no, go ahead.

**Interviewee:** I was just going to say there were some times when we got the surveys at the exact same time. Like we'd be walking down the street at the exact same time, in lockstep, and **[unintelligible 00:20:03]** our data's harmonized, right?

**Interviewer:** Right. Does it know that we're together?

**Interviewee:** Yes.

**Interviewer:** What did you typically tell friends and family then, obviously not your brother, when they asked you about the study? I'm sure people heard your watch vibrate or the ding.

**Interviewee:** Yes, so my PI, my supervisor's really interested in it. She isn't the biggest fan of ecological time assessments because of the participant burden. Most of the studies that we run are among older adults and so we debriefed on the technological burden that comes with having to charge and remember the charter. I think with my PI, I've talked about this in a logistics-of-running-a-study-type-of perspective a lot. My brother's also a researcher, but in soil science and so-

**Interviewer:** That's interesting.

**Interviewee:** -the data he collects are very different. Humans are never involved, a sample size of three because it's soil.

**Interviewer:** Yes. Less bias coming from soil. [chuckles]

**Interviewee:** I think the conversations that we had-- we're pretty nerdy as well. We talked a lot about response bias, and how to draw inferences from incomplete and missing data on imputation strategies. For him, it was just like a crash course on human subjects research. One of my friends, when I was around him and it started buzzing off the wazoo, I told him about the study. He was like, "Well, I guess Google and Apple, they're already collecting all that information, so you might as well get paid for it, for science for just helping a company's bottom line." Those are the main reactions that I've got. I think many of my other friends know that I also just like participating in a research study, so they're like, "Ugh, another one."

**Interviewer:** There she goes again. Now another one.

**Interviewee:** There she goes again. Okay.

**Interviewer:** Okay. For this next section we just want to know about response accuracy. Besides if you didn't answer it, but this is when you did answer them. We're curious to know if there were other ways you dealt with some of the challenges or burden when answering the surveys. How did you typically handle distractions when taking a survey?

**Interviewee:** I'm sorry. Distractions, things that would prevent me from taking the survey? Is that what you--

**Interviewer:** Or just if you were taking a survey while talking to someone, would you be fully engaged in a survey? Would you try to do both?

**Interviewee:** Oh, yes. If I was talking to someone, I wouldn't do the survey.

**Interviewer:** Okay.

**Interviewee:** I think I tended to prioritize anything that I was doing above the survey unless it was a task that I really wanted to procrastinate on and then I'm like, "Okay, I have to contribute to science."

**Interviewer:** "I can't do this paper right now. I'm contributing to science."

**Interviewee:** Exactly.

**Interviewer:** Were there any situations where your responses to the surveys may have been less accurate? Like if you were-- I don't know, not thinking as much through your responses. I know you mentioned that first question, but that was a confusing question.

**Interviewee:** Accurate. I think all of my responses about my mood state, I tried to be as truthful as possible. Some of the questions that were asked about my plans to eat healthily and things like that. I don't plan to eat unhealthily or plan to eat healthily either, so I was never sure quite how to answer that. I always just said, "No, I don't have any plans," because truly I've not thought that far ahead. It's whatever's in the fridge is what I'm going to end up eating.

**Interviewer:** Yes.

**Interviewee:** There are also questions on, the sleep adequacy, whether or not you plan to-- No, no. If you had a goal, I believe on how much physical activity you did or if you have a goal on how much sleep you wanted to get. I think you all were missing a response option because there is like, "Yes, I had a goal and I met my goal."

**Interviewer:** I know **[unintelligible 00:25:27]**

**Interviewee:** Yes, I had a goal but I did not meet my goal but there was no option for-- No, I did not have a goal and so how could I meet or not meet it? It was just like--

**Interviewer:** I know what you're talking about.

**Interviewee:** The wording of that question was funny, but I'm not sure if my answer then was inaccurate or just-- I'm not sure if you would--

**Interviewer:** You basically just answered it the closest that you could with the options that were given in that situation.

**Interviewee:** Yes. I'm trying to think of other questions that might be inaccurate. When I was responding to some of the watch questions about physical activity, again, I wasn't sure what you all meant by physical activity. If physical activity is I got up just now and I walked from my chair to the bathroom-- I think in our studies of older adults, we consider that to be physical activity. It's not exercise, it's not strenuous, but it is some level of activity. I think I just always responded somewhat for those because I wasn't sure what you were getting at. There was sometimes when it would ask me like, "Were you physically active 136 minutes ago?" I don't know.

**Interviewer:** [chuckles] An hour or two hours?

**Interviewee:** Yes, and so I'm thinking back **[unintelligible 00:27:12]** If I had maybe just came in an hour ago from getting groceries, then I would say yes and if I didn't recall doing a large activity, I would say no but there, I just think there's a lot of nondifferential misclassification of my time and what I was doing. especially if the time period that y'all were referencing was **[unintelligible 00:27:38]**

**Interviewer:** Okay. I have one last main question here for this part. What did you think about the questions and messages that did not appear to be measuring health and activities on the phone and the watch?

**Interviewee:** The COVID questions?

**Interviewer:** COVID questions could have been. Any questions that you saw that came up that didn't measure health or activities?

**Interviewee:** I'm trying to think of what other question.

**Interviewer:** Any--

**Interviewee:** What question didn't measure health or your activities?

**Interviewer:** Yes, I can tell you like, "Is the sky blue yes or no?"

**Interviewee:** Oh, those. Yes, to test if I'm paying attention and not looking through.

**Interviewer:** Were they memorable at all? I guess not.

**Interviewee:** I think I did take a screenshot I think of one or two of them that amused me. I think the only one that I remember off the top of head was asking us to select which one of these is a verb or something like that, but all of the words can be used as verbs or they could also be used as nouns.

**Interviewer:** Oh, yes.

**Interviewee:** I think I understood the intent of the questions. I selected the one that was most clearly a verb. Flower is a noun, but to flower is also a verb. That was just me being nitpicky though.

**Interviewer:** As a researcher, do you have any suggestions to make them better? Because you have that understanding.

**Interviewee:** Yes. To make those questions better. I wonder if you could implement those are you paying attention questions for maybe the first month only and then see if people are answering them on average correctly. Then if people are, drop them because it does only take an extra second to answer it, it adds to the length of the survey. Overall, I think it's a good idea to keep it. It's a great measure of how well you're measuring what you think you're measuring that the responses or at least not do, we hope to people just clicking through. I'm sure a reviewer will be happy that you have that data quality assurance measure in there. Inspirational messages at the end of every first survey were really cute. I thought they were cute and adorable.

**Interviewer:** Oh, good. I love that.

**Interviewee:** I took pictures **[unintelligible 00:30:55]** lots of people, my brother thought they were annoying **[unintelligible 00:30:59]** too on the nose. It's a mixed bag, I guess.

**Interviewer:** Is he still in it or is he done?

**Interviewee:** He's almost done.

**Interviewer:** What's his name? We'll keep this as confidential.

**Interviewee:** Daniel.

**Interviewer:** Daniel. Okay.

**Interviewee:** Yes.

**Interviewer:** He said it's annoying, that's so funny.

**Interviewee:** He was very irritated.

[laughter]

**Interviewer:** It's so funny.

**Interviewee:** He's going to have a lot of strong opinions for you, I'm sure **[unintelligible 00:31:25]**

**Interviewer:** I'll be ready. I'll be ready. [laughs] Do you have any other-- Anything that you wanted to talk about before we wrap up the sections part that we didn't discuss.

**Interviewee:** In the future section, are you going to ask me anything about the device itself or--

**Interviewer:** You can tell me about the device for sure, the watch or the app.

**Interviewee:** Again, I'm weird as a young person and that I just forget my phone, I forget my chargers everywhere, but if there could have been a device that just had an ever so slightly longer battery life, I think you'd have gotten a bit more complete data from me. The charging is weird, even though it's using the fast charger and plugging it directly into the wall, sometimes the-- What's it called? The contact, it's just a little bit finicky and I'd have it set there charging, but it just wasn't quite in the right position. Then I'd have to keep it off for a bit longer. Yes. I think the app's interface is fine. Let me open it actually. Are you able to push a survey to me right now so I can **[unintelligible 00:32:47]**

**Interviewer:** No, I can't. You could do-- Let me see, hang on. Let me pull up some steps where you can get a survey to you right now though on your watch. I can walk you through them.

**Interviewee:** Let me see if I can--

**Interviewer:** Where is it? Oh, no, that's uploading. There is a point where I can **[unintelligible 00:33:11]** survey. Okay. Go ahead and open the time app on your phone if you **[unintelligible 00:33:19]** it and then tap on the icon five times.

**Interviewee:** Okay. On my phone and tap on the icon five times. **[unintelligible 00:33:30]**

**Interviewer:** Then it should have you enter a password. The password is details, lowercased D.

**Interviewee:** Okay.

**Interviewer:** Then there should be an option to press a test like micro **[unintelligible 00:33:51]** or **[unintelligible 00:33:54]** Your choice.

**Interviewee: [unintelligible 00:33:54]**

**Interviewer:** Yes. Then now it should bring a question on your watch.

**Interviewee:** Yes, physically active 46 minutes ago.

**Interviewer:** The vibration is so loud. [chuckles]

**Interviewee:** Oh, something-- A setting on the watch that initially changed but then changed it back. I like things that have a really, really large font just to prevent eyestrain and squinting, but when I try to set the Fossil watch in the default, base watch settings to increase the font size, all sorts of things went really wonky and the magnifier went really wonky too. I just did a factory reset on that first day though and reinstalled the app.

**Interviewer:** Having that option would be--

**Interviewee:** Yes, having that option for slightly larger font would be nice. Interesting. Yes, those were the only things that I can think of right now. Oh, also, one reason why I'll be glad to be done with this study is having the Bluetooth and location and internet on on my phone all the time. It just drains the battery **[unintelligible 00:35:22]**

**Interviewer:** Drain your battery. Yes.

**Interviewee:** If the app and phone app could be engineered in such a way that my constant location isn't required because there'll be time where I just turned it off because it's like, "All right, I'm two hours away from home, but my battery is at 10% **[unintelligible 00:35:43]** I can't have **[inaudible 00:35:45]**

**Interviewer: [unintelligible 00:35:46]** Yes.

**Interviewee: [unintelligible 00:35:46]** things on. Yes. If there was maybe a way that it could just use less background battery power, that'll be nice.

**Interviewer:** You're going to have good battery life on your phone again and watch. [chuckles]

**Interviewee:** Yes.

**Interviewer:** Thank you. That was amazing, amazing feedback. I really appreciate that.

**[00:36:09] [END OF AUDIO]**
