# Supplementary material for: Burden and Inattentive Responding in a 12-Month Intensive Longitudinal Study: Interview Study Among Young Adults
Source: JMIR Form Res. 2024 Aug 2;8:e52165. doi: 10.2196/52165 (PMC11329843; doi:10.2196/52165)
Supplement: Multimedia Appendix 1 [file formative_v8i1e52165_app1.zip › Transcripts/facelesschimpacclaim_audio_8.2.22.m4a.docx]

**Interviewer:** Record. To start, can you provide me with some of your overall general feedback?

[silence]

Might ask you again, I might.

**Interviewee:** Okay. Now. Got it. I'm sorry.

**Interviewer:** No problem. I've done that so many times, don't worry about it**.**

[laughter]

**Interviewee:** Overall feedback. I don't really have too much feedback on the watch because I turned on the Do Not Disturb pretty early in the study, it was buzzing during class and while talking with friends and it was a bit too much and the instructions didn't say anything about requirements for how many watch responses I needed to do so I turned that off. The phone?

**Interviewer:** Overall impression. [chuckles] Or if anything sticks out to you during the year like, "I want to tell them about this or I like this or I really enjoyed this."

**Interviewee:** I guess for the Sunday daily questionnaire, it would often ask me, if I walked a dog, even though I don't have a dog and that was repetitive. I think it also asked me about dogs on my watch or something before, and I think I reached out once and was like, "This question isn't going to be relevant to me for the rest of the year can you do something about it?" [chuckles]

**Interviewer:** Can I skip it?

**Interviewee:** I guess that's one thing. Another thing is, I feel like when it would ask me, "Oh, are you feeling this, are you feeling that?" Oftentimes, I'm not really actively thinking about how stressed I am, how tense I am, so oftentimes, I would just click the middle option, because I'm not really strongly feeling anything.

**Interviewer:** Either way.

**Interviewee:** I wasn't quite sure what the expectations were there but my responses, I assume we're pretty uniform for a lot of the times. I don't know what my feedback would be there. [silence]

**Interviewer:** With going back to what you just said about not really feeling either way, that is how you were feeling in that moment, though, correct? I know it was uniform throughout the study, but that's how you were feeling. It wasn't like, "I'm super stressed or I'm not super stressed, it's like I'm in the middle." Do you think it was it was accurately depicting how you felt?

**Interviewee:** Yes. I guess, in the future, a neutral option would be nice. I feel like I had one more thing. I'm trying to remember.

**[pause 00:04:04]**

I don't remember. Maybe as we're going along the questions-

**Interviewer:** If it comes up.

**Interviewee:** -if something comes up then, yes, I'll **[unintelligible 00:04:20]**

**Interviewer:** For sure. I'm going to ask more specific questions. If a question is ever unclear, please feel free to ask me for clarification but I'm going to start with as I asked for, general experience, but I want to learn a little bit about how you learned about the study. How did you first hear about the study?

**Interviewee:** ResearchMatch.

**Interviewer:** ResearchMatch? Do you remember what aspects of the study interested you to want to join? I know it's been a year but-- [chuckles]

**Interviewee:** I think I found out about it when I first signed up for it and so to be honest, I was saying yes to all the ones that I was eligible for. [chuckles]

**Interviewer:** [laughs]

**Interviewee:** I guess it was just the excitement of trying something new.

**Interviewer:** A new study. Can you describe to me what motivated you to continue to answer surveys throughout the study?

**Interviewee:** What kept me going at the beginning was definitely the compensation. I think afterwards, it became a habit. Now that I'm not wearing the watch, it just feels really weird to not have anything on my wrist.

**Interviewer:** It's going to be weird for a little while, even just not getting prompts on your phone, like "What the heck? I missing out on something." It was important to have compensation, at least in the study?

**Interviewee:** Yes.

**Interviewer:** Can you describe the typical process of answering surveys on a buzz day?

**Interviewee:** What exactly do you mean by that?

**Interviewer:** Do you have a certain number of surveys that you wanted to answer like a goal, did you keep track?

**Interviewee:** I did try to go for eight, the minimum and then if I wasn't busy, then sometimes I'd go for the extra four questions. I think, in general, I liked getting most of the questions answered earlier in the day so then I had more flexibility at night. [silence] I know that the one or two most recent ones, I think once I reached eight or so, I turned off the notifications so that I could focus on other stuff.

**Interviewer:** Did you track your completion, and did you use the app like the little bar that was--?

**Interviewee:** The bar?

**Interviewer:** I know it's different for smartphones. If you scroll down, it'll show it to you on your phone or on the app, it'll show you how many you've completed.

**Interviewee:** Yes.

**Interviewer:** I know it's different on other Androids. Would anything have made participation in the study more fun or rewarding?

**Interviewee:** More fun or rewarding?

**[pause 00:07:45]**

I guess it might have been interesting to see what trends you were seeing but I don't know if that's something you're allowed to give out, even the statistics. I think just overall statistics would have been interesting, but I can't think of anything major that would make it more fun.

**Interviewer:** We're wrapping up data collection in the next couple of weeks and so once that's all done, we can actually share some stuff with you guys. Hopefully, we'll start looking at trends, looking at all the different data, and potentially sharing something with you guys. I don't know if you remember getting the newsletters in your email, they came out every couple of months, a quarterly newsletter, maybe.

**Interviewee:** I don't remember now, but maybe.

**Interviewer:** [laughs] Hopefully, we'll keep you on the email chain, so if anything comes up, or if we'd make a new one after this, I think we'll send that to you so you can see some of **[unintelligible 00:09:07]** trends. For this next section, though, I want to learn a little bit about increased burden that the study may have caused. Obviously, we know at times, it wasn't easy to be in the study and so I want to learn a little bit about the challenges that you may have faced. What were some situations in which it was particularly challenging to answer some surveys?

**Interviewee:** I think there are a couple or a few times when a buzz day would overlap with some social thing and so I would tell whoever I was with, "Oh, I have this survey thing that comes every hour or so. Every hour, I need to quickly answer some questions on my phone." I did have a friend who was like, " Oh, you're being rude," and blah, blah, blah. That was not pleasant, but I feel like that was more of my friend's problem than just-- let's see, [silence] it was, I guess a little annoying when I would be watching something and then if one of the buzz surveys came up or something then it would close my Amazon Prime or Netflix or whatever app I was using.

**Interviewer:** You're talking about watching something on your phone, right?

**Interviewee:** Right, those were times when I was like, "Another survey." Oh, definitely while cooking. There were a couple of times when I knew that I still needed to do a few more for the minimum eight surveys that I needed to answer. I would answer with half-greasy hands.

**Interviewer:** It's dedication.

[laughter]

**Interviewee:** I guess that's about it. I think the biggest inconvenience was when I was with friends, but most of the time he would be okay.

**Interviewer:** Understood. What did you typically tell friends or family about the study if they asked?

**Interviewee:** I just told them that I wear this watch and they sometimes ask me questions and every couple of weeks or so they'll ask me questions every hour.

**Interviewer:** What most frequently led you to be unable to or just completely miss answering the survey?

**Interviewee:** If I had turned off notifications and forgot to turn them on in the morning, then I would miss a few morning ones until I would see that I had gotten those surveys and remember that I needed to turn back my notification settings on. I think that was the biggest one, to be honest. If I needed to focus and I turn off the notifications. There were times when I set my wake-up time much earlier than I actually woke up, so I would miss the first few because of that. I didn't really consider that really missing the surveys.

**Interviewer:** You're sleeping.

**Interviewee:** Yes. I guess that's about it.

**Interviewer:** Did you ever prefer to just dismiss a survey? Like you saw it on there and you were like, "I can't right now," and dismiss it?

**Interviewee:** Yes. If I was doing something else and if I had already reached the minimum, then I was definitely more likely to ignore it. If I wasn't doing anything and it came up, then I would usually answer it.

**Interviewer:** For this last section here, I want to learn a little bit about response accuracy. Besides obviously not answering, we're curious how you dealt with other challenges or burdens. How did you typically handle distractions with taking a survey?

**Interviewee:** Distractions with taking surveys. I guess if I was really busy but I had to answer a survey, then I would spend less time on each question. I don't know how much that affected my responses because a lot of the times my responses were in the middle. If I felt like I answered a question incorrectly, I did go back and fix it. I did this every time. Even when I was doing it pretty fast, I think I would still go back for those. I guess that's it.

**Interviewer:** Were there ever any situations where your responses may have been less accurate? Thinking about maybe if you were around someone else or a certain group of people, or if it was a time of day, like morning versus night, for instance?

**Interviewee:** My end-of-the-day surveys might have been a little more biased towards what I was feeling at that moment, or towards the end of the day. I do remember recently, there was one time where I was super tense in the morning, but not in the afternoon, and then when it asked me like, "Oh, how are you feeling today?" I would just answer like, "Oh, I wasn't feeling, any negative emotions." Then afterwards, I'd be like, "Oh, wait, I did have a pretty stressful morning." I think my responses did tend to get a little bit grouped together. If I was feeling super stressful, then oftentimes I would say that I was also feeling tense. Those were linked.

**[pause 00:16:50]**

I think one thing is I do have this constant need to stay productive. I feel like even when I wasn't exactly procrastinating, I would never actually click the, "Are you procrastinating?"

**Interviewer:** I'm not.

**Interviewee:** Not at all. I don't think I ever selected not at all because I constantly feel like I should be more productive. That might have been a bit biased. I also did avoid clicking the extremes. Either not at all or very much so because it was rare that I would intensely be feeling something. Oh, when it asked me, "Did you travel away from your house?" I interpret that as did you go anywhere that was outside your usual location? Even if I did leave my apartment and go to school because that's part of my daily life, I selected no for that. I don't know if that was the intended [crosstalk]

**Interviewer:** You're saying even if you left your house to go to school, it was part of your routine, so you said no, you did not travel outside?

**Interviewee:** Yes.

**Interviewer:** Okay. Makes sense.

**Interviewee:** That's all that comes to mind right now.

**Interviewer:** Do you think your motivation or accuracy changed the longer you were in the study?

**Interviewee:** My accuracy? I don't think so, because I did try to stay consistent, so even though in the middle, I was like, "Oh wait, maybe if I did actually leave my apartment," if I should be saying yes to that problem, I thought like, "I wasn't really doing that the first half, so it doesn't quite make sense to change my response to that now." I did try to stay consistent with those, so I don't think so.

**Interviewer:** Did anything make the study easier or harder over time?

**Interviewee:** Having. same questions asked, made it easier because then you knew what to expect.

**Interviewer:** Last main question here before we jump into something else. What did you think about-- this is random, it's not on the topic of accuracy. What did you think of the questions and messages that were not related to measuring either health behaviors, routines, or moods that came up on the phone?

**Interviewee:** Oh, the ones that measured attention, I didn't really think too much of it. I just assumed that that was so that you could tell whether someone was actually reading the questions or not.

**Interviewer:** Were there any memorable ones that stuck out?

**Interviewee:** I remember there was one that asked us to click the four-letter word. I know there were more. I feel like that's the only one that I remember.

**Interviewer:** It's the only one that stuck out.

**Interviewee:** Yes.

**Interviewer:** Are there any additional points that we didn't cover or discuss that you'd like to talk about?

**Interviewee:** I don't think so.

**Interviewer:** Thank you for answering all of those questions.

**[00:22:05] [END OF AUDIO]**
