# Supplementary material for: Burden and Inattentive Responding in a 12-Month Intensive Longitudinal Study: Interview Study Among Young Adults
Source: JMIR Form Res. 2024 Aug 2;8:e52165. doi: 10.2196/52165 (PMC11329843; doi:10.2196/52165)
Supplement: Multimedia Appendix 1 [file formative_v8i1e52165_app1.zip › Transcripts/moisturedecodelyricist_audio_5.3.22.m4a.docx]

**Interviewer:** Okay. To start, do you have any questions for me before we begin?

**Interviewee:** Not really before. There were just a couple of the questions that you asked in the exit-type surveys, which were a little bit unclear. Then there was a question that I would want to give some clarity on. First, there were questions asking about drinking caffeinated drinks. I don't drink caffeinated drinks. It's really difficult to answer those kind of questions.

**Interviewer:** There's no option, you're saying?

**Interviewee:** Yes. I would maybe consume a caffeine from high dosage ibuprofen if I'm getting a migraine. That just helps it get to me faster so I can avoid that. If I consume caffeine, I can't sleep at all because I'm just really, really sensitive to it since I don't really consume it.

**Interviewer:** Okay.

**Interviewee:** Those answers, there is no good way to answer those.

**Interviewer:** Did you just put the most negative option for those?

**Interviewee:** Yes, I chose the lowest one on the left side.

**Interviewer:** Okay. That was on the 12-month survey?

**Interviewee:** Yes, I think so.

**Interviewer:** The long one?

**Interviewee:** Yes.

**Interviewer:** Okay.

**Interviewee:** There was questions about timing, about one. There were a lot of questions about intent. Those are just bizarre questions to me, mainly because I don't plan most of anything that I do. I mean I plan it, but that doesn't mean I'm going to follow through with it. A lot of things like, do you intend on getting seven hours of sleep? For me, I go to sleep maybe 2:00 or 3:00 or 12:00 or 11:00 and I wake up when I wake up. I don't plan anything. The times that I actually wake up and am annoyed from waking up is when somebody calls me in the morning and they wake me up. Or someone plays music in the morning and they wake me up. I never have any intent behind sleep.

I'm self-employed, a founder of a company so I don't have to be awake for a certain time.

**Interviewer:** No like a set schedule.

**Interviewee:** A lot of the intent questions are weird.

**Interviewer:** How did you typically answer them then? When you would come across them, how would you typically answer them?

**Interviewee:** Unlike the daily surveys, I don't know. I must take a billion screenshots because it always throws me off. My phone is low battery. Oh, I should complain about that too. My phone is brand new and I have to charge it every single day, which is-

**Interviewer:** Unusual.

**Interviewee:** -probably not good for my phone. [laughs]

**Interviewer:** It should be a lot better now that the time app is off your phone. It will be drastically better.

**Interviewee:** I took some screenshot. Sometimes it just cooks the phone, sometimes it drains it over time, but 50% battery usage is coming from that application and I'm like, "Oh boy. My phone's life is going to be a little bit less."

**Interviewer:** Wow. Yes, that's a lot.

**Interviewee:** That's a normal over a normal day. There's idle there's internet usage, screen on, but then 50% of it is from the time study.

**Interviewer:** Wow.

**Interviewee:** It acts like malware almost. [laughs] How much energy it's consuming?

**Interviewer:** 50% is a lot.

**Interviewee:** If you want screenshots for that, I have those because they really surprised me when I got them.

**Interviewer:** Is it screenshots of, like, the battery usage? It says 50%. That it'll say idle time.

**Interviewee:** Yes. Is that something that's interesting to you or just to take a note of?

**Interviewer:** Yes, I can bring that up in our next meeting for sure. I know exactly what that screenshot looks like.

**Interviewee:** Okay, great.

**Interviewer:** You're saying, yes, 50%, that's a lot. It shouldn't be that much. Did you reach out to anyone in the lab about the battery drainage on your phone? Was there ever steps that anyone took with you?

**Interviewee:** No, I mostly complained to my sister.

**Interviewer:** She told me about it and they [crosstalk] Okay.

**Interviewee:** I complained to her about a lot of stuff.

**Interviewer:** I do the same to my brother. Okay. Then you said there was another question that-- was it the intent questions that you wanted clarity on?

**Interviewee:** Well, it--

**Interviewer:** Was there's a specific question?

**Interviewee:** Yes. I don't know if they test the AB test well with a lot of people, perhaps it's only me. There was another question about the same thing with eating food, healthy food. Over last summer, I was working with the YMCA in New York, and we have this, like a project-based learning assignment where my students were surveying people, getting the data back, and building a little facts and figures and presentations.

**Interviewer:** That's awesome.

**Interviewee:** So they can understand how do you build a strong presentation, et cetera, et cetera. One of the student groups, they had related to food and people's behaviors and attitudes towards food. I thought it was nice, but when they were building their questions and asking them and going to send them out in the survey, we talked a little bit amongst the group and asked them, okay, how do you know what healthy means to somebody?

Because, sure, you can have a statistic that says 60% of people think X, Y, or Z, but everybody's situation is their actions, especially with regards to food, is predicated tremendously to their socioeconomic status, to their ability to like you can see it in the data maybe. I took a walk down to past the normal grocery store, past the supermarket, down to the Asian store just to get four ingredients, because I could. Some people don't have that upstream, and then I don't have a car. When I do have a car and I go and do some big shopping at Walmart or so, sorry, I'm not in New York now. I'm in Iowa.

**Interviewer:** [laughs] I was like, "Wow, there's Walmart in New York." [crosstalk] know that.

**Interviewee:** Oh, that'd be crazy. Yes, if somebody doesn't have a car, then the shopping they do changes quite a bit. All that to say, I cook almost every single meal I eat. I say almost 99% of the meals I eat, I cook. When you say unhealthy, I don't know what that really means anymore, because right now, I have basmati rice cooking on the stove. What do I consider unhealthy? Maybe a snack to me is a cup of frozen blueberries. I don't know if you'd call that unhealthy, but to me, that's a snack. All the questions, do you intend on eating healthy? No, I eat what I eat. What I eat happens to be ridiculously healthy, according to everybody else's standard.

To me, it's like, do you intend on eating healthy? No, I don't really have what's around. The answer that you ask that you can get back from that, it's weird. Not to attack the studies too much but-

**Interviewer:** No, that's good to hear.

**Interviewee:** -my 12th-grade students noticed that, too.

**Interviewer:** It is very specific for each person. Healthy could mean one thing to one person. Healthy could mean something to someone else. No, it's good to hear you, I don't know what they view, but how you viewed that question. How did you typically answer it then? Like, when it came up, did you skip it? Did you answer it?

**Interviewee:** Okay. Those questions tend to be the end. After a certain point in that line of questions, I get really irritated. Why isn't this over yet?

**Interviewer:** This is on phone surveys?

**Interviewee:** Yes.

**Interviewer:** Okay.

**Interviewee:** There may be sometimes when it's inconsistent, but the answer should ordinarily say that, "I don't intend on eating, but I likely will." It's like that soft likely. I don't like absolutes. I'm a researcher as well. I don't like absolute terms, but it's like saying more often than not, I will most likely do X, Y, or Z. Especially with respective food.

**Interviewer:** Do you work in public health? You have such a good public health view of things. It's awesome.

**Interviewee:** My sister is epidemiologist. You see my stats. I dance, I skate. Other than that, I don't do anything else.

**Interviewer:** No, but to say who has access to healthy foods, it does depend on socioeconomic status. It depends on so many variables.

**Interviewee:** Oh, yes.

**Interviewer:** Thank you for that insight. That is awesome. It's good to hear.

**Interviewee:** Environmental scientist is my first line of education. I'm a UV Madison environmental science major as an undergrad. In my sophomore year, I joined biosystems engineering, so it's agricultural engineering. After that was plant pathology, mycology, fungi interaction with soil and plants. Then soil science, and right at the end, I was looking at it's remote sensing with an application of rice cropping in Vietnam. From the freshman year, the first semester, there's a class called People, Land, and Food. Every environmental science student needs to take this class.

**Interviewer:** That's awesome.

**Interviewee:** It essentially asks us to look at the human problems surrounding people, land, and food. Food security and global food security was a large part of that. Wars are started for food and water.

**Interviewer:** Absolutely.

**Interviewee:** That's just something that every environmental scientist should always think about. Even though if you're a cleanup crew person or you're in ag tech like myself, or your writing policy, you have to consider those factors.

**Interviewer:** That's awesome. I could sit and talk about this forever. I'll move on to the questions though. Is there anything that you will miss about the time study?

**Interviewee:** I don't think I will. There was a question. I take screenshots for things that I have, so there's another question. I feel guilty when I answer questions on my phone. Guilty was the wrong word for me. I don't know how I answered this one but in the situation with other people, I feel like I'm killing the situation by answering the question sometimes. Then there's a particular question that asks, "Who are you with?" and I have to answer, "Alone, alone, alone." That's very annoying to be reminded that you're alone. I've never thought how alone I am until I have to answer these questions.

It's bizarre because my whole time I was in my undergrad, I spent so much time at the lab by myself, MS by myself, live here alone by myself. I never felt lonely until I keep getting asked about it.

**Interviewer:** Ugh. That's terrible.

**Interviewee:** I think that was really bizarre.

**Interviewer:** Yes.

**Interviewee:** I've been away from home for so long and being asked who you're with, it's weird.

**Interviewer:** Yes, definitely. It's a very valid point. We're definitely not missing any of the questions then. For this next section, I want to learn a little bit about your experience in participating in the study in general and your motivation behind it. How did you learn about the study?

**Interviewee:** My sister told me about it.

**Interviewer:** What features about the study interested you to want to participate?

**Interviewee:** The way she described it was that the study would have almost, I got to stir the pot one second.

**Interviewer:** Yes, that's fine. No problem.

**[pause 00:15:36]**

**Interviewee:** She described it as it having two sections. One where there are people that are wanting to be active, physically doing stuff around, and then people that are doing stuff on the regular already. She falls into that former category and I fall into the latter category. I thought it'd be interesting data, although it'd be a little bit confounded because we're siblings. That our behaviors are kind of, also male, female, so maybe there be a different there. Say maybe there seems to be a huge difference there, but I think we're raised in the same house, we're exposed to similar things, similar education levels. I thought that it'd be a useful data for the study. That's pretty much it.

**Interviewer:** Yes.

**Interviewee:** Oh, and there was, I did want to see what does motion data look like. At the same time, I'm also a data **[unintelligible 00:17:20]**. I'm a little bit tired of high-dimensional data, so I don't want to look at it anymore. Data sets that I have are like, one record has 6,000 attributes. I don't need to look at any more data.

**Interviewer:** Yes. [chuckles]

**Interviewee:** I think those are my motivations mostly.

**Interviewer:** Can you describe what motivated you to continue answering surveys in the study, either on the phone or on the watch?

**Interviewee:** I stopped the watch really early on because it was next impossible to work. I'm sure you got my messages somehow through whoever I was texting. I also program and it's almost impossible to think straight if every 10 minutes you're getting annoyed by the watch. I did ask you guys or your side like, "Have you actually tried working with this thing on?" I say actually, oh, not to criticize.

**Interviewer:** Yes, you can. Hey, I told you to be honest, so that's okay.

**Interviewee:** I just thought it was impossible to work with the surveys going on and then on the phone, my friend/boss said the same thing. He says, "I can see patterns in your productivity."

**Interviewer:** Oh, wow.

**Interviewee:** "When you're on the burst thingy." Because he could tell when I was on the burst thing even if it was just normal work. Everything I was doing slowed down tremendously during those times. We put up for it for a while and then we started having-- we attend more conferences, we are talking with potential business partners and we said no, can't have it interrupting anymore. I think if you were going to anywhere important to try to set up your company, if you were setting up for the future, you wouldn't want to have anything interrupting you while you were doing that.

**Interviewer:** Yes.

**Interviewee:** Even sometimes I'd be referencing something. I can use my phone as a second computing device and then the survey pops up and like, I can't deal with this right now. It's just like, I have my laptop that you're seeing me through. I have a powerful computer for computation and I have my phone. Oftentimes, when I'm working, my phone is that secondary device that I can use to do something on the side where I'm not directly interfering with the three-screen setup I have. I'm having to use a different input device or the same input device. Even though I'm not hearing it, the oh, man, the sounds. Those are maybe the ones.

**Interviewer:** Which one? On both.

**Interviewee:** On the wrist, I just take it off and I don't want it. Vibration on the wrist feels like tapping me, hey, hey, listen, pay attention to me. I turn that off and then I realized that even when I was doing a stretching session, if I use my phone to use a timer, it would communicate to the watch and then bug me anyway even if it was muted, even if it was do not disturbed. I was like, okay, now I can't use my phone for a timer because it's going to communicate to the watch. It's like, okay, not fun. I can't use the phone for this, so it takes away some of that.

That's maybe a phone watch thing in general problem but it was the same idea. It felt like I was being bothered during something that should be relaxing. I went off on a tangent. Sorry.

**Interviewer:** No, this is good. Honestly, all of this feedback is amazing. Don't worry if you go off on a tangent. That's okay.

**Interviewee:** I forgot the original question.

**Interviewer:** It was just what motivated you to continue answering surveys but you basically answered that for me.

**Interviewee:** Why didn't I quit the studying?

**Interviewer:** Yes, that's a good question.

**Interviewee:** I value quality data as a data scientist. I know consistent data, and obviously cyclical things like I have to take out or understand a year-long cycle, especially in agronomy, and agricultural-related stuff. Having a complete time-lapse of a person's behavior is very useful. I felt that in order to help you guys or help me help you complete your cycle, I was to eliminate all the most irritating parts.

**Interviewer:** Just continue on.

**Interviewee:** I could try to get you the cleanest data possible. I will say that a lot of the frustration that you may see in cycles have to do with me trying to work with during the day and I just get more and more annoyed that I can't pay attention to things anymore throughout the day. If you normalize that out, that's fine but just know that that's my tolerance to distraction getting worn down.

**Interviewer:** As the study went on.

**Interviewee:** As the study goes on, and also during the day. This is my third Zoom call. You can be mentally exhausted.

**Interviewer:** Oh, for sure.

**Interviewee:** Maybe in the beginning of the day, you'd be like, "Yes, mm, mm," and then towards the end of the day you're like, "Okay, all right. I got it." I know there's a are you frustrated question. I don't get frustrated very much but I do know that trying to think straight and if I can't think straight, I feel like something's wrong. You may see that in my data. I don't know when that really pops up but it's really clear. Everything is okay, but I can't focus there now. It's really hard to. I'm like I'm annoyed because I can't focus. I think annoyed is a better word than frustrating for me. Some of your word choice doesn't match perfectly. I must have taken screenshots of some of those as well when the word [crosstalk].

**Interviewer:** These were all the phone surveys.

**Interviewee:** Oh, yes. The watch stuff, I couldn't deal with that.

**Interviewer:** Let's see, I know you just told me that it changed from the beginning of the study to the end. We'll say at the end of the study, how many phone surveys do you think you answered on a typical day? Or did it just vary?

**Interviewee:** Two or three. I have to change the sound settings. It's muted, no vibration. If it catches me when I'm there, and I'm not busy, then I'll answer it. Essentially, where you see the data become skinny, especially for those births periods, I think you can understand that I'm actually working. I say actually because before when I'm answering all the time, compare that to now, that's the difference between I am working, versus I have a free moment. I'm 28 years old. I can't imagine anybody that has a normal job doing this. I really have a hard time imagining they could get away with that.

**Interviewer:** Did your job change from then versus now as far because of the pandemic? Was there any aspect of your job that changed?

**Interviewee:** I won't say aspects of it, per se. I did work for YMCA over the summer doing a project-based learning program. At the same time, I am a researcher, so I can do that work from almost anywhere. After that was over which was an in-person type deal, I moved out here. I did you guys record and [crosstalk]. I did take a two-month or three-month break but then I was just working remotely. Technically, we don't have a office right now. I'm just at my house. I won't say that it necessarily had that huge impact because of the pandemic or anything. I'm going to charge my phone because I can finally get a decent battery back.

**Interviewer:** It's going to be really nice **[unintelligible 00:27:54]** that. You've answered a lot of these for me, so I'm going to skip ahead. I also want to be mindful of your time as well. What did you tell your friends or family when they asked you about the study, for instance, your boss that you were telling me about? What did you tell them about the study when they asked you about it?

**Interviewee:** I told them that it's like what we're doing for understanding farmers' and growers' attitudes towards soil and resources over time but it is more intrusive.

**Interviewer:** This study is.

**Interviewee:** We're very observational. We do have a time when we go and have a conversation with them, two-hour, three-hour conversation. Anything that they want to give or anything they want to share, it's on them. That's how we like doing our science. The way that you guys have their study set up is that we're engaging with it almost all the time. I think that's the cleanest difference, I'd say. We're a researcher. Almost everybody I know is a scientist so they understand.

**Interviewer:** Being part of study. Were there any situations in which your responses to the surveys may have been less accurate?

**Interviewee:** Oh, for sure. The go-to-sleep times, extremely inaccurate. It keeps asking me, when do I intend on going to sleep. I noticed that if I push it to make it a realistic time for me to go to sleep then it annoys me through the entire night until I go to sleep. That's not helpful because the reason why I stay up late is because nobody's bothering me at night. I don't need to be bothered throughout the night. Suppose I'm slightly tired, if I start going to sleep, and then I get annoyed to wake up again to answer a survey. That's part of why I don't like all of that. I try to push it earlier in the day so I can actually go to sleep naturally.

What else? When I'm in New York and skating, man, so sometimes I think I skate from it's from 7:00 to 12:00. I don't want to be bothered. I don't know how to answer some of the questions, I'm furious that you're listening to, I don't know, man, you can be listening to an amazing song. Then you get interrupted to look at a blue screen or your music stops or something like that and you're just like, why? You say, I see you, sorry.

**Interviewer:** It's okay.

**Interviewee:** You know right about how you're feeling just the moment before. Even saying that is a very, there's a word for it or maybe there's an idea for it. I think you might know the feeling.

**Interviewer:** Yes, I do.

**Interviewee:** You're enjoying yourself and then someone says, "Hey, come over here," and they're like, "Oh, but don't worry, you can go right back to it." I'm like, "Excuse me."

**Interviewer:** Take you out of that moment. Yes.

**Interviewee:** Even if I know I should be responding to how you're feeling just before you messed up my night. I'm not saying you but you messed up my night. I mean, I think you can see that in the data as well. Yes, that's there. Okay, sleeping wake-up times. Wake-up times, I never know, sleep I never know. I keep thinking to myself, why don't you just pull that off of the watch? I kept saying, you have so many instances of me going to sleep. Why not do simple machine learning on that? Build a small model off of times, when you're pretty confident and then have it learn for the rest of the nights. Hey, if I were in your position, that's what I would do.

I understand training is important but if you have 100 people, and people are in the study for maybe 100 days, minimum, I'm not sure what you have your usage statistics. Instead of being annoying to ask every single night, to train a model to do that, after the fact. Or at least refresh it 1/3 training or 1/5 training, and 5/4, and then check it with the independent validation maybe like five months in or six months in? That's what I would do. I'm not sure if you have plans to do that. If you're doing a study in the future, people get exhausted over time.

**Interviewer:** Totally. Yes. I mean, this is a long study. A year is a long time. It's a long time. Okay, so there's a last portion of the interview where your assistant may have told you. I'm going to share my screen with you and pull up images of maps of your frequently visited spots. Before we get to that, is there anything else because honestly, this is such good feedback. I love that we've kind of gone on tangents because I think you get good, better information that way. Is there anything else that you wanted to tell me about your experience before we get to that?

**Interviewee:** Yes, so when I go to a new place according to this that you have some kind of algorithm and maybe a simple AI that wants to know, what's the spot that you're at? It keeps asking me. I don't feel inclined to answer after the second time. It still feels like it's naive and I think it needs to be smarter. When I was in New York, I went to my girlfriend's house, and it keeps saying, "Oh, where are you now?" I'm like, "Shut up." I don't want that. I know that I'm here. I'm here. We're just going to go out. Don't even ask. I answered this already.

**Interviewer:** Yes, you answered it already.

**Interviewee:** It keeps asking. Then I said, "Okay, fine." I'm going to disable that setting to ask me because I think that literally, you can go and just flag that location. As a data scientist. That's what I do. I felt that a lot of the naive kinds of questions answered after we flagged it once or twice to stop asking. Because you can be excited to go somewhere and then it asks you, and then you're like, okay, don't, you're making my treat a routine. That's what it feels like when I'm asked over and over and over again. Especially going to my girlfriend's house. You know why I'm there. Don't ask me again.

**Interviewer:** Yes. Don't ask me again.

**Interviewee:** Okay, I think that's it.

**Interviewer:** Okay, thank you. Honestly, that's such good, valuable information. I appreciate that. Thank you.

**[00:36:55] [END OF AUDIO]**
