# Supplementary material for: Burden and Inattentive Responding in a 12-Month Intensive Longitudinal Study: Interview Study Among Young Adults
Source: JMIR Form Res. 2024 Aug 2;8:e52165. doi: 10.2196/52165 (PMC11329843; doi:10.2196/52165)
Supplement: Multimedia Appendix 1 [file formative_v8i1e52165_app1.zip › Transcripts/mysidvattedlexica_audio_8.10.22.m4a.docx]

**Interviewer:** Okay, I should have started. If a question isn't clear, you can just ask me any time for any clarification, and we'll also have time at the end for any questions that you might have.

**Interviewee:** Okay.

**Interviewer:** First, I just want to learn a little about your experience in the study in general. How did you first learn about this study?

**Interviewee:** I think it's from a website, Research Gate, is that what it's called? I think so.

**Interviewer:** How did you specifically find our study? Was it like just featured in something or did anyone tell you about it?

**Interviewee:** The website sent me an email and asked me if I'm interested in-- I think if I click on 'I'm interested', and then your team reached out to me.

**Interviewer:** What features of the study interested you in participating, was the watch or anything else like that?

**Interviewee:** Yes, the watch and the tracking activity and things like that.

**Interviewer:** Then, can you also describe what motivated you to continue to answer surveys in the study because we know there were a lot of surveys and it was a year-long study?

**Interviewee:** Yes.

**Interviewer:** Can you describe some of your motivational factors behind continuing to answer the surveys?

**Interviewee:** It became like a habit as I go on doing it, but then I thought that it is really annoying sometimes. [chuckles] I'm doing a certain thing and then the vibration comes-

**Interviewer:** Yes.

**Interviewee:** -especially **[unintelligible 00:01:49]** like a lot.

**Interviewer:** Yes, yes, the **[unintelligible 00:01:53]** days were a lot. For you, how important was the compensation for the **[unintelligible 00:02:00]** to you?

**Interviewee:** I actually forgot about it already. [laughs]

**Interviewer:** Oh.

**Interviewee:** I was not keeping track of it. I have no idea how much balance I have right now or how much I've earned so far.

**Interviewer:** One of the compensation for the study was up to $100 per month.

**Interviewee:** Oh.

**Interviewer:** If you had kept track of that, how motivational or a factor would that happen to you?

**Interviewee:** Oh, that is pretty motivational. Yes.

**Interviewer:** Speaking about **[unintelligible 00:02:46]** like we were talking about before, can you describe the process of answering the phone surveys on a typical **[unintelligible 00:02:52]**?

**Interviewee:** Well, what do you mean by that?

**Interviewer:** Did you have a goal of how many phone surveys that you wanted to answer on a **[unintelligible 00:03:07]** or did you just try to answer them as they came in?

**Interviewee:** I tried to answer them as they came in because they would keep pushing notifications. I'd rather get it done, but sometimes I just, I am really busy and I really don't have time for that.

**Interviewer:** How many phone surveys do you think you answered on a typical **[unintelligible 00:03:29]**?

**Interviewee:** I would try to keep it like A, but I didn't really keep track of it.

**Interviewer:** It's okay. Do you have any suggestions on what we could have made to make participation in the study more fun or rewarding?

**Interviewee:** Maybe like the last survey of that day, like if there can be like a, or even for the other **[unintelligible 00:04:01]** surveys, if there could be a button saying that like 'to push it later', I delay it for like a couple of minutes because sometimes I'm like, "Okay, I got this," but I can answer it like five minutes or something later, but then when I come back later, it disappears, so I can't really answer that.

**Interviewer:** We'll look into that. All right, so thank you for that. Now, now we're going to just talk a bit more about some of the challenges that you might have experienced. Mmh?

**Interviewee:** Oh, okay, keep going.

**Interviewer:** No, do you have something to say before?

**Interviewee:** No, nothing. I'm just trying to let you finish because are you asking if there's any challenge?

**Interviewer:** Yes, if you encountered any of the challenges during the study? Were there any situations in which it was difficult to answer the surveys?

**Interviewee:** Yes, I mentioned sometimes I am like doing stuff with my hands and then I really can't reach out to my phone where I watch to answer the survey.

**Interviewer:** I remember you also said something about the vibrations before. Did any of the frequency of the vibrations bother you?

**Interviewee:** Yes, I do find it very disturbing.

**Interviewer:** All right. Then, can you also describe what you might have said when friends or family asked you about the study because if they saw you answering surveys on your watch or something like that?

**Interviewee:** Yes, I did tell them, just tell them that, "I'm doing this study," and then like, "Let me answer the question real quick".

**Interviewer:** Then, let's see. Was there anything else about the app or some of the procedures that were particularly destructive other than when you were doing something else with your hands or the frequency of the vibrations?

**Interviewee:** Nothing else I can think of now.

**Interviewer:** Then, how did you deal with distractions when taking the survey? Say you were like right in the middle of the survey and something else popped up, did you try to finish the survey first or did you handle the other distraction before returning to the survey?

**Interviewee:** I don't really get distraction during the survey much unless I'm at work, which I will actually ignore the survey.

**Interviewer:** Was there any situations that you can think of in which responses to your surveys may have been less accurate like say if you were around a certain person or if it was a certain type of day or you think you were answering the questions too fast?

**Interviewee:** I would say if I will start with people, then I may just put it in vibration and then, or maybe sometimes I forgot to charge my watch, and then I wouldn't get survey at all.

**Interviewer:** How do you think your motivation or accuracy changed as you were in the study for longer? I remember before you said it got a little easier over time because it got more repetitive you said?

**Interviewee:** Yes. Also, **[unintelligible 00:07:30]** we'll get more boring too. I do expect the survey to come, but I'm just used to it, so I would answer a question and then without thinking.

**Interviewer:** Do you remember seeing any questions or messages that weren't related to measuring health behaviors, routines, or your mood on the phone?

**Interviewee:** Yes, I do remember seeing them, things like what is one plus one and stuff like that.

**Interviewer:** What do you think about them?

**Interviewee:** I know why they're there. [laughs] They're trying to keep track like if the person's actually looking at the watch, I guess. Yes, I have no opinion on them, they're just silly questions, you see.

**Interviewer:** Were there any that you think were most memorable though?

**Interviewee:** What do you mean?

**Interviewer:** Like some of the questions or some of the messages were about fun facts or things like that. Do any of them stick out to you or that you can remember?

**Interviewee:** I do remember some of them, but it's not like it sticks to me a lot or something. It just, "Okay, here you go again". Like, "Okay, there's one plus one," and then, "Okay, there's the sky is blue," and things like that, so.

**Interviewer:** Do you have any suggestions on what we can maybe do to make them better?

**Interviewee:** No, I think they're okay.

**Interviewer:** Is there anything else that we didn't cover, things that you would like to discuss right now?

**Interviewee:** What is it?

**Interviewer:** Do you have any questions for us or anything that you would like to discuss with us right now?

**Interviewee:** Oh, I do have a question. I know I'm getting a message about how much is in my balance, can I see how much in total I've earned because sometimes I spend it and then I forget? How much in total did I earn throughout the whole study?

**Interviewer:** We can look into that for you. If anything, they will send you a message later about if we can tell you how much you've earned over the entirety of the study.

**Interviewee:** Yes, because I do get the balance message but I don't remember how much different it was compared to the last month. Then, sometimes I think that maybe you guys are missing data or something because every time someone would message me, every so often they would message me like, "Oh, okay, can you force update?" And blah, blah, blah, and then, I did. Then, I also have my Internet on all day and everything connected, so I don't know why that was an issue, but maybe sometimes they are missing some data from me.

**Interviewer:** Usually when you do perform a forced upload, we will include that data into your compensation but I can look into that for you. Yes, I can look into that for you, and we'll send you a message if we can tell you that information. I'm sorry to hear about the force uploads. It wasn't anything on your part, sometimes the watches or the coding for the data sometimes just switches out, so we need to ask participants to do force uploads because it might not just upload automatically.

**Interviewee:** Yes, I don't think it's uploaded automatically. Also somehow, it was using up all my data at the same time so I don't know what's going on. Like as I check the monthly data usage, the time study app, it will use up 496 Gigabit or something like **[inaudible 00:11:31]**. I don't know why that is because it's not like I've been using it to watch video. I don't know why it's using that much data.

**Interviewer:** Okay, yes. We'll look into that view. I'm sorry to hear about that. We'll send you messages about that as soon as we hear back from the other researchers in the lab.

**Interviewee:** Okay.

**Interviewer:** All right.

**[00:11:55] [END OF AUDIO]**
