# Supplementary material for: Burden and Inattentive Responding in a 12-Month Intensive Longitudinal Study: Interview Study Among Young Adults
Source: JMIR Form Res. 2024 Aug 2;8:e52165. doi: 10.2196/52165 (PMC11329843; doi:10.2196/52165)
Supplement: Multimedia Appendix 1 [file formative_v8i1e52165_app1.zip › Transcripts/thusflattenengraver_audio_6.7.22.m4a.docx]

**Interviewer:** Okay. To start, can you provide me with some of your overall general feedback regarding the study?

**Interviewee:** It was an interesting concept. It was cool to be provided a watch. I didn't have one, and I didn't really have an interest in having one, but it was cool to have that for a little while. I overall think it's interesting idea that y'all are looking at it seems like health metrics and stuff, and that's cool to be a part of. Just like, "Oh, I like contributing to science." The daily-- When are you going to bed? When did you wake up? Those weren't too bad. Slightly annoying. [chuckles] I'll preface this by saying that honestly, I'm not somebody who-- I by default all of my notifications are off. My phone's on vibrate or not even on vibrate like before.

Personally, I don't like looking at my phone a lot, so honestly, I wasn't the best I would say with answering the burst periods. Those were tough to do, I didn't personally like being interrupted. I would do what I could but it just felt annoying [chuckles] the amount of times. In the very beginning, I was pretty good. I tried to answer as much as I could but then I feel like it died off over the years. Sorry, [chuckles].

**Interviewer:** That's okay.

**Interviewee:** The people I interacted with when the watch was broken, it did fall apart at some point and they'll send a new one. That was really quick, really nice when I needed a new clean card after mine got-- My wallet got stolen. That was really nice that came really quick. Everybody in the study I feel was really friendly. It was a bit of a pain just doing the questions a lot being interrupted. I'd dread those days, [chuckles] personally. I thought the puns were-- It was cute the little factoids and stuff.

**Interviewer:** At the end of the questions.

**Interviewee:** Yes, that was a good idea. For $100 a month, I don't know, that's a lot of surveys. That's $3 a day and then for a bunch of interruptions, I did consider asking y'all if I could just drop out, but I was like at least I'll just try my best. I can just answer the ones that I feel like it because at that point too, the part I saw in an email that y'all done gathering new participants. Overall it wasn't bad, but I'm glad it's done [laughs].

**Interviewer:** Yes, for sure.

**Interviewee: [unintelligible 00:03:49]** honest. Yes.

**Interviewer:** That's fair for sure. Okay, I'm going to ask more specific questions and some of the questions will be along the lines of what you were just telling me about. If a question is ever unclear, please feel free to ask me for clarification. We want to learn a little bit about your experience in general in the study and so first question I have for you is, how did you first learn about the study?

**Interviewee:** My sister told me and she I think found out from Reddit or something, but yes, she linked the study to me and she went through the study too.

**Interviewer:** What about the study interested you when you heard about it?

**Interviewee:** I thought it was cool to just contribute to science and be paid just for wearing a watch and answering a few survey questions. Seemed like a pretty easy thing to do for a little extra money. Knowing that you're part of a study. I've done other studies before, I majored in exercise and sports science. It's cool that you are looking at physical activity and mood and different like pulse and stuff.

**Interviewer:** Okay. Can you-- We already answered this earlier, but what motivated you to continue to keep answering surveys **[inaudible 00:05:17]** to just complete the study? What do you think your biggest motivation was?

**Interviewee:** I like finishing things that I start, I felt like I was almost there. It seemed it would be a valuable thing for you all and I just didn't-- It wasn't hurting me overall to use the watch and as long as-- If it got too annoying I could just not answer as many questions, but I felt like there was a minimum that I could at least answer and not be terribly bothersome and it would still be helpful.

**Interviewer:** Definitely. Okay. Can you describe to me the process of answering surveys on a burst day? You said a minimum amount. Did you have a goal that you tried to reach or an average that you tried to have every day for those burst days?

**Interviewee:** In the very beginning I tried to do as much as possible, and it was cool at first seeing, okay, how many could I do? Then after a few months, especially I had some life changes happen too. Before I was in school, just taking classes and I was still in school, but it shifted to doing my clinical rotations. I'm in the clinic all day. Talking to people, it just was not feasible to just look at my phone-

**Interviewer:** Definitely.

**Interviewee:** -when I have to put my attention with patients. I think at that point I'm just going to do as basically as much as I can outside of those hours. Then even on weekends, when I would be away from my partner for the whole week and then only see him on weekends, it just got annoying being interrupted and I was like, I'm just going to answer what I feel like. I'd love to contribute but just my personal piece and my personal presence with that person is a little more important to me.

**Interviewer:** Yes, it's important.

**Interviewee:** I would say I didn't have a real set number goal except as many as possible. Then as few make me not overly annoying. [chuckles] Sorry.

**Interviewer:** No, that's okay. This is all very valuable so don't be sorry at all. Not at all. Let's see, what do I have here? What would've made participation in the study more fun or rewarding or more motivating I guess to stay in the study besides paying more money because that would obviously be more motivating.

**Interviewee:** That's the most obvious one. [chuckles] Maybe if it wasn't so many questions throughout the day, those vast periods. Also the random watch questions. That was like, were you active 13 minutes ago? I don't know.

**Interviewer:** Avoiding those questions then you're saying?

**Interviewee:** Yes. Honestly, I did put it on do not disturb for the watch part because I just-- Oh, maybe the noise, maybe changing the noise, it would just buzz so much. That got on my nerves. Maybe if you looked at different things, maybe if you got to choose your chime, I don't know. That would potentially be less irritating if you had at least some control over it.

**Interviewer:** Yes, definitely.

**Interviewee:** Yes or maybe if the payment system was a little different versus all or nothing. It feels a little demoralizing when you tried your best but you still didn't make the minimum and all the effort goes to nothing versus if you were paid per each. Like one that you tried at least it's something, even if it's just a little bit.

**Interviewer:** Not completed.

**Interviewee:** I feel like that could help future participants not feel as if I don't know. Like their efforts were going wasted or nothing or something like that.

**Interviewer:** For this next section, we want to learn about situations of increased burden that this study caused. We know obviously like you're saying, it wasn't easy and there's a lot of surveys and a lot of time that was put into it. Can you talk about a situation when it was particularly challenging to answer a survey? Does something stick out to you where it was like I-- I know you mentioned when you're with your partner or when you're in a clinic setting, but outside of those two, was there anything that stood out to you?

**Interviewee:** Driving for sure. Sometimes when I'm driving, if I'm going on a trip or something, or since I'm in school and some of these clinicals, they're like an hour away. That's a full hour I can't answer **[inaudible 00:11:19]** It would be particularly annoying when I'd have Google Maps to help me navigate and-

**Interviewer:** Then it comes over.

**Interviewee:** -then it goes right on top and I can't even see like, oh wait, when is my X set? It was annoying that that had to pop up. Then you can't exactly just say, "Look I can't." Maybe that'd be another design thing if you really can't for an hour or something. I guess you could turn the notifications off for an hour. You can't just have it go away because then it'll pop back up.

**Interviewer:** Swipe away.

**Interviewee:** Then clinical being with someone that you'd like to be more present with. At some point, we have exams, the end of the semester. I don't think you're allowed to wear this [crosstalk]

**Interviewer:** Oh yes. I know certain ones.

**Interviewee:** Oh yes. You certainly can't be on your phone.

**Interviewer:** Some programs are super intense.

**Interviewee:** When I'm--

**Interviewer:** That's an interesting point. Did you ever have to take off your watch? Did any professors or in certain classes, did they require you to take off smartwatches?

**Interviewee:** You know, I--

**Interviewer:** That's a good point that I haven't thought of before to ask.

**Interviewee:** Because I know that the policy-- I remember them talking specifically about smartwatches that they would ask us to take them off, but this was before the pandemic. Then a lot of online anyways, but then things went back in person and I think, I'm not sure, they always had proctors. I don't remember having to-- I put my phone away of course.

**Interviewer:** Yes, it's like before I was in undergrad before there were smartwatches really. That's interesting. I wonder if that issue comes up often for college students or anyone that's in school.

**Interviewee:** It definitely was mentioned as something that was not allowed anymore.

**Interviewer:** I know for big exams, like NCLEX or something like that, **[unintelligible 00:13:52]** to wear anything in there. Interesting. Good point. That's a good point you brought up. Okay, let's see. You've answered these ones already. What did you typically tell friends or family when they asked you about the study or someone heard the watch vibrating?

**Interviewee:** I would tell them, I signed up for this study, they pay me to just wear this watch and I have to answer questions on it. [chuckles] Usually I tell them that it's somewhat annoying and I'm sorry. [laughs] It goes off and then there were times where I would rebel and be like, "Look, sorry y'all, I'm just going to take this off." I would tell people that it was part of a study and they'd ask me questions about my mood and **[unintelligible 00:14:53]** my heart rate and physical activity and stuff.

**Interviewer:** Okay. A couple more questions here. These are more about response accuracy, so when you answered questions, how you dealt with challenges and burdens while you were answering them, and how accurate maybe certain answers were in different circumstances. How did you typically handle distractions while you were taking a survey?

**Interviewee:** How did I handle distractions while I was taking the survey? I'd get a little reminder on my phone and that was how it handled my distraction. I'd be like, "Oh, oops, I was distracted. Let me go back to the phone." Because usually, I intended to just get it all the way done through and didn't usually have a problem going back to it. Like after several months or so, I guess in the fall, June last year was when I started. Sometime in the fall, I started just feeling more distracted just in general. I don't know. I didn't I guess prioritize it. I wouldn't say I had a strategy to like, I'm going to try to not be distracted while answering these questions.

I usually just try to do it as fast as possible, get it done with so it wouldn't annoy me again.

**Interviewer:** Yes. Sure. Were there situations in which your responses to the surveys may have been less accurate if you were around certain people and you were just like, I got to get this done or I guess depending on the time of day, location?

**Interviewee:** I'd say mine were really accurate. I honestly probably put a little too much thought into it. I tend to be thoughtful and that's probably why I avoided it more because I'm like, "Okay how am I really feeling?" I feel like a lot of my responses felt generally the same except when something did happen that day that was really different. Even though some of them felt contradictory, I'm like, "Oh, I'm a little focused, but I'm also a little fatigued." I would say that I definitely didn't just pick whatever, just to get all the way through. I would say they're as accurate as I could possibly make them.

**Interviewer:** Okay. Let's see. Last question, you answered that one for me. Last question I have for you here. What did you think about the questions and messages that were not related to measuring health and activities on the watch and the phone? Either health behaviors, any routines, or moods? None of those questions.

**Interviewee:** You mean like, which is not a tree or something?

**Interviewer:** Yes.

**Interviewee:** I thought they were semi-amusing. Like, 'Oh are they trying to make sure I'm not just going through the motions?" They were mildly amusing I guess is how I would describe them. Not a big burden to have one more, "Okay, something different."

**Interviewer:** Did any of them stick out that were memorable?

**Interviewee:** Let's see. There was one where I'm like, okay, technically there's more than one right answer. It was like, which of these is not a verb or something or which of these is a verb? I was like, technically you can make the other one a verb. Yes because you can verbify.

**Interviewer:** Yes, I think I know which you're talking about. It has once been brought up before.

**Interviewee:** I just remember what they vaguely were, they were always really easy. Like which of the following is flat or round or not a country or a plant?

**Interviewer:** Cool. Okay. Thank you for answering those. I know those were a lot of questions, so I appreciate you giving me all the feedback on the--

**[00:19:55] [END OF AUDIO]**
