# Supplementary material for: Burden and Inattentive Responding in a 12-Month Intensive Longitudinal Study: Interview Study Among Young Adults
Source: JMIR Form Res. 2024 Aug 2;8:e52165. doi: 10.2196/52165 (PMC11329843; doi:10.2196/52165)
Supplement: Multimedia Appendix 1 [file formative_v8i1e52165_app1.zip › Transcripts/afflictedrevenueepilepsy_audio_6.6.22.m4a.docx]

**Interviewer:** To start, can you provide me with some of your overall general feedback regarding the study?

**Interviewee:** It was interesting. I guess the biggest thing is obviously the first questions are the same for the most part, and then even the Sunday ones, they're all the same. Even today, the ones on the watch are very repetitive. They ask, for the most part, "Are you with your family, you into an exercise? Are you with friends?" There's one more I can't remember. Some days, like seven times a day or even more, it was repetitive and, of course, it was tough to answer some things like when I'm in the shower, in a car, or things like that, which sucks for me because I want to get everything 100%. That was the toughest part.

**Interviewer: [unintelligible 00:00:54]** pretty good. Most of this is funny too.

**Interviewee:** No, there were some days I'm just one-

**Interviewer:** Getting closed.

**Interviewee:** Other than just the tough part, trying to get everything, it was fun. The questions are interesting. I like the whole anything that measures psychology, physical or mental health. I love that. That's why I wanted to participate in the research study.

**Interviewer:** I'm going to ask, obviously more specific questions. If a question is ever unclear, please feel free to ask me for clarification. You can interrupt me at any time. No problem. First, I want to learn a little bit about your motivation and your experience for joining and participating in the study. First question I have is, how did you learn about the study?

**Interviewee:** My sister, she does research for her job and so she does a whole bunch of research studies. She introduced me to a couple of sites, and this was on one of those sites where you apply to see if you're a good fit, you meet their criteria, and so I applied and just got back **[unintelligible 00:02:16]**. Again, I had to do with psychology and then physical and mental health. That's what I enjoy. That's what I am pursuing in academia right now. I am a huge health nut.

**Interviewer:** Are you studying physical activity then?

**Interviewee:** I'm studying psychology.

**Interviewer:** Oh, awesome.

**Interviewee:** Right now it's IO psychology, but psychology in general. I wanted to do exercise psychology or sports psychology, things like that.

**Interviewer:** That's a fascinating area.

**Interviewee:** Absolutely. Anything psychology is fascinating. I'm like, "Yes, whatever."

**Interviewer:** Can you describe to us what motivated you to continue to answer all of the questions and surveys in the study throughout the year?

**Interviewee:** I wanted to get 100% every single day. Anything I do, I want to try to- because you can see your efforts and you can see your success. I wanted to get 100% every single day, and I wanted to, of course, keep the watch. It's a free watch if I do whatever I needed to do. Then contributing, again, to the field. There was a lot of research studies that popped up that I could have done but, again, this one was the most interesting and the most impactful in the area that I would impact. That's what kept me doing it every day.

**Interviewer:** Did you keep track then? You mentioned you could see progress. Were you keeping track then on the app of how many surveys you answered?

**Interviewee:** It was not- I might keep track-- I remember if I did for the birth surveys, for the four-day periods, I knew how many I missed during those four days. I knew that for sure and then with the watch, I knew how many I missed every day. Maybe a few days back, because I tried to hit at least 95% or something like that, for the week, but I didn't write anything down or anything like that.

**Interviewer:** How important was compensation in this study?

**Interviewee:** It was definitely a motivator. I would say half of why I wanted to do it or a third of why I wanted to do it was the watch, to keep that. A third was, again, the field and the impact, and then another third was compensation. It was a little bit more when I started the actual research study but less now that I'm in a new role making more money.

**Interviewer:** [laughs]. We were all in a different place a year ago. It was **[unintelligible 00:05:08]**. Pretty early on in COVID too, so it was **[unintelligible 00:05:11]**.

**Interviewee:** Exactly.

[laughter]

**Interviewee:** Now we're-

**Interviewer:** I get it.

**Interviewee:** We're on the other side, even though COVID is going to happen forever. It was much different a year ago.

**Interviewer:** Can you describe to me the process of answering phone surveys on a typical birthday? Like from morning till evening, how was the process of it?

**Interviewee:** It was fine. Fortunately, the birth surveys tended to fall on the weekend so it was always a Saturday, Sunday in there, which is much easier because I have my phone on, I don't have it on silent because I'm here. Then even Fridays I work remotely, so it would be on. I would hear it whether I'm in bed or on my laptop on Fridays. I would always be on the lookout because I know it's between 45 to hour 15 when it sends it out. It's not every hour, I realize that soon on, very early in the game. Can't finesse it.

I'll get it and then go through it and then after a while, probably after four or five months, I realized it's the same questions. I would know and it'd be easy and, of course, they'll throw in a random question or two, but pretty easy. Then at work on a Monday or a Tuesday or a Thursday, sometimes it'll be on silent so I'd have to be on the lookout a little bit more to fill the vibration of my phone, or whatever, or I'd be in a meeting or on a call where I can't hear it and just be on a lookout. Just try to do it as fast as possible. Even on the road, I try to answer some. I answer most of them on the road. Who cares? [laughs]. I did it.

[laughter]

**Interviewee:** I answer them as quick as I can or get to **[unintelligible 00:07:12]** something on the interstate looking up. It was pretty. I always tried to overshoot if I was going to bed at 11:00, try to say I'm going to bed at 11:30 so I could still get that last one, or if I'm waking up at 5:00 I'd say I'm waking up at 4:30 because it takes an hour.

**Interviewer:** To get the-

**Interviewee:** Things of that nature.

**Interviewer:** Did you have a goal number of surveys that you wanted to answer?

**Interviewee:** On a daily?

**Interviewer:** Just all of them? Yes.

**Interviewee:** For the births, I always wanted at least 15 or 16 which is hard to do, but that's when I realized late in the game, that's why you have to aim earlier than you wake up and later than you plan on going to bed. Then after I realized that, unfortunately, maybe seven months then I was able to hit some of those or more of those. Then for the watch, the same thing. I tried to aim earlier and later sometimes, but I tried to get at least 60 of those a day. Then, again, I wanted to hit 90%, honestly 100%, but if I missed one or two, that's fine. If I missed three or four, then that's a terrible day.

**Interviewer:** [laughs]

**Interviewee:** Terrible day.

**Interviewer:** Trust me, you didn't have any terrible days. [laughs] You didn't have any terrible days.

**Interviewee:** Forgot my watch a couple of times at home. I went out somewhere and I'm just like, "Oh gosh, dang it." Of course, I come back [crosstalk].

**Interviewer:** You feel naked without it.

**Interviewee:** Yes, because I know it's on my freaking coffee table ringing and I'm missing these surveys. A couple of times it was tough or it died and, of course, this is all erased all after.

**Interviewer:** You mentioned the random questions that came up. What did you think of those questions?

**Interviewee:** It's funny I kept thinking, those psychology classes, I forget what they call them. They're check questions or, oh, I forget what they were called. It's funny knowing what they are to make sure you're not getting fatigue. You are doing these surveys, make sure you're not just **[unintelligible 00:09:23]** stringing everything. It was funny to me seeing those but I thought they made sense and I would get them. I can go through everything fast but then seeing it like, "Oh, okay, boom." It was cool. They were fine.

**Interviewer:** Let's see. I think that was- oh, what would have made participation in this study more fun or rewarding? More motivating, I guess, just saying.

**Interviewee:** More fun, more motivating?

**Interviewer:** Besides paying more, that would obviously be.

[laughter]

**Interviewee:** Absolutely. Just the goal of what I wanted to hit, it would have been cool. At least I wasn't aware of anything where you could track the overall year, how you're doing, that would have been cool because I'm all about short-term and long-term goals. That would have been really cool, but I guess to a certain degree it kind of motivated me even more because I couldn't see it. I knew I was aiming for at least 95% for the entire study. I think that would have been cool.

**Interviewer:** That's an interesting number. No one has brought that specific statistic up of wanting to know that statistic that would be interesting to know for sure.

**Interviewee:** There were times when I had to restart my watch or something would happen and the notifications, not the notifications, but on the watch where you can see how many went out, how many missed, I couldn't see that, and that was frustrating. I just need to know how many I've missed, even in days I know I've hit every single one I don't know that, I might have missed one.

**Interviewer:** What if there is just one that snuck in there, that was the point.

**Interviewee:** Yes, and there were times where I wouldn't feel it and I would miss it or I wouldn't feel it, and I would just look at my watch and see that, "Oh my gosh there's a survey." There were times where they double stacked to where you might answer one, but then you miss the second one if you did it automatically. There's some things there, but yes, keeping track would have been phenomenal for a type one person like me.

**Interviewer:** Yes, that's a very interesting point. I like that though. We're wrapping up on data collection soon, I think we should be done at the end of August. I believe that's what we said in our last newsletter to you guys. Hopefully, we can give you guys some sort of visualization of data throughout the study.

**Interviewee:** That'd be cool.

**Interviewer:** There's obviously a lot of data as you know, you gave us a lot of data, but we're hoping to get something to you guys. May be interesting to see it like you said, that percentage or something to know, a year's worth of data.

**Interviewee:** Absolutely. Question, how long have y'all been doing this, and how many people are doing this?

**Interviewer:** We've been doing data collection for, when did we start? 2020, and then we'll be wrapping up at the end of the summer. Almost done, it's been a long couple of years.

**Interviewee:** Is this your project or a team of people?

**Interviewer:** A team. Yes, I just work in the lab, but yes, there's a team. We work with Northeastern, so USC and Northeastern are collaborating on the study. For this next section, it's going to be about situations of increased burden that the study may have caused. Obviously, we know that the time study was not easy at times, and so we want to learn a little bit more about any challenges that you may have experienced. What were some situations in which it was particularly challenging to answer surveys, either phone or watch surveys?

**Interviewee:** I'll start chronologically throughout the day. I usually work out in the mornings, first thing I'll get up, eat, go to the gym and then shower, go to work. That period where if I'm in the car, I can do a survey on the watch pretty easy, like a birth survey that's the **[inaudible 00:13:44]**, and that's tough. I'm on three different interstates to get to where I work, going probably about a hundred hours an hour, again, y'all aren't showing this to law enforcement. I think I'm good there.

**Interviewer:** I'm going to have to write it down. Don't worry.

**Interviewee:** You changed my name so they don't know who I am. The birth survey is difficult to do obviously at that, I probably got the majority of them done, but that was tough. Then after the workout going in the shower, I would try to time it or I would know, this survey came 10 minutes ago, maybe I should wait an extra couple of minutes and then go after it, but then I realized sometimes it might take 20 or 30 minutes for the next one to come up. That was something I--

**Interviewer:** Dedication.

**Interviewee:** Almost half the time I would miss one. At one point it was a guarantee I would miss one while I'm in the shower so that would suck. Then charging it, because when you're charging it, it's a very short charger, so you would have to keep it somewhere, and if you're moving around then it's hard to reach in the five seconds since you have allowed it to answer it.

**Interviewer:** You're talking about the watch questions, right?

**Interviewee:** Yes, the watch. The difficulty there, of course in the shower that's fine. I think it gives you maybe seven minutes or so, total. Usually, I was good with those, and if I'm here it's on loud. Then I guess the biggest challenge with that is if you're actually on the phone talking, you don't get that notification. Probably I would say at least the half that I missed throughout the entire year might have been me. I probably say a third might've been me on the phone. I'm a recruiter, so 70% of my job is on the phone. I missed a lot on calls, unfortunately. That's it, but I think those were the biggest challenges, to be honest.

**Interviewer:** What part of the app or the study procedures were most disruptive? Was it the vibration from either the phone or the watch, was it actually taking time to do it, the amount? What was most disruptive of all of those?

**Interviewee:** The sound of the watch or the vibration of the watch wasn't- I got used to it earlier on, but for my coworkers that don't know what the hell is going on. The fact that it was probably distracting them distracted me a little bit, and then the phone, I don't know, it's loud and it's egregious in a way, I don't know. Some people were like, "What the hell is that?" I'm like, "It's my survey, get over it." It was a distraction, but I would probably say the watch was a little bit more of a distraction. Actually, the time it took to do either wasn't that much, click or even on the phone, it probably took me 19 seconds to go through all of them, I think it's 19 questions.

**Interviewer:** I was going to say that's a very specific number, 19 seconds, that makes sense then.

**Interviewee:** It probably took me sooner, but like, I think it's 19 questions for the surveys, the regular ones, there's not a test question in there. I'd probably say like 15 to 19 seconds.

**Interviewer:** You may not have done this as you were trying to get a 100%, but can you describe an instance where you preferred dismissing a survey on your phone rather than answering it?

**Interviewee:** I may not have purposefully dismissed one, I may have pushed one off then tried to get to it later, like physical therapy, because I'm in physical therapy right now, and so if I'm working with my therapist then I may have tried to push it off and get to it later, which I didn't get to it or exercising. [laughs] I guess we're all adults but like sex.

**Interviewer:** Yes. That's a valid point.

**Interviewee:** With the watch. She got a little irritated after a while. Unfortunately, I had to **[inaudible 00:18:21]**

**Interviewer:** That's a valid point.

**Interviewee:** I'm pretty sure, or work when I first got into my job in May. Literally right as I started, and of course, I think it's like two weeks of the birth surveys. When you start to make sure you can actually handle the watch. I was in training all day. I know I missed a few of those first couple of weeks because my boss is literally staring at me, training.

**Interviewer:** You're trying to be on your best behavior.

**Interviewee:** Yes. That was kind of tough. Other than that, there really weren't too many that I actually purposefully dismissed, because I wanted the 100% **[inaudible 00:19:05]**.

**Interviewer:** What did you tell family or friends when they asked you or coworkers when they asked you about the study? What did you **[inaudible 00:19:14]**

**Interviewee:** I told them about it. It's a cool, research study by USC. Has a lot to do with physical and mental health. There's some psychology involved. I would tell them, about the birth surveys and the kind of questions that they asked. How many per surveys, four days in a row, every couple of weeks, and then, depending on when I get up, maybe like 60 to 65 short surveys. I described them. A lot of them thought it was cool. My sister actually got it, but never actually did it. She was jealous after. I think I told her like maybe halfway through and she was like, "Oh dang it. I should have done it."

**Interviewer:** She could have done it.

**Interviewee:** She could have done it, right?

**Interviewer:** For this last section here we want to learn a little bit about response to accuracy. Besides not answering questions, which didn't happen often, we're curious if there were any other ways that you dealt with some challenges or burdens with regards to accuracy. How did you typically handle distractions while taking a survey?

**Interviewee:** I normally, if I'm doing a survey, because obviously the watch-- I can literally answer that like that. No matter what I'm doing, if I'm looking at it and the survey's on, I can answer that, no problem. If it's a burst, that's tough. If I am having a conversation with the president or my boss who's the recruiting manager or somebody that I'm meeting for the first time, then that's a little tough because even if it's only 19 questions, I may be going through it looking up and down, and sometimes I may just start it and then put it down for a second to try to finish a conversation or get to a point where I can't get back to it.

**Interviewer:** Yes, that's good.

**Interviewee:** Yes, I hope that answered the question. I don't even remember what the question was.

**Interviewer:** Yes, no, that did. Just how did you handle distractions, but yes, that was good. Were there situations in which your responses to any of the surveys or questions may have been less accurate? If you were just answering them, not really thinking about how you answered them?

**Interviewee:** Sex is that way, one of those--

**Interviewer:** You're like, "Shut it up."

**Interviewee:** Trying to-- Oh, man, the double-surveyed ones on the watch. That, I don't know, it didn't happen too often, maybe a few times a month, but that one is hard because you get one survey, a short survey, and then one literally right on top of it. You answer that one, the second one correctly, and then you're just trying to answer the one that comes after it because it'll literally go away in a nanosecond if you don't answer it right after.

**Interviewer:** That's a frustrating.

**Interviewee:** Yes, absolutely.

**Interviewer:** Sure. Oh, go ahead.

**Interviewee:** Oh, and in the shower, because I would keep my watch in the restroom with me trying to get out of the shower and hit one. That probably was the one on the phones in the mornings, that's the one-- I was able to read the question most of the time, but if I couldn't hear the vibration the entire time, I would just get out **[unintelligible 00:22:50]**

**Interviewer:** Answer, yes.

**Interviewee:** Oh, yes, just anything, whether it's yes, no, sort of, I don't know what the hell the question was. That probably happened probably 10 times or so trying to just hit something absolutely.

**Interviewer:** Do you think your responses changed at all in different types of circumstances you were in? Obviously not showering, but if someone else was around, do you think you would answer differently or if you're in a different location, different space?

**Interviewee:** If I'm working versus relaxing? Absolutely. That's something I noticed more so halfway through is the one question that I thought was interesting because it could come up in different scenarios as are you procrastinating, and in the morning if I wake up at five and go work out and blah, blah, blah. I'm not procrastinating, but then later on in the day if I miss something or if I don't have my numbers or if I'm lazy whatever, then it might change, but then sometimes I'm like, "No, I did this in the morning. I'm not procrastinating." Sometimes you'll be like, "Well, I didn't do this, this hour. Maybe I am procrastinating," because it didn't say for the day.

**Interviewer:** You take that moment.

**Interviewee:** Some of those questions didn't ask for the day. It just depends on your mood and your situation and if in the morning if I sleep in, I don't think I'm procrastinating, it's the weekend I'm trying to relax, but then sometimes like, "Oh, you know what? I could have got up and did some things, maybe I am procrastinating." It did depend on the situation, and your mood, and the context.

**Interviewer:** Let's see. Last big question here, how do you think your motivation or accuracy changed as you were in the study longer? Was it easier or harder over time in the study?

**Interviewee:** Good question. I guess both, right? It was easier because, one, for the birth surveys, I knew what questions it was going to ask except for, again, the check questions every now and then. It was easier in that fashion. Then with the watch again, a lot of them are repetitive. Again, easier in that fashion, but a little-- Then you're motivated to just reach the end, the one-year mark, right?

**Interviewer:** Yes, that's **[unintelligible 00:25:26]**

**Interviewee:** I've been in this for six months, nine months knowing hell I'm about to stop now. You're motivated to get through. I don't know if I would say I was any less motivated. I would say probably only more motivated as it went on because I was already in it and I like to finish what I start.

**Interviewer:** Okay. Let me see. I think that was all of the questions I had for you. Thank you. That was great feedback. That was so--

**[00:26:02] [END OF AUDIO]**
