# Supplementary material for: Burden and Inattentive Responding in a 12-Month Intensive Longitudinal Study: Interview Study Among Young Adults
Source: JMIR Form Res. 2024 Aug 2;8:e52165. doi: 10.2196/52165 (PMC11329843; doi:10.2196/52165)
Supplement: Multimedia Appendix 1 [file formative_v8i1e52165_app1.zip › Transcripts/yearlingfiberspotty_audio_6.22.22.m4a.docx]

**Interviewee:** Is it the video too?

**Interviewer:** No. It's just the audio.

**Interviewee:** Oh, okay.

**Interviewer:** If a question isn't clear, you can go ahead and just ask me for any clarification. We also have time at the end for any questions that you might have. As stated before, I will send you updated instructions on how to personalize your watch later. All right. First, I want to just first learn a little bit about your experience participating in the study in general. How did you learn about the study?

**Interviewee:** ResearchMatch, I think.

**Interviewer:** Do you have to look up any criteria on ResearchMatch or were you just browsing the page?

**Interviewee:** I'm subscribed to their emails, newsletters. I probably read one of their emails and signed up, said I was interested, and then signed up.

**Interviewer:** Then what features of the study were interesting to you?

**Interviewee:** Definitely the smartwatch appealed to me. I'm a poor grad student myself and I could benefit from monetary compensation, so that was a huge motivator.

**Interviewer:** Then can you describe what motivated you to continue answering the surveys in the study? We know that there was a lot of surveys, then a lot of burst periods that you had to go through, and it's for 12 months. Can you elaborate on what kept you going through the study?

**Interviewee:** Like I said, the monetary compensation was a big factor. Actually, I think this might be more of an answer to the previous question, how I initially started the study. I also enjoy tracking health or, I don't know, just answering health-related questions, I guess. I think that was what initially appealed to me as well. Why I continued? I know the study's voluntary, but also I still think that I personally hold that responsibility. Also knowing that it will contribute to science. I also hope that it would amount to something, I guess.

**Interviewer:** Thank you so much. On a typical burst day, can you describe the process of answering your phone surveys?

**Interviewee:** I get a notification and then I answer survey questions. I don't know if that answered your question.

**Interviewer:** Yes. On burst days, did you have a goal for the number of surveys you were trying to complete?

**Interviewee:** 11 was ideal, but I know that just going about my day, especially if I have a full day, then it was difficult to reach that number. I tried to meet eight in those days, or if I was really jam-packed and had no room for more, I don't know, tasks, then I think I just got to it whenever I could. I know that sometimes I didn't meet eight.

**Interviewer:** It's fine. You were able to track completion by knowing that there were 11 surveys on a typical burst day and then tracking from there?

**Interviewee:** Sorry, could you please repeat that?

**Interviewer:** I was just asking, how did you track completion on the typical burst days? You knew there were 11 surveys, did you just track them?

**Interviewee:** I think that there were more than 11 that were offered, but it was on my phone notification, the silent notification, how many were prompted, and how many were answered. I guess I kept track of how many I'd done.

**Interviewer:** Then what could have made participation in the study more fun or rewarding other than the monetary compensation? Was it interesting to answer the surveys or anything else like that?

**Interviewee:** It's the same set of questions, so they get repetitive. Maybe this is my phone, I think the vibration pattern, it doesn't last for a second or two, but then I think it goes on pretty long, and that can be distracting and also unpleasant. Even if that pattern was a bit shorter, it could be because I'm sensitive to sounds, but I think that might have been better.

**Interviewer:** We know that the notifications can be a little distracting sometimes. We're going to move on. We know that participating in a time study isn't easy and we really appreciate your participation. We want to learn a little more about some more challenges that you might have experienced. Were there other situations that made it challenging to answer the surveys other than being busy?

**Interviewee:** Just like I answered this in the REDCap surveys too. If I'm physically carrying something, obviously, I cannot reach my phone and answer them. If I'm in the middle of a meeting or a lecture, then I shouldn't use my phone, ideally. Even in social settings, I think it can be a little awkward or can be a little rude to the person that I'm with because if I'm in the middle of a conversation and I'm reaching for my phone and being distracted by it, then I think that's a little insensitive to them.

Sometimes I would answer them and then I would tell them, "I'm actually in a research study and it's giving me survey questions, so I'm answering them." Sometimes I've done that. I think by being busy, I think it referred to me being engaged in work and then that would also break concentration. I think that was another thing.

**Interviewer:** Then can you elaborate a little bit on when you were in social settings, what did you say when friends or family asked you about the study that you were participating in?

**Interviewee:** That I was loaned a watch and that I answered questions on my phone and my watch too, goes for about a year, asks about my health. I think that's what I told them. [chuckles]

**Interviewer:** Then were there any instances in which you preferred dismissing a survey on your phone rather than answering it if you saw there was a survey on your phone and chose to just not answer it other than when you were in social engagements or in a class or was that it?

**Interviewee:** Maybe when I was asleep and then I was probably sleeping till a later time than I said I would on the phone. It was probably buzzing, then I might have just put it off, or if I was in a crowded area and I was just trying to stay focused in the present moment, I would just probably dismiss it. I'm sure there were times when I didn't do it because I didn't feel like it. Actually, I think there were certain barriers like that, I'd mentioned being in a crowded area or being asleep.

**Interviewer:** Then besides not answering, we're also curious on how you dealt with some other challenges or burdens. How did you handle distractions when you were taking a survey and perhaps there was something else distracting you?

**Interviewee:** When I'm taking a survey and I get distracted?

**Interviewer:** Yes. How do you handle that?

**Interviewee:** I'm not sure. I might have just gotten distracted or I might have just tried to finish it quickly and then do whatever distracted me, that required my attention. I don't know.

**Interviewer:** That's fine. Thank you for answering. Also, any situations in which your responses to the surveys might have been less accurate, you answered without thinking about your responses?

**Interviewee:** I think that if that was the case, I think it would've been because I wasn't feeling anything in particular. You're referring to the burst questions, right?

**Interviewer:** This is just the surveys in general.

**Interviewee:** Oh, okay. Well, as for the burst questions, I think they're-- I feel X, Y, and Z. Then sometimes you find I don't really feel anything in particular, then I just press moderately, but then I pay enough attention so that I can pick out one of those fake questions. I don't know what they're called exactly.

**Interviewer:** Oh, the filler questions to make sure that you were paying attention?

**Interviewee:** The filler ones, validated question, I think. The smartwatch questions, I think I answered-- I think I gave reasonable thought into it before answering, although they were quick so I wasn't able to get to all of them. Sometimes I didn't know some of the words.

**Interviewer:** No, that's okay. Do you notice a few responses changed if you were in different situations? Such as like if you were in a place with a lot of friends or depending on your location or a certain time of day you noticed your responses would change.

**Interviewee:** I think I probably reported more positive affect when I was with my friends or on a vacation or something, after the school is over, after I finished exams or something. I know you'd ask about sleep too, and sleep habits were also different when I was in school and also when I was back home.

**Interviewer:** Then how do you think your motivation or accuracy changed as you were in the study for longer periods of time? Did it get easier over time to answer all the questions while you were doing the study, or did it get harder?

**Interviewee:** I think it definitely took less time as time went on because I knew what questions there were, so I knew what I was expecting. I remember initially I read every single-- the jokes at the end that they give you. Those were really cute. Well, then as time went on I was like-- I don't think I read them very much.

**Interviewer:** It's okay. Speaking about the jokes at the end of the surveys and some of the filler questions that we put in there to ensure that participants were paying attention, were there any that stood out to you or were very memorable?

**Interviewee:** The filler ones or the jokes?

**Interviewer:** Oh, okay.

**Interviewee:** I was asking a question. What was memorable?

**Interviewer:** Oh, were there any of the jokes that stood out to you?

**Interviewee:** I think there was one regarding dinosaur and T-Rex or something like that, but I don't remember.

**Interviewer:** Oh, no, it's okay. Do you have any suggestions on how we can make them better?

**Interviewer:** I think the jokes are pretty cute actually. I got to admit, a month or two in, I'd already stopped reading them.

**Interviewer:** Do you have any improvements we can make on them to maybe help you pay more attention to those? Or is it just that the study became a regular thing that you just weren't looking at them anymore?

**Interviewee:** In my mind, it would be my duty to answer the questions. I think that I just decided to save time by not reading the jokes at the end, but I think they're fine. I don't know if other participants say they read all.

**Interviewer:** Oh, no, it's okay. Thank you for answering that. This is going to wrap up the last part of-- this part of the exit interview. Are there any additional points that we didn't cover or things you would like to discuss or any questions that you might have about the time survey?

**Interviewee:** I know the watch also goes off pretty aggressively, [chuckles] likewise with the phone vibration pattern. If that could be changed to a smaller vibration frequency, not the time but the vibration frequency because it goes off five or eight times or something during one frequency. If it could be less, that would be good.

Also when I'm charging it, that is when I'm not wearing it, it still goes off anyway, pretty often every few minutes or so. I think if it could not go off when it's being charged, when someone's not wearing it, I think that would be really helpful. I charge going to the shower and I didn't turn on do not disturb, and my friends and family, whoever who's there, they have to endure that sound too.

**Interviewer:** We'll make note of that. Thank you for telling us that. Any other questions that you might have?

**Interviewer:** A couple of things. This might have been a no, but is there a way to see my data too, my health behavior patterns, or anything?

**Interviewer:** Did you ever see the newsletters that we sent out? Sometimes we sent out-

**Interviewee:** Yes.

**Interviewer:** -newsletters. We compile your data with other participants, but I'm afraid that we won't-- I don't think we can let you see your individual data.

**Interviewee:** No, that's fair. Then I guess the other thing was, how do I keep up with your work and publication work? Even if it's not publication, the newsletter facts are really interesting.

**Interviewer:** We can send you an email detailing that, but you can also keep up with our publications and research on The REACH Lab website. We have a website dedicated to our surveys and just a list of our publications so we can also send that to you.

**Interviewee:** Okay. Sounds good.

**Interviewer:** Let me make a note of that.

**[00:17:38] [END OF AUDIO]**
