# Supplementary material for: Burden and Inattentive Responding in a 12-Month Intensive Longitudinal Study: Interview Study Among Young Adults
Source: JMIR Form Res. 2024 Aug 2;8:e52165. doi: 10.2196/52165 (PMC11329843; doi:10.2196/52165)
Supplement: Multimedia Appendix 1 [file formative_v8i1e52165_app1.zip › Transcripts/remoldexcludingaffair_audio_7.25.22.m4a.docx]

**Interviewer:** To start, can you provide me with some of your overall general feedback regarding the study?

**Interviewee:** Yes. It was something that I definitely took a little bit of time to get used to. Just the idea of having to consistently respond to surveys took a little bit of time to get used to, but ultimately, you get used to it pretty quickly, I guess, and so it becomes second nature. I guess the only overall feedback is that it can be tricky when it comes to setting the times at which you're potentially going to be going to bed or I guess the planning, like being proactive about what time you're going to receive those surveys because sometimes you, or at least from my experience, I don't necessarily stick to that as I think the night before I'm going to be waking up at eight o'clock, but for whatever reason, I don't.

**Interviewer:** Life happens.

**Interviewee:** Life happens. Exactly. That was something that, looking back, was harder than I imagined. I thought I would be having this sticking to some routine that made sense and sometimes things are out of whack. I went on vacation last week and so I was all over the place. Sometimes the watch dies if you're like-- When I'm at the gym sometimes, I like to just put on a little timer to time myself and I guess that uses up more battery life.

Trying to reconcile that sometimes is difficult because then it dies quicker on those days. Just those little things that make a difference. Overall, the study's awesome, and I had a good time. It was, again, very interesting and so I'm looking forward to the data that's collected. I'm excited to see what comes of it. I think it's a fascinating study, and I'm happy that you all are able to do this with as many people as you've been able to do it with.

**Interviewer:** Thank you. We're wrapping up data collection at, what are we? in July? at the end of August. We're hoping to come up with something that we can share with you guys-- It's like a visualization. Obviously, there's a ton of data, so it's coming up with something about that and then sharing, I guess, what we've been working on, but we have to wait for data collection to finish for that.

**Interviewee:** I love the graphics that you all have been sending out in the past. That's a really cool visualization.

**Interviewer:** Awesome. Good. I was going to be one of my questions was did you see any of those newsletters? That's good. I'm glad you like those.

**Interviewee:** I saved them on my phone so just to peek back at them, I guess, at some point. They're cool.

**Interviewer:** Oh, that's awesome. Cool. That's awesome. I'm going to ask more specific questions. If a question's ever unclear, please feel free to ask me for clarification. I'm going to start with your motivation for being in the study, staying in the study. Just a little bit about your experience in participating. First question I have is how did you learn about the study?

**Interviewee:** Through ResearchMatch, if I'm not mistaken. My sister sent me the website almost a year ago, a year and change ago so makes sense. This is one of the first studies that I saw come up in my email and I just decided it sounded cool, so I figured why not? Time, in particular, is something that I've been both interested in and I've always hated time in a way. I think I'm very much a chronically late person, and so it's just intriguing the way that time functions in my life, so I thought, "Why not?" It sounded pretty cool and I liked the premise and it just piqued my interest. I'm a curious person, in general, so I figured I'd go for it.

**Interviewer:** I love it, and the name of the study, the TIME study. It worked out perfect. Did your sister join the study as well? Or was it just you then?

**Interviewee:** No, it was just me. She's the one who put me onto ResearchMatch, the website, but yes, I don't think she's ever really done a study. I don't think she's ever done a study, actually. She just found it.

**Interviewer:** She's like, "You'll like this. This would be interesting."

**Interviewee:** Yes. Exactly. This is kind of your speed. Why not?

**Interviewer:** Can you describe to me what motivated you to continue to answer surveys in the study? Obviously, we know there were a lot.

**Interviewee:** Yes. For sure. I guess the general idea that I felt a responsibility towards the study to answer questions. Obviously, the money incentive is a thing, too. I think that's in the back of my head, though. I didn't say, "Oh, I'm going to not make money if I don't do this." I think for the most part, in reality, it was just a habit. Over time, it became a habit and a sense of responsibility to this study. I just felt like I wanted to, so I did.

**Interviewer:** Can you describe the process of answering surveys? Mainly on a burst day because that's when, obviously, a lot of the surveys came in. Can you tell me about your typical day of answering surveys on a burst day?

**Interviewee:** Yes. Oh man, sometimes they were pretty stressful especially when you have other plans, especially when it's on the weekend, actually. I always just wished that the burst periods would come during the week because I was just a little bit more relaxed, I guess, or not necessarily being pulled in different directions as I usually am on the weekends. Just with events, and plans, and family, and friends, and things like that, so I tried to-- I wanted the burst periods to come as much as possible during the week, which never happened. It was Saturday, Sunday, Friday, and then Thursday, but it usually landed on the weekends.

As far as the general process, I guess, so morning comes and you do the daily survey, the wake-up survey pretty, pretty easy. Usually, towards the beginnings of the day and the end of the day depending. Obviously, there's a lot of variables that exist here. Like you said, life happens, but usually, towards the middle of the day, it's not too bad. I guess it's that in your head, you realize it's going to keep coming every hour, so you just make a mental note and you realize, "Okay, in an hour, I'll be here," and just be mindful of when the survey's coming or general idea. What was your question exactly? Describing the process of the day?

**Interviewer:** Yes. Just what was it like? Did you try to aim for a certain number of surveys? Did you track your completion on the app?

**Interviewee:** Yes. For sure. I'm a particularly competitive person, I think. I'd like to describe myself as, I guess, and so I liked the challenge of trying to get 11 for the burst periods, in particular. I just thought, "You know what? If I'm going to do this, I might as well do it to the fullest." A lot of the time, as you can probably tell with my data, it did not happen, but I really tried.

**Interviewer:** You tried.

**Interviewee:** I tried. Yes, I really tried. It's funny because I guess in reality, it's okay if especially if you were getting 8 as opposed to 11. That's totally fine, but I would kick myself in the foot. I figured, "Dang, I could have gotten 11, but I didn't." I would try to strive for that, but sometimes, obviously out of necessity or circumstance, it just doesn't always happen.

**Interviewer:** Great. Last question for this section. What would've made participation in the study more fun or just rewarding for you as a participant? Obviously, paying more would be nice.

**Interviewee:** For sure.

**Interviewer:** Any other reason?

**Interviewee:** With anything, right?

**Interviewer:** Yes, with anything. Exactly.

**Interviewee:** That's an interesting question. I don't know. Maybe some more interactive GUIs, the interface. Something like that. Maybe making it a little bit more of a game or something. I think it functions well and I appreciate it as it is, but I'm just thinking that could be a little bit more incentivizing, a little bit easier to just to answer the questions and make it a little playful perhaps. I don't know. I guess it's not also the goal of the study, but it might make it just [crosstalk] **[unintelligible 00:08:47]**.

**Interviewer:** More engagement. Yes.

**Interviewee:** Yes. For sure. As it is, it's cool. I loved the little prompts at the end of the-- Sometimes I would read them and they made me chuckle, laugh a little bit, and I appreciate that. That sort of a thing I think is pretty neat. Just engagement in particular. Yes.

**Interviewer:** For this next section, I want to learn about situations of increased burden. Obviously, we know the TIME study was long and could be challenging at times, so we want to learn about those challenges that you may have experienced. What were some situations in which it was particularly challenging to answer the surveys?

**Interviewee:** Driving.

**Interviewer:** Yes. Definitely.

**Interviewee:** I know it wasn't recommended, but I certainly found myself a few times, I'm like, "You know what?-

**Interviewer:** Don't tell me.

**Interviewee:** -I can do this." I'm also, unfortunately, a little bit of a risk taker, but not terribly. I'm never going to do it so that it makes me lose control, but that's, I guess, one of the situations in which it became difficult. Also, just in general, whenever you're trying to relax, for example. I found myself actually a few times, I went to Puerto Rico for a vacation, and so I was at the beach and was like, "Dang, I don't want to take off my watch because I know I'm not supposed to, but I also don't want to have a watch tan."

**Interviewer:** That's gnarly.

**Interviewee:** That sort of a thing. Silly things like that, I guess, and going swimming.

**Interviewer:** It's understandable.

**Interviewee:** Yes. There's, I guess, just a bunch of examples, but I guess those are some of the big ones for me. Actually, recently, I was at the gym and I was doing something that required wrist movement. I was wearing the watch and I realized, not really in the moment, but I think over time, I was doing something so that I guess I had it a little tighter than I should have. It hit the watch, my wrist hit the watch, and I realized there was a little bit of pain, and so I've-- It's nothing crazy, but I have this weird feeling. It's okay.

**Interviewer:** Oh God.

**Interviewee:** I don't want to worry you all or anything, but that's not anything that's-- It's nothing terrible, but I realized it potentially could have been because of the way that my wrist was moving and the watch was sitting on my wrist. It could have caused a little bit of-- I don't know. It got caught in there or something. That was inconvenient. I guess that had nothing to do with the surveys, but--

**Interviewer:** Still. You're wearing the watch. Give the watch a break after today.

**Interviewee:** That's exactly what I'm thinking. I'm going to give it at least a month or so. Give it some time.

**Interviewer:** Give your wrist a break. For sure.

**Interviewee:** Otherwise, it was not bad at all. Nothing painful.

**Interviewer:** What part of the study was most disruptive, or of the app? Was it the vibration from the surveys coming in? Was it actually answering the surveys themselves? What was most disruptive?

**Interviewee:** In particular, the burst periods. The vibration itself, I got used to that. That's not a problem. Actually, I guess one thing in particular at night. Whenever you're trying to go to bed and the survey comes up, it can be very bright, and so if lights are off, that's a little annoying, but that makes sense. I would want to see my survey. That's the thing. It's the consistency, I guess, of it that can be a little bit a nuisance some, but otherwise, the vibrations were fine, the surveys themselves are perfectly fine. Whenever it catches you on an off day, that's one of the biggest thing for me.

**Interviewer:** Understandable. Let's see. What most frequently led you to be unable to or to miss answering surveys? Obviously, driving, but were there any other situations where it's like, "I can't answer them right now."

**Interviewee:** I think sometimes I really wanted to be intentional about if I'm spending time with my mom, for example, I didn't want to be on my phone and so I felt torn. I know that I definitely didn't answer surveys because of that sometimes, but they also, my folks, in particular, they became accustomed to that. It's been a year so they knew I was doing this. It became a thing.

I guess over time, it was totally fine, but I think that's another example. With friends, with family, when you're trying to be intentional about spending time with people and not looking at your phone, it can be challenging. You feel torn. You don't want to look at your phone and take a couple minutes to do a survey when you're trying to talk and just pay attention to the people that you're with, for sure.

**Interviewer:** Would you dismiss surveys then? Would you ever just actually just dismiss a survey then?

**Interviewee:** I think I definitely have, but honestly, especially with my close friends and family, they sort of understood towards the end. I actually have a really stupid story about an incident that happened when I was trying to do a survey. I wasn't necessarily being intentional with my time. I was out with friends and I got a survey on my phone. It was pretty late. It was, I don't know, maybe 12:00 AM or so. I was with three friends. I decided the nearest couch, I sat on it so that I can just answer the survey.

It just so happened that there was a group of guys that were around that couch. Everybody was standing. Nobody was sitting there. They were standing around the couch. When I sat down, I guess one of them got offended because, it didn't appear this way, but I guess they had reserved the couch or got bottle service or something like that. It almost became a problem because I was sitting on their couch, and I was just innocently trying to answer these surveys. This dude walks up and is like, "What are you doing here?"

**Interviewer:** Tried to fight.

**Interviewee:** Yes. It literally almost became a fight because of the survey, and I was just like, "Dude, I promise you, I'm just trying to answer this survey." I just wanted a minute. My friends, I love them, but two of them were really ready to fight, and I was not trying to cause anything. I didn't want any trouble. I thought it was really funny that that happened because I just wanted to answer a survey.

**Interviewer:** That's (1) dedication to the study. (2) That's a first. I have not heard that story before.

**Interviewee:** I'm glad.

[laughter]

**Interviewer:** I'm glad it didn't end up in a fight because that would have been terrible. Oh, gosh.

**Interviewee:** Yes. It was a little bit of almost a shove. One of the guys came up to my friends afterward and he just apologized after like 20 minutes. Nothing crazy.

**Interviewer:** Ah, boys and their bottle service, I guess.

**Interviewee:** Exactly. That toxic masculinity sometimes.

**Interviewer:** Toxic masculinity. Exactly. That's hilarious. Well, I'm glad no one got hurt. That actually leads into you're talking about your friends. My last question for here is what did you typically tell friends and family about the study when you first joined the study?

**Interviewee:** Interesting. I just let them know that I was a part of this year-long study. I told them the premise and what it was supposed to gauge. That was it. Everybody was pretty understanding, and they also asked questions and thought it was interesting. I definitely think it encouraged a few people to do this sort of a thing, to join surveys and studies because of this. This one, in particular, just because it had already, I guess, closed by the time I started or around the time I started and so-- I thought it's funny that that happened. All my friends and family were understanding, I guess. Nothing crazy. It's not like it's invasive, necessarily. It's just consistently time-consuming.

**Interviewer:** Time-consuming, for sure. Yes. Let's see. Last section here is about response accuracy. Besides not answering surveys, I am curious if there are other ways that you dealt with some challenges or burdens while answering surveys. How did you typically handle distractions when taking a survey? Like a group of guys that are going to fight your friends.

**Interviewee:** Yes. For sure. I tried to extricate myself as much as I could from situations that were distracting. That, a lot of the times, means I'm going to go sit down somewhere and just focus on this. Again, a lot of the times, the surveys would come when I'm already in a convenient spot to answer and I would go ahead and do that. That was pretty simple. I can't think of too many other distractions other than the ones that I've already mentioned.

I think it was convenient, too, that I knew over time I could wait a good minute and a half, two minutes even, before it would disappear, which I could just maybe take that time to remove myself if I was doing-- If I were washing the dishes, for example, I knew I could finish and then get to the survey. Over time, you just realized it's okay if you don't answer it right away and then get to it in a minute or two. Over time, distractions didn't really matter, I guess, to me. It became part of the process.

**Interviewer:** Just, like you said, routine. Were there situations in which your responses to the surveys may have been less accurate? Thinking maybe depending on who you're around or different times of the day.

**Interviewee:** I don't think so, honestly. I am pretty anal about accuracy, I guess. I do think, though, that some of the questions-- A lot of the questions around COVID, for example, on Sunday burst period or Sunday, just at the end of the week, I guess, for Sunday late at-night surveys, the ones that ask you the most [crosstalk] **[unintelligible 00:19:49]**.

**Interviewer:** The longer ones. Yes.

**Interviewee:** At the end, it's a lot of COVID questions. I was actually a contact tracer for like two years at the beginning of COVID. It was actually awesome and pretty fascinating. For me, I guess there wasn't really too much nuance there as far as how did COVID affect you? It was just like, a little bit, not at all, extremely. For almost all of them, I actually just put down it hadn't really affected me just because, well, personally, it hadn't. That's, I think, truthful and true outside of work.

As a contact tracer, you deal with it every day. Yes, it'd impacted me, but personally-- Especially now answering questions around-- I'd had COVID twice now. On those weeks, I think I did answer, "Yes, I have viral symptoms. It has impacted me." Thankfully, I'm glad that I didn't have family members pass away, for example. In that sense, it wasn't, I guess impacting--

**Interviewer:** A huge impact.

**Interviewee:** Right. I guess for most of those, I answered "No" even though I was involved on a daily basis with contact tracing or just, in general, COVID-related work. That, to me, was like, "I'm not necessarily sure if this is particularly the right way of going about it," but that's how I saw it. I answered mostly--

**Interviewer:** That's what I was going to ask. You were consistent, I guess, throughout the study.

**Interviewee:** Yes. Exactly. Right. Except for the couple times that I had COVID, pretty much it was a straight "No impact." That's pretty much it.

**Interviewer:** That makes sense. Let's see here. Two more questions for you. How do you think your motivation or accuracy changed as you were in the study longer?

**Interviewee:** I think that piece of habit definitely played a role. I didn't necessarily feel as motivated towards the end, but it was just a part of my daily routine. I guess for me it was just easy because it was second nature, I guess, by that point. Maybe for friends and family, it was a little bit more frustrating just because they're like, "He's going to go do a survey."

**Interviewer:** There he goes again.

**Interviewee:** Yes. I don't think it's anything crazy for them either, but maybe a little nuisance. Motivation definitely waned towards the end. Naturally, that happens. Regardless, I still felt like, "I want to finish this. I want to see it through." Especially when I would get those text messages from you all. I'm like, "Dang, I didn't do well enough this month." Made me more motivated for the month after that. I wonder if the data even reflects that. I wonder.

**Interviewer:** That's interesting. Yes.

**Interviewee:** Yes. Because I'd felt like, okay, if they texted me this month and I didn't do as great with the surveys, I know I was pushing myself a little bit more to just achieve more of the surveys and answer more surveys the month after. I guess that was some way of motivating me. I don't like disappointing people.

**Interviewer:** You're not disappointing us. Don't worry.

**Interviewee:** Thank you. I appreciate that. I guess in the back of my head, that's what it was. That's pretty much it as far as motivation.

**Interviewer:** Let's see. Last question I have here, what did you think-- You mentioned the little notes we put at the end of answering surveys. What did you think of the questions and messages that were not related to either health behaviors, routines, or mood that came up on the phone and the watch?

**Interviewee:** I thought that they were cute. I thought it was nice, honestly. I thought some of them were pretty corny, but I also thought that it was adorable. It made me smile. A lot of the times, it's nice to just have a cute little message. It brightens your day a little bit. I actually enjoyed that. It was even not related to the [crosstalk] **[unintelligible 00:24:28]**.

**Interviewer:** Something different.

**Interviewee:** Yes, something different. Definitely.

**Interviewer:** Well, thank you for answering all those questions. Are there any points that we didn't discuss that you wanted to talk about or go over?

**Interviewee:** Points that we didn't discuss? I feel like there are so many thoughts that I have just in general, but in reality, I think we covered most of them. If anything, I could, I guess just email you some thoughts, but I don't think we didn't cover anything. It's been a year and I feel like there's a lot to unpack, so I might have to--

**Interviewer:** Tell me later.

**Interviewee:** Yes. Write down some things. Nothing crazy. Nothing that we didn't probably talk about. That fight or almost a fight was the biggest problem I had.

**Interviewer:** That is a big one.

**Interviewee:** Yes. I don't think so. I think we covered pretty much everything, and I'm just grateful. It was cool. I'm glad I could be part of it.

**Interviewer:** Awesome. Yes. Thank you.

**[00:25:30] [END OF AUDIO]**
