# Supplementary material for: Burden and Inattentive Responding in a 12-Month Intensive Longitudinal Study: Interview Study Among Young Adults
Source: JMIR Form Res. 2024 Aug 2;8:e52165. doi: 10.2196/52165 (PMC11329843; doi:10.2196/52165)
Supplement: Multimedia Appendix 1 [file formative_v8i1e52165_app1.zip › Transcripts/quizzicalpremiumfrayed_audio_8.4.22.m4a.docx]

**Interviewer:** To start, can you provide me with your overall general feedback regarding the study?

**Interviewee:** It's the first time I've done a study like this. It did get a little bit, I don't know how to say, annoying at first. It was a little bit of time to adjust. I think it was also because I started nursing school during this time, so I was a bit stressed, so I didn't really want to have an added stress of answering surveys all the time.

Other than that, the watch, I've had to replace it, I think two times because it wasn't turning on. That was a little bit of an inconvenience. Yes. overall, I did enjoy answering the questions. We're getting paid for it, so I think it was worth it.

**Interviewer:** You're a dedicated nursing school is not easy. Doing the study with nursing school, that's pretty good.

**Interviewee:** I have to turn it off a couple of times, and then you guys have to remind me. "You have to turn it on."

**Interviewer:** I forgot about it.

**Interviewee:** It's okay.

**Interviewer:** [chuckles] I'm going to ask more specific questions about your experience in the study. If a question is ever unclear, please feel free to ask me for clarification. I'm going to start with, again, the overall experience in the study. First, I want to learn a little bit about how you first found out about the study. I know it's been a year, so you may not remember.

**Interviewee:** Yes, I think it was from the researchmatch.com website. They sent me emails, and then this one showed up, and it seemed interesting, so I said, "Interested," and then you guys contacted me.

**Interviewer:** Do you remember what interested you about the study or what stuck out?

**Interviewee:** The main part, it was a year-long study, so I wanted to see if I could actually follow through with it and watch too. It was just different. It seemed fun you guys sending out watches and then answering surveys and getting compensated for it.

**Interviewer:** Can you describe to me what motivated you to continue to answer surveys in the study?

**Interviewee:** I would say I was interested in the actual study, what you guys would find. I know that if I don't follow through with the whole year, I feel like it's not like a valid data from the start. Then also, I don't know, it just seems interesting. I just decided to follow through with it, stick with it.

**Interviewer:** Yes. You mentioned obviously, it was nice getting paid for the study. Was it important? How important was compensation in the study?

**Interviewee:** Like, asking if I was to pay for this, would I still do it? Like that. I felt like it did motivate me more, but I would have still done the study even if I wasn't compensated for it. The compensation did motivate me to finish the study.

**Interviewer:** Yes, definitely. Yes, of course. Along those lines besides paying more money, because that would be more motivating, is there anything else that would have made the study more fun or rewarding, or motivating?

**Interviewee:** No, I can't really think of anything. No.

**Interviewer:** Can you describe the typical process of answering phone surveys during the first period, like what that day was like for you?

**Interviewee:** On the phone, it would send a survey, I think, every hour, and then it's pretty much the same questions every hour, so I felt like I was pretty consistent answering those if I wasn't busy. It was on usually the weekends to Monday-- I believe, Friday through Monday. I did decide to postpone it a couple of times.

**Interviewer:** Delay it?

**Interviewee:** Yes. I think that was a nice option because I do work on the weekends too, so I probably wouldn't have able to answer as many questions when I was working. I think I was able to answer most of the surveys throughout the day. I think it was pretty manageable.

**Interviewer:** Did you have a goal number that you would try to reach each day, each per se?

**Interviewee:** Not exactly a goal number, just answering the majority of the questions throughout the day.

**Interviewer:** Did you track completion on the app, like how the app will show you how many you've answered?

**Interviewee:** Not for the phone, but for the smartwatch, I did.

**Interviewer:** See how many answered today.

**Interviewee:** Yes.

**Interviewer:** For this next section of questions, I want to learn a little bit about any situations of increased burden that the study may have caused. We know, obviously, it wasn't easy at times to be in the **[unintelligible 00:06:25]** study, and so I want to learn a little bit more about the challenges that you may have faced. What were some situations in which it was particularly challenging to answer some of the surveys?

**Interviewee:** I know I mentioned nursing school, so when I'm in the hospital, I actually didn't realize that I could just do a not to serve mode, and it won't be questions. That's part of the reason I just watch. I commute to school, too. I live in Stockton, and I commute to the Bay Area, to Oakland.

**Interviewer:** Oh, my God.

**Interviewee:** Yes, I was driving a lot of time. [crosstalk] It is, but **[unintelligible 00:07:16]** schools, I just try to get in anywhere I could.

**Interviewer:** Are you at-- Is it University Pacific? Which school? Which nursing--

**Interviewee:** I graduated from **[unintelligible 00:07:28]**, but they didn't have a nursing program at the time that I graduated, and so I just decided to apply to nursing programs like ADMs or an accelerated bachelor program. They do have one in town. I don't know if you've heard the San Joaquin Delta college. It's a pretty popular one, but it's really competitive, so I couldn't get into that. I did end up getting to community colleges, so I'm going to Merritt College right now, and I still got accepted to one in San Francisco, but it wasn't until later. Then I also got accepted into CSU, San Marcos.

**Interviewer:** Oh, yes. That would have been a boat for you.

**Interviewee:** Merritt College was the first one that got back to me, so I went there. Yes. Even though it's a bit of a drive.

**Interviewer:** Like you said, you just go honestly, with nursing school, it's so competitive.

**Interviewee:** It is. I think for that program, it was a lot of system, so I got lucky.

**Interviewer:** Oh, my goodness.

**Interviewee:** Yes. I got it.

**Interviewer:** University of Pacific, did they add a nursing school now?

**Interviewee:** They did, but I think only if you have-- I'm not sure. I didn't really look into it. It's pretty new, but I think it's only for those that already have a bachelor's, so I think it's like a graduate program.

**Interviewer:** That's a drive, that's for sure. It is good for you, though. That's exciting. Congratulations.

**Interviewee:** It is, yes. Thank you.

**Interviewer:** Anyway, I'm sorry, you were talking about, yes, you commute.

**Interviewee:** Yes. It was hard answering questions when I'm driving hours throughout the day, and other than that, working. Then during hospitals, the clinical rotation, during Zoom meetings for class. Also, when I'm napping too, I get disturbed from the watch. I forget to put it on do not disturb mode. I don't really plan my nap, so I just fall asleep. Oh, you just woke me up.

**Interviewer:** Oh, it's so frustrating.

**Interviewee:** It's over now. I don't have to deal with it.

**Interviewer:** You can take naps all you want, long naps. [laughs]

**Interviewee:** Yes.

**Interviewer:** What part of the app was most disruptive or procedures or any part of the study? Was it the vibration? Was it actually taking time to answer the survey? What part was most disruptive to you?

**Interviewee:** I would say probably just the vibration. I would check maybe it's a different notification or something. I would say just maybe the vibration.

**Interviewer:** Definitely. What most frequently led you to be unable to or just to miss answering a survey completely?

**Interviewee:** I don't know. Like I said, I was starting nursing school. I just wanted to focus on that. Minimize my distractions like leaving my phone on silent or the watch or ticking it off to completely focus on my schooling.

**Interviewer:** Did you ever prefer to dismiss a survey if you saw it come up, you just dismiss it away?

**Interviewee:** I did. I know whenever I look over at my phone, it's vibrating or something. I think in the test or something. I can't look at it. I'm supposed to turn off my phone during exams, but I leave it on silent, and sometimes I do look over, and it's the surveys if I'm busy at work too, I do. Right now, I am serving so it does get busy sometimes. I just leave it unattended. I just get the survey.

**Interviewer:** When you're on your phone?

**Interviewee:** Yes.

**Interviewer:** Let's see. What did you typically tell family, friends or classmates, coworkers, if they asked you about the study? I'm sure they heard the vibration.

**Interviewee:** I think I noticed I would do something like tap something on my watch or the vibrations. They're like, oh, whose phone is that? I'm like, oh, I think it's just my survey, and I'll apologize. Maybe we're talking in a conversation and having dinner or something. They do understand that it's not much of a distraction to them. I just explained to them. It's just a survey that I'm doing for a research project.

**Interviewer:** For this last section here, I want to learn a little bit about response accuracy. Besides not answering surveys or missing surveys, I'm curious if there were other ways that you dealt with some maybe challenges or burdens. How did you typically handle distractions when taking a survey?

**Interviewee:** Are you asking when I'm taking a survey in the middle of doing something? I just answer it as quick as I can. Sometimes I do repeat answers, especially if it's **[unintelligible 00:13:54]** I do tend to just answer this thing as I did before. Only if I was in the middle of doing something really busy but other than that, I felt like I was pretty accurate responding to how I felt in that moment when I answered the survey.

**Interviewer:** Were there any specific situations in which your responses may have been less accurate, or maybe if your responses changed morning to evening or around certain situations with certain people?

**Interviewee:** Let's see. I don't know. Maybe when I'm studying or something, I might have felt frustrated. Maybe that influenced the accuracy of my responses. If I was like frustrated, I would just answer, not randomly, but what I would typically respond.

**Interviewer:** Do you think your motivation or accuracy changed the longer you were in the study?

**Interviewee:** I don't think so. I think I answered pretty consistently throughout the study. I would say in the beginning, since it was new, I would read into the questions more carefully and be more careful with my answers so that it's more accurate. I feel like since it was just a beginning, just getting used to it, I think that factored into it. Since the questions are pretty similar or repetitive, I feel like I didn't have to take that much time carefully answering it. I feel like my responses were pretty accurate throughout the study.

**Interviewer:** Did anything make the study easier or harder over time?

**Interviewee:** No. I can't really think of anything. No.

**Interviewer:** Last question, and this is off-topic but on topic, what did you think about the questions and messages that were not related to measuring either health behaviors, routines, or moods that came up on the phone?

**Interviewee:** Just random questions. I think I liked it. It tests us if we're just answering randomly. I could just tap my watch and not look at it. I think that was a good part on you guys. I felt like some of the questions it was random. It broke the repetitiveness of the questions. I think it was a good idea, a fun idea.

**Interviewer:** Were there any memorable ones that stuck out?

**Interviewee:** No. I can't remember any.

**Interviewer:** I know it's like answering questions for years. They're out of my head. **[inaudible 00:17:40]** Are there any additional points that we didn't cover that you would like to discuss that maybe came up?

**Interviewee:** No, I think we covered everything.

**Interviewer:** For this last section, thank you for answering those questions. I know that was a lot. Thank you.

**[00:18:05] [END OF AUDIO]**
