# Supplementary material for: Burden and Inattentive Responding in a 12-Month Intensive Longitudinal Study: Interview Study Among Young Adults
Source: JMIR Form Res. 2024 Aug 2;8:e52165. doi: 10.2196/52165 (PMC11329843; doi:10.2196/52165)
Supplement: Multimedia Appendix 1 [file formative_v8i1e52165_app1.zip › Transcripts/headwearskirmishantidote_audio_6.27.22.m4a.docx]

**Interviewer:** To start, can you provide me with some of your overall general feedback regarding the study?

**Interviewee:** Is there anything specific that you're looking for? Because on occasion, it would be a little bit annoying because it interferes with social life a little bit, and when you're talking with somebody it can be rude to just look at your watch or look at your phone or something. There's a little bit of that, but that's what you knew going in, so I knew this was going to happen. It's just every once in a while you're just like, "This is annoying and inconvenient," but other than that, it felt fine. For the most part, it didn't seem like it was really going out my way too much.

**Interviewer:** I'll ask more specific questions along those lines too, of the annoyances of getting prompted in the study. First, I want to learn a little bit more about your overall experience participating in the study. First, how did you learn about the study?

**Interviewee:** Shoot, how did I learn about the study?

**Interviewer:** I know it's been a long time.

**Interviewee:** It might've been through ResearchMatch, that website, possibly.

**Interviewer:** Do you remember what features of the study interested in you in wanting to participate?

**Interviewee:** I think it was just because of it was more of a health-based study, I like participating in those just because I know those results generally, they tend to improve public health and that's the field I'm in there. Obviously, the compensation was also nice, but I also like the fact that it was a year-long study because my field is epi, so anytime I see some cohort study like this I'm like, "They need people, I'm going to join because I know it's a pain in the ass to get people for those studies."

**Interviewer:** Thank you. We appreciate that, especially someone from a public health field you know the importance of a year-long study. Can you describe to us what motivated you to continue to answer surveys in the study?

**Interviewee:** It was just doing the science, just making sure I was doing it to completion. Again, part of the whole, you don't want to lose people in the middle of a cohort study deal, so I don't want to be like, I'll pull the plug here. It like, I'm in it, so I'm in it to the very end mindset for me.

**Interviewer:** How motivating was the compensation?

**Interviewee:** On a scale from 1 to 10, in the middle, a 4-ish, 5-ish.

**Interviewer:** Can you describe the process of answering phone surveys on a typical **[00:03:10]** birthday? Like how that day was for you?

**Interviewee:** I'm still a student, so I pretty much met at home. I keep my phone on vibrate the whole time, and I usually have it on a charging doc, so when I know it's a burst period, I usually just keep it in my pocket because otherwise, I'm going to miss it. It would just be the survey went off, if I'm not in a meeting or doing something or driving or something, I would answer it. That's pretty much how it was during the burst periods.

**Interviewer:** Did you have a goal number of surveys that you wanted to answer each day?

**Interviewee:** It was more a goal of how many not to miss in a given day. It was I try not to miss two or more, was the goal for each each day, so like missing three would be, oh, I didn't hit that goal sort of deal.

**Interviewer:** Would you track your completion on the app?

**Interviewee:** From time to time. Like on a given day, maybe if I was interested in seeing how many did I miss, but I didn't do it day to day sort of deal.

**Interviewer:** What would have made participation in the study more fun or rewarding for you, besides paying more money because that would be more rewarding, obviously?

**Interviewee:** I think for me, I would like to see maybe not like a monthly update, but get a feel of how, and I know there's some obviously limitations to this, but how is my data helping or is there a way for you guys to share that sort of data with me, so I can make better decisions about what I'm doing? I know I sit too much because I'm a dry lab person, so I just sit in front of my computer all day anyways. This study was like, "Oh, yes, I'm sitting way too much. I need to get up and move every now and then," so did my habits actually end up changing sort of deal?

**Interviewer:** We're finishing data collection in August, so at the end of August we will be done. Obviously, there's a lot of data, we've been collecting a lot of data. We're trying to come up with a way that we can share some of that with you. either like a visualization, a graph or something, but we'll hopefully have more information for you guys once we wrap up with data collection. Because I know a lot are obviously very interested in that because it's a significant amount of data, and it'd be interesting to know on your end. We'll hopefully get something to you then.

For this next section, you talked about how it would be awkward in social situations, answering surveys. I want to learn a little bit more of the increased burden that the time study may have caused because we know obviously it wasn't easy being in a study that long. What were some situations in which it was particularly challenging to answer the surveys, like social settings as you had said?

**Interviewee:** In social settings, these might be very specific to me, but when I'm playing online games particularly like in MMO, which is what I usually play, answering a survey in the middle of that could be challenging depending on what I'm doing at the time. When I'm out exercising, when I'm climbing, having your phone in your pocket limits your movement, so it's a little bit challenging to answer surveys there. When I'm exercising, I'm sure other exercises would be easier, but for climbing, you don't usually have your phone in your pocket.

**Interviewer:** You're talking about rock climbing?

**Interviewee:** Yes, rock climbing. That might limit your movement because you have something in your pocket that you don't want to fall on.

**Interviewer:** Yes, of course.

**Interviewee:** Again, this might be more specific to me, but like tabletop RPGs, I am in a dungeon dragon session, so having to answer a survey there, it's distracting because that social role playing, if I miss something, one, it's rude and two, it's like, okay, now I have to catch up and it might slow down the game, which is inconvenient.

**Interviewer:** That's good to know for sure. What was the most disruptive part of the procedures of the study? Was it the app itself? Was it the sound? Was it completing the survey? What did you find was most disruptive?

**Interviewee:** I think it was just completing the survey was the most disruptive.

**Interviewer:** What most frequently led you, besides being challenging to answer them, but what most frequently led you to be unable to or to miss answering a phone survey?

**Interviewee:** It was either physically the phone wasn't physically in my possession, so again, with the whole like rock climbing and it's not on my person. Driving, that's the other big one. Then if I'm in a social situation and like the social situation is commanding more of my attention, then the survey, it wouldn't be the time to answer the survey, then I would miss it. Those would be the top three.

**Interviewer:** Would you ever dismiss a survey?

**Interviewee:** I don't remember off the top of my head, but I imagined I would have over the course of the year. I probably would've dismissed a handful, maybe.

**Interviewer:** Do you know what types of situations that would've happened?

**Interviewee:** Probably where I was very invested socially, and I don't want to be distracted.

**Interviewer:** When you were in those social situations and you would answer the survey or people probably heard your watch vibrating, what did you typically tell friends or family about the study, if they asked?

**Interviewee:** I don't think nobody asked. Nobody asked, they just assumed that I'm doing something with a research or something because I do participate in research fairly often, so that a lot of my friends and family are used to it. They're like, "Oh, he's probably just participating in some weird data collection or something."

**Interviewer:** They're used to it. They're like, "Oh, he is answering surveys or doing something." For this next section, I want to learn a little bit more about response accuracy when you were answering the surveys. Besides obviously not answering them, other ways that you dealt with challenges or burdens from outside distractions. On that note, how did you typically handle distractions when taking a survey?

**Interviewee:** Distractions in terms of like, they're distracting me from completing the survey?

**Interviewer:** Yes, or maybe if you are in the middle of a game, but you do decide to answer a survey, how did you typically handle that? Were you fully invested in the survey? How was that situation for you?

**Interviewee:** For the most part, it was if I'm doing the survey, I'm going to do it and answer it honestly. At the same time, in a social situation, try to get through it as quickly as I can, while still being as honest and accurate as possible. On occasions I would answer a question, like, wait, I go back and re-answer it, but that's generally how I would go about it.

**Interviewer:** Do you think there were ever situations in which your responses were maybe less accurate? Like you did, I'm not even thinking about this and I just want to get it done?

**Interviewee:** There probably were a handful of occasions where it was like that, but for the most part I tried to be as honest as I could be.

**Interviewer:** Did you find that your responses changed, like if someone else was around or if you were in a certain location or certain times of the day, did you find that your responses would change? Like morning to night even?

**Interviewee:** I think in terms of time, no. It's more like what was happening during the day. If I was in a meeting with my advisor and you're talking about deadlines, I might feel more stressed and if I got a survey immediately after that, then my answers would change. But in terms of just straight morning versus afternoon versus night, not really.

**Interviewer:** How do you think your motivation or accuracy changed as you were in the study longer?

**Interviewee:** Accuracy might've ended up going down a little bit, I think, towards the tail end probably is longer. Motivation, probably stayed roughly about the same for the most part because after a month or two, you're just like, "Okay, I'm in it. This is just going to be routine."

**Interviewer:** Definitely. This is not on that topic, but still on the topic of a study question. What did you think about the questions or messages that were not related to either measuring health behaviors, routines, or mood on the phone?

**Interviewee:** I didn't mind them so much. It was sort of like, "Oh, this is how are they going to make sure I'm paying attention today." That's why you're asking those questions, so I was like, "Oh, this is an odd one," or, "I've seen this one before," sort of deal.

**Interviewer:** Were any of them memorable, that stuck out to you?

**Interviewee:** I don't remember any. None of them really stood out to me. It was like, here, this is the interesting question of the day.

**Interviewer:** I'm going to change up the questions here a little bit. Do anyone else who participated in this study?

**Interviewee:** No.

**Interviewer:** I'll skip some questions. You talked about rock climbing, but in the past month, have you exercised or performed any type of physical activity such as going for a walk?

**Interviewee:** Yes.

**Interviewer:** What types of exercises do you typically do?

**Interviewee:** Typically, just walk the dog on weekdays, that's Monday through Friday. It usually ends up being about a mile-ish in a day, once in the morning and once at night, it's about a mile. Then Sundays, we go out rock climbing for about an hour and a half, two hours at the gym. Saturdays is usually no exercise.

**Interviewer:** Then you said rock climbing in the morning?

**Interviewee:** Early afternoon, like around 1:00 PM to 2:00 Pm is when we usually start.

**Interviewer:** You answered all my questions there. I was going to ask you what times of day and what days, but you got that. Talk about sleep, were there any instances where the phone surveys or the watch surveys disrupted your sleep?

**Interviewee:** I don't think so.

**Interviewer:** Good, I'm glad. That's a good thing. Do you remember seeing any of the newsletters or birthday the mails?

**Interviewee:** Yes, I do remember seeing those.

**Interviewer:** What'd you think of those?

**Interviewee:** I liked the fact that the team was engaged with the participants, sharing what's going on with the study, getting a sense of how our participation is being helpful.

**Interviewer:** Last question I have for you here. Did you have any technical problems with the phone or the watch time app that you solved yourself without our assistance?

**Interviewee:** I'm not sure if it was a time app issue, but sometimes the watch would disconnect from the phone and just resetting, reconnecting it via OS fixit, but nothing specifically with the app.

**Interviewer:** That's good. Do you have any other questions or anything that you wanted to bring up that maybe wasn't brought up?

**Interviewee:** Nothing that I can think of.

**Interviewer:** Okay.

**[00:16:07] [END OF AUDIO]**
