# Supplementary material for: Burden and Inattentive Responding in a 12-Month Intensive Longitudinal Study: Interview Study Among Young Adults
Source: JMIR Form Res. 2024 Aug 2;8:e52165. doi: 10.2196/52165 (PMC11329843; doi:10.2196/52165)
Supplement: Multimedia Appendix 1 [file formative_v8i1e52165_app1.zip › Transcripts/brinkaminounframed_audio_7.5.22.m4a.docx]

**Interviewer:** of course. To start, can you provide me with some of your overall general feedback regarding the study?

**Interviewee:** General feedback? Honestly, it has been pretty good a few times. I did the exit survey as well and I answered that over there. The few times when I was not responsive is mostly around, let's say so I graduated last year. I think soon-ish afterward we started the survey. I started my full-time job in July. Over the past six months and the three months actually, it has been really busy. I think that was the only time when I was honestly sometimes totally did not even realize my watch was buzzing [laughs] with the notification or by the time I look at it, it was too late. Overall, besides those busy times and days when I couldn't answer the survey, overall it has been really good. The burst periods were a little hard sometimes depending if I'm traveling or with family and I'm not paying attention to my phone. Overall, it was pretty good. I think the one thing I noticed about burst surveys, if I delayed it for the two days, then for the next survey I didn't have that much break in between. Then it used to reset if I don't delay it afterward. It'll be every two weeks, I guess. A few feedback on a few of the questions, especially maybe I took it too literally [laughs] It was like I intend to exercise today. Was sometimes a little confusing because I would've worked out in the morning, but this question is asked in the evening. I'm like, "Yes, I do, but I'm not going to work out after this answer [laughs] because I have already worked out."

**Interviewer:** You would say yes then if you did already exercise that day?

**Interviewee:** I did. Otherwise, yes, it was pretty straightforward. I like that on the watch it was very quick to answer. The one thing I noticed was if I accidentally picked an answer and I undo it, it was the only time I can undo and not undo after that if I accidentally do it again, which has happened before [laughs]. Then it's pretty straightforward and easy.

**Interviewer:** Great. For this next section, I'll ask more specific questions.

**Interviewee:** Sure, yes.

**Interviewer:** We want to learn about your experience participating in the study in general. First, I want to learn a little bit more about how you learned about the study. If you could tell me a little bit more about how you learned about the study.

**Interviewee:** I actually did another study in USC and I, oh, my God, it's so bad on me. I don't remember what the study was about, but I did participate in it. It was something similar. It was answering a lot of these surveys. It was through a phone and not through a watch app. The first study I found was through the ResearchMatch. I tried to be very active on that, mostly because I have a friend who did research. His PhD thesis revolved around finding people willing to participate in the research. I know how painful it can get. I try to be cognizant of the fact that I did a PhD too, so if I was in that position, I would be like, "Oh, my God, please, be helpful."

**Interviewer:** Anybody?

[laughter]

**Interviewee:** I know. Overall I've always been motivated in helping out with research. First was through ResearchMatch. I think because I have done a USC research before I was contacted directly through the email for this research.

**Interviewer:** Do you remember what parts of the study interested you? I know you said it was similar to the first one. Was that the main reason? What interested you specifically about this study?

**Interviewee:** It was very similar in the sense it was I think from what I remember, it was mostly around when I work out, I have to start recording it that, "Hey, I'm about to work out." Then it would ask me questions in between. It was sometimes a little challenging. [chuckles] I'll be in the middle of a plank, for example, I'll keep my phone in front of me, so that added a little extra element to the workout.

**Interviewer:** Yes, for sure.

**Interviewee:** This one was similar in the sense that it felt like I didn't have to do much outside my regular routine. It was a very simple addition. If I'm physically active, it would actually ask me that, "Were you physically active eight minutes ago or 10 minutes ago?" Besides this touching a button on the screen, it wasn't really much disruption in my routine, which is why I wanted to be a part of this.

**Interviewer:** Can you tell me what motivated you to continue answering surveys in the study?

**Interviewee:** Honestly, initially, I was just getting used to it, but after that, I just got used to it [laughs]. It became part of a routine almost. I didn't have to really be conscious of the fact that I have to answer the surveys, et cetera.

**Interviewer:** Talk about compensation. How important was compensation for you in the study?

**Interviewee:** Honestly, until you guys used to tell me that, "Oh, this much has been deposited, I didn't really remember that. Okay. Yes. I'm getting some money deposited for this." [laughs] The compensation was good, but it wasn't the main motivating factor. Like I said that just being a PhD student myself or being a part of the research community myself in the past, I understand the importance of this research and how it can be impactful. Especially for research which relies on actual data from humans, it's critical. I'm cognizant of the part of the work that goes in the research and I wanted to help out as much as I can.

**Interviewer:** Well, we thank you for that. Thank you for-

[laughter]

**Interviewer:** -volunteering for that.

**Interviewee:** I think it's, again, one more thing was that I didn't think I would be that busy. I got contacted by USC Research again for another part of the study, and I don't really fit into the criteria, which is why I'm not participating. Probably one more reason I will take a break is because this work is busy at this time. Fortunately, when I started, I was okay and I'm still within one year of my full-time job. I'm not that that busy. This was a good time to participate.

**Interviewer:** The world a year ago was a little bit different. I don't know. COVID has changed so many things that it's hard to-- I don't even know what day it is anymore all that. It's changed.

**Interviewee:** Yes. Exactly.

**Interviewer:** Talking about the burst periods, can you describe the process of answering phone surveys throughout the day on a typical burst day? What was that process like for you?

**Interviewee:** I think it was easier for me to answer the burst surveys in the morning. Sometimes I knew that minimum I have to answer eight. I used to make sure that in the morning when I'm at home, I'll keep my phone with me just so that I can answer at least eight. If I'm home the whole day, sometimes I used to answer even more. After the eight were done, it at least mentally, I was like, "Okay, I'm going out now. If I miss my phone vibrations, it would be easy." I wouldn't have to be consciously looking at my phone all the time. Again, when I'm at home, I feel like I do more activities. When I'm outside I'm just sitting and hanging out. [laughs] I think just besides the fact that I have to be a little conscious of where my phone is, that was the only thing that changed-

**Interviewer:** That changed, yes.

**Interviewee:** -for the rest of the day. Because every other day non-burst periods it would be my watch. I wear the watch anyways.

**Interviewer:** What would have made participation in the study more fun or rewarding for you as a participant?

**Interviewee:** I don't know. This one's hard. I can't really think of anything right now. Probably, one more thing I would say is, this is something of a feedback. In terms of the people around me, let's say I'm hanging out with my family and I'm charging my watch, it would give me the surveys even when I'm charging the watch. It scared of a few cats for my mother-in-law. [laughs]

**Interviewer:** It's so loud. It is.

**Interviewee:** It is.

**Interviewer:** Especially on the charger.

**Interviewee:** Yes. It's so loud vibrations. I don't know if that can be a software improvement in the app to detect when it's on charge it can be-- because when it's charging, it's not necessary the watch is going to be next to me or next to whoever is participating I guess. Other than that, I don't think I have any other feedback compared to that. Yes.

**Interviewer:** For this next section, what you were just talking about the burden of the sound of the vibration on the charger. I want to know a little bit about those burdens of increased burden that you might have faced in the study. Or any challenges or anything. Were there any situations in which it was particularly challenging to answer or survey?

**Interviewee:** Yes. I think charging the watch as one example. Another one is, let's say, because especially around COVID, we avoided flying, so we drove a lot if you have to visit family. When I was driving, sometimes I would be sporting network or, and that was especially challenging during the burst periods, I guess because if I'm driving somewhere long distance around the burst period or if I am the one driving. Sometimes, I don't know if I should have done that, but if it's a phone survey, I would ask my husband to read out the questions for me and I would say yes or no, whatever. Otherwise, I think during driving I couldn't answer, for example. Sometimes I'm very, very busy meeting days it was very hard to answer.

Those are the times I used to keep my phone on this side sometimes because keeping it on the desk would cause a lot of vibration, and I didn't want it to disrupt the meeting, especially when I'm talking. Sometimes it was a little distracting when I get a survey and I'm on the call and I will end up looking and just, even though it takes a second or two seconds, it'll just shift my focus a little bit. Nothing that affected my focus because I would just get back into what I was doing, but sometimes those breaks would be a little stressful. Again, I think the biggest-- It wasn't really, I wouldn't call it a stress, but I had to be cognizant of where my phone is around the burst period times.

Sometimes the watch charging used to take longer. This has happened to me. I would put my watch on charge and forget about it because I was doing something else. That has happened. I don't know, maybe-- This was really good though that getting the notification on the phone was amazing because I would literally forget that the watch was charging, but if I get the notification and I check it-

**Interviewer:** Yes. Then you go get it.

**Interviewee:** -I remember to wear it. I think sometimes I used to get a little tired wearing it at night because I like to not have even my rings on me if I'm-- Otherwise, it wasn't that bad.

**Interviewer:** Besides like missing a survey, was there ever a situation in which you preferred to just dismiss a survey?

**Interviewee:** Not really. No. I think, yes, the one time I've dismissed a survey is I'm on a video call on my phone, and I didn't want to be going to the service screen or my phone was literally sitting over there. That's the only time I think I have really dismissed a survey or when I'm driving, and it blocks a top thing of my map. [laughs]

**Interviewer:** Yes, I know, you're not the only one to say that.

**Interviewee:** Yes. That time I dismiss say just like go. [laughs]

**Interviewer:** Yes. I'm trying to see where I'm going here. Coming up on an intersection. What did you typically tell your family and friends when they asked you about the study? Did you have a-- Or coworkers like that? If they ask for it?

**Interviewee:** Coworkers usually never asked, it's usually my family, especially when the watch will vibrate, they're like, "Whose watch is that?" [laughs]

**Interviewer:** What is that noise?

**Interviewee:** Yes. [laughs] My phone will vibrate because these survey vibrations are very different compared to when you will get a call or text. I would tell them that, "Yes, I'm participating in the research study and again, my mother-in-law is also in university, so she was very interested in this. She was like, "Oh, that's really cool that you do that." Overall I got a very positive response.[laughs]

**Interviewer:** You get some mother-in-law bonus points. That's good.

**Interviewee:** I know. Yes, exactly.

[laughter]

**Interviewer:** For this next section, this last section here, I want to learn a little bit more about response accuracy. Besides not answering some of the questions, we're curious if there are other ways that you dealt with some maybe challenges or burdens while answering a survey just in life. How did you typically handle distractions when taking a survey?

**Interviewee:** I actually, sometimes my husband would be talking to me and I would be like, "Give 30 seconds." [laughs] Hold that thought? [laughs].

**Interviewer:** What is it?

**Interviewee:** That's what I used to him, but he got used to it too, so he is fine.

**Interviewer:** That's so funny. That's awesome.

**Interviewee:** Other times I would-- I think my family also got used to it after one point that they knew, almost everyone knew that I'm doing this, so they would repeat it for me. [laughs]

**Interviewer:** Yes. She's taking a survey, let her do her thing.

**Interviewee:** Yes. Coworkers never really ask because I, again, if it's on my watch, I answer the survey, but if it's on the phone, I would not pick up the phone when I'm in a meeting.

**Interviewer:** Were there situations in which your responses to either one of the surveys or questions may have been less accurate?

**Interviewee:** You know, that has happened a few times when I would go back because I'm in the flow of hitting what I'm used to. I would go back, it's like, "Wait, hang on." I would say yes when I am distracted with work especially, sometimes it would call it there would be 70% accurate instead of like a hundred percent accurate. Mostly because I'm just like, yes, maybe, maybe or something, majority like the 90 to 95% of times throughout the whole year, I have tried to be really accurate on this.

**Interviewer:** Do you think your responses changed throughout the day? Do you think your responses in the morning were different versus the evening or if you're at work?

**Interviewee:** They definitely are. I have had days when it's been really stressful at work and I have had like stressed out. Hell yes. [laughs]

**Interviewer:** Yes. What was that option?

**Interviewee:** Frustrated. Yes. Sometimes that too. Mornings are usually, yes, everything is great, but evenings are, sometimes it will depend how the day was. that has definitely happened. But I think in the burst periods sometimes when I'm busy in the evenings and I do my surveys in the mornings, I would say they might be a little more biased to the moment of the day. If I space my burst surveys I'll throughout the day, they vary. Unless I'm hanging out at home, I guess doing nothing [laughs] Yes. That's pretty much the same.

**Interviewer:** Yes. That changes things. Let's see. How do you think your motivation or accuracy changed the longer you were in the study or as you were in the study longer?

**Interviewee:** It. Yes. Just because it was a routine and I got I knew what questions will be asked, how in between there were some questions just to test if I'm actually focused on not that's select the fourth, or this was my favorite who is a president, and they would be like, Mickey Mouse. I'm like, I wish I can click Mickey Mouse. I think overall it just became I got more used to since it became a part of the routine, I would like, it wasn't too much of an effort to answer accurately.

**Interviewer:** Okay. Well, you answered my last question. I was going to have this interview. What did you think about those messages that weren't related? Like the president ones?

**Interviewee:** I love them. Sometimes I had to go back and like, so again, because I was anticipating it's a different question, we can go next. I'm like, wait, no, that was not the question. I would go back and then select the correct one. [laughs]

**Interviewer:** Yes. Where's my Mickey Mouse question?

**Interviewee:** Exactly. also, I really like the last, sometimes you guys have really some written messages after I finish a survey, those are really nice.

**Interviewer:** Oh, at the end of like the little trivia facts and what the jokes and stuff.

**Interviewee:** Yes.

**Interviewer:** Good. Oh, I'm glad. Let's see, any points that we didn't cover that you'd like to discuss before I move on to the last part?

**Interviewee:** Actually, no. I think, yes, no, we pretty much covered it all.

**Interviewer:** Okay. Well, thank you for answering all of those. I know those were a lot, so thank you.

**[00:19:51] [END OF AUDIO]**
