# Supplementary material for: Burden and Inattentive Responding in a 12-Month Intensive Longitudinal Study: Interview Study Among Young Adults
Source: JMIR Form Res. 2024 Aug 2;8:e52165. doi: 10.2196/52165 (PMC11329843; doi:10.2196/52165)
Supplement: Multimedia Appendix 1 [file formative_v8i1e52165_app1.zip › Transcripts/genderradiantlycharging_audio_4.25.22.m4a.docx]

**Interviewer:** To start, can provide me with some of your overall feedback regarding the study?

**Interviewee:** For the most part it went pretty well. There were a few glitches, but most of it was able to be sorted out. I do know this last first survey, I think the last day of it a couple days ago, I never got the end-of-day survey. I know that that did happen a couple of times throughout, where even though I was on my phone and waiting for it, it hit bedtime and it still hadn't shown up with the burst survey. Other than just minor little things like that, it went pretty well.

**Interviewer:** Was sleep time and wait time, were set the same and it just never showed up?

**Interviewee:** Yes. This last time, I meant to go in a minute before if it hadn't shown up, and delay the sleep time to see if I could get it to prompt that. I was doing something and I had the phone right next to me, waiting for it, and then I looked at the clock and realized it was right at time and I wasn't able to change the sleep time, so I never did get that one.

**Interviewer:** Another question I have for you. Is there anything that you're going to miss about the time study?

**Interviewee:** I did like especially on the burst days, having that pop-up, it helped me to reflect on how I was feeling. I did actually like that aspect of it, making me stop and think and be a bit more mindful throughout the day of how I was doing. Also, it helped me to realize a lot more this entire study, how active I actually am.

I have livestock and a lot of animals, so I'm actually pretty active throughout the day. I'm not big on actual exercise like most people think, going to a gym or actively setting aside time to be exercising during the day. I was always like, ''Oh no, I'm not that active,'' and this has actually helping me to realize I really am a lot more active throughout the day than I had realized before. That's definitely have had a very positive impact.

**Interviewer:** What livestock do you have? I'm so jealous. I love that so much.

**Interviewee:** Oh, gosh. We have seven alpacas, two potbelly pigs. About 140, 150 pounds sulcata tortoise, chickens, ducks, the chinchillas, they're inside animals. Then we have 12 dogs and more cats than I'm open to counting. [laughs]

**Interviewer:** That is amazing. You must have so much land then.

**Interviewee:** We have about three-quarters of an acre.

**Interviewer:** That's amazing. Did you say 12 dogs?

**Interviewee:** Yes.

**Interviewer:** I have one and I'm like, I don't know, one dog is hard enough, 12? That's amazing.

**Interviewee:** Oh, they're not little dogs. 9 Siberian Huskies and 3 St. Bernard's.

**Interviewer:** Oh my gosh. Are they indoor? Do you have dog hair everywhere all the time?

**Interviewee:** Oh gosh, all the time.

**Interviewer:** You just live in dog hair?

**Interviewee:** Pretty much.

**Interviewer:** I feel like I'm a professional vacuumer because I'm just always vacuuming dog hair.

**Interviewee:** When we bought this house, we put in all hard floors.

**Interviewer:** Smart.

**Interviewee:** So it's sweeping, it's just constant sweeping all the time. Then we have very sandy soil. At the yard, huskies dig, of course. The backyard is theirs, we don't try to plant grass because it's not going to survive. Then they just track in all the sand too, so it's just sand and hair 24/7.

**Interviewer:** Just all the time?

**Interviewee:** Yes.

**Interviewer:** It's like in food, it's everywhere. I feel like I live dog hair. [laughs] I can't imagine what you're going-- That's amazing, though. I love that so much, I am so jealous. That is amazing.

**Interviewee:** Oh yes. It's a lot of work, but we love it. I definitely couldn't do it without my husband's help at all.

**Interviewer:** You're in Northern California then, right? You must be, if you have some land like that.

**Interviewee:** Central, actually. We're about two hours south of Sacramento. We're in a little agriculture town. Our house, funny enough, it's in the middle of town, but we're in a little area that's unincorporated, so we're county.

**Interviewer:** Oh, awesome.

**Interviewee:** We've got the three-quarters of an acre and we're able to have the animals, but literally, if you go to the end of our street to the car street, you step onto that street in your city

**Interviewer:** [laughs]. Then you got lucky then, you got the perfect spot.

**Interviewee:** Exactly. It works out great.

**Interviewer:** Oh, I love that. That is so awesome. I'm jealous. I guess get my mind back in this. For this next section, we want to know about your motivation and how you heard about the time study, and a little bit about your experience in participating in the study. How did you learn about the time study?

**Interviewee:** Almost positive, is through ResearchMatch.com.

**Interviewer:** Do you remember what features about the study interest you to decide to participate?

**Interviewee:** Oh, gosh, [chuckles] I go back a year. It was something different, then I do a lot of the online surveys and I do get stuff from ResearchMatch regularly. Usually, it's just answering a few questions online. Some of them, it just depends on how much time I've gotten to dedicate to those. This one was more interactive, so that seemed really interesting. Other than that, it was something different than most of them.

**Interviewer:** Typical?

**Interviewee:** Yes.

**Interviewer:** Can you describe to us what motivated you to continue answering surveys in the study? Throughout the study, what kept you wanting to save?

**Interviewee:** Other than the payment, obviously, [laughs] that certainly helps. It was just to prove I could stick with it. I'm drawing a blank on the phrase I'm wanting, but just proving that I could stick with it and do the entire year.

**Interviewer:** Like you said, you mentioned about money. Obviously, money is motivating. How important was compensation for you in this study?

**Interviewee:** Probably about 50-50. I don't know that I would've stuck with it quite, as much as I did want to have that motivation to get into the end. There were a couple of months where it was getting difficult, especially near the end there. I don't know, I'm not sure if I may have managed to get through to the end if it wasn't for the compensation for the amount of time being put into it.

**Interviewer:** Besides paying more money because that would, of course, be more motivating. Is there anything else that we could do to increase motivation for you as a participant?

**Interviewee:** No, not really. I think I did pretty good. If I had any questions or concerns, you guys were good at getting back to me on it. One thing maybe, the emails. I did like getting the emails, like I mentioned, when you guys had done when you reached all the participants. If those may be a little bit more frequent, it might be nice because that was motivating to see how it was going overall.

**Interviewer:** Seeing data or seeing other participants, like demographics, things like that?

**Interviewee:** Yes. The demographics that was within the study and showing that, it was fun to watch. You go through the emails and see who all was participating and just realized it was a group effort. All these people involved in it.

**Interviewer:** Good to know that it's not just you in the study, that there's a bunch of other people in it as well.

**Interviewee:** That reminder was definitely motivating.

**Interviewer:** Can you describe the process of answering phone surveys on a typical burst day? How you typically went about it or what the process was for you?

**Interviewee:** One thing that was helpful was that the question, with the exception of sometimes that there was the random ones in the middle, but the basic questions were in the same order, so I knew when it started up. I could have in my mind and go back over the-- since the last time I answered, what have I been doing? How have I been feeling? I was able to just go through that without having to stop and individually think on each question.t I had that kind of, this is the next question and go with that. Then just keeping that in mind as a whole, instead of individually.

**Interviewer:** It helped having the same order for each one?

**Interviewee:** Yes, definitely.

**Interviewer:** How many phone surveys do you think you answered on a typical day, burst day, I should say?

**Interviewee:** That I answered, probably around 12 average a day.

**Interviewer:** Did you have a goal that you were trying to reach for each burst day?

**Interviewee:** Usually trying to aim for about 13.

**Interviewer:** Would you keep track of it because the app would show how many you answered or how many were prompted? Would you be mindful of that or ever check that?

**Interviewee:** Yes, I did. In the beginning especially more so, I actually had gotten a new phone part way through and my new phone, the way that the notifications were set up in order to keep it from being a large notification that took up space and minute, I had to remember to actually click on it to check. I wasn't doing it quite as frequently, but I was still going through and checking, especially near the end of the day, as it was starting to get more towards evening to see how many I had gotten. Yes, especially in the beginning I was watching that a lot more.

**Interviewer:** What would have made participation in the study more fun or rewarding?

**Interviewee:** I think like I said, the emails and seeing the demographics of other people involved. Other than that, nothing I can think of.

**Interviewer:** For this next section, I just want to understand some situations of increased burden that the time study might have caused. Obviously, we know that participating in the study was not easy, it was a long time and there was a lot that went into it. Of course, we appreciate you participating. We just want to learn more about the challenges that you may have experienced from the study. What were some situations in which were particularly challenging to answer surveys?

**Interviewee:** For one, the big work thing that I had. I'm a wedding planner now and so with one of my wedding day landed on a burst period, so that was pretty much not happening almost at all during the day. That was probably definitely the most difficult one. Other than that, most things, meetings and things like that, I wouldn't miss more than one typically.

Outside of that one big social events, it did get complicated because having to stop, especially if you're socializing with people and having to stop then, okay, do I either explain to them what I'm doing and then try to explain to them the whole study or just, "Give me a minute, let me do this real quick." That got kind of a thing. I'm not super sociable, so it wasn't too many times.

Other than that, I think the other times it really caused me major issues was typical marital stuff. If my husband and I are arguing or getting into it and my phone goes off, he got to know the notifications, so then he's going, "Just deal with," and I'm like, "It can wait, we need to deal with this. It's more important." That did happen a couple of times.

**Interviewer:** That's a good point, for sure. Just interrupting formal married life.

**Interviewee:** Yes, exactly.

**Interviewer:** What about the app, like you're talking about like in those instances, what about the app or just the procedures of the study were more disruptive? Was it like the sound, was it having to go answer the surveys or the watch survey either one? Was it vibration? What was the most disruptive?

**Interviewer:** Probably the notification itself going off because then it's knowing that you then have to stop and do it. Like I said, my husband got to know what that notification was and so most of the time it wasn't a big deal, but during those occasional times where it was an issue and he knew what that notification meant. I'd say that was probably the more disturbing part of it.

**Interviewer:** Did you keep your phone on, like you got a sound notification most of the time then?

**Interviewee:** Yes.

**Interviewer:** Then for the watch, how about the watch, how did know it was-?

**Interviewee:** Watch was on vibrate.

**Interviewer:** What most frequently led you, besides obviously the situations that you just listed, to be unable to or to miss answering a phone survey? Although you answered quite mostly all of them.

**Interviewee:** Other than those, probably just driving. Not worth getting a speeding ticket or getting a ticket from being on my phone.

**Interviewer:** Definitely not. You're like, the study doesn't pay me that much money.

[laughter]

Your husband probably knew about it from the beginning, but when you were out at social gatherings and you did decide to tell them about what you were participating in, what did you typically tell them when they asked you about the study?

**Interviewee:** I didn't tell too many people, a couple people that are close and just explaining, "Sorry, let me answer these questions real quick. I'm participating in a research study and I have to answer questions on the watch and then on the phone some days." Other than that, I didn't really get into any specifics with what any of it was.

**Interviewer:** Just the logistical part of it?

**Interviewee:** Yes.

**Interviewer:** For the next section, we want to know a bit about response accuracy. Besides obviously not answering them if you're busy, we're curious about other ways that you dealt with some of these challenges or burdens when answering the questions and whatnot. How did you typically handle distractions when taking a survey, if you were busy but you still answered a survey?

**Interviewee:** With the phone surveys, a lot of that, like I was saying earlier, with the questions being in the same order, that helped a lot because at the start of it, I hear then get the notification go and I could run through my head real quick how I was doing since the last survey, over the last hour, so I could already have that in mind. It would allow me to, if needed, to split my attention, but still be able to accurately answer the phone questions.

The watch questions could be a little bit more difficult because it did require actually stopping to read them, so sometimes if I was missing those, it was because I couldn't quite pull my attention or wasn't registering, giving enough time. Unfortunately, that also sometimes would hit things and so then there's those times where, especially like with washing dishes, I wear gloves and so if it's going off, there were a few times where trying to move the glove and it would hit it and I'm like, "I don't even know what the question was, I didn't mean to do that." That one may have had a little bit lower on the accuracy than the phone questions

**Interviewer:** Did you ever use the undo option on the watch, if that was ever available for you? Did you find that useful to have?

**Interviewee:** Very useful.

**Interviewer:** Were there situations in which your responses, besides washing dishes, but phone surveys or watch-based, may have been less accurate?

**Interviewee:** Not that I can think of.

**Interviewer:** How do you think your motivation or accuracy changed as you were in the study longer?

**Interviewee:** Motivation to complete did go down near the end, just the last few months, it just gone. I think especially knowing that it was coming close to an end as well, it was just like, okay, I'm just ready to like let's get to the end of it and be done with it and not have to keep stopping every hour or so to answer these questions and the time it takes for that. I think the motivation was getting a little less there at the end, but definitely tried to still keep up with the accuracy on it.

**Interviewer:** Do you think the study got easier as it went on or harder because it was just the motivation to stay into it made it more difficult?

**Interviewee:** Definitely harder.

**Interviewer:** Couple more questions here. What did you or did you even notice any of the questions and messages that were not related to measuring health behaviors, routines, and mood on the phone?

**Interviewee:** On the phone, yes. There were some, they seemed almost like they were accuracy questions, making sure that you were paying attention.

**Interviewer:** What did you think of those?

**Interviewee:** Those were good. A few of them, some of the grammar ones made me stop to, "Oh shoot, which of these--?" Some of them were pretty quick to figure out, but a few of them, the grammar ones, I was like, "Oh shoot, what's the difference between a verb and an adverb?"

**Interviewer:** Like thinking back to elementary school.

**Interviewee:** Like, "Don't make me think about school."

**Interviewer:** Big schoolhouse rock. What was the song that went with verb?

[laughter]

That's funny. Do you have any suggestions for us on how we could make those questions better?

**Interviewee:** I think with those ones, maybe making the answers a little bit more obvious. Some of them, "Which of these is a state?" Those, they're pretty obvious which ones they are versus, like I said, the other ones making you go-- and maybe like nouns were a little more easier than the difference between the adverb and a verb and which one's an action.

**Interviewer:** For sure. Just a little bit more stand out easy to answer?

**Interviewee:** Yes.

**Interviewer:** Are there any additional points that we didn't cover that you would like to discuss that maybe came up in the study that you wanted to talk more about?

**Interviewee:** No. I think in the beginning there was a few times where the watch questions were cut off and that did make a couple of them a little bit difficult, but that actually seemed to improve after the first few months. I don't know if it was an update or something that was sent through, so after that it was pretty good.

**Interviewer:** That was all the questions I have for you actually.

**[00:19:50] [END OF AUDIO]**
