# Supplementary material for: Burden and Inattentive Responding in a 12-Month Intensive Longitudinal Study: Interview Study Among Young Adults
Source: JMIR Form Res. 2024 Aug 2;8:e52165. doi: 10.2196/52165 (PMC11329843; doi:10.2196/52165)
Supplement: Multimedia Appendix 1 [file formative_v8i1e52165_app1.zip › Transcripts/handcufffootloosecabful_audio_8.12.22.m4a.docx]

**Interviewer:** To start, can you provide me with some overall general feedback regarding the study?

**Interviewee:** You mean general feedback for the app and the phone or?

**Interviewer:** Either one.

**Interviewee:** Okay. Well, when I was doing the survey, it reminds me that the questions on the watch is very interruptive and sometimes I had to do stuff that is time sensitive, like cooking or things like that. I just cannot answer it or I may feel a little bit irritated. That's the most irritating thing. Else, I feel like keeping a routine of answering questions, and sometimes I fell asleep during the night, not during the night but right before when I **[inaudible 00:00:57].**

**Interviewer:** Your sleep time.

**Interviewee:** Yes. My sleep time. Sometimes I worked overnight. My sleep schedule recently, during the summer, it's not always fixed. Whenever I have a deadline or presentation tomorrow, I tend to sleep a little bit, and then wake up and do my work, and then sleep a little bit. That doesn't really reflect on a survey or reflect on the, I don't know, on the watch questions. Those days, I don't really have a planned sleep schedule or I don't have anything planned. Those days are chaotic.

Other than that, oh, yes, and I moved to an apartment that doesn't have a chime or anything. Most of my exercise or my light exercise are walking around the neighborhood. In Georgia, I didn't expect it. It rains all the time. Those days, I'm just like, "Okay, I will stay inside. There's no way I can walk." Walking really benefits my mental health. I'm a research assistant. I have to be on screen and on computer all the time. Those time when I walk outside, it really benefits my mental health because sometimes I cannot do that because of the weather.

**Interviewer:** That's a good point. That's hard.

**Interviewee:** Yes. That's hard. Then sometimes the weather is very nice but then I have a deadline tomorrow or the next day.

**Interviewer:** It's always that way.

**Interviewee:** It's hard that way, especially these months when we are having summer. It's just raining outside and I have to work inside. That's all.

**Interviewer:** Gosh. Yes, that's hard. Are you a PhD student?

**Interviewee:** Yes, I am.

**Interviewer:** That explains the late nights and presentations, and everything. Oh, gosh. I'm going to ask questions along the lines of what you just said and then maybe some other specific questions. First, I want to learn a little bit more about your experience participating in the study. First question I have is, how did you learn about the study? Do you remember how you first learned about it?

**Interviewee:** I think I signed up for research, let me just pull this up real quick. It's, oh, ResearchMatch. It was on ResearchMatch. Yes.

**Interviewer:** Do you remember what aspects of the study interested you most to want to join?

**Interviewee:** Because of the compensation and it's a longitudinal study. I know that it is repetitive but there's nothing like chaotic or it will change my schedule completely. There are some studies that will change my schedule so much that I don't even consider it. It's just predictable. The compensation's good. Then I interested in the way you design the whole thing. That is very, very compelling to participate in.

**Interviewer:** Definitely. Okay. Can you describe what motivated you to continue to answer surveys in the study?

**Interviewee:** That was sometimes when I was down, especially during Christmas. I think I got depression. Not that kind of depression but was a little bit depressed. I just finished my first semester in my PhD program and it was a lot. I just want to take some time out but then when I took time off, I feel like I don't have anything to do and I don't have any accomplishment. Answering things on the phone, answering things on a survey gives me a sense of accomplishment. Then every month when you guys loved, the balance on my card is a little bit rewarding.

**Interviewer:** For sure. Feels like, okay, my hard work paid off at least.

**Interviewee:** That keeps me on track. Yes.

**Interviewer:** Can you describe the process of answering typical or phone surveys on a typical buzz day? How that was for you?

**Interviewee:** I think recently on my second semester, on the spring and summer, I have the option-- no, I do have the option to turn off the snooze-- to snooze the buzz period. I didn't use it the first six months but I use it a little bit more frequently. It depends on the plan for my next two days. If I have presentations, if I have deadlines, I probably may hit the snooze button. On that date, I usually make sure that I don't have anything to do. I just keep my phone around, keep it charged.

Sometimes I forget it because I was doing something that I really need to do like them is coding because that requires a lot of mental concentration. Sometimes cooking is time pressure. Sometimes it's just like I was not really near my phone. I tried to keep my phone around but then **[unintelligible 00:06:46]**. Oh, especially when it was charging. Oh, there's one thing about the app, that it consumed too much energy.

**Interviewer:** On your phone?

**Interviewee:** Yes. My battery went out pretty quickly and my phone is, I think, it optimized for battery power and this too went out pretty quickly. I have to charge it, especially on the buzz day, I think it's when the phone consume most of the energy. Whenever I charge, I tend to forget to get it back, to be nearby. That was the process. I think I try as much as possible to answer the end of the survey during the buzz period and it's predictable, after a few months, it's predictable that it will pop up like two hours or one hour before I sleep. That's convenient. Yes.

**Interviewer:** Did you have a goal number of surveys that you tried to answer?

**Interviewee:** I tried to answer eight. I think you guys told me about eight or something. I tried to answer eight. Sometimes when I didn't answer or I do have a busy part of my day when I skip the surveys. I skipped a survey during those busy part. I tend to feel like the app asks me more towards the end of the day than when I try to answer it evenly throughout the day. I don't know if it's true but I feel like it. I can keep up with eight or nine surveys a day.

**Interviewer:** What was the busier part of your day? Was it the morning or was it the evening when you got most of the surveys?

**Interviewee:** I don't really have a fixed schedule. It's just based on what my work and what is the progress, and how I have to involve in that. Sometimes for the situation I just described I should say most probably the morning part or the afternoon part. The evening is-- I don't meet anyone on the evening. It's not that busy in the evening. Generally, you can say that I'm more busy when the sun is up, as well as **[inaudible 00:09:22]**.

**Interviewer:** Good. What would have made participation in the study more fun or rewarding besides paying more? Paying more would be more rewarding, of course, but what would be more funner rewarding for you?

**Interviewee:** I think the question is repetitive. There's no way around that. I don't think there's a way around that. I think the watch question, it's much, much interruptive but it's a little bit more interesting than what is on the phone. The phone is very predictable. Sometimes I know what questions will come up next. Either I already have the answer for that. That's funny.

**Interviewer:** Changing up the questions, you're saying, would be a little bit more--

**Interviewee:** I'm not really sure because changing up will increase the time that I have to spend on my phone.

**Interviewer:** True.

**Interviewee:** If I know what to expect, I click very fast, be the phone is I think it is good already. I don't think it need to be changed. It just say that's the choice you guys have to make, and it has the pro and the cons. I think the pros outweigh the cons. I think that's good.

**Interviewer:** Along those lines of the questions that would come up, what did you think about the questions and messages that were not related to health behaviors, routines, or moods that came up both on the phone and the watch?

**Interviewee:** I think they're all right. Sometimes changing is good. There's a thing. My watch, because my wrist is a little bit smaller than the band, and I don't think they're is it that size from me anywhere else, even on Amazon, it tend to **[unintelligible 00:11:34]** [crosstalk] Sometimes when I do my work and when the watch buzz, and I accidentally touched something. That's a little bit noisy, but I try to minimize it when I realized that it is like that. Sometimes when it buzz, my watch buzzes, I couldn't answer quick enough.

**Interviewer:** Yes.

**Interviewee:** The questions disappear. [crosstalk] This is sometimes, it's not like all the time.

**Interviewer:** Were you ever able to undo your answers on the watch? If it happened, were you able to, if you caught it, I guess in time?

**Interviewee:** Yes. It was once, but then it allowed me to redo the question once. Sometimes I'm like, "Oh my gosh, I hit the wrong answer," and that happens and I send the wrong message or the wrong answers. It's not like when I first discovered, I try to minimize it. It happens sometimes, but not like 50% of them. No, I don't think so. It's just like 20% or 10% of the time.

**Interviewer:** Okay. Not that many times. For this next section, I want to learn about some situations of increased burden. You mentioned that it was hard at times to answer some of the surveys. Certain situations were tough. I just want to learn a little bit more about the challenges that you may have experienced during the time study. Obviously, we know it wasn't easy at times, so what were some situations in which it was particularly challenging to answer surveys?

**Interviewee:** When I was in class-- oh, yes, there was one more thing. I tend to turn the-- not the snooze, but the do not disturb function on my watch when I'm in a meeting, or when I'm doing something and then I forget to turn it back on.

**Interviewer:** Yes. That happens.

**Interviewee:** Then the phone reminded me. Sometimes I take a nap during the day, and the watch kept buzzing. I just like, I was very irritated at the time, so I just turn it off. Not turn it off, but turn the do not disturb mode on.

**Interviewer:** Absolutely, yes.

**Interviewee:** Yes. Napping, meeting, in the class, or doing something important, like being-- I cook a lot and-

**Interviewer:** That's good.

**Interviewee:** -it requires a lot of attention.

**Interviewer:** Definitely. That's good that you find the time to cook as a student. That's awesome. I love that. [chuckles] What most frequently led you to be unable to or to miss answering surveys, phone surveys specifically?

**Interviewee:** Charging the phone. Phone is not around most of the time because of charging, because of I'm taking a bath and I forget to-- so there are times when I have to put my phone away and I forget to get it back with me. Those situations could be like I'm charging the phone, I'm taking a bath. I just leave the house for a quick errand, or the phone ran out of battery sometimes. That's almost it.

**Interviewer:** Were there ever any situations where you preferred to just dismiss a survey, like you saw it and you were like, "I can't right now," dismiss it?

**Interviewee:** Yes. There were during the meeting or when I was talking to someone, like a social engagement that I really, really have to carry out. When I'm around my boyfriend, he understand all and all, so it's easy, but then someone who are strangers or my colleagues, or other people who don't really know that I'm taking the research, I would just dismiss it.

**Interviewer:** What did you typically tell your boyfriend or family, or other friends about study, if they asked about it, what would you typically tell them?

**Interviewee:** I just tell them everything. I even send them the website of this study.

**Interviewer:** That's awesome. I love that. That's cool.

**Interviewee:** Yes. People close to me pretty understand what I'm trying to do or what I'm doing, but I cannot fully explain it to people who are a little bit far away.

**Interviewer:** Definitely. Okay. For this last section of questions here, I want to learn a little bit about response accuracy. Besides not answering, if something was too hard to answer a survey, I'm curious if there are other ways that you dealt with some challenges or burdens in the study. How did you typically handle distractions when taking a survey?

**Interviewee:** I spend most of my time alone or with my boyfriend. I would just say, "Okay, hang in there, I need to do this real quick." Usually, I will make priority for the daily survey, the weekend survey, unless I fell asleep before the survey.

**Interviewer:** It happens.

**Interviewee:** I will just tell them to hang on while I finished the survey. It's really helpful that everything is in place, I know where people are before I answer it, I know which questions will come up before I answer the current question. I know it. I respond pretty quickly and pretty accurately. That's a huge plus point. It minimized my social life, the interruption in my social life. I think that's a good point.

**Interviewer:** Were there situations in which your surveys may have been a little bit less accurate, like you just answered it without even thinking about it?

**Interviewee:** I tend not to. Sometimes I do make mistake like that. I answer it too quick, but then I tap back,

**Interviewer:** Can go back.

**Interviewee:** Yes. That I can change it and think a little bit more about that particular questions. On some days, some questions, I will have a very clear answer, but then for some other questions I may have a little bit of like hesitation. Those questions I tend to think a little bit more, but then for most of question with search predictability, I think I'm comfortable with dealing with it.

**Interviewer:** How about morning versus evening or if you were around certain people, how do you think that changed?

**Interviewee:** As I share, when I'm alone or while I'm with my boyfriend, it's pretty easy because we can pause whatever we are doing, if the situation allows, not cooking for sure. That is accurate, but then for with other people, I think it would be a little bit difficult or I may have provided the wrong answers, but they're not accurate. For morning versus afternoon, I'm not sure. For morning, let's see. I think they're probably the same, I don't think that the accuracy very between morning or evening. Morning, I have work and study, and then evening, I have house chores and work, and then cooking. It's just the same.

**Interviewer:** Last question here. How do you think your motivation or accuracy changed as you were in the study longer?

**Interviewee:** I was pretty motivated at first.

**Interviewer:** Definitely.

**Interviewee:** First six months but then the last six months is not as motivated. It was not because of the study. Mainly it is because of how I psychologically go through the whole program.

**Interviewer:** That's a lot.

**Interviewee:** The factors come from the study. I think I know that it's repetitive. Sometimes it's daunting because it's repetitive but sometimes it's efficient because it's repetitive.

**Interviewer:** Catch 22. That's good. What made the study easier or harder over time? What kind of aspects of it?

**Interviewee:** Harder?

**Interviewer:** Or easier, if it got easier over time.

**Interviewee:** It's easier because I am familiar with the phone questions. It's harder is that, I don't know. I feel like it's become so familiar, so routine that there's no need to answer all. I've been through all, I've been through a very good period, a very bad period, and I'm feeling like I cannot hit that bad period anymore. Whatever I do now doesn't have real consequences. It's complacent, I think. Then I try to hit the minimum eight answers per day in the buzz period as recommended. At least I can do that.

**Interviewer:** Thank you for answering all of those questions. I know that was a lot. I appreciate that.

**[00:22:41] [END OF AUDIO]**
