# Supplementary material for: Burden and Inattentive Responding in a 12-Month Intensive Longitudinal Study: Interview Study Among Young Adults
Source: JMIR Form Res. 2024 Aug 2;8:e52165. doi: 10.2196/52165 (PMC11329843; doi:10.2196/52165)
Supplement: Multimedia Appendix 1 [file formative_v8i1e52165_app1.zip › Transcripts/whoeverrelightspookily7_audio_7.16.22.m4a.docx]

**Interviewer:** Okay, that should be working. If any question isn't clear, you can just asking for any clarification. We'll also have time at the end of the interview in case you have any questions. I just want to learn a bit more about your experience about the study in general. How did you learn about this study?

**Interviewee:** I just randomly came across it. I usually sometimes do focus groups for just extra cash and whatnot if I have the time and convenience and I just happen to come across this. That's how I found about it, so by accident.

**Interviewer:** Yes. What about the study made you want to participate? Were there any aspects of the study that interested you and made you want to participate?

**Interviewee:** I liked the part about-- I guess I think I heard about the watch recording physical activity. I liked that part. Yes, I actually originally thought it was going to be a, 'competition' where whoever did the most physical activity and it would be shown every week or every month or something like that, but that wasn't the case. It was still fine with me.

**Interviewer:** Can you describe what motivated you the most to continue answering surveys in the study, because we know that there was a lot of surveys that you had to do every week? What motivated you?

**Interviewee:** It was the money and the convenience of it. It was just done on my phone. Very convenient.

**Interviewer:** How important was the compensation for you?

**Interviewee:** It was very important.

**Interviewer:** Can you describe the process of answering phone surveys on a typical birthday?

**Interviewee:** It was very annoying because I couldn't control where I was at certain times. Many times I would hear the survey ringing, but I was preoccupied with something else, so I just couldn't get to it.

**Interviewer:** How about on days where you could answer the birth? If you were somewhere convenient where it was easier for you to get out your phone and answer the birth? Can you describe what the process was for that?

**Interviewee:** It was much easier. However, the things were all repetitive. Usually my thing, the activity I'm doing hasn't changed over the past two or three birth surveys. It's like I'm answering basically exact same thing. If I was-- Basically, yes. It is basically the same answers because nothing has changed over three or four birth surveys that came up.

**Interviewer:** I see. When you were able to continually answer the birth surveys, how were you able to track completion or like on those birthdays where it was more convenient, did you have a goal of how many birth surveys you wanted to answer?

**Interviewee:** The goal was to do as many as I could, but hitting a minimum of 10 because I think that was the most for whatever money I can get.

**Interviewer:** Okay. Then what made participation in the study more fun or rewarding? We know that the monetary factor was a big motivator, but were there any other factors that helped you answer the surveys or made participation more fun?

**Interviewee:** There wasn't.

**Interviewer:** I also want to hear more about any challenges that you might have experienced. We know that, as you said before, that it was hard if you were in an inconvenient place. Were there any other apps or procedures that made it more destructive, like the notification or anything like that?

**Interviewee:** Some of the stuff I would say would be the wake-up times and the sleeping times, which for the most part, I can control when I wake up. As for when I would go to sleep, it wouldn't be as much control. I've had times where I would put, let's say, at 10:00 PM that I would go to sleep, and then I would answer the daily ending survey, but then I wouldn't actually be able to sleep at 10 AM. It'd be at like 12 o'clock. That was something. I believe the app consumed-- I don't know if you've got any complaints about this, but the app consumes quite a bit of battery life from the phone.

I did notice that my phone would overheat more when doing certain things, such as charging, when it normally wouldn't. At night when my phone would be roughly 80% battery, and then when I woke up in roughly six and a half hours, it would be 40%, 30%, and that wasn't the case before. I can only conclude it's the time study app. Yes, those are really my two biggest complaints, more being the battery life of the app draining the phone, but I understand, yes, I guess fully optimization or something. I don't know how the app was processed or whatever, but yes, that's the only thing I would say.

**Interviewer:** I see. Okay. We'll look into that. Thank you for your responses on that. Were there any other situations in which it was particularly challenging to answer the surveys? Can you describe an inconvenient location or situation in which it was much harder for you to answer the birth surveys?

**Interviewee:** When I'm in a vehicle and my hands are preoccupied, I definitely can't answer it. As an example, if I'm on the bus and it's crowded and I can't pull up my phone, that's an example. Also, if I'm operating any type of vehicle, I can't stop the vehicle and answer the surveys. Just scenarios where I just don't have the use of my hands or I cannot get to the phone within a certain period of time. Yes.

**Interviewer:** Okay. Were there any situations in which friends or family asked you about the study and what did you say to them if they did?

**Interviewee:** Yes, my friends and other people would see me on my phone. It'd be buzzing, "What is that?" There was some notification, "Who was it?" Stuff like that. Yes, they asked about it. I just told them the truth. I'm just doing a study that involves such-and-such and I get X amount of money for it.

**Interviewer:** I'm also curious about any other challenges or burdens you might have had while answering the survey questions. How are you able to handle the distractions when you're taking the survey? Such as when you had the ability to pull up a survey, but there might have been distractions on the side.

**Interviewee:** I would have to go somewhere else where I might not be distracted, or if I could, because the surveys would basically be the same questions, I can answer the surveys in 15, 20 seconds because it's just the same answers and it's the same questions. I'd be able to do it fast. In scenarios where I could not, I would have to get my phone and go somewhere else temporarily for 30 seconds and then come back.

**Interviewer:** During those situations in which you're answering them quickly, do you think your responses might have been less accurate?

**Interviewee:** I don't think they were, because the answers to my last birth survey was pretty much the same as the surveys I was taking because really nothing has changed. If it has, though, I would've definitely accommodated for it. I do believe my answers were very much accurate.

**Interviewer:** On the same vein of questions, do you think your responses changed if you were around certain people or if you were in a certain location or during a different time of day?

**Interviewee:** Yes, of course. Yes.

**Interviewer:** Can you describe a situation in which you think your responses changed based on those factors?

**Interviewee:** Well, probably a good example would be if I'm mad or nervous about something and then I see someone that comes with me that I like, then I'll be less frustrated or less nervous because there's someone else with me. That's an example, or if I'm not really happy like today or just for the moment right now, and then I see someone that I like, and they start talking to me, then I'd be more happy than I was a minute ago.

**Interviewer:** Okay. Thank you for your responses for that, and how do you think your motivation or accuracy changed as you were in the study for a longer amount of time? Did it get easier or harder over time?

**Interviewee:** I would say I had the same amount of motivation, and accuracy is the same. Really, I kept it consistent.

**Interviewer:** Then for the surveys, do you remember seeing any questions or messages that weren't related to measuring health behaviors or routines or your mood on the phone, like fun fact questions or anything like that?

**Interviewee:** Yes, I have seen this. A lot of them, I had seen multiple times such as, "What color is the sky? Pick the letter, number three," stuff like that. I did see those, yes.

**Interviewer:** What did you think about them?

**Interviewee:** I just assumed they were in place for people that quickly wanted to run through the surveys by potentially putting random responses as a matter of inaccuracies, and then that throws them off. If that were the case, you can spot it very quickly. If that was the intention, I don't think it was a very good one.

**Interviewer:** Oh, okay. Any suggestions on how we can make them better?

**Interviewee:** I would say, if you could-- it'd probably be bad, but if you put more sentences in the ones that you were trying to throw off, to throw the people off, which would indicate they would have to read for more than two seconds, because if I see the question, I skimmed through it really quickly, "What color is the sky?" I already know the answer, but if it was two sentences at least I would have to read those two sentences in order to know something, or if I'm thrown a math question like the standard, like, "Jack as 10 apples. Sally, it's eight. How many--?" Stuff like that, that'd require a little bit more critical thinking.

**Interviewer:** Oh, okay. We'll think about that. Thank you. Thank you for your responses and thank you for the suggestions. Just as a quick wrap up, are there any additional points or things that we didn't cover that you would like to discuss?

**Interviewee:** I don't think there really is. For me, the watch surveys, it was quite a bit of surveys. I think at the very least I would get 50 surveys at least from the watch. Despite it taking one second to answer the question, it is still just a hassle because your watch is constantly buzzing, and you might not be able to get to it. That's something, but overall, I think the survey was fine. The study was fine, I don't really have a whole lot of complaints.

**Interviewer:** Do you have any last suggestions or comments that we can do to make the study better?

**Interviewee:** I think it would be-- maybe it'd be more-- I guess as a way of creating some competition relating to physical activity, if all the watch data maybe you see-- I assume you're able to see how many steps that we've taken over the past week or month. Then I would-- and then all the people in the surveys, you could just put their last names or something like that, or the top three winners or something like that, or everyone in the survey, "These are your steps for-- do something every month," I guess, or something like that, and then provide more compensation or incentives for the people that are first, second, and third place, some kind of competition form, which would motivate people more to exercise. That's the only thing I would say.

**Interviewer:** Okay. We'll take that into account. Thank you so much for your responses on that.

**[00:14:45] [END OF AUDIO]**
