# Supplementary material for: Burden and Inattentive Responding in a 12-Month Intensive Longitudinal Study: Interview Study Among Young Adults
Source: JMIR Form Res. 2024 Aug 2;8:e52165. doi: 10.2196/52165 (PMC11329843; doi:10.2196/52165)
Supplement: Multimedia Appendix 1 [file formative_v8i1e52165_app1.zip › Transcripts/unfreezefrayingknoll _audio_5.16.22.m4a.docx]

**Interviewer:** Okay. That's all done. Before I officially get started, do you have any questions for me before we begin?

**Interviewee:** No.

**Interviewer:** Okay. First question I have for you, is there anything about the time study that you'll miss?

**Interviewee:** It was nice getting the prompts on the watch just to reflect on how I was feeling. Because maybe you don't take notice to that but then the questions would kind of be, okay, yes, maybe I am feeling happy or a little frustrated.

**Interviewer:** Nice to reflect back on the day and or the moment at least.

**Interviewee:** Right.

**Interviewer:** I want to learn a little bit about your experience in participating in the study in general, your motivation for joining the study and whatnot. How did you first learn about the study?

**Interviewee:** I think I'm part of the research list and it would just send emails. I think I got both of the USC app studies. My background is in nutrition and fitness. I was like it'd be nice to contribute to research. I've done it before, so I might as well since I'm already going to be working out. I wear a fitness app technically, so it wasn't anything-- well, it was extra. It was a lot of extra work, but not too bad.

**Interviewer:** That's right. You participated in the other study as well?

**Interviewee:** Yes.

**Interviewer:** Wow. It's been a year since then.

**Interviewee:** Right.

**Interviewer:** That's insane.

**Interviewee:** Yes, it doesn't feel like it. It went by pretty fast.

**Interviewer:** Yes. Wow. Fast year. That's crazy. I want to hear a little bit more about your motivation to continue answering surveys on the phone. Obviously, we know how hard it could be to answer some surveys on the phone. What motivated you to continue answering surveys in the study?

**Interviewee:** Trying just get to the eight minimum since that was the goal. I was like, okay, if I get eight today, that's great. Then on the other days where maybe I had some more free time, it wasn't as busy, getting the 11 was the goal. The motivation was getting that number. The pay was partly, but mostly, I just wanted to get it done.

**Interviewer:** You mentioned the pay,. How important was compensation for you in the study? Did it factor in?

**Interviewee:** Yes. I guess with the bonus for the 11, that did factor in, but the pay wasn't the most important part or the main reason. It was just trying to get all the data that I could get in.

**Interviewer:** Yes. That's awesome. Can you describe the process of answering phone surveys on a typical day? How many you answered or what it was like?

**Interviewee:** Yes. I feel like typically the first one would come maybe at an hour after waking up or what I set in for waking up. Depending on my work schedule, I could do more than others, but for the most part, it could go pretty quick just because I know what to expect and what order they come in too. With that, I was pretty quick to get through.

Sometimes something I was doing wasn't on the list, so then it just went unnoticed, I guess, which I guess is fine depending on what variables you're looking at. I feel like I was probably answering more earlier and later in the day, and in the middle was hit or miss.

**Interviewer:** Yes. More work probably went on in the middle of the day.

**Interviewee:** Yes.

**Interviewer:** Is there anything that we could have done to make participation in this study more fun or rewarding besides obviously paying more? Because that always motivates people.

**Interviewee:** It was nice getting like the little, maybe the data like you would send every maybe couple of times, showing how many I've completed and some of the different things you found so far. That was nice getting those. I feel like maybe more of those would've been nice.

**Interviewer:** Yes, definitely. Maybe a once a month would be better.

**Interviewee:** Yes.

**Interviewer:** Let's see. Did I skip a question? For this next section, I want to talk about different situations of increased burden that the surveys may have caused or the study in general may have caused. Maybe different challenges that you may have experienced while participating in this study. What were some situations in which it was particularly challenging to answer surveys? I know you mentioned like the middle of the day being at work. Does anything stick out as far as a specific situation?

**Interviewee:** I think it's more so just the watch vibrating. It's loud when it does. With work typically it was fine. I can you look down real quick and answer or just click it away to stop the buzzing, but that was sometimes depending on the situation, especially if I was with a patient. I never got anything negative from them or even my coworkers. I guess I was always cognizant of the noise of or always looking at my watch.

**Interviewer:** Watch more than the phone you're saying for the--

**Interviewee:** Yes. The phone wasn't too bad during the first periods. I guess-- yes.

**Interviewer:** What most frequently led you to be unable to or to miss answering some phone surveys?

**Interviewee:** For that, probably work because I would leave it on silent, just because that's where my phone's usually on. I would just look back and forth. If I'm on the phone, I can answer it pretty quickly. Other than that, it wasn't too bad. What was the question again?

**Interviewer:** Oh, just what most frequently led you to be like unable to, but yes, you answered it.

**Interviewee:** Yes, that and work. Sometimes I would be maybe in the middle of one, but I don't know what that time when it cut off was, and then it would just disappear.

**Interviewer:** Yes. That's frustrating. Were there any situations where you preferred to just dismiss a survey even though you saw it? Can you tell me about maybe a situation that you remember doing that?

**Interviewee:** At work, if I'm on a video call or something, I can see it's up, I'll ignore it. Or yes, driving, that's another instance. Sometimes I have someone in the car with me, and I would have them answer it for me. Other than that, that would go away.

**Interviewer:** Okay. The last question for this section, what did you say when friends or family asked you about the study? Or like you said, your patients. Say if a patient asks you about the study, what would you typically tell them?

**Interviewee:** That I'm in a research study and it asks me different questions about how I'm feeling and different activities I do throughout the day. They'll ask prompts on my watch, and then every so often, I'll get a survey every hour that I'm awake during those periods.

**Interviewer:** You're like, yes, that vibration on that watch is too loud.

**Interviewee:** Yes.

**Interviewer:** Okay. For this next section, I want to learn about response accuracy. Obviously besides not answering like if you were in a situation where you couldn't answer, we want to know if there were any other ways that you dealt with some of the challenges or burdens. Like for instance, if the watch is vibrating and you just wanted to shut it down, how did you handle those? How did you typically handle distractions when taking a survey?

**Interviewee:** With those, typically I can get through it and not be distracted. Not always was an issue. I feel like most of the times I was able to answer the little prompts that came up just to test, see if I was paying attention. I guess there really wasn't any issues really with that. If I was distracted, I might have to go out of the app into maybe my email or something, but then it would prompt me that it's time for the phone survey, I would go back and finish it.

**Interviewer:** Okay. Perfect. How do you think your motivation or accuracy changed as you were in the study longer? Like from the beginning of the study to the end?

**Interviewee:** Well, in the beginning of the study I remember, for some time, I did not see the exercise and physical activity box. I did not click that for some time. Then at some point, it went through and I was like, "Oh wow."

**Interviewer:** That's new.

**Interviewee:** Because usually, I would just start it on my watch, or if it says I exercised in the past hour, yes, but it wasn't always checking that box during that hour.

**Interviewer:** How long do you think? First six months? First four months?

**Interviewee:** It was a couple of months.

**Interviewer:** Okay.

**Interviewee:** There were some things, that again, there were no categories for. That gets left unnoticed, but I feel like as time got went on, I did get more accurate, especially with that. Although, I think it also at some point in the study, the read while sitting. With that, I think of that as just reading a book, sitting down, but then sometimes I would be reading like on my phone and maybe just like a small portion, and then it's like, okay, well, how long should I have been reading for it to count? For that, I usually only checked that if I was actually doing some reading and focusing on that.

**Interviewer:** If you were reading a book, you're saying?

**Interviewee:** Yes, reading a book or an article.

**Interviewer:** I know it's hard. Like I was reading an Instagram post . Does that count?

[laughter]

**Interviewer:** I was reading through comments on this Instagram post. Do you think the study got easier over time or do you think it got harder over time or about the same?

**Interviewee:** I would say it got easier. It was just about the same because then you just kind of get into a habit and it's just part of your routine.

**Interviewer:** Okay. Let's see. Another question here. You've mentioned this earlier, but what did you think about the questions and messages that were not related to measuring health behaviors, routines or mood on the phone and the watch, if it came up on both?

**Interviewee:** Okay. Just the random questions?

**Interviewer:** Yes.

**Interviewee:** They were fine. Some of them, I did have to think a bit for a second, like, "Oh, does the sun rise in the east or the west?" because you hear it so often.

**Interviewer:** I know.

**Interviewee:** And then once it's time to get tested, I was like, pressure. Yes. For the most part, yes, they were easy.

**Interviewer:** Easy. Do you have any suggestions for us on how to make those better?

**Interviewee:** No, I don't think so.

**Interviewer:** I'm going to ask a couple of other questions here. We're going to change up the questions a little bit. Do you know anyone else who participated in this study?

**Interviewee:** No.

**Interviewer:** We could skip some questions. Okay, so exercise. In the past month, have you exercised or performed any type of physical activities such as going for a walk?

**Interviewee:** Yes.

**Interviewer:** What type of exercise do you typically do?

**Interviewee:** Walking, indoor cycling, yoga, tennis.

**Interviewer:** Oh, wow. I love that.

**Interviewee:** Those are the main. Oh, yes, weightlifting, strength.

**Interviewer:** Are there certain days of the week that you do? Do you play tennis on a specific day? Do you strength train on a specific day?

**Interviewee:** Every Sunday I cycle because I'm a fitness instructor, so.

**Interviewer:** Oh, there you go. That's awesome. Are there certain times of the day then that you're doing this?

**Interviewee:** For the cycle, it's morning. Other days, it's evenings.

**Interviewer:** Evenings, okay. You're doing this at a gym then?

**Interviewee:** Yes. Mostly at a gym and then other times outside or inside.

**Interviewer:** Now I have some questions about sleep. Were there any instances where the phone surveys or the watch surveys disrupted your sleep?

**Interviewee:** No. For naps, I would just take it off. Again, for naps, I would just put it on silent. For the most part, it didn't. One time when I put the sleep time in, the surveys did go a little bit past that, but then it stopped. That was only the one time.

**Interviewer:** One time. Okay. That's good. Not too much disturbing.

**Interviewee:** Yes.

**Interviewer:** Okay, let me see. I think we answered most of these already. Did you have any technical problems with the phone or the watch time app that you solved yourself without our assistance?

**Interviewee:** No. Sometimes it would seem like it would be a while until the watch surveys started prompting after a first period, but it always came back within that next day. Maybe most of the day it didn't.

**Interviewer:** Yes, but it did eventually?

**Interviewee:** Yes.

**Interviewer:** Okay. Let me see here. Are there any additional points that we didn't cover so far that you'd like to discuss? Maybe something came up that you wanted us to know about?

**Interviewee:** Not that I can think of.

**Interviewer:** If anything comes up, you can always let me know.

**Interviewee:** Okay.

**Interviewer:** Okay. For this last-

**[00:16:13] [END OF AUDIO]**
