# Supplementary material for: Burden and Inattentive Responding in a 12-Month Intensive Longitudinal Study: Interview Study Among Young Adults
Source: JMIR Form Res. 2024 Aug 2;8:e52165. doi: 10.2196/52165 (PMC11329843; doi:10.2196/52165)
Supplement: Multimedia Appendix 1 [file formative_v8i1e52165_app1.zip › Transcripts/rippingpeprepose_audio_5.26.22.m4a.docx]

**Interviewer:** Okay. To start, can you provide me with some of your overall feedback regarding the study?

**Interviewee:** Sometimes the watch notifications are a little distracting, especially in class and stuff, so I would often have to turn on do not disturb. The phone ones, like the burst surveys, they weren't that bad, especially I knew about when to expect it and stuff. I think they were like pretty quick. Usually, the Sunday ones took longer because it was everything, but yes, I think the phone ones were better than the watch.

**Interviewer:** I'm going to ask more specific questions about your experience or overall in the study, but this first section of questions is going to be more about your experience in the study. First question I have is, how did you learn about the study?

**Interviewee:** From ResearchMatch, I think?

**Interviewer:** ResearchMatch?

**Interviewee:** Yes.

**Interviewer:** Okay. I know it's been a year, it's been a while, but do you remember what interested you most about the study or what made you want to join?

**Interviewee:** I think it was interesting because it was monitoring it over a long time where it was more normal habits, I guess, other than other studies where it's more specific things on one day, so I think that was interesting.

**Interviewer:** More instead of being that short-term thing, it's a year-long time.

**Interviewee:** Yes. [chuckles]

**Interviewer:** Can you describe to me what motivated you to continue to answer surveys in the study?

**Interviewee:** I think just because out of habit I was already used to it, especially on my phone. The watch sometimes I forget to turn off do not disturb, so I have to remember, but the phone since it popped up, I was already used to it.

**Interviewer:** How important was compensation in the study?

**Interviewee:** On a scale of 1 to 10, I'd say a 6.

**Interviewer:** You talked about phone surveys being your favorite out of the two compared to the watch. Can you describe the typical process of answering phone surveys on a birthday, what that was like for you?

**Interviewee:** Once the first one, the first few, were prompted, I got an idea of about what time they would be coming, so I would know to expect it. Sometimes I wouldn't change my schedule around them, but I would know if one is coming in about 15 minutes, I'd make a mental note to remember.

**Interviewer:** Did you have a goal number of surveys that you tried to reach each day?

**Interviewee:** I think on a day where I wasn't as busy, I think eight because it was the baseline, like I said. I think I just tried to do as many as I could.

**Interviewer:** Would you track completion at all on the app, when it would show you how many you've completed?

**Interviewee:** Sometimes at the end of the day I would check if I had any more left, especially the final day surveys.

**Interviewer:** [crosstalk].

**Interviewee:** Yes. I would know if I had already done it or if there was going to be more.

**Interviewer:** More coming in.

**Interviewee:** Yes.

**Interviewer:** Let's see. What would have made participation in this study more fun or rewarding for you as a participant besides paying more? That would be rewarding, of course. [chuckles]

**Interviewee:** I guess, I don't like rewarding but knowing what was being tracked in a way. I didn't know sometimes if some things in particular like heart rate or something or sleep was more important, so I guess just knowing that.

**Interviewer:** Knowing what it is. We were tracking heart rate, but hopefully, we'll provide some-- we're finishing up data collection at the end of this month and so hopefully we can get some data to you, some visualization to know what was going on with it, why not.

For this next section of questions I want to learn a little bit about situations of increased burden. Obviously, we know at times the study wasn't easy, could have been a little challenging, so I want to learn a little bit about the challenges that you may have faced. What were some situations in which it was particularly challenging to answer surveys?

**Interviewee:** I would say if I was in class or especially taking a test, because then I had to put everything away. It comes to the top of my head, on Sundays sometimes because it was a longer survey, I had to sit there longer and do it. Other than that, I think it was fine.

**Interviewer:** Did the professors make you take off your watch-bearing tests? I'm always curious about that.

**Interviewee:** They never said anything-

**Interviewer:** Okay.

**Interviewee:** -but in high school they would have me do that, so I had a habit. I was like, "I don't want to be accused of cheating."

**Interviewer:** Yes. I always think-- Smart watches were barely a thing when I was in college, so I always think about that like, [chuckles] "Are you even allowed to wear those in a test?"

**Interviewee:** I think especially in the bigger lecture halls and stuff, they didn't even notice [crosstalk].

**Interviewer:** What part, I know you mentioned the vibrator or the notifications from the watch, but what part of the study or the app was most disruptive? Was it like vibrations from both either this phone or the watch? Was it actually having to do the surveys? What was more disruptive for you?

**Interviewee:** I would say the vibrations. I don't know. They were longer vibrations. I don't know if there was a way to change that or not, but sometimes like--

**Interviewer:** Very intense.

**Interviewee:** Yes, because on my phone sometimes if I have a phone call, if it vibrates too long, I turn it off because I can't stand the vibrating thing.

**Interviewer:** [laughs]

**Interviewee:** [chuckles] I'd say maybe just the fact that I was being notified, but doing the surveys themselves weren't that bad.

**Interviewer:** You're talking about the vibration on the watch, right, that was really long.

**Interviewee:** Yes.

**Interviewer:** Was it the phone too? Yes. It's ntense.

**Interviewee:** Yes.

**Interviewer:** [laughs] What most frequently led you to be unable to or to miss answering surveys?

**Interviewee:** For the watch, I would say if I turned on do not disturb and then I forgot to turn it back on. For the phone, if I was away from my phone, then I wouldn't hear the vibrations, but if I was nearby, if I heard it or I was on my phone, then I would answer them.

**Interviewer:** What about dismissing? Did you ever prefer to dismiss a survey?

**Interviewee:** On my phone, if I was doing something that was timed,I had to be on my phone, then I would. Usually, if I wasn't then I would just answer.

**Interviewer:** What did you typically tell friends or family or classmates about the study if they asked?

**Interviewee:** My family, I would tell them that I was doing a study if they asked about the surveys and stuff. I don't think my friends ever asked. Classmates didn't notice, so.

**Interviewer:** They didn't hear the stupid vibration on the watch?

**Interviewee:** No, I think they just thought it was a text or someone's calling.

**Interviewer:** [laughs] They're like, "Gosh, she gets a lot of text messages on her watch."

**Interviewee:** [chuckles] Yes.

**Interviewer:** [laughs] This next section of questions, I want to learn a little bit about response accuracy, so besides not answering, I'm curious how you dealt with other ways, some other challenges or burdens. How did you typically handle distractions when you were taking a survey?

**Interviewee:** If I was in the middle of the survey and I was distracted?

**Interviewer:** Yes.

**Interviewee:** Usually, I would finish the survey, I'd be like, "Hang on a second," and try to finish it. Sometimes if it was important and I had to leave, I would stop the survey in the middle. I would leave it open, but I think when I got back it had closed out.

**Interviewer:** Were there certain situations in which your responses may have been less accurate? You just didn't really think through your responses, you just answered them.

**Interviewee:** I think most of the time I would be reading them-- They're pretty accurate. I think after some time I knew what the next question was going to be, so I already knew which one to put on, which option to select. I think for the most part I tried my best to make them accurate.

**Interviewer:** Do you think your responses changed if you were around someone else or morning versus night? Did you notice that your responses would be a little bit different during those times?

**Interviewee:** If I was around somebody, usually, they weren't looking at my phone. Those would be accurate. I think if something had happened, especially with the first ones or even the end of the day ones and the non-first days, if something had happened recently within the past hour or so that I remembered then my answers would reflect that. If something frustrated me in the morning and then by the end of the day I forgot about it, I don't know if that would be-- if I had remembered it at the end of the day.

**Interviewer:** When you're answering the end-of-the-day surveys, along those lines, when asked about how you felt over the past day, did you pick something out that stuck out to you? Did you sum up your day? How did you generally pick an answer for that one?

**Interviewee:** I tried to take an average, I guess, of my day. If I was really nervous about something in the morning and then a test or something, and then afterwards I was fine, I always tried to make it reflect both parts of that.

**Interviewer:** How do you think your motivation or accuracy changed as you were in the study longer?

**Interviewee:** I think for the watch, it definitely went down. I think for the phone surveys-- I think at some point it just became a habit, so I was already doing it, so at times I would forget that it was even a study and stuff. I was just doing that.

**Interviewer:** It's going to be weird now not getting all of that-

**Interviewee:** I know. I don't have to-

**Interviewer:** -with your adjustment.

**Interviewee:** -place it separate time on Sunday for the survey.

**Interviewer:** [laughs] Got all this extra time in the world now on first weekends. [chuckles] Last question here along the lines, but not really. What did you think about the questions and messages that were not related to measuring either health, behaviors, routines, or mood on the phone?

**Interviewee:** I thought they were something to make sure people were answering them correctly. They weren't just touching random ones. Sometimes I would know which question was coming next, and then that would throw me off [crosstalk] I'm like,-

**Interviewer:** Throw you of.

**Interviewee:** -"I have to answer this." [chuckles]

**Interviewer:** Were any of them memorable? Any of them stick out?

**Interviewee:** I think things where it would ask about, "Which of these is a famous astronaut?" I was like, "Wait, let me remember what each of these people do." Other than that, I think they're just random ones.

**Interviewer:** Let's see. Are there any additional points that we didn't cover that you would like to discuss or anything that came up over the year that you wanted to tell us about?

**Interviewee:** No, I don't think so.

**Interviewer:** Thank you for answering those questions.

**[00:14:11] [END OF AUDIO]**
