# Supplementary material for: Burden and Inattentive Responding in a 12-Month Intensive Longitudinal Study: Interview Study Among Young Adults
Source: JMIR Form Res. 2024 Aug 2;8:e52165. doi: 10.2196/52165 (PMC11329843; doi:10.2196/52165)
Supplement: Multimedia Appendix 1 [file formative_v8i1e52165_app1.zip › Transcripts/backfirebankedprudishly_audio_5.31.22.m4a.docx]

**Interviewer:** Hi. If the question is not clear, you can ask me for clarification. We'll also have **[inaudible 00:00:07]** at the end to any questions that you have, but do you have any questions now before I begin?

**Interviewee:** No. You can start.

**Interviewer:** I wanted to first learn a little about your experience participating in the study as general. How did you learn about this study?

**Interviewee:** I heard it through the Grapevine. Some of my family members said that they heard of an opportunity of which I could make a little bit of money potentially.

**Interviewer:** Oh, okay. [crosstalk]

**Interviewee:** They forwarded me that information. Sorry.

**Interviewer:** Oh, no, it's okay. Then was there any other features of the study that made you want to participate such as other than the compensation?

**Interviewee:** Well, I did find it very interesting, the idea of participating in something really long and intensive just doing something good for science, I guess.

**Interviewer:** Thank you. Can you describe what motivated you to continue **[unintelligible 00:01:20]** in the study?

**Interviewee:** I guess getting paid was a big one. College student, anything helps. That was a big one I guess. I guess science major. I like science. Would've been nice to participate in a study like that, especially if I could put it on my resume or something.

**Interviewer:** Then was the ease of answering the survey questions also motivating?

**Interviewee:** Yes. They were very easy. I didn't really feel like it was super difficult to follow. Yes.

**Interviewer:** Then on a typical birthday, can you describe the process of answering the phone surveys?

**Interviewee:** On a typical what? Birthday?

**Interviewer:** A birthday.

**Interviewee:** Well, usually, I answer the surveys pretty quickly. Sometimes it's pretty unavoidable when I can't answer it such as when I'm busy with something else and I can't immediately get to it, but I've been pretty good about trying to get to it on time. I used to take a moment and I think about what I am feeling in the moment to answer the questions correctly, which has been pretty helpful in tethering myself, very emotionally rewarding almost if it makes sense. Reminder to slow down.

**Interviewer: [unintelligible 00:03:08]**

**Interviewee:** Thank you.

**Interviewer:** Did you have a specific number of goals or surveys that you were trying to reach?

**Interviewee:** It wasn't really how much I wanted to reach. I really just wanted to try to get all of them honestly, but I didn't have a set number in mind. It was just trying to get as many as I can and if I missed some, but usually, I try to get more than eight, the number that--

**Interviewer:** Thank you so much. Then what else do you think would've made the participation in the study more fun or rewarding?

**Interviewee:** I'm not really sure. I guess there's only so much you can do, but in and of itself, I thought it was a pretty good study. I didn't have a lot of issues with it. I'm not sure how to make it even more rewarding than it already was.

**Interviewer:** Oh, okay. I know participating in the time study was not easy and we really appreciate all of your participation and all of the hard work you put in to help us get data. I wanted to learn more about any challenges you might have experienced. Did you have any situations in which it was particularly challenging to answer the surveys?

**Interviewee:** Yes. It was particularly hard to answer surveys when I'm taking a test or in class or at work. Sometimes I could a lot better. Other times, it was just not possible for me to do it in time, but other than that, it was pretty simple to keep up with.

**Interviewer:** Then were there any questions or things you noticed on the surveys that were weird or were hard to answer, like questions in particular?

**Interviewee:** All the questions were pretty straightforward. There were a lot of questions that were pretty repetitive or questions that I couldn't really say no to because I would be given something like often, sometimes, or rarely, but there would be some moments where my response would be something like, "I've never actually done that, so I can't say sometimes or rarely because I've never done it rarely because I've never done in general," if that makes sense.

**Interviewer:** Yes. Do you have any examples of a specific question that you remember that was on?

**Interviewee:** Oh, that's a very-- let me think. I think it was an exercise question, something about how often I go to the gym, I guess, but I've never really been inside a gym. I've usually done my exercise in other ways and stuff. Gym memberships are expensive.

**Interviewer:** They are.

**Interviewee:** Yes. I couldn't really say rarely to that one, but I chose that one anyway because there wasn't an option for never. I think that one is one example. I can't really think of any others off the top of my head, but yes.

**Interviewer:** Were there any procedures that you found the most disruptive such as the end-of-day survey or anything that disturbed you when you were trying to **[unintelligible 00:07:19]** something?

**Interviewee:** The end-of-the-day surveys, for the most part, I tried to time them around when I was getting ready to sleep, going to bed. Not directly at the moment where I'm trying to fall asleep so that I don't get interrupted by the loud buzzing if that makes sense. I didn't really have much of a trouble working around that.

**Interviewer:** Then did you also find any of the notifications either on the phone or the watch disruptive or annoying in any way?

**Interviewee:** Yes, definitely, especially for the watch. It would've been nice if I had some way to turn down the vibration because I could still very much hear the notifications. It's just that they were too intense.

**Interviewer:** We'll keep that in mind. Then what do you say if friends or family asked you about the study or if they noticed that your watch was buzzing a lot, especially on the days with lot of surveys?

**Interviewee:** Surprisingly enough, not a lot of people have asked me about that, which I find very interesting, but more people have asked me about that. I usually said I'm just participating in a study. I didn't really go into a whole lot of details I guess. They usually just left it at that. They weren't very curious.

**Interviewer:** That could just be them not being very curious. Then was there anything else that led to you not answering a survey other than quizzes or test or university classes?

**Interviewee:** No, not really. The only times I really didn't answer some questions is either when I missed it due to those circumstances.

**Interviewer:** Then besides not answering some of the surveys because of the situation, I'm also curious on how you dealt with some of the other challenges or burdens. How did you handle distractions when you were taking the surveys?

**Interviewee:** Well, for the most part, I was able. For the first maybe a few weeks, it was really destructive, but eventually, I got into a routine where I could more comfortably answer it and immediately go back to my work like getting used to the idea of being prompted on the phone or on the watch, and then I'll go straight back to what I was doing. Before then, yes, it was a little bit difficult of an adjustment to get down. It didn't take too long before I could do it.

**Interviewer:** Can you go into a little more detail about how you were able to set up this routine of answering the questions on the watch?

**Interviewee:** Well, I suppose it got easier to understand the pattern that was going on. It felt like they weren't exactly super randomized, I guess. I could feel they come every while and usually at the same amount, so I wasn't constantly disrupted-disrupted. I knew they were going to come at certain times.

**Interviewer:** Okay. Thank you. Were there any situations while you were doing the responses that may have been less accurate? Like if you ever answered without thinking about your response and wanted to change it.

**Interviewee:** That has happened a lot, which is really nice on the watch because I can just undo it. I think that was a very good feature. There were sometimes where I did think **[unintelligible 00:11:30]** but now with the watch prompting me, I could actually take a moment to think, "Oh, wait, is this actually how I feel?" That helped me. I could answer more properly.

**Interviewer:** Did you ever notice if your responses changed, if someone else was around, or depending on your location or what time of day it was?

**Interviewee:** I don't think I could particularly notice anything different from my surroundings, because if that was the only answer it might be a little bit more relaxed.

**Interviewer:** Okay. How do you think your motivation or accuracy changed as you were in the study longer? I know earlier we just talked about how it became easier to set up a routine later in the study. Was anything else about the study that made the motivation better or your accuracy on the survey questions?

**Interviewee:** Well, I suppose as I started using the watch more and more for daily functions, I realized that it was pretty really useful, honestly, and that kind of motivated me to keep on going as well. I started to feel like I could actually go through this 12-months study because it wasn't difficult to do. It didn't require a whole lot of hard effort and thinking. I just had to go through it and that helped me a lot too.

**Interviewer:** All right. Thank you so much. One last question on this section. What did you think about the questions and messages that weren't related to measuring health

behaviors, routines, or the mood? These were like the fun fact questions that we sometimes had at the end of the surveys or the thank you messages.

**Interviewee:** They were kind of cute. They were cute. That weren't bad.

**Interviewer:** Okay. Do you have any-- Oh, please go on.

**Interviewee:** I liked reading them after each one, lots of interesting facts. Some I knew, some I didn't. That was kind of nice to know.

**Interviewer:** Do you remember if any of them were particularly memorable?

**Interviewee:** I'm not sure if memorable or not, but I have pulled a few of them out for some people.

**Interviewer:** Okay. All right. Do you have any suggestions on how we can make these messages better?

**Interviewee:** I rather liked them. I think they were nice as they were. I liked the uplifting messages about having a good day and stuff.

**Interviewer:** All right. Thank you so much for your responses on this. We're going to change up the questions a little bit. **[unintelligible 00:14:38]**, I know you mentioned some family members who knew about the study. Do you know anyone else who participated in this study?

**Interviewee:** No, just **[inaudible 00:14:53]**

**Interviewer:** All right. Who in your family also participated in this study? Can we get their name?

**Interviewee:** Maria Adriana Rojas. R-O-J-A-S.

**Interviewer:** Okay. What is your relationship with them?

**Interviewee:** She is my sister.

**Interviewer:** Okay. How frequently do you interact with her?

**Interviewee:** Not super often. We live in different parts of the state, **[unintelligible 00:15:25]** after college but we talk sometimes via text.

**Interviewer:** All right. If you were to categorize how often you interact with her, would you say once a month or once a week?

**Interviewee:** A couple times a month, maybe less.

**Interviewer:** Okay. Now we're going to talk a little bit about your physical activity. In the past month, have you exercised or performed any type of physical activity, like going for a walk?

**Interviewee:** Yes.

**Interviewer:** Okay. What time do you exercise?

**Interviewee:** What times?

**Interviewer:** What types?

**Interviewee:** Types. Oh, well, I do a lot of walks. I usually walk to work, which is pretty much a mile away or so or less. My work has a lot of physical aspects to it. A lot of lifting, a lot of carrying things, a lot of walking back and forth, and cleaning. I get a lot of my energy used up with that. I have a swimming pool in an apartment complex near me, which I like to visit sometimes.

**Interviewer:** Okay. You would walk to your work on every business day. How about for swimming? What days of the week do you usually do that and what times of day?

**Interviewee:** I didn't really go that often. It's mostly during the summer-ey months. Maybe once or twice a week.

**Interviewer:** Okay. What times of day usually?

**Interviewee:** Usually in the late afternoon like around three or four.

**Interviewer:** All right. Now we're going to talk a little bit about going back to the watch. Were there any instances in which the phone or watch surveys disrupted your sleep?

**Interviewee:** No, they didn't really.

**Interviewer:** Okay. Did you have any technical problems with the phone or the watch time app that you were able to solve by yourself without our assistance?

**Interviewee:** Some. There was a few times where the watch froze and I couldn't really get it to respond. Turning it on and off and restarting it worked out and I haven't had that

issue much at all. My phone having trouble connecting to the watch, sometimes it would say that it's not connected even though I am connected. That was also something that I had to restart my phone for and sometimes that worked. For the most part, everything was fine.

**Interviewer:** Okay. Yes. The classic turn-it-off-and-on-again strategy. Do you remember seeing any newsletters or birthday reminders?

**Interviewee:** Birthday reminders? Yes.

**Interviewer:** Okay. What did you think about them?

**Interviewee:** The birthday reminders were really good. I liked them. They really helped in planning out the next couple days, getting ready for the first period so I know not to do anything super disruptive.

**Interviewer:** Okay. Do you remember seeing anything about newsletters?

**Interviewee:** Newsletters? No, I haven't gotten those.

**Interviewer:** Okay, that's fine. How could we make participation in the study more fun or rewarding?

**Interviewee:** I'm not sure honestly, I'm sorry.

**Interviewer:** It's okay.

**Interviewee:** I think y'all did a really good job with keeping it pretty different every day just having a different method and stuff like that. That was nice. I liked doing that.

**Interviewer:** Overall, what would you suggest we do to help make the time study better?

**Interviewee:** I suppose given what you're trying to do, there's not a whole lot you could improve. I think this was a pretty good way of conducting it. Honestly, I didn't really have much problems with the study itself, apart from the way I was notified. Very loud, very buzzy.

**Interviewer:** Yes. Okay.

**Interviewee:** Apart from maybe turning that down a little bit. Yes.

**Interviewer:** All right. We'll keep that in mind. Then finally, what will you miss the most about this study?

**Interviewee:** I guess having something to remind myself to do small things like sometimes when the watch asked if I'm feeling hungry or not, I'm starting to realize, oh, I should probably take a break and eat something because I haven't eaten all day.

**Interviewer:** Yes.

**Interviewee:** I'm actually feeling hungry now, so it was a nice reminder for those sorts of things.

**Interviewer:** Okay.

**Interviewee:** I guess only that. Yes.

**Interviewer:** All right. Well, that's the end of that part of the exit interview. We have one more part--

**[00:21:06] [END OF AUDIO]**
