# Supplementary material for: Burden and Inattentive Responding in a 12-Month Intensive Longitudinal Study: Interview Study Among Young Adults
Source: JMIR Form Res. 2024 Aug 2;8:e52165. doi: 10.2196/52165 (PMC11329843; doi:10.2196/52165)
Supplement: Multimedia Appendix 1 [file formative_v8i1e52165_app1.zip › Transcripts/reliablydetaildebate_audio_6.13.22.m4a.docx]

**Interviewer:** Perfect. To start, can you provide me with some of your overall general feedback regarding the study?

**Interviewee:** I think the only feedback that stood out to me, I guess, was if I were doing a study or if I were doing one of the burst surveys and it gives me like, I don't know what, let's say five minutes to answer, if I saw the notification and then I started it at four minutes and 30 seconds and I managed to get maybe partway through the survey, once it hit that end window, it would just close out regardless of how long I had actually been in the survey itself. I guess that's the only real feedback I have. Otherwise, it was generally pretty fine.

**Interviewer:** Just maybe having the survey open longer or if you've started it, keep it open.

**Interviewee:** I think if you were currently working on the survey, then there's no need to close out of it until you know you're done. I guess leaving it open for longer would defeat the purpose, so I don't really know what the appropriate solution there is.

**Interviewer:** I'm going to ask more specific questions about your experience. First, I want to learn a little bit about your experience participating in the study in general. If a question is ever unclear, please feel free to ask me for clarification or you could interrupt me at any time. That's totally fine. First, how did you learn about the study? It's been a year, so **[unintelligible 00:02:00].**

**Interviewee:** It may have been through ResearchMatch, if that's something you guys use. Otherwise, I have a couple of different email newsletters that I get that are just like, "Hey, here's a study you might be interested in." I think it may have been ResearchMatch, but otherwise, they would've been just finding it on the Internet.

**Interviewer:** Do you remember what features about the study interest you or what motivated you to want to participate?

**Interviewee:** I mean, for me, it's mostly scientific curiosity. That's mostly why I do a lot of these studies, and of course, getting paid.

**Interviewer:** For sure. Can you describe to us what motivated you to continue answering studies throughout the survey? Sorry, about the study, not the survey.

**Interviewee:** After a while, it kind of just became part of the routine. It's not like I needed that much motivation. It just became something I did and definitely knowing that the more surveys I filled out, the more I would get paid. Definitely a motivation there.

**Interviewer:** Can you describe to me the process of answering phone surveys on a typical burst day?

**Interviewee:** On a typical burst day, depending on what time I said I would wake up, it seemed to be about at the 30-minute mark, like 8:30, 9:30, what have you, about an hour after I had set my wake time, and then maybe about every hour, give or take, like 10 minutes. It'd give me another buzz and I would open up my phone and answer whatever surveys.

**Interviewer:** Feel free to drink water. Yes, don't worry about that at all. How many phone surveys do you think you answered on a typical day? Did you have a goal that you tried to reach?

**Interviewee:** Yes, I definitely tried to get at least 12 just because for the past year-ish, two years basically ever since we started working from home, my work schedule has typically tried to be like wake up early and then get off work early. I'm usually up at 5:00, 5:30 and then I go to sleep at 10:30 to 11:00. That's a solid, what, I don't know, 17 hours, 16 hours.

I would try to at least get at least 12, at least 13 just because obviously, sometimes, I would be driving or commuting or sometimes, I would be biking, then I wouldn't be able to, but being awake for that long definitely gave me a bit bigger of a window.

**Interviewer:** Did you track your completion on the app?

**Interviewee:** Yes because it was really quick because swiping down from the top of the phone screen would tell you like, oh, how many you had and how many you missed. It was pretty easy just to check every so often.

**Interviewer:** What would have made participation in the study more fun or rewarding besides, of course, paying more money? That would be more rewarding.

**Interviewee:** I don't know if there's anything more rewarding than that, honestly. This would just be a secondary function. I guess depending on what the point of the study itself is, if it is focused on exercise tracking, then having any other metric would be cool, but if that's not really the point of the study, then there's really no need for it.

**Interviewer:** Getting some of that feedback would help. For this next section, we want to learn a little bit about situations of increased burden. We know participating in the study may not have been easy at times. We want to learn about some challenges that you may have experienced while in the study. What were some situations in which it was particularly challenging to answer a survey?

**Interviewee:** I would say because it was on weekends, there were times where I would be out on a bike ride or something. Even though I wasn't necessarily biking for six, seven hours straight, it would be like, okay, the three hours I was biking, then obviously those would be bunk. Then it would be like, oh well, after the bike ride, I'm hanging out with friends that I went biking with or we go to see a movie, stuff like that. There were some days where I went to drive up to NorCal or something and that's an eight-hour drive so obviously, on those days, you can't really do much.

That's pretty much it. I think by virtue of it being on weekends just made it clash a bit with regular, life but what can you do?

**Interviewer:** What part of the app was more disruptive for you? Was it getting prompted? Was it actually doing the surveys or the questions on the watch or the buzzing? I know the buzzing hits a lot.

**Interviewee:** I think it was doing the actual survey on the phone just because in those scenarios where I am with friends, but there would be times where I'd be like, okay, well, we're all hanging out, but it's no big deal. Then every hour, I would just be bringing out my phone, like, "Sorry guys. I just have some stuff to do real quick." Again, it only took a minute so no big deal, but I guess it's disruptive, is the word for it.

**Interviewer:** Let's see. Can you describe an instance where you preferred-- Pandemic dogs are-- That was fun. Can you describe an instance where you prefer dismissing a survey on your phone rather than answering it?

**Interviewee:** Yes. There's the movie example. If I'm in a movie theater, I'm not going to turn on my phone and make it really obvious. I guess, let's see, if I'm like out to dinner with people and then-- I mean, this one is obviously really recent and not real. This one was really recent and a very freak accident thing. I went up to NorCal to visit one of my friend's family members who was in the hospital and stuff like that. That would be not a great time to be answering surveys.

**Interviewer:** Of course. What did you typically tell friends or family when they asked you about the study? I'm sure they saw you answering surveys and whatnot. You know, the vibration.

**Interviewee:** My friends all know that I do a lot of these clinical trial things anyways. They took it in stride and they said, oh, it's just a clinical trial I'm doing. I'd have to answer these things on my phone and my watch. At the end of it, I get paid every month and I potentially get to keep the watch and stuff like that. They take it in stride because they know this is just something I do on a fairly regular basis.

**Interviewer:** Yes. Like, "Oh, another research study being done."

**Interviewee:** Yes.

**Interviewer:** Now, I want to learn a little bit about response accuracy. Besides not answering, I'm curious if there are any other ways that you dealt with some maybe challenges or burdens while you were answering surveys. How did you typically handle distractions while taking a survey?

**Interviewee:** I guess depending on the distraction itself, I would say that a lot of the time, if I were in a position to answer surveys, then I wouldn't really need-- It would be a time where I could devote my attention to it for the minute, minute-and-a-half it takes. For distractions, it was just no big deal. If there is a big enough distraction, then I wouldn't answer the survey.

**Interviewer:** Do you think there were situations in which your responses to a survey may have been less accurate? You're just kind of, "I'm just going to get through this just to get through this."

**Interviewee:** Yes. I'd say the only time that I could argue that was the case would probably be if I were like, I had just started driving where it was like, "Okay, I'm still pulling out of my driveway," or stuff like that. There were times where I would get overconfident and be like, "Oh yes, I'm biking, but I could probably still do this. It's just a straightaway. There's no one else."

**Interviewer:** That's impressive.

**Interviewee:** Yes. Like no one's around me and stuff like that. I am capable of biking without my hands, but it's still not something you really want to be doing.

**Interviewer:** [laughs] That's impressive. Do you think that your responses change depending on the time of day or location that you were in?

**Interviewee:** Absolutely.

**Interviewer:** Can you tell me a little bit about that?

**Interviewee:** Let's see. I guess on the days that I went into the office, I would wake up at 4:30 or 5:00 or whatever, and then my commute was about 40 minutes or so. Then the survey would typically come in-- If it were a Thursday or a Friday, the survey would typically come in around like maybe 5:40 or something, right when I pulled into the office parking lot. It'd be like, "Oh God, this is how I'm going to start my day, I guess," or after it's 9:45 PM or something and you've been doing this for 12, 13, 14 hours at this point, then it's like, "Oh, how frustrated do you feel right now?" It's like, "God, I feel pretty frustrated."

**Interviewer:** Yes, for sure. It's understandable. Let's see, a couple more questions here. How do you think your motivation or accuracy changed as you were in the study longer?

**Interviewee:** I think as time went on, it just became easier. Like the motivation to effort, that balance or ratio just definitely slid towards, "Oh yes, I may as well because I'm so used to doing this by now." Also, I guess there's a cost kind of thing where, "Oh, I've been into this for seven months already. There's no point in me not continuing this because I've done it for seven months already."

**Interviewer:** Last question here, what did you think about the questions and messages that were not related to measuring health behaviors, routines, or moods on the phone and the watch?

**Interviewee:** They were fine. [laughs] I'm trying to just think of a few from the watch.

**Interviewer:** If any stuck out or were memorable?

**Interviewee:** All of the memorable ones were just either like, "Oh, were you physically active or were you sad today or how happy do you feel?" I'm assuming they were the questions to make sure that you're still paying attention. Those were always fun, but otherwise, no opinion, really.

**Interviewer:** I'm going to change up the questions here a little bit. Do you know anyone else who participated in this study?

**Interviewee:** No, I do not.

**Interviewer:** Okay, we can skip some questions. Exercise, I know you talked about cycling. In the past month, have you exercised or performed any type of physical activity such as going for a walk?

**Interviewee:** Yes.

**Interviewer:** Okay. What types of exercise do you typically do then?

**Interviewee:** Typically cycling.

**Interviewer:** Are there certain days of the week that you're cycling?

**Interviewee:** Typically, weekends just because that's for my free days.

**Interviewer:** Are you outside cycling? I'm just assuming that like a--

**Interviewee:** Yes.

**Interviewer:** -then are there certain times of the day that you're cycling?

**Interviewee:** I try to do not super early mornings, but morning into afternoon, just to avoid noon time.

**Interviewer:** Heat.

**Interviewee:** Sunlight.

**Interviewer:** I cycle too. Where do you typically cycle? You're in California, right? You're in Southern California.

**Interviewee:** Yes. I'm over in Anaheim, so typically, I go down to Newport-ish. One of my friends lives--

**Interviewer:** Do you go down the-- What is that, along Wash right there?

**Interviewee:** -I've only done that once. Typically, all of our friends, we just meet up at my friend's place, and then we just bike along the coast. That'll be Newport or we have another friend in Long Beach. We'll all drive to Long Beach and bike along down the--

**Interviewer:** The river there.

**Interviewee:** -or San Gabriel River, down to the beach and we'll bike up the coast or we'll do like Santa Monica and just bike up and down the strand. We did San Clemente a while back and then we drove down to San Diego for a weekend and just rode around down there too. Typically, it'll be like preferably somewhere along the beach.

**Interviewer:** You're so close to the beach, I'm jealous. You got some nice rides there.

**Interviewee:** Are you straight up next to USC then or-

**Interviewer:** Pasadena. I have a lot of the foothills to ride in, but there's of a lot of hills.

**Interviewee:** I used to love the LA River too. You could start at the Rose Bowl, but I guess there are some parts of the LA River that get sketchy.

**Interviewer:** I know. That's the problem. I know you can ride from, what is it called? Not Dougherty, kind of Azusa, Irwindale area. You can ride along Wash that way all the way to-- I think it goes all the way to like--

**Interviewee:** It goes all the way to Long Beach.

**Interviewer:** -Seal Beach. It goes to Long Beach. I heard sketchy in some parts too.

**Interviewee:** Yes, definitely sketchy in some parts and I guess--

**Interviewer:** Hot.

**Interviewee:** -yes, exactly. If you start inland and then you're ending inland, it's just not a good time.

**Interviewer:** Just straight concrete the whole time. [laughs] I could talk about cycling forever. I should stop. Were there any instances where the phone or watch service disrupted your sleep?

**Interviewee:** No.

**Interviewer:** Okay, cool. That's good. Let's see. You answered that one. Do you remember seeing the newsletters or birthday emails?

**Interviewee:** Every so often, I feel like I got them maybe quarterly-ish. Yes, just a handful.

**Interviewer:** What did you think of those newsletters?

**Interviewee:** They're cute. It was nice to see that it was like, "Oh yes, this is how many people are in the study." It's like, "Oh yes, there are a bunch of other people other than me doing this." Then seeing all of those stats throughout the time was pretty cool.

**Interviewer:** Let's see, final question here. Are there any additional points that we didn't cover or things that you'd like to discuss that we didn't bring up?

**Interviewee:** No. I think everything's good.

**Interviewer:** Okay, cool. Thank you for answering all those questions. I know that was a lot, so thank you.
