# Supplementary material for: Burden and Inattentive Responding in a 12-Month Intensive Longitudinal Study: Interview Study Among Young Adults
Source: JMIR Form Res. 2024 Aug 2;8:e52165. doi: 10.2196/52165 (PMC11329843; doi:10.2196/52165)
Supplement: Multimedia Appendix 1 [file formative_v8i1e52165_app1.zip › Transcripts/nucleusbackwatercanning_audio_6.22.22.m4a.docx]

**Interviewer:** To start, can you provide me with some of your overall feedback regarding the study?

**Participant:** I think overall, the first couple of months might have been a little harder to just get used to the routine. You couldn't really get a sense of when you were going to get some of those, watch check-ins and the burst periods and how the cycle worked, if it was reset every 24 hours. I think it took a couple months for me to get used to it but after that it really just came naturally to me.

The device itself was really great that the battery lasted really long and I only had to take 30 minutes to an hour every day and it goes from 0 to a 100 really quickly like I said, in an hour most. I definitely like the vibration in the watch.

It's very evident that there was a prompt or something and it was really easy. It was not a big question that you had to read. It was just two words. Are you feeling tense or stressed or whatever and then there was just no option. That was all really good, overall, honestly. Maybe some things, I think it's because I did not use the Do Not Disturb more than I probably should have.

I do remember learning that once you turn that on, maybe the watch prompts will go away or something. I feel there were a couple times where I did and I was still getting the watch prompts, so I don't know if I didn't do it correctly or if the watch prompts wouldn't just start coming yet. It would be nice if I could even set a schedule or something like specific days of the week. These specific hours just don't give me any watch prompts or the phone surveys.

I know that I will miss those, but I am okay to miss those if there are other priorities, obviously. Sometimes, it was just a little hard getting it under control like you just felt it was sometimes just bombarding you with prompts and surveys. There was no real good way to just turn it off. The Do Not Disturb, like I said, I think it did work majority of the times. I'm just saying there were a few times where it was not really effective in all the surveys and the prompts just overrode the Do Not Disturb and was just still continuing to--

**Interviewer:** Is this on the watch?

**Participant:** This is both on the watch and the phone. The phone surveys during the burst periods and on the watch. I don't know, again, if I did not set the Do Not Disturb properly or what, but anyway that was one thing. Notifications, I did have some issues with that especially during the burst periods. I did a lot of troubleshooting and everything with the team that I just did not feel any vibration or buzz or not even in the notification panel that you have a survey available or something, but then you have count of it, right?

Prompted these many times and then answered or unanswered these many times. I was just seeing the number going up and up and up, like one burst day that it prompted me 14 or 15 times it says, but I did not receive more than four or five prompts. There were some definitely issue with the notifications. It counted it as prompts but I didn't really see any notifications like a vibration or anything in the phone. This was for the burst period during the burst days. Some of the notifications just didn't--

**Interviewer:** Did that ever get resolved or was that throughout?

**Participant:** Yes, it did. They had to do a lot of troubleshooting. I made sure all of my notifications were turned on and nothing on my phone that it was preventing the notifications from. I don't think they did resolve it, but they did find out it was something on their end that was not pushing the notifications correctly. I had to just uninstall and reinstall, look back into the time study app and do more setting changes but it did get resolved.

There was a couple of burst days, I was like, I only got five, how am I supposed to complete 11 or more if I only got five? But then I look at the the numbers and it's, "Oh, the app still prompted me 15 times, but I only received five of those prompt notifications."

Other than those minor technology stuff, one major thing I had was the data not transferring and I don't know it was after a good eight, nine months into the study, so this was only a couple months back and I had to work out a lot with the team through back and forth messages that they were just like, "Hey, we're not receiving any of your data from your phone or your watch, so we don't know what happened."

Obviously, they asked me to install an app and go to the root folder of the time study app and just whether all of the data was still there. Then they gave me step-by-step instructions on how do I get it to them and how do I manually push the data **[inaudible 00:05:41]**.

That took a bit of resolving over two weeks even, two to three weeks and every-- Plenty of things and finally I think it worked out and they were able to obviously get hold of all the data. That was just so weird, if it had happened at first, then it makes sense but it happened eight months in when--

**Interviewer:** Nothing changed like Wi-Fi wise, nothing changed?

**Participant:** No.

**Interviewer:** That's interesting.

**Participant:** Yes, it was all the same settings in the phone, all the same Wi-Fi connections, same setup as until there was no major or minor changes at all with the phone itself, the device and the settings of the device. Just happened pretty randomly, so sorry I've been going on for a long time.

**Interviewer:** No, this is all good feedback. Don't be sorry.

**Participant:** Yes, those are all the minor and the major, I guess challenges that I mostly faced with the notifications and everything. Battery, I think was good. That was a good experience actually that I was at first concerned that the notifications of the survey, so the app running in the background almost always drain battery, but it actually did not. I didn't really have good intervention or anything that, "Oh, this app is just killing my phone with battery or my watch," but thankfully, no, it did not happen on devices.

The battery was definitely a plus. I didn't feel any of the notifications honestly to be that annoying or something unless I specified, "Don't give me any surveys or prompts during this time but then when it over **[inaudible 00:07:25]** that and still gave it to me. That's the only thing only time it was frustrating, but the other times where it was just normally coming through the phone or the watch, it was okay. It was not bugging me or anything.

**Interviewer:** Good.

**Participant:** Yes.

**Interviewer:** Okay thank you for all the feedback. Not too much at all anything is valuable, so I'm glad at least a lot of the technical issues got worked out. I know the data was a big one, **[unintelligible 00:07:55]**.

**Participant:** Yes, because that kind of is the desired outcome for all of this, so we go to waste.

**Interviewer:** We want that. For these first couple of questions, I want to learn a little bit about your experience in the study, motivation, all of those things, just in general. First question I have is how did you learn about this study?

**Participant:** I believe it was through ResearchMatch, I believe the organization. I filled out an initial screener saying I was interested in the study and then I got an email and that's how I found out about it. It was not through a brochure or word of mouth or anything. It was ResearchMatch, if I remember correctly.

**Interviewer:** Okay, do you remember what features about the study interested you or made you want to participate?

**Participant:** I think it aligned with some of the goals that I had for myself, over a year ago when I was starting the study was to obviously just around fitness and sleeping well and eating well and all of that in this study. I was just starting to feel, "Maybe this study will help me feel more accountable even for some of the goals that I was setting for myself and it will help me understand what I am going through from a physical health perspective or a mental health perspective on a day-to-day basis."

All of that, I was wanting to do one way or the other. After I looked at the study, it was just struck to me that maybe this is the avenue through which I can get to my goals and help me achieve my goals. There was a lot of alignment and sync with what I was trying to do and what the study was wanting me to do, which was more of the same thing. Obviously, the study also means you have more accountability, more commitment. They're also providing me with the right tools and resources to go through this journey. That's how I got interested into it.

**Interviewer:** Can you describe to me what motivated you to continue to answer surveys and questions in the study.

**Participant:** I was learning a lot more about myself. I think that's the biggest thing. If not for the study, people do it through journals or writing daily journals or writing something on their phones or notebooks. I don't have a habit of that. Doing these surveys, it definitely helps me understand different patterns and friends on why I was behaving like something certain times of the day or not

Those daily surveys or the watch check-ins, it just allowed me to quickly take a step back and reflect on what's actually **[inaudible 00:10:57]** me to react or not react certain in ways. It was just a really good self-reflection exercise to be honest. Like I said, I understood better about my own characteristics and personality and being able to work on those. I think I learned a lot about myself and why I-- The things the way I was doing, and the study had definitely helped me understand that more better.

**Interviewer:** That's good. That's a good outcome. Can you describe to me the process of answering the phone surveys on a typical burst day? From start to finish?

**Participant:** I think on the burst days, it's a bit too frequent. I don't know. It's not like it follows a very rigid schedule. Average I get like one an hour, but there have been like three, four hours where I've not got anything. Then there have been times where I got four surveys or something within the same hour. I don't know **[inaudible 00:12:08]** nice if there was somewhat of a more predictable schedule, knowing that, "Okay, I know I'm going to get the next survey."

If there was like a countdown or something, but maybe that's not the point of the study. You really maybe wanted to be really just random. On normal days that randomness worked out pretty well. But on burst days you are answering like 12 or 15 surveys a day for like three or four days continuously. It would've been nice like, I took the first survey and then there was a countdown or something that tells you, "Okay, you're going to receive the next burst survey in 78 minutes from now or whatever."

And then there's a countdown. I've missed some of those because I didn't really know when the next one was going to show up and then I was doing something and then it just skips my mind, right? I would've been more aware of it. I don't know if there was some way to know in the back of my head, "This is when I'm going to receive my next survey."

Even if it's not down to the minute level detail, it could have been like a range. Like it's going to be between 8:00 to 8:20, is when you're going to receive your next burst survey or something. Even that would've helped rather than just-- With the burst surveys, you have to have your phone in your hand almost all the time. If it's in vibrate and silent, if my phone's on the kitchen counter or a table or anything, even if it vibrates, then I'm not going to know.

With the watch, it's a different story because you're wearing it really almost all the time. If the phone's not in your hand, you're most likely to miss the burst surveys. That's why I was suggesting if there was a range and say, "I took this burst survey I guess the next one's going to be 8:10 or 8:20, whatever," then I can stay away from my phone for that long and then pick up my phone when I know the survey's going to actually come.

**Interviewer:** That makes sense. Did you have a typical goal that you tried to answer each burst day?

**Participant:** Like how many surveys?

**Interviewer:** Yes.

**Participant:** Yes, the typical goal was, what was needed, minimum to qualify for the incentive. I think they said it was 11 or more. That was my typical goal. Honestly, it was just from the time I wake up to the time I sleep, even if it's not 11 or whatever, how many ever comes. Typically, I was just thinking it was a good practice based on how many surverys I was getting. It was about 13 or 15 usually. I was under the impression that, even if I missed one or two, maybe that's okay. My goal was to answer everything but one or two that I obviously have to miss for unavoidable, certain **[inaudible 00:15:08]** like that.

**Interviewer:** For these next questions, obviously we know that at times being in the time study wasn't easy. We want to learn more about the challenges that you experienced throughout the year. What were some situations in which it was particularly challenging to answer the surveys, either on the phone or the watch?

**Participant:** Working. When you're working obviously. When you're in the middle of a meeting or when you're in the middle of some task where you're focusing, this could definitely be a quick distraction, right? **[inaudible 00:15:44]** whatever you're doing. That part was challenging obviously because this is like throughout the day, really 24/7, 365 type of thing. I think that part was challenging.

Obviously, when you're working or doing something that requires a lot more focus and this surveys is a distraction or something. The other challenges were really just during special occasions or when you're on vacation or you're doing a trip or you're in the car driving. During the holidays, when your burst period falls within, I don't know, a holiday or something, it was obviously really hard because you don't have your phone in your hand all the time when you're on a holiday.

You're doing something and it doesn't really occur to you that you are supposed to be filling those out. I don't know, stability around being able to set schedules, just minimally at least, I'm going to be on vacation these four days or something and the surveys or the frequency of the surveys adjust a little bit to that, would've been a little bit more comfortable.

**Interviewer:** Can you describe to me an instance where you prefer dismissing a survey on the phone instead of answering it?

**Participant:** When I see it and I don't answer it?

**Interviewer:** Yes.

**Participant:** That's rarely happened to me. If I really just cannot spend a couple of minutes answering that survey because I was in the middle of another phone call or something, talking to someone, or I'm driving and I see there's a survey, but I can't really just pull the car over and take that survey. I'm just going to continue driving. Driving or being on the phone with someone, or doing any other focused activity, I guess that I just cannot override to take the survey.

**Interviewer:** What did you typically tell friends or family when they asked you about the study?

**Participant:** My wife has asked me a lot about this.

**Interviewer:** [chuckles] What are you doing?

**Participant:** What are you doing? But no, I honestly, the way I've explained it is that it's just something that I would've otherwise done, one way or the other. It keeps track of my everyday activities or emotions or what am I eating? How is my physical activity or sleep quality? One way or the other, I would've done this and doing this through a study, it's a win-win.

They are learning a lot from what I'm doing, and with that research, it's going to help a lot of people and it personally is helping me too. Obviously, to be enrolled in the study, it's keeping me more accountable. If I'm just answering every day that I am not having any physical activity, I feel guilty. I'm like, "Oh, okay, I actually should probably engage in some physical activity."

It's that commitment and that accountability. I think it was just like I explained, it's just a really holistic, all inclusive wellness program in which you just learn a lot about yourself, you reflect on it and then you adjust your behaviors, or eating, or activity, or sleep, based on those reflections. We all do that subconsciously, or consciously, or more rigorously, or more casually, so I was just like, "This is just my way of doing it. It's just works for me. It helps to study people too, so might as well." [chuckles]

**Interviewer:** You're going to have to find some different app now or something.

[chuckles]

Something similar to the time study, Time 2.0. [chuckles]

**Participant:** Yes. Honestly, that would be super helpful.

**Interviewer:** For these last questions here, I want to learn a little bit about response accuracy. Besides not answering a survey, we're curious if there were any other ways that you dealt with some challenges or hurdles while you were actually taking the surveys. How did you typically handle distractions when taking a survey?

**Participant:** I would sometimes pause the survey midway. As in, I would have-- That's the challenge. I would have really appreciated it if there was actually a pause button or something, and then I continue to take the survey because I could have started the survey and answered the first four questions, and then there's something important like I'm getting a call or my wife wants to know something, then I want to pause and answer and take care of that and come back to the survey, but before you could do that the survey is already gone.

It's super **[inaudible 00:20:51]**, I believe. Halfway through the survey, it could still just go away if you're distracted for 30 seconds or more sometimes. That's been really challenging. It would be really nice when you get the survey itself, you could just have a button like an alarm clock, snooze for five minutes or something, so you delay the survey for five minutes.

I absolutely cannot take it right now, but I can take it in five minutes. I don't want to miss the survey, so can you pop that in a notification for me in five minutes? That's one thing. The other thing, it would be helpful to not be distracted when I'm taking the survey because I don't **[inaudible 00:21:35]** the survey mindlessly when I'm doing other things. It's to build that feature in to focus while you're taking the survey.

If you really have a distraction that you know of, at least allow me to indicate that like, "Hey, I am engaging in something else, and I want to pause the survey and I'll come back in a couple of minutes or whatever." Otherwise, you're just like, "I know the survey is going to go away within 30 seconds or 40 seconds, so even if I'm distracted, whatever, fine. I'll just answer the survey to whatever I can focus on it."

That's not good for me, that's not good for you. It would be a lot better if it was a little bit more accommodating, honestly. The accuracy, like you said, of the responses would be much better.

**Interviewer:** As far as being more accurate, were there situations in which your survey responses would have been less accurate? Thinking like, "I'm just going to answer these and get it over with." Didn't think much about them, and I just answer them.

**Participant:** Not really, honestly. It's just some of those exposed scenarios, I guess, when you're out. For me personally, at least, you're out at a dinner or there's people around you or something, you cannot go by yourself or take your phone and focus on the survey. You're just passively filling the survey out while you're doing other things.

If I can manage to find time like I said during the everyday course or things like that, I can take a quick two minutes to do that, but when I'm outside or on vacation or with friends or with people around, then those are the times I would say the responses would probably be least accurate.

I'm not even in the frame of mind to think through these questions given my current situation. I'm just going to, like you said, "Go ahead and get done with it," than actually think about it. Yes, for me, it's some of those exposed scenarios that that's like not the normal routine, anything that's out of the normal routine, then the accuracy could be less than what it would have been for normal.

**Interviewer:** Let's see. Good. This is the last question then within the last section of this interview. This is not about accuracy or anything, it's kind of an offshoot question. What did you think about the questions and messages that were not related to measuring either health behaviors, routines, or mood that came up on your phone and the watch? If you remember them.

**Participant:** I'm trying to remember. Some of those could have been simplified. I think it was mostly the multiple-choice questions that I found to be not very relevant. The question where you only choose one option seemed a little bit more relevant to whether it was asking about your sleep or health and all of that, but sometimes, I don't know, about the social activities I don't know. I think it had some to do with COVID too, if you've experienced [crosstalk] viral symptoms or not.

Asking something like that every day, it's just really not useful. I think most of the questions that I found to be irrelevant were the ones where you could select multiple answers, and those were about COVID or avoiding or going to social gatherings and things like that. Those really didn't really matter to me that much. Those were not health or wellness or sleep focused.

Some of those could have been avoided or could have been, I don't know, a one-time question a week or something rather than having it as part of the daily survey. Those things are not really changing on a day-to-day basis, so there's no point in me answering those same questions every day.

**Interviewer:** How about any-- Did you notice any of the questions that seemed to be random questions?

**Participant:** I don't think so. They were maybe, I don't know, about walking a dog or something. I don't know how relevant that was. One way or the other, it all made sense to me. There wasn't something like, "Why are you even asking me this, and how does this contribute to the study in any way, shape, or form?" I didn't really feel anything that absurd or random.

Most of them like I said were, holistically, it made sense why I was being asked some of these questions. It aligns with the objectives of the study in one way or the other. There wasn't anything that stood out to me that was just super random.

**Interviewer:** I think that was it. Are there any other points or anything that you wanted to tell me that we didn't discuss that you'd like to discuss?

**Participant:** Not really, no. I think the first when you were talking about the overall experience, it all just came out of my mouth. [chuckles] The first 10 minutes where I was just-

**Interviewer: [unintelligible 00:27:18]**.

**Participant:** -talking non-stop. No, those were all the thoughts that I had gathered about this study. I just blurted them all out in the beginning. No, there's really nothing else that we haven't already talked about.

**Interviewer:** Okay. Well, thank you. That was great feedback. That was valuable feedback.

**Participant:** Sure.

**Interviewer:** I appreciate that.

**[00:27:44] [END OF AUDIO]**
