# Supplementary material for: Burden and Inattentive Responding in a 12-Month Intensive Longitudinal Study: Interview Study Among Young Adults
Source: JMIR Form Res. 2024 Aug 2;8:e52165. doi: 10.2196/52165 (PMC11329843; doi:10.2196/52165)
Supplement: Multimedia Appendix 1 [file formative_v8i1e52165_app1.zip › Transcripts/espionagechihuahuagraffiti_audio_7.25.22.m4a.docx]

**Interviewer:** Okay, to start, can you provide me with some of your overall general feedback about the study?

**Interviewee:** Honestly, the thing that I liked the least was the vibration, the fact that I could not change the vibration mode and that was mostly because it was so strong that I would have otherwise not silenced, for example, my phone or my watch in certain scenarios, but I knew the vibration was so disruptive that I would just silence it. There were questions or prompts or whatever, that I could have responded to that I didn't just because I have to silence or put it to Do Not Disturb either on my phone or the watch, depending on the day.

**Interviewer:** Both vibrations were pretty bad you're saying, both phone and watch?

**Interviewee:** Yes. They were way more disruptive than any other vibration. I understand why the one in the phone needs to be like that because you might not have it nearby or something like that but the one in the watch it's should be on your wrist. It was just I don't know I found that a little disruptive.

**Interviewer:** For sure.

**Interviewee:** There were some questions that, to be honest, I didn't know the answer to. Some of the prompts that you have to just validate that I am not randomly selecting an answer. There were some that I just didn't know the answer to. I think it's probably because I'm international so all the ones that were America-centered of like, "Which of these was a precedent?"

[laughter]

**Interviewer:** You're like, "I don't even know."

**Interviewee:** There were some that even if they were America-centered, the answer was pretty obvious but there are a couple that I had to ask a friend or something because I didn't know what to--

**Interviewer:** I'm sorry. That's terrible, that should have been easier.

**Interviewee:** Especially if it's for you to validate that I'm paying attention to the question. What else? I think in the first days-- Something that I didn't like about that I think it was more than that I didn't like it, I felt that it was not explained to me correctly during the orientation, or however you want to call it, which is that you could not really change the dates of the first period.

You could delay it by two days but that was pretty much it. What I was told during orientation is that I was going to be able to choose which four days, or which eight days, within a month, I could do the first period. Again, more than the fact that I could not do it was the miscommunication.

**Interviewer:** The oversight on our part.

**Interviewee:** Again, I just feel that sometimes it would have been more convenient for me to choose different dates based on schedules.

**Interviewer:** Schedule.

**Interviewee:** What else? I think some of the questions the answers grammatically did not answer the question. I don't know if that makes sense. In the first-period questions, the options didn't make sense completely-

**Interviewer:** To the question.

**Interviewee:** -to the question and sometimes it was hard to know what-- My definition of extremely or whatever might be different from someone else's. Sometimes, I wonder I don't know, it might have been helpful to have even if it's just during orientation, just guidance of what it means to be completely in control. There were some questions that I was like, "Yes? No? I don't know." It was hard to know how to differentiate the levels and if that was an agreement, it's what you were expecting.

**Interviewer:** That's a good point. That's something that would be good to know going into all the surveys that you have to answer. Thanks for letting us know about that.

**Interviewee:** Honestly, I think that was pretty much it. It was a bit annoying that the battery was draining so much just because it's hard to because you should be wearing it for almost 24 hours. It's hard to find time to charge it. Things that I did like, I like that the phone reminded me to remove the Do Not Disturb mode from the flash, because sometimes I would put it on and then forget to remove it. I did appreciate the reminder of that. I don't know what else.

**Interviewer:** If something comes up later, you can always-- I might jog your memory with some of the questions I ask. If something comes up, please feel free to let me know. Let's see. For this first section, I want to learn a little bit about your experience participating in this study overall, and your motivation behind it. The first question I have is how did you learn about the study? Where did you first learn about it?

**Interviewee:** I think it was through Research Match, I think is the name of the platform. In Research Match. I just got an email saying if I had certain characteristics that I could participate.

**Interviewer:** Do you remember what features of the study interested you to want to join?

**Interviewee:** I think it was because it had to do with sleep and exercise. Honestly, I don't remember particularly what was the thing. It just sounded fun, I guess.

**Interviewer:** Can you describe to us what motivated you to continue to answer surveys throughout the study?

**Interviewee:** I don't know I felt that with time it felt, "Argh, I'm already doing it."

**Interviewer:** Might as well.

**Interviewee:** The more I progressed in the study, it was like, I don't know, I was less likely to quit and just keep going. In part, I'm a PhD student so I know how hard it is to do research and so far it was just shared suffering, I guess.

**Interviewer:** We appreciate that.

**Interviewee:** The compensation, definitely, whatever you were like, "I don't want to keep doing more." Especially adverse periods, I think were the hardest sometimes, because it felt like the same thing over and over and over. Some days between two first periods, nothing had changed so it was like--

**Interviewer:** Same answers.

**Interviewee:** I guess between the compensation and just I don't know, feeling bad for the researcher.

[laughter

**Interviewer:** Sharing in the agony of research. Can you describe the process? I know you've mentioned the first period being tough. Can you describe the typical process of answering surveys during the first period?

**Interviewee:** I would just stop whatever I was doing and read through the questions. Usually, by the third questionnaire of the first day, you know the order of them, so you go through them faster except the ones where there's a change that you have to stop and remember what were the options. I would just stop what I was doing. Sometimes if I was, for example, driving, but my partner was with me in the car or something, I would just ask them to read out loud the questions and just select for me.

I think the only time that I didn't answer-- I don't think I missed a day of first period, but I might have throughout the study, but the times that I didn't answer a questionnaire was either because I was driving and I couldn't look at my phone, or I shouldn't look at my phone or because I was in a meeting, or teaching or something and I just had to ignore.

**Interviewer:** Did you track your completion or have a goal number of surveys that you try to answer each time?

**Interviewee:** At the beginning, I aimed for the threshold to get the extra compensation. To be honest, towards the end, I just aimed for eight. Whenever I was done with eight, I was like, "Okay, I'm just going to ignore the rest."

**Interviewer:** The rest of the day.

**Interviewee:** Yes. I made sure that at least I was doing eight, but towards the end, I was no longer aiming to go big on, I think it was 11 or whatever.

**Interviewer:** 11, yes. What would have made, I guess participation in this study because obviously, it's a year-long, that's a long time. Is there anything that would've made it more fun or rewarding besides paying more money that would be more rewarding, but to help make it fun throughout the year?

**Interviewee:** I don't know what the solution would be, but I think the first periods were the hardest part of it, to be honest. Oh, I have another thing. Sometimes the watch would ask me like, "Were you exercising 128 minutes ago?" I'm like, "Wait a second, I have to make the math." Then the question would disappear.

**Interviewer:** You're like, "I can't. How long ago was it?"

**Interviewee:** Maybe ask me about 5 minutes ago or even 10 minutes ago. Sometimes it would come up with these random numbers.I don't know if it's a random generator or what, but those were hard to answer too. I don't remember.

**Interviewer:** For sure. That's understandable.

**Interviewee:** I think maybe having access to some of the statistics would have made it fun. If at the end of the day or in the morning you could see I don't know your heart rate or if you exercised or not, or any insights on your sleep, how many times you woke-- Any info that you could give back as feedback so it feels like you're not doing it just for someone else to look at the data, but you're also receiving something in return.

I'm trying to think of what else, but honestly, I don't know. The surveys on Sundays were long and there were some that were repetitive. Especially the COVID one. I didn't feel they really-- I don't think things changed that much during the one-year period for those. What else? I can't think of what things could motivate me to do it more. Honestly, changing the notification, that would have made a huge thing for me.

**Interviewer:** The vibration?

**Interviewee:** Hitting the-- Yes. Both on the phone or in the watch.

**Interviewer:** It depends.

**Interviewee:** Not even in meetings or stuff like that, but if you're with friends or stuff or colleagues or something, it's just really disruptive and you don't want your watch to be vibrating every, I don't know, 15 minutes.

**Interviewer:** For sure. It's a lot. You were talking about statistics. We're finishing up data collection at the end of August, and once we're done with data collection, we're trying to come up with a way, obviously, there's a lot of data, but some visualization of some type of data that we can give you guys. Hopefully, we'll be sharing that after August. I should just say. That way we can kind of-- [crosstalk]

**Interviewee:** Oh, I think I really enjoyed, I think it was Thanksgiving that you guys sent the updates.

**Interviewer:** Oh, the newsletters?

**Interviewee:** Yes. I don't know how hard those were to make, but if you could do them more often, it was just fun. Especially not the super professional stuff, but half of you like to eat pizza and Fridays or whatever. Those little things were really fun.

**Interviewer:** Made it more interesting. I get it. What time? I'm going to switch rooms real quick if you don't mind. It'll be like a three-minute transfer.

**Interviewee:** No problem.

**Interviewer:** I'm just going to bring my laptop in.

**Interviewee:** Okay.

**[pause 00:15:18]**

**Interviewer:** Sorry, this is like a super awkward small group deal sort of thing. The joys of working in the office. Thank you. Sorry about that. For this next section of questions, I want to learn a little bit more about situations of increased burden. Obviously, you were saying when you're driving or when you're teaching, it was a little bit more challenging to answer some of the surveys. I want to learn a little bit more about those challenges that you faced.

Besides those situations that you already told me, were there any other situations in which it was particularly challenging to answer the surveys?

**Interviewee:** I guess holidays like Christmas, New Year's, stuff like that, that you're with family and you don't want to be on your phone all the time.

**Interviewer:** That's a good point.

**Interviewee:** In my case as well, conferences. If I'm in a conference that's four days where I should have my phone off almost all the time. Those were a little bit challenging.

**Interviewer:** Those are good points. What most frequently led you to be unable to or to miss answering surveys?

**Interviewee:** Honestly, meetings, most probably the fact that you could not choose the days made it that sometimes, it just landed on a day where I just had a lot of meetings

**Interviewer:** Can't do anything about that.

[laughter]

Were there any instances where you preferred to just dismiss a survey? Like you saw it come up and you were like, "I can't answer this," and just dismiss it instead?

**Interviewee:** I guess when I was doing something else that was important. If it wasn't a social situation and it was during the first period, I knew it was not a short thing. It would require me to be on my phone for a period of time and if I was in the middle of a conversation, it would just dismiss it, or if I was in the middle of exercising and I knew the survey was there, but I'm not going to stop just to answer.

**Interviewer:** Like, "I'm in the middle of a plank right now. I'm not trying to answer."

**Interviewee:** Yes. Or, "I'm running right now."

**Interviewer:** Too hard.

**Interviewee:** It was mostly that and driving and exercising, if I was biking or something like that, the same thing. Mostly, social situations that require my attention and that both with friends and family, work-related stuff, or exercising.

**Interviewer:** What did you-- [crosstallk]

**Interviewee:** A couple of times I missed them because I was asleep.

**Interviewer:** Just like if you are taking a nap or if your sleep time was set earlier and you slept in?

**Interviewee:** Yes, exactly. More of the second.

**Interviewer:** You're like, "I'm tired, I'm sleeping today." Did they ever wake you up? Either the phone or the watch?

**Interviewee:** No. I set both the phone and my watch in Do Not Disturb at night. The surveys would come in, but I would not fill them because they wouldn't go through.

**Interviewer:** That's good. They at least didn't wake you up. What did you typically tell family or friends when they asked you about this study? Did you have a go-to response?

**Interviewee:** I'd just say that it was part of a research study. I think after a while everyone just knew what it was for.

**Interviewer:** Like, "Oh, she's going to answer the survey again."

**Interviewee:** Yes. I explained the whole thing to a couple of people that shared the most amount of time with me, the whole thing of what it was, but most people I just said like, "Oh, this is just part of a research study." That's it.

**Interviewer:** For this next section, I want to learn a little bit about response accuracy. Besides not answering a survey, I'm curious if there are any other ways that you dealt with challenges or distractions while taking a survey. The first question is, how did you handle distractions when taking a survey? I know you mentioned with driving you would have your partner would ask the questions for you, but I guess if you were working and whatnot?

**Interviewee:** If I was answering the surveys because I could stop whatever I was doing to answer the survey. Whenever I was answering the survey, it was because I could, otherwise I would just not answer it. I would just stop whatever I was doing and just answered the survey, and be done with it. I don't think I had distractions during answering the surveys.

**Interviewer:** Would there have been situations where your responses would've been maybe less accurate? Like you just quickly answered through or maybe if your responses changed depending on the person you were around or the time of day?

**Interviewee:** No. I think I tried to be accurate or as accurate as possible and I would go back if I made a mistake or something. I think the ones that were hard to answer-- The location survey for some reason, most of the time, it would pop up when I was doing groceries, but there's no that option. There's like shopping, I think it was the only related to a store, I was like, "Is this shopping or is this other because I wouldn't consider it groceries as shopping, but it's technically in the store."

[laughter]

**Interviewer:** I guess it's fun shopping.

**Interviewee:** Yes. Those maybe are not super accurate. I also moved during the study within the same city but moved houses and I think the app probably got super lost of why home was no longer--

**Interviewer:** Where are you?

**Interviewee:** No, I think I tried to be accurate. There are some things that were hard to judge, which it's more-- Yes, because the questions were really subjective and I didn't know sometimes how to answer. Some didn't even make sense to whatever I was doing. It was hard to understand the difference-- The yes or no-

**Interviewer:** Scale

**Interviewee:** -of levels are easy, but the scale, the intermediates was hard to judge what meant quite a bit versus extremely, or totally whatever it was.

**Interviewer:** Last question for here. How do you think your motivation or accuracy changed as you were in the study longer?

**Interviewee:** I wouldn't say the accuracy changed. The motivation, I think mostly what I said at the beginning, I was aiming to do 11 or more in the first periods and towards the end, I was just aiming for the minimum which was eight. I think towards the end I was more likely to make the decision of going into Do Not Disturb in my watch than towards the beginning. At the beginning, I would only do it in super important situations and towards the end, it was more anything that I thought it was going to be a distraction I would just silence the watch.

**Interviewer:** Are there any other points that we didn't discuss or cover that you'd like to discuss, maybe something that you want to bring up?

**Interviewee:** No, I think that's pretty much it. Oh, I guess sometimes I think there was a problem with my watch or my phone not sending the data to you guys at some point.

**Interviewer:** Oh, that's right. Yes. I remember. I think we got all of it though.

**Interviewee:** I think eventually you did, but it was hard to know-

**Interviewer:** When it was coming in.

**Interviewee:** -if it was going in or not. Maybe a confirmation of something like, "We got your results or we got X thing," just to know because it kind of feels while I'm doing my part of it, but if you don't get it, then how are you been able to know I'm doing it.

**Interviewer:** What am I doing? Yes, for sure.

**Interviewee:** Maybe getting a message or something. I don't know how often you send data from the app to the server, but if whenever that happens you just get a notification in your phone saying, "Your data was received," you know like, "Oh, it's working."

**Interviewer:** Yes, right, but not this backup of data. Yes. Okay. Thank you for answering all of those questions.

**[00:26:48] [END OF AUDIO]**
