# Supplementary material for: Burden and Inattentive Responding in a 12-Month Intensive Longitudinal Study: Interview Study Among Young Adults
Source: JMIR Form Res. 2024 Aug 2;8:e52165. doi: 10.2196/52165 (PMC11329843; doi:10.2196/52165)
Supplement: Multimedia Appendix 1 [file formative_v8i1e52165_app1.zip › Transcripts/freightbrisklypopulace_audio_6.10.22.m4a.docx]

**Interviewee:** Okay.

**Interviewer:** To start, can you provide me with some of your overall general feedback regarding the study?

**Interviewee:** This is just like I can also just describe my personality, what I guess what I thought was very interesting. When I first started the study, I was really annoyed with the frequency of the watch. At first before we got the watch, it was always just through our phone and that was pretty good. Once I got the watch, it was super annoying. Because I felt like every 10, 15 seconds there was a buzzing on my watch.

As the year went on, I noticed that it was a lot less. It was a lot less frequent, which was really helpful because I work-- at the time I got it, I was still in school. Just a couple times I'd be trying to pay attention, I'll be smacking it and sometimes I would accidentally hit the wrong one. I can't undo it. It doesn't give me a lot of chances to free answer. I wasn't sure it was always accurate. It was probably one or two times a month rather than every single time I hit something incorrectly.

Going back to the frequency aspect, it was a little tedious. I'm sure that's part of the second survey it asked. This is just me embellishing it regarding that aspect. It definitely was very disruptive a lot of times. After having it for let's say half a year plus, it got a lot easier to manage. Then I started encountering a lot of more technical issues. For example, when I started doing the burst period, I had a lot of difficulty trying to answer the questions on a timely manner because sometimes I'd be multitasking and trying to answer it, making sure I get the eight minimum.

In the beginning, I really wanted to do the 11 to get the extra cash. I'm very cash-oriented. [chuckles] I was like, "Ooh, money." It was a lot harder than I thought to get it. Which is probably why you want- that the model was made the way it was. I found myself struggling to answer it all eight minimum a day. I also just realized it might also be my phone issue. I don't know if it's just what it was. There was a lot of issues with me trying to even know when the surveys popped up. I'd be waiting. I'd even leave it right there waiting for it to ping every hour for when the burst period was.

There was a couple months, I think I had that problem. I was just a little frustrated about that because I felt like I wasn't going to get the basic pay that I usually would've gotten. I was like, "Oh, man, this is a little unfortunate." I just noticed that there was a lot of tech issues from the app on my phone. Keeping in mind my phone is a Pixel 3a. It started getting a little old.

Now looking at my own phone, my messages aren't even sending. There's some weird thing happening on my phone too. I was like, I don't know how much of it was the app, how much of it was my phone. Since I don't have another phone, that's-

**Interviewer:** It's the only option.

**Interviewee:** -that's Android. It's the only thing I can use. That was something I thought was interesting. Maybe if there's a broad consensus of an issue with the surveys popping up, that might be another issue.

The other one I had was also my watch kept dying. When I would be working out, it would be really awkward because sometimes I rock-climb so my hand presses down on the button. I think it either shuts it off or something happens and the spring completely freezes and I can't answer any more surveys and I can't do anything until the watch completely dies. Sometimes it was on the full charge and it dies for me to recharge it and have the watch back to standard usage.

I thought that was difficult for me personally because I would have to miss a couple of days because it wouldn't charge. I did reach out a couple of times and everyone was really responsive when I used the different things. There was one point where I was going to send the watch back because I thought it was broken. It worked after just giving it a hot second. I gave it two days death and then it charged again.

**Interviewer:** It revived. It revived.

**Interviewee:** I was like, "Oh, this is really weird." For the most part, I thought the experience definitely had some tech issues there. At first, I thought it was because I'd used my watch for [chuckles] exercise, but it was okay.

**Interviewer:** [chuckles] That's interesting though, with rock climbing.

**Interviewee:** Yes. My hand or something.

**Interviewer:** Yes, your hand. Yes. That's--

**Interviewee:** Oh, when I'm trying to talk through, I have presentations, I'm leaning and sometimes it hits there. I switched it a couple times before and it was okay. My left hand's my dominant watch hand versus my right. Sometimes I do that it also just turns off. I thought that was interesting.

**Interviewer:** Those are valid things to know, especially when we're trying to capture physical activity. These are things that maybe having a watch is a downside. That's good to know.

**Interviewee:** It's probably a lot better for people who don't than have it so close up to here. Because for me, I have a smaller wrist than normal. I needed to get--

**Interviewer:** It's a big face.

**Interviewee:** Yes. I needed to get the extra band to really tighten it. Then even then my wrist is just perfectly smacks the thing. I wonder if that's the reason. Overall that was pretty good.

I thought in regards to the questions and stuff, I thought some of it was helpful, because I myself, I remember the study itself had to do a lot with anxiety and depression and things like that. That last year, I actually went through pretty major depressive episode. I'm okay, but I thought that it was really helpful to be able to have a lot more self-awareness for me because I was able to monitor my moods. At that point, I actually thought those were super helpful for me to learn about myself. I looked forward to the burst periods to see, "How am I feeling throughout the day?" It was really cool.

Some of the questions also, although some of it was repetitive, I felt like the phone app questions were pretty solid. Then the ones on watch was weird sometimes. Because I'll be reading it and I wouldn't really-- some of it would get cut off. I would guess some of it. Then some of them were also asking the same question twice and I'll just be like, "I just answered this." I guess I answer it again.

**Interviewer:** That happens. Yes.

**Interviewee:** That was really interesting. I'm trying to see what else I can answer. Those are my initial--

**Interviewer:** No, those are good. Some of the questions I ask may remind you of things. Please feel free to tell me then, or if something comes to mind, interrupt me anytime. That's totally fine. Any feedback is welcome. We want to learn about your motivation for joining the study, staying in the study, things like that. First question I have for you is, how did you learn about the study?

**Interviewee:** My sister's ex-partner at the time, [laughs] he was doing it. He was trying to get my sister to do it. Because he's like, "You get money. This is really great." I was like, "Money?" [laughs]

**Interviewer:** You're like, "Sign me up." [laughs]

**Interviewee:** Yes. [laughs]. At the time I was still a student. It was like a side contract. I wasn't allowed to get a full-time job or part-time job because my-

**Interviewer:** Oh, wow.

**Interviewee:** -other job would be happening too soon. I couldn't get any money. I was like, "Oh, man, this will be a rough couple of months until I start full-time." Then I heard about this. I was like, "Oh, my gosh, I could get a little extra pocket. [crosstalk] It was through a friend and he send her the information. I stole the information [chuckles]. I signed up because I'm more-- she had the chance to. I might encourage her to try it. I've been trying to encourage her to try it, but she's a little bit less motivated to do things consistently.

**Interviewer:** [laughs]

**Interviewee:** For me, I really wanted to get extra money. That's honestly the true motivation. Afterwards, I learned about the study and it was really cool.

**Interviewer:** What about the study when you learned about it? What interests you most?

**Interviewee:** I think originally I thought, so the way that her ex pitched it to me was that it was for cancer or health. I thought that was really interesting. Then I looked into it a little bit more and I remembered it's about stress and anxiety. Because I was going through that phase of my life and having a really depressive episode, I was like, "This is probably going to help us a lot as a society." Especially with how the course of mental health is going and just how saturated the market is, yet not enough accessible resources are out there.

On top of that, psychology, especially mental health and psychology hasn't been fully funded and researched extensively. There's a lot of new factors, especially for people in my generation. My age group, we're facing just really different situations and challenges compared to what our parents went through. POC, you can hear a lot of people have a lot of trauma from their country from moving over [crosstaslk]--

**Interviewer:** In our generation.

**Interviewee:** Yes. Also war torn countries. Then oftentimes my parent would be like, "Oh, back in my day, you didn't have to struggle through these things." I was like, "That's true, but we're struggling with a very different subset of issues, and I think if we can't dismiss that." I thought that it would be really great to partake and participate in something like that, especially since there's so little research being done nor betterment of really traditional and more dated [chuckles] psychological research. I thought that was also like a really comforting point of like, I'm doing something to help.

**Interviewer:** I love it. That's awesome. Good.

**Interviewee:** Thank you.

**Interviewer:** Thank you for coming to my Ted Talk. [laughs] That was so good. That was awesome. Can you describe what motivated you to continue to answer surveys in the study?

**Interviewee:** It always was a bit of the monetary factor and then the other factor was also like I found little bits of joy in the consistency that we talked about earlier. I looked forward to something to do and also it was very simple and also in a small way, it was like having a buddy [chuckles] checking on you. How are you feeling? Are you planning to exercise? Do you sit? Do you eat? It reminded me to not just stay stationary. I think it just encouraged me to think about things I wouldn't always think about.

Prior to that, I didn't think about the amount of how long I would sit. I felt ashamed at first. "Yes, I'm going to sit for four or 4 hours because of my profession." At the same time, I also realized that maybe this is how our world's headed to and how things are in totality. I thought it was really interesting. What motivated me to keep going is partially because I don't like to give up. Secondly, I just really enjoyed having that monotonous consistency and just being able to have a level of self-awareness and self-check-in. Sometimes the survey dates were at inopportune times, but that's part of the reason why it's going on, so you can catch it-

**Interviewer:** Knowing those things.

**Interviewee:** -happening out there.

**Interviewer:** Yes, definitely. How feasible is it in real life? When things real things are happening? Real-life things are happening.

**Interviewee:** You just learn to adapt, adapt and revise and recover. [chuckles] That's what you definitely do. Oh, it makes me think actually, if you wanted to add additional question, maybe one of them can be like, did you eat water when you want to drink water or something like that. Healthy eating was a really interesting one, but I wonder if for specific people, they also struggle with eating consistently because some people don't eat consistently in high-functioning jobs and things like that.

**Interviewer:** Students especially, like undergrad students.

**Interviewee:** One almond a day? Easy.

**Interviewer:** Yes, you're like, "I got this." Have a beer at night and an almond in the day and I'm good. [chuckles] Can you describe to me the process of answering surveys on a typical burst day?

**Interviewee:** On a typical burst day? Because I knew what was coming next, eventually be able to really quick, how am I feeling? Then this is probably an answer. On a typical burst day, I'll do like a start to finish. I wake up and check to see how many I missed. Because sometimes I plan to wake up at a certain time but end up not waking up at the time I hope for.

**Interviewer:** Sleeping in longer?

**Interviewee:** Yes. Sometimes I just struggle to wake up as the week goes on. I'll check how many I missed and I get up and try to answer as many questions as much as I can. I would spend one or two seconds, a few seconds just looking at it and being like, "This is how I'm currently feeling." I'll just try to really focus on them in the moment versus like, "Oh, I could feel like this." Just try to not try to change my answer too much.

I'm trying to think of a typical burst day, how that would look like, how to describe it. It's just basically like I was trying to have my phone with me as often as possible, keep my phone charged and just have it right here. Typically I would be either at home or on occasion I'll be out and about trying to meet with people. If it's a weekend and my phone app is like, wait, I'm so sorry, give me a second, I need two minutes and I'll answer my questions. Over time, I've gotten better about multitasking the destruction aspect. It's not too bad. People get curious like, "Oh, what is it?" I'm like, "Oh, it's this." Most people have a lot of iPhones, so I'm like, don't really qualify for it because you get an Android.

**Interviewer:** What did you typically tell them?

**Interviewee:** I told them it was a survey that pays me. [laughs] I say it's health, they're doing research on health and the betterment of the course of humanity. They're like, "Oh, that's cool." I was like, "Yes." Then they saw how truthfully you had to answer the questions and they're like, "Oh, that's not cool anymore." [laughter]

**Interviewer:** They'll never mind. [laughs] I'm glad I have an iPhone. [laughs]

**Interviewee:** Good icebreaker when it's really awkward.

**Interviewer:** Yes, for sure. Sorry, I have to answer this survey. I'm going to have to step away. [chuckles]

**Interviewee:** Yes. If I need to get out of awkward situation. Sorry, this survey needs my full attention. What letter is in Donald Duck's name. [laughter]

**Interviewer:** It's going to take me an hour at least. What did you think of those questions? The questions that were not related to measuring health and activities?

**Interviewee:** How do I think about the purpose of the survey, because I kept telling myself different things. At one point, I was like, "Oh, what is the survey for? Oh. It's for Alzheimer's." I was like, "Wait, No. What type of survey am I doing?" I think I got a couple of different surveys mixed up in my head, just because ever since I started doing a survey, I think there's an algorithm that says, oh, you like to do surveys. Would you like to join this survey?

**Interviewer:** For sure.

**Interviewee:** I thought those were really fun and they're really enjoyable to disrupt your method of thinking. It did make me feel bad when I didn't know some of the answers. I was like, "What do I put?"

**Interviewer:** You're not alone.

**Interviewee:** For the most part, they're pretty good. It made it more entertaining. I think it helps deescalate the frustration of the quantity of buzzes I would get in the beginning. This is really cool. I wish there was a few more on the phone versus on here, but because when you're on the go, I know the reason why we have the wash is probably more for convenience and answering, but I feel like it's such a quick catch and go process.

The questions you have can't be too mentally intensive and you have to click it and continue versus on the phone, there might be opportunity to have some more lengthy or more thought-provoking questions, if you're serious, you can ask them those. That's an opportunity you can definitely have, I think, on the phone versus on the watch. That can be more the end of the day. At the end of the day or beginning of the day. Someone can get a general larger or deeper question. If that's something that you want it to look into.

**Interviewer:** Yes, it's a good idea.

**Interviewee:** If it's all good, it's totally fine. I know it's **[unintelligible 00:19:01]**.

**Interviewer:** No, honestly, we take all of the suggestions that you guys give. All of it is very helpful. Thank you. Truly. What would have made participation in the study more fun or rewarding?

**Interviewee:** I think being able to see the amount of money I was making consistently. Maybe there's a little aspect of it that's more easier to track because I wouldn't want to always just go on to check what my balance was as a message or wait at the end of the month to get an update or actually even having a little similar to how there's, like a burst period.

**Interviewer:** Like a bar?

**Interviewee:** Yes, it's like a bar. You're like, "Oh, okay, so this is how much I'm making." I will be able to answer it a little bit more frequently because I didn't see anything. At one point, I felt frustrated. I don't know how much I'm actually making. If I even hit my mark, am I going to be able to keep the watch? Am I going to get the basic amount that I hope I can do? It feels bad to also not hit the quota then it just a little upset.

I know that was like a frustration I had and then at one point I was like, "Ugh, it is what it is." I partially gave up on consistently trying to answer right away. If I hear a buzzing in the other room, there's one time I was like, "Oh it's okay. I still have eight more." **[unintelligible 00:20:31]** that I would get, so I wouldn't go answer it compared to like typically it would be a lot better to see it, see that progress.

I think also I really enjoy seeing those emails about this is-- X amount people are doing this or this is what the survey says about your cohort or your class doing this, or things like that. I think that was really interesting and I really enjoyed seeing like research because I personally really seeing quantitatively just like the fruits of the labor and **[unintelligible 00:21:12].**

**Interviewer:** Yes. Knowing what you're doing is doing something.

**Interviewee:** I know it's also a lot harder on-- This is a small suggestion. I know that's really hard, because there's not enough bodies or hours in the day to do things but if you did like bimonthly, like every other month you have like a little newsletter saying, "Hey," something about what we're doing or this is the progress and this is what this says about you. It helps, like, helps me be able to like keep track and look know more about what the study has to do.

**Interviewer:** Definitely. More maybe personalized data or something like that.

**Interviewee:** I think as if there are more researchers or students that like want to improve the process, either they decide to make, if there's more people who are tech savvy, they decide to make a portal like have everything out or a website so people can log in and see things that might not be like a bad way to help consolidate information. People can go there to see what's going on. It's also like I know a lot of how the research appears to be, or the research tech is very bare bones and it does its job. You also want to be more cost-efficient because like research costs a lot of money.

**Interviewer:** [laughs] Yes. Let's see. For this next section of questions you have, have answered or led to some of these, but we want to know about situations of increased burden. We know obviously the study wasn't easy and we of course appreciate you participating, but we want to learn about more specific challenges that you may have experienced. What were some situations in which it was particularly challenging to answer a survey?

**Interviewee:** I know initially when I talked, like when I was interviewing to be eligible for it, they asked how many hours do you drive? I found out I actually had to drive here and there every or so often and it's often unfortunate timing because I can't defer my survey for more than two days and I would actually end up having to go out and do-- I'm an auditor so I had to go count items. **[unintelligible 00:23:32]**, I'd be multitasking and it would be really difficult. I'd be driving for three plus hours or if I'm on that would be the more the difficult parts. I also had a tendency to get a little bit more ill because of I think depression equals sick sickness sometimes.

Also, I would get really sick and I would sleep a lot and there was a couple times I would just sleep through all like a whole day of surveys and I was like, Oh no I feel really bad. I would hit that quota too. That wasn't super frequent. It wouldn't be always on the day of the burst period. It would be more on a standard day, but when I had to do a watch, but when that happened that that wasn't a good time.

Then I mentioned earlier when exercising. I don't always have my phone walking, running around. When the watch with the exercise actually was very helpful so I'd be able to hit it really quick and continue doing whatever I was doing but if it was a burst period, I can't hear it buzzing from five feet away and I have to run into it. If I try to grab it playing badminton or something, I'd be like, "Wait a second guys."

**Interviewer:** I got to answer this.

**Interviewee:** Yes. Those are some examples. Socially I think it wasn't too bad for me because I personally am not a super social person. I don't go out to like a lot of events per se, and if I did I would just say like, oh, give me a few minutes and no one would get too bothered by it. I can imagine those who are concertgoers or they go or exhibit attendees, they probably-- Like art exhibits or things like that. They probably would have some difficulty with that but even so, I don't think it's more of a once-in every so often opportunities.

To consolidate it I would say driving is one. I know most people wouldn't be expected to be driving less frequently, but I found myself actually at least two to three times having to try to answer while driving and okay, it wasn't good. There was a couple of times too, I had peers with me and I'll be like, take it, answer these questions.

**Interviewer:** "Answer for me."

**Interviewee:** Yes. It was helpful and there was a little stress in making, like, as I've gotten through it, there was a little stress trying to hit the eight surveys a day so I always wanted to make sure any opportunity I get I could try to answer them because I don't know why I would just missed one or two, but I'd always be like one or two shy and I'd be in despair.

**Interviewer:** Let's see. I have just a couple more questions here **[unintelligible 00:26:25].**

**Interviewee:** No, totally fine **[unintelligible 00:26:27].**

**Interviewer:** We have a survey at the end. I'll explain that in a little bit. I just want to make sure I save time for that but I'm going to have, I have two more questions for you. These are just about distractions so you told me obviously if you were driving you would maybe potentially give it to someone or do it while driving, but how did you typically handle distractions while taking a survey? If someone else is around or?

**Interviewee:** I would just say like, oh, like would it, can you gimme like two minutes and usually those aren't too bad and I try to multitask by explaining to them telling a story about it. Makes it more engaging with other people. Other times to be honest, I'll be sneakily trained to do it as much as possible and I think that's it. [chuckles] I just try to do it if I can't and sometimes if I take too long then the message just disappear. If I grab my phone too late after a buzz still loads long, I try answering the whole of questions that goes away and I'm sad because I don't think you have to complete it to fruitions for it to count official.

**Interviewer:** Would you notice that you answered differently throughout the day? Were your responses different from morning till evening or if you were in a specific location, would your responses change?

**Interviewee:** I think when I was out and about, I usually had more consistent, like neutral answers. I'll be like, "Oh, I'm meh," or whatever. If I was like, "Are you frustrated today? Are you nervous?" Whenever I'm by myself, I usually am frustrated with myself so if you see a lot of-- Basically, if I'm by myself, I found myself answering more critically and I'd be more upset and when I was with other people, I would be nervous but not as angry. If I'm with my romantic partner or family, I'd be more angry. Now I'm thinking about the, the questions. That's actually really interesting because you can find some correlation between like alone versus something else.

Let me think. I do realize like throughout the day in the morning, I'm usually a little bit more grumpy and at the very end I'm also really grumpy toward myself so mainly more self-critical. I'd be more frustrated really early in the morning and really late at night.

**Interviewer:** It did vary throughout the day then?

**Interviewee:** Yes, and I think it just depends on how you prep your day. That's really insightful.

**Interviewer:** Yes. Interesting to think back on that from the past year. A year's a long time.

**Interviewee:** It is.

[crosstalk]

**Interviewee:** Sorry.

**Interviewer:** No, don't be sorry. Go ahead.

**Interviewee:** For the questions, maybe something you can also ask is some insight too is like to see if anyone has consistent hobbies like if people start projects, how much do they stick through with it if you want to see because I've noticed that there's a couple of times I would start projects and then finish some of them or some of them I wouldn't finish and I think it depended on where I was at in my life. It would be really interesting to see how many people like a resiliency factor with individuals that either finish the whole study to fruition or those who just drop off midway or whatever that may look like to see if they have any like major projects or hobbies that they try to keep up with.

**Interviewer:** Especially this age group a lot's changing. In this time period up until your 30s I can tell you.

**Interviewee:** I started rock climbing actually three to four months ago and since then like I've exercised a lot and that's improved my mental health a lot.

**Interviewer:** Big time.

**Interviewee:** I think if I did the survey while still like exercising consistently I think my answers will all be drastically different to be honest. Maybe that's something to think of like when you're making your questions you can say like have you exercised today rather than saying like are you planning to exercise and it'll be really interesting to see how that turns out.

**Interviewer:** That's good. That's awesome.

**[00:31:04] [END OF AUDIO]**
