# Supplementary material for: Burden and Inattentive Responding in a 12-Month Intensive Longitudinal Study: Interview Study Among Young Adults
Source: JMIR Form Res. 2024 Aug 2;8:e52165. doi: 10.2196/52165 (PMC11329843; doi:10.2196/52165)
Supplement: Multimedia Appendix 1 [file formative_v8i1e52165_app1.zip › Transcripts/moocherchemicalsbanker_audio_7.28.22.m4a.docx]

**Interviewer:** Okay, to start can you provide me with some of your general overall feedback regarding the study?

**Respondent:** Well, I don't really get used to it. I would say. The first two weeks I really got used to it. I don't know, now I don't even notice that, oh I have to make some effort to take my phone, respond to the survey or respond to my watches question, does it feel so natural at this point?

**Interviewer:** Become habit?

**Respondent:** Like just a normal daily life routine. Like, go brush my **[inaudible 00:00:46]**, answer the survey,

**Interviewer:** It's going to be weird now not answering surveys.

**Respondent:** Yes. It feels wrong. It feels like I forget to turn the notifications or is my phone down?

**Interviewer:** Am I doing something wrong?

**Respondent:** Yes.

**Interviewer:** Okay. I'm going to ask more specific questions about your experience. If anything is ever unclear, please feel free to ask me for clarification, but for this first section, I want to learn about just like I was asking before, like your overall experience participating in this study. First question I have is, how did you learn about this study? I know it's been a year. It's been a while. How did you learn about this study?

**Respondent:** I think I have an account on Research Match, and they sent me possible research opportunities, and that's definitely the way I learned about it.

**Interviewer:** Do you remember what aspects of the study interested you?

**Respondent:** I don't know exactly. I think I saw something about like Samsung phone owners. I'm like, "I have a Samsung. There's something about Samsung I need to know."

**Interviewer:** No, there's not, don't worry. Can you describe to us what motivated you to continue answering surveys in the study?

**Respondent:** I guess I was interested in where is it going to, and also questions about, "Are you physically active right now?" They remind me to be physically active, and I said it's sounded like a discipline thing where procrastinating today and I'm like, "Yes."

**Interviewer:** How would you know?

**Respondent:** Yes, I don't know, it's like a discipline thing. I get reminded of what I do when I get conscious about it and sometimes I feel guilty, but I don't know, it's like motivating me to be productive or be physically active or remember like, "Oh, I wasn't physically active today at all." Of course, there's aspect of receiving compensation.

**Interviewer:** Yes.

**Respondent:** Why not? I don't work, I can't work right now.

**Interviewer:** Yes. You must be a student?

**Respondent:** Yes, I am one.

**Interviewer:** How important was compensation having it in the study, how important was it to you?

**Respondent:** I think it was quite important. I guess it's also one of the motivators.

**Interviewer:** Yes.

**Respondent:** I don't know, it's nice that the time that I spent, I get at least something out of it.

**Interviewer:** For sure. Yes.

**Respondent:** My phone has some battery problems, which is if I go somewhere, usually, I would just turn to the most saving energy mode, but with this app I can't because I won't receive any service. I just have to make this choice of like, "I have to spend less time in this other place so that my phone doesn't die and I can order an Uber and get back."

**Interviewer:** Yes, that's a good point.

**Respondent:** Yes. It's nice to receive at least some compensation for making this choice to spend less time.

**Interviewer:** Sorry, my dog.

**Respondent:** It's okay.

**Interviewer:** She's horrible. Yes, for sure. Would anything have made participation in this study more fun and rewarding besides paying more money? That would be more rewarding? I'm listening. I'm just going to put her in the **[inaudible 00:05:01]**.

**Respondent:** Okay. I don't know. I feel like this AD is probably the best one, so I don't know if anything else can really help.

**Interviewer:** You were saying which part is the best one?

**Respondent:** Well, like maybe increase, monitor compensations.

**Interviewer:** Yes. Okay.

**Respondent:** Besides that I don't know what else can there be?

**Interviewer:** Yes. Okay, can you describe the process of answering surveys on a typical **[unintelligible 00:05:43]** day? Like how that was for you?

**Respondent:** Well, I usually don't know when exactly it's the **[unintelligible 00:05:54]** day is going to come. For me it's like, okay, morning **[inaudible 00:05:58]** doesn't stop. I'm like all good, it's Busday.

**Interviewer:** Yes.

**Respondent:** Usually, I just try to have the sound on my phone. Oftentimes, it's muted and I just carry around with me and I carry the charger with me because of those battery problems.

**Interviewer:** Yes.

**Respondent:** I try to turn it off during class period.

**Interviewer:** Yes. Would you turn it on, like do not disturb or do you turn your phone off?

**Respondent:** No, I just turn off the sound.

**Interviewer:** Okay.

**Respondent:** Notification music doesn't ring while I'm in the classroom.

**Interviewer:** Yes. Smart. Would you keep track of the amount of surveys you answered, like on the tracker? Or did you have a goal that you were trying to reach each time?

**Respondent:** Yes, have a goal. I try to have at least 8. From once I hit 8, I'm trying to get 11.

**Interviewer:** Yes, try to get that bonus.

**Respondent:** Yes.

**Interviewer:** For this next section we want to learn a little bit about increased burden that the time study may have caused. Obviously, we know it may not have been easy at times, especially as a student, like you said, being in class. We want to learn a little bit about the challenges that you may have experienced. What were some situations in which it was particularly challenging to answer surveys?

**Respondent:** Well, as I said in the classroom. Also when I'm on a phone call and, all of a sudden, the sound happens and person on the other, I don't know, side is like, "What is happening?" [crosstalk] It's the scientist? No. They want to talk to you and I don't know for some reason it only happens when my mom calls me.

**Interviewer:** Oh, wow.

**Respondent:** I don't know why.

**Interviewer:** That's interesting.

**Respondent:** As soon as I'm talking to her, it's always going to be a survey or a location survey. It's something like that.

**Interviewer:** That's crazy. I swear on our end there was no way for us to do that, but that's actually interesting though, she brought on the survey power.

**Respondent:** I did. I guess as a bit of a challenging part, again, it's connected to being a student, but I don't know, I had a class where we needed to do some public speaking, make speeches and I feel like almost every time I have to do this public speaking, my watch wants to ask me something.

**Interviewer:** Oh, and it's so loud.

**Respondent:** Yes. I'm still talking [crosstalk] and my watch is glowing and wants attention.

**Interviewer:** Oh, I'm sorry. That's terrible.

**Respondent:** That's okay. I don't have [crosstalk].

**Interviewer:** That is a challenge. Good.

**Respondent:** Yes, and I guess sometimes when I need to quickly use my phones for something and then it pops up and I'm like, "I need to do this really quick. I can't just quickly answer and it's taking time."

**Interviewer:** That's a good point for sure.

**Respondent:** It happens not that often. That was okay.

**Interviewer:** How did you typically or what most frequently led you to miss answering a survey? Instead of answering it or be unable to, if you were like in class, obviously, if you're doing speech, you're not going to be, "Hang on, let me answer the survey."

**Respondent:** Yes. In the classroom or I don't know, like when my phone is charging in the one room and I'm in the other room and I hear it and I'm like, "I'm just busy. I can't just stand up every time. Walk there, get the phone sometimes," I'm just like, "No, I simply cannot do this."

**Interviewer:** Were there instances where you preferred to dismiss it you saw it and you're like, "I'm not now," and you just dismissed it?

**Respondent:** No. I don't think I ever dismissed a question, but I don't know questions about, "Do you feel in control or I don't know are you procrastinating," because maybe I'm procrastinating other things but I'm being productive in something else and I don't know how to answer it. If I'm being pessimistic, then yes, ultimately, I'm so procrastinating, but if optimistic then no I'm not because I'm doing this other thing, I'm just prioritizing. That's why I usually answer the moderate level because I don't know, am I procrastinating?

**Interviewer:** Would it depend on your mood at that moment, how you would decide to answer it? Or would you generally just go for the moderate route?

**Respondent:** Honestly, I generally just go for the moderate one because if I wasn't doing anything at all during the day, procrastinating everything that obviously, it's yes, very much so. If it's this situation when I'm just doing homework for one class, and I'm not doing for the other because I really don't feel like it.

**Interviewer:** I remember those days.

**Respondent:** It's just moderate because I simply don't know how to answer here or if I wasn't doing any schoolwork but I was really physically active or doing something, some other activity, then how is that procrastinating if I'm being active. Which is why again, I'm answering moderate because I don't know how to possibly evaluate my day. Based on the question of are you procrastinating right now? It's depends on what.

**Interviewer:** Yes. We're all procrastinating on something at all times of day, that's a good point. What did you typically tell friends or family about the study? If they ask about it. For instance, your mom, when your phone's vibrating, when she calls?

**Respondent:** I usually just say that I'm participating in this study. I don't know, I remember in the beginning I would be in a cafeteria, all of a sudden, the survey is playing and I'm sitting with friends. I'm like, "Oh that's the scientist, never mind I'm still listening to you."

**Interviewer:** I love that. That's the best.

**Respondent:** Yes. They get used to it. At first it was a bit weird, we're in the middle of conversation, I'm like, "Oh yes, I'm listening to you. I'm listening. I'm just not looking at you.

**Interviewer:** Yes, for sure, keep talking.

**Respondent:** Yes. Don't let it interrupt our daily activities.

**Interviewer:** I guess along those lines of when you're taking a survey, how did you typically handle distractions when taking a survey?

**Respondent:** I try to pause everything else, quickly answer it. It depends if there are people around me then I have to be like, "I need to answer this real quick, hang on."

**Interviewer:** Don't mind me.

**Respondent:** Yes, but if I'm just alone and I need to do some homework, then, of course, I'll just be like, "Homework, move aside I have a survey."

**Interviewer:** Yes. Were there situations in which your responses may have been less accurate? For instance, if you were like talking to friends and you're like, "I just got to get through this." Or maybe a certain time of the day.

**Respondent:** I guess sometimes because I feel like in those situations I tend to answer moderate often because I'm like, "I don't have time to evaluate completely my day in the situation." I just do it based on my immediate feelings and I don't really go down to think about [crosstalk] it. If I'm alone and do nothing, of course, I'll be like, "Am I sad right now?" If I'm with friends I'm like, "I don't know, maybe a little bit, I don't know what to say." I guess I'm happy. Not extremely, but a little moderate.

**Interviewer:** Moderate, easy. Do you think your motivation or accuracy changed the longer you were in the study? Or as the study went on?

**Respondent:** Yes. I feel like it definitely changed because at first, there was this little excitement of like, "Oh I received the survey. It's awesome." Later it's like, "I have a survey. Everything, sorry, I need to do it," and definitely there was not as much excitement later on. I still think I was answering accurately because it takes more time to create an inaccurate answer than just be like, "Am I sad? Maybe a little bit, am I happy? Moderately," then aside from procrastination question that pauses me every time I see it.

**Interviewer:** Yes. Definitely.

**Respondent:** I feel like it was still as accurate but I wasn't that motivated because I was like, "Oh I just need to do it."

**Interviewer:** Yes. Was there anything that made the study harder or easier as it went on?

**Respondent:** I wouldn't really say that. It depends. If it's like a fallen **[unintelligible 00:16:34]** or like Christmas in **[unintelligible 00:16:38]** or something like that and, of course, it's easier to be answering those questions the first days. If it's still like a school year and it's just not that easy because I may be not just in a classroom, I may be doing something immediate for the class and that's a bit harder and especially when finals week I guess you remember that [crosstalk].

**Interviewer:** Oh, okay. Let me tell you something. Nothing in life will ever compare to the stress that you feel during finals week. Nothing, that is the most stressful time of all the land. Oh my God, I'm so not jealous of that.

**Respondent:** I was lucky I only had like one exam concerns about it. It was French exam.

**Interviewer:** Oh, is that bad?

**Respondent:** Yes, but still I had three other big writing [crosstalk].

**Interviewer:** Papers and stuff.

**Respondent:** Maybe it's an exam when I'm timed and I need to do multiple answer questions, but still it's a lot of sitting and working and trying to not be stressed about it but it's hard because, I don't know, it's just hard. It's finals.

**Interviewer:** Yes. Your grades sometimes depends on it.

**Respondent:** Yes, and it's hard to answer surveys to think about I need to pause my coursework to answer because I'm like, "No, I can't pause my coursework." If I forget about the **[inaudible 00:18:08]**, I may get stuck on it.

**Interviewer:** Yes, that's a good point. Yes, you lose your train of thought or something.

**Respondent:** Yes, and I feel like during the finals week answering surveys it's not fun at all. That's the time when I probably didn't have as accurate answers because I was like, "I need to get through this real quick and keep working."

**Interviewer:** Yes. That makes sense. Absolutely. Let's see. One last main question here, and this is off the topic, not on the topic of accuracy. What did you think about the questions and messages that were not related to measuring either health behaviors, routines, or moods that came up both on the phone and the watch?

**Respondent:** Yes, I feel like definitely questions about like, "Are you in control? Are you procrastinating?" Those are probably, I was like, "How is it really related to health?" I understand it's sadness and happiness and maybe it's definitely stress, frustration, being nervous, those things are related, but how is my productivity level, my health at all and this question on the watch, like procrastinating today. I feel like all other questions on the watch, aside from physically active or sedentary, everything else is like, "Why? Is it important?"

**Interviewer:** Do you remember the random questions that would come up?

**Respondent:** Yes. I feel like there was a random question about is technology bad. Something like this. Technology replace people and I'm like, "I don't know. It depends."

**Interviewer:** This is too deep. [chuckles]

**Respondent:** I don't have expertise in answering it. I don't know.

**Interviewer:** Yes, absolutely. Are there any other points that we didn't discuss that you would like to tell me about that came up?

**Respondent:** Yes. I remember one time when I was traveling by train and the trains only go here at weird times at night. I made myself fall asleep at 7:30 in the evening to wake up during like 1:00 so that I can go on the train. I remember how the time-setting app was not ready for such weird wake up times. When I was in the train and it gave me the evening serve and I told it that, "Yes, I've been sleeping during that time." It counted as if it's been two days in the room.

Yes. I was, like, "But it's not two days in a row. It's just that I was sleeping during that time and now I'm not sleeping because I'm traveling." I think that sort of thing happens two times because I was going back and forth. I don't know. I just feel like the app is not ready for times when people wake up at irregular times or do traveling. [chuckles]

**Interviewer:** Yes. It kind of wigs out with that or time zone changes, "What's happening right now?"

**Respondent:** Yes. It's not that much of a problem, but that creates this inaccuracy because I don't know. The app just doesn't understand that I'm traveling. [chuckles] I wish I could just be like, "I'm traveling right now. It's okay."

**Interviewer:** Yes. Don't worry. [chuckles]

**Respondent:** Yes. It's not been two days. It's still one day.

**Interviewer:** [laughs] Was it on the weekends? Usually, was it like a Saturday when it would do that? Like Saturday to Sunday? I know Saturdays, it would prompt you twice sometimes.

**Respondent:** Well, I don't remember exactly. I think it was during the week when I went traveling. It was some kind of a break, like fall **[unintelligible 00:22:29]** usually, it doesn't end on the weekend. It's in the middle of the week.

**Interviewer:** Middle of the week.

**Respondent:** Yes. I think I received two of those stories because it said that I had this whole day.

**Interviewer:** It's like, "Where were you during this day?"

**Respondent:** Yes. **[inaudible 00:22:52]**.

**Interviewer:** That's a good point to bring up. Anything else that came up?

**Respondent:** Sometimes during first days, I just wouldn't receive a survey for five hours. After those five hours, there will be.

**Interviewer:** When it came in, do you remember seeing if it showed missing surveys on your app?

**Respondent:** It shows like it's missing surveys, but that's just not possible because the phone is right next to me with the sound on. Where are they?

**Interviewer:** Yes. That was kind of a glitch that we were working with, was the notification prompts, it wasn't prompting. Even though it said they were coming in. Did that happen often?

**Respondent:** I would say it happened at least five times, different days, different months. I remember because I'm like, "What is happening?"

**Interviewer:** I know. I'm like, "I'm doing my best here."

**Respondent:** Yes. How did I miss five of them? I've been here all this time.

**Interviewer:** It wasn't you. Don't worry.
