# Supplementary material for: Burden and Inattentive Responding in a 12-Month Intensive Longitudinal Study: Interview Study Among Young Adults
Source: JMIR Form Res. 2024 Aug 2;8:e52165. doi: 10.2196/52165 (PMC11329843; doi:10.2196/52165)
Supplement: Multimedia Appendix 1 [file formative_v8i1e52165_app1.zip › Transcripts/resalepartlyfrigidity_audio_5.31.22.m4a.docx]

**Interviewer:** To start, can you provide me with some of your overall general feedback regarding the study?

**Interviewee:** Well, the questions could probably be clarified. Some of them were confusing. It wasn't always clear what the question was trying to get at. I found the phone way easier than the watch. I assume you guys added the watch because other people felt the watch was easier.

The main thing was that the notifications on the watch are so short that if you're doing something with your hands, which I bake a lot, often by the time my hands are clean, the notification is gone versus the phone went through a bit longer. That made it hard to respond to a lot of the ones on the watch itself. It wasn't all that disruptive. Sometimes when taking drives, it would pop up over the GPS, which was annoying. I don't know, it was fine.

**Interviewer:** You talked about some of the questions not being very clear on their meaning. Was it both on the watch and the phone? Was it one over the other?

**Interviewee:** I think usually the watch ones partially because they were really short. Sometimes it's hard to tell exactly the question that was trying to be asked.

**Interviewer:** Did you typically answer the ones that were a little confusing, the same throughout the study, or did it vary depending on circumstances?

**Interviewee:** Probably by month two, I just entered them however I had been answering them, and probably in a few weeks, I was like, maybe they mean this.

**Interviewer:** For this first section of official questions, I want to learn a little bit more about your experience participating in the study in general like how did you learn about the study, things like that? First question is, how did you learn about the study?

**Interviewee:** A friend who's also participating sent it to me and said, "You have an Android, right?"

**Interviewer:** Then you decided to. Was there anything about the study specifically that stood out to you that made you want to join?

**Interviewee:** I've never done anything that long. I've done focus groups but that's it. It was interesting and new I guess. It didn't seem like it was all that invasive.

**Interviewer:** Can you describe to me what motivated you to continue answering surveys both on the watch and the phone in the study?

**Interviewee:** We used the card for date money.

**Interviewer:** Yes good. That's awesome. That's the best. Compensation was important, obviously, an important part of the study. Can you describe the process of the answering phone surveys on a typical birthday, so those four days of a bunch of surveys?

**Interviewee:** Yes. Every hour-ish, it would pop up. Most of the time, I got it on the first a beep, but if I didn't, it would go off again a few minutes later and go through the 10 or 12 questions. Then an hour later, it would prompt again and then a couple of times it checks your sleep time. I think twice a day.

**Interviewer:** Yes. How many surveys do you think you answered on a typical verse day?

**Interviewee:** Probably nine.

**Interviewer:** Did you have a cert-- [crosstalk]. Oh, go ahead, sorry.

**Interviewee:** I don't think I hit the 11 most days.

**Interviewer:** Did you have a certain goal that you were trying to reach for those?

**Interviewee:** I was usually trying for the 11.

**Interviewer:** What would have made participation in the study more fun or rewarding for you as a participant that are not money related because paying more would obviously be more motivating?

**Interviewee:** I don't know if more motivating but easier would be being able to move the verse periods a little bit further one way or the other. Just because we had multiple times when it fell on a wedding or a funeral or a family vacation or whatever, where it was harder to actually get to the number.

**Interviewer:** That's a good idea. For the next section, I just want to know about certain situations of increased burden that the study might have caused. We know obviously that being in the study wasn't easy at times, and we just want to learn more challenges that you may have experienced. What were some situations in which it was particularly challenging to answer the surveys both phone and watch?

**Interviewee:** I think primarily if I was traveling or if I was driving. We do a lot of weekend trips. If I'm driving, I obviously can't be respond sending. Also, longer drives makes it harder to keep the locks alive.

**Interviewer:** Yes, that's true too.

**Interviewee:** I think that was the biggest difficulty is just scheduling-wise.

**Interviewer:** What was the most disruptive part about the app or the procedures in the study?

**Interviewee:** The sound is obnoxious. I'm like, "Oh, gosh, please stop going off." That's a little disruptive. On the first days, they're long but it gets faster as you get used to it because you know the questions. It wasn't disruptive later in the 12-month period.

**Interviewer:** What most frequently led you to be unable to or to miss answering phone surveys besides obviously driving as you said earlier?

**Interviewee:** I don't know. If I'm in meetings, my phone is on silent.

**Interviewer:** Were there any instances where you preferred to just dismiss a survey instead of answering it?

**Interviewee:** I ignored some of them. Is that the same thing?

**Interviewer:** Yes, same thing. If you just click on ignore, make it go away.

**Interviewee:** Yes, if my hands were a bit full or whatever. Usually, if my phone was on me, I answered it.

**Interviewer:** What did you typically tell friends or family if they asked about the study, or if work was asking about the study?

**Interviewee:** That I was getting paid to participate in a research study and most of them were like, "Oh, cool. How do I join?"

**Interviewer: "**I want to be part of that." For this next section, I wanted to learn a little bit about response accuracy. Besides obviously not answering, we're curious if there are any other ways that you dealt with maybe some challenges or burdens like distractions and whatnot. How did you typically handle distractions or anything going on around you while taking the survey?

**Interviewee:** There were definitely times when I timed out halfway through one of the verse surveys, and it would just disappear and I'd be like, "Oh, darn it." Other than that, they were generally short enough that I could either wait to answer them or answer really quickly and get back to what I was doing.

**Interviewer:** Able to set aside time for it.

**Interviewee:** My husband knows what it is when it goes off.

**Interviewer:** It's like there's that study again beeping you or watch vibrating. It's so loud.

**Interviewee:** It's really loud.

**Interviewer:** It is. Did your responses change if someone else was around or if it was a certain time of the day? Did you notice that your responses would change?

**Interviewee:** No, I don't think so.

**Interviewer:** How do you think your motivation or accuracy changed as you were in the study longer? Did get easier, harder over time?

**Interviewee:** I think I just got more used to the questions so less second-guessing what are they asking for. I don't think necessarily time and study affected the accuracy. I know there were times when I caught myself like, "Wait, I didn't read that, go back," but I don't think there was any rhyme or reason to win that.

**Interviewer:** Last specific question here. What did you think about the questions and messages that were not related to measuring either health behaviors, routines, mood, or feelings on the phone?

**Interviewee:** Make sure you're actually reading the question, question? I guess the purpose of them. They were really annoying on the watch because sometimes they would ask them multiple times a day. They didn't really bother me that much on the phone. Rarely prompted them.

**Interviewer:** Any suggestions on how to make those better?

**Interviewee:** Mine was just more like, okay, stop. [I am reading, I promise.

**Interviewer:** Then maybe just not having those repetitive ones. on the watch helped make it better.

**Interviewee:** Yes.

**Interviewer:** You mentioned this earlier. I'm going to change up the questions a little bit. You mentioned having a friend in the study. is it just one person that you knew in the study or multiple people?

**Interviewee:** Just one.

**Interviewer:** What is their first and last name and your relationship to them? Of course this information will be kept confidential.

**Interviewee:** Steven Dhou, D-H-O-U. Friend.

**Interviewer:** How frequently do you interact with them?

**Interviewee:** I probably see them two or three times a year.

**Interviewer:** Now questions about exercise. In the past month, have you exercised or performed any type of physical activities such as going for a walk?

**Interviewee:** Yes.

**Interviewer:** What types of exercise do you typically do?

**Interviewee:** Walking most often, hula hooping--

**Interviewer:** That's awesome.

**Interviewee:** Kind of random things here and there, but those are the regulars.

**Interviewer:** Hula hooping is so much harder than it as a child. It used to become so natural as a child, and then you get older and it's like, "What happened to my hips?"

**Interviewee:** I know. I used to just be able to hula hoop for hours, and now ten minutes is pushing it.

**Interviewer:** It's a workout too. It's hard. Oh, that's awesome, though. I love that. Are there certain days of the week that you go for a walk or that you're doing any hula hooping?

**Interviewee:** No. It just tends to change based on what appointments are that week.

**Interviewer:** Where are you typically going for a walk or doing hula hoops? Is it outside? Are you on a treadmill?

**Interviewee:** Usually in our neighborhood.

**Interviewer:** Okay. Let's see. Were there any instances where the phone surveys or the watch surveys disrupted your sleep?

**Interviewee:** Probably not after the first couple of weeks. I know, I, a few times, set the sleeps wrong and it would wake me up and I'd be like, "Argh."

**Interviewer:** An intense wake up. [crosstalk]

**Interviewee:** Back just through my probably snoozing sometime.

**Interviewer:** Okay, so that didn't bother you that the rest of the study after you figured that out? Cool. Okay, good. Did you have any technical problems with the phone or the watch time app that you solved yourself without our assistance?

**Interviewee:** Just the faces changing on the watch. The other issues that I had, I did have to reach out for, like the app crashing and stuff.

**Interviewer:** Do you remember seeing any of the newsletters or birthday emails that were sent?

**Interviewee:** I remember seeing at least one newsletter, yes.

**Interviewer:** What did you think of at least that one?

**Interviewee:** It was slightly interesting. It would have been more interesting to see more data, "Thanks for participating." It'd be cool to see because I know it had a couple of "here's what we've learned so far" that were interesting.

**Interviewer:** We hope once you're done with data collection, once everyone's done, that we can start sending out some personalized data to you guys or visualization or something, which would be more interesting for you guys to see. Final question here, what will you miss most about this study?

**Interviewee:** Date money.

**Interviewer:** Yes, for sure. You're going to have to find another study to get a monthly date money fund. I love that. That's awesome.

**[00:13:50] [END OF AUDIO]**
