# Supplementary material for: Burden and Inattentive Responding in a 12-Month Intensive Longitudinal Study: Interview Study Among Young Adults
Source: JMIR Form Res. 2024 Aug 2;8:e52165. doi: 10.2196/52165 (PMC11329843; doi:10.2196/52165)
Supplement: Multimedia Appendix 1 [file formative_v8i1e52165_app1.zip › Transcripts/beavertomatoupscale_audio_8.3.22.m4a.docx]

**Interviewer:** Awesome. Okay, so to start, can you provide me with some of your overall general feedback?

**Interviewee:** I think generally, it's pretty good because I think you already have a bunch of other people who do this before me, so I think it was improved, and in the middle of my study, there's some small changes that I can see. I think generally, it's good. All the question is good. Some very interesting questions on the watch throughout the day. I see you have some questions to check if the answer is for you. It's very interesting to see some general knowledge questions, something [crosstalk] this. She's really good. Let me think.

One thing that I didn't like is I always put my phone on vibrate mode and I assume it's supposed to vibrate during bus periods when I have a survey, but I don't think it's ever vibrate, so I have to remember around what time during the hour I had the survey. I had to turn my phone on to do it.

**Interviewer:** Oh, wow. It didn't vibrate. Was that the whole study it did that for you?

**Interviewee:** No, I think it changed. At the beginning, it vibrate, and then something chang and it stopped.

**Interviewer:** That's frustrating.

**Interviewee:** That's fine, I still remember what hour, so that's fine for me, but I think that's something you came from, I don't know, what happened with the App.

**Interviewer:** Yes, definitely. You would literally just have it, you would turn your phone on sound or would you just look at your phone to see if a survey was coming in?

**Interviewee:** Yes. I think when you said like if it started like 9:30 and the next one will be around 10:30.

**Interviewer:** Yes, roughly then.

**Interviewee:** Yes.

**Interviewer:** You're dedicated, that's for sure. Okay, I'm going to ask more specific questions about the study. If a question's ever unclear, please feel free to ask for clarification, but I want to go into a little bit more about your overall experience in the study. The first question I have is, how did you learn about the study? How'd you first learn about it?

**Interviewee:** I have participated in a research match, so they matched two research study. That might be interesting.

**Interviewer:** Do you remember what aspect of the study when you saw it originally intrigued you or wanted you to be interested in it?

**Interviewee:** I don't remember exactly.

**Interviewer:** I know, it's been a year.

**Interviewee:** It's almost a year ago. I think tracking my health is something that I'm interested in as well because after COVID, it's like I don't want to do all the physical activity. I think have something to track my health. I still have something [laughs] to check my health. I want to see how that goes, and I do research as well, so I've never seen a one-year research, so I'm just curious how that works.

**Interviewer:** Yes, definitely. That's a big commitment for sure, a year of research.

**Interviewee:** How long has this been going on? Like three years?

**Interviewer:** We are actually wrapping up data collection. We're in August now, so this month in a couple of weeks, we'll be done. Yes, we're about three years in, so you're at the tail end of data collection.

**Interviewee:** That's great.

**Interviewer:** I know, we need to have a time party or something.

**Interviewee:** Yes, when you get published.

**Interviewer:** Yes, exactly. Can you describe to us what motivated you to continue to answer surveys in the study?

**Interviewee:** I do research, so I just don't want to start in the middle. I feel like if I start in the middle, I have to just throw out all my data from the beginning and that's going to be a waste. That's why I just keep going and it doesn't hurt me that much. It's just answering surveys every day.

**Interviewer:** The compensation, was that a factor at all for you, a motivating factor at all?

**Interviewee:** I'm not sure. I wouldn't say it's the biggest motivation, but it probably does.

**Interviewer:** It helps, yes.

**Interviewee:** Keeping more money.

**Interviewer:** Yes, for sure. Can you describe the typical process of answering phone surveys on a birthday? Did you have a set goal that you wanted to reach? What was it, morning till evening?

**Interviewee:** Whenever I see it because, for example, sometimes I don't see it. Also, if I'm in the middle of my work, then I just don't bother. Every other time if I see it, then I answer it.

**Interviewer:** Do you have a goal number or like, "I try to reach a certain number"?

**Interviewee:** No, I don't think so.

**Interviewer:** Okay. Besides, we talked about paying more money, compensation is always good, but besides paying more money, would anything else have made participation in this study more fun or rewarding?

**Interviewee:** More fun. Interesting. I guess it's very repetitive, but that's the goal of the study. If it's less repetitive, that'd be more fun, but I don't think that can be changed.

**Interviewer:** Yes. You're talking with like the phone surveys, watch survey?

**Interviewee:** Yes, because right now, the first second I see the question, I know what it's about.

**Interviewer:** Okay. For this next section, I want to learn a little bit more about increased burden that the study may have caused. We know obviously at times it's probably challenging, and so we want to learn a little little bit more about what you may have experienced with those challenges. What were some situations in which it was particularly challenging to answer the surveys?

**Interviewee:** I think the most annoying thing is like when I'm in a meeting, one-on-one meeting with my mentor, and then the watch start buzzing. It's really annoying. I don't want to explain to everyone that I see, "Oh, doing this." Saying that I have to do this. I usually just silence it, but it's quite annoying, but that's the only time. I don't think the buzz survey is that annoying because I think you have a window that you can answer it so you don't have to do it right away. My phone doesn't vibrate, so that's not annoying. [chuckles]

**Interviewer:** You don't have to worry about that, yes. [chuckles] Was it the actual vibration itself that was most disruptive or was it taking the time to actually answer the survey?

**Interviewee:** The vibration.

**Interviewer:** For the watch, I guess.

**Interviewee:** It's pretty noisy if you're in an office and no one's talking and you just start buzzing.

**Interviewer:** It's super loud, yes.

**Interviewee:** Yes, for sure.

**Interviewer:** Are you a PhD student? Then you talk about your mentor.

**Interviewee:** I'm a research assistant. I will get my PhD next year, my dear. Kind of the same thing.

**Interviewer:** Nice. Oh, exciting. Yes, the same thing. Congratulations. That's exciting. You're very familiar then with research. What most frequently led you to be unable to or to miss answering surveys?

**Interviewee:** Can you repeat that?

**Interviewer:** What most frequently led you to be unable to or to just completely miss answering surveys besides, obviously, your phone not vibrating?

**Interviewee:** I think that sometimes, first of all, I bike to work and I can see the question, but I cannot stop my bike to answer the survey.

**Interviewer:** Oh, yes.

**Interviewee:** Or like when I'm in the lab, I wear gloves and I don't want to touch my watch with my nasty gloves and that I just ignore. Those are the only time I think.

**Interviewer:** What about to dismiss? Did you ever see a survey and dismiss it besides like, "I can't answer this right now," but actually just dismiss it?

**Interviewee:** Yes, I think with the watch, you can just click the button on the side to dismiss it. That's during a meeting when it starts buzzing and I actually have to do that to stop it.

**Interviewer:** What about on the phone?

**Interviewee:** I don't know. Can you dismiss a buzz someway? [laughs]

**Interviewer:** Yes, true.

**Interviewee:** Oh, wow. I don't think on the phone you can dismiss it. I think it just expire after a while.

**Interviewer:** Expire, yes. What did you typically tell friends or family or coworkers in the lab if they asked about this study?

**Interviewee:** Actually, I'm doing my research thing that I have to do. My friend question like, "Why is so long? Can you just stop it?" [laughs] "No, I have to finish. It's almost done."

**Interviewer:** They probably just knew they heard the sound, they're like, "Here comes another one."

**Interviewee:** Yes, but I just tell them it's a research thing that I want to do, that's it, and that's usually the end.

**Interviewer:** For this last section of questions here, besides not answering surveys, I'm curious if there were other ways that you dealt with some challenges or burdens while in the study. How did you typically handle distractions? When actually taking a survey?

**Interviewee:** Distraction from the environment when I'm doing the survey. I'm not easily distracted. I don't think I have any problem with that. Watch survey is pretty fast.

**Interviewer:** Yes, quick and easy.

**Interviewee:** It's like one second. Then the buzz survey is a little longer, but I think usually when I do the survey, I'm alone or I'm not talking to anyone.

**Interviewer:** Do you--Oh, go ahead.

**Interviewee:** I guess I'm just not distracted wondering what is going on right now to answer the question.

**Interviewer:** Do you think your responses changed depending on the time of day or where the situations in which your responses may have been less accurate like morning versus night or if you're in the lab versus at home?

**Interviewee:** I guess maybe sometimes. If I'm really busy, maybe I'll just skim through it faster. Maybe like in the middle of the day, I'm working and I want to do it fast, I may not take as long. It's more of reflex of answering those questions. Most of the time, I think about it for at least a second before answering.

**Interviewer:** Let's see, you answered that question for me already. Let see, how do you think your motivation or accuracy changed the longer you are in the study?

**Interviewee:** I don't think it changed. I guess the first month probably. I think more I guess. When I see a question, I think about it more because--

**Interviewer:** It was new.

**Interviewee:** Yes, but now, I just go with the flow. [chuckles]

**Interviewer:** Did anything make the study easier or harder the longer it went on?

**Interviewee:** It's probably easier the longer it's been on because I know what the question are, I know what to expect. I can ask when the buzz fares is, and I know when I have to answer this. I know one more question [crosstalk]. It's easier.

**Interviewer:** Are there any other points that we didn't cover that you'd like to discuss? Maybe something that came up that you're like, "I need to tell that team. They need to know this."

**Interviewee:** No, I think the only thing was the vibration thing that I wanted just to share with you, but that's the only thing.

**Interviewer:** Big thing.

**Interviewee:** Yes.

**Interviewer:** Okay.

**Interviewee:** You must know.

**Interviewer:** I know, it's always hard on the spot to like, "I don't know, I can't think of anything. I can't remember more impression."

**Interviewee:** I should write down. [chuckles]

**Interviewer:** Yes.
