# Supplementary material for: Burden and Inattentive Responding in a 12-Month Intensive Longitudinal Study: Interview Study Among Young Adults
Source: JMIR Form Res. 2024 Aug 2;8:e52165. doi: 10.2196/52165 (PMC11329843; doi:10.2196/52165)
Supplement: Multimedia Appendix 1 [file formative_v8i1e52165_app1.zip › Transcripts/retrievergeckoabroad_audio_8.1.22.m4a.docx]

**Interviewer:** To start, can you provide me with some of your overall general feedback regarding the study?

**Participant:** I would say that unless you design the study in a way such that you care only about one or two variables that you are asking for, and the others were just thrown in. I would think that only a few variables of the questions you're asking can be neatly extracted from the responses because a lot of the times, the questions that you're asking don't really apply. Do you get what I mean by that?

**Interviewer:** You're talking about watch questions, phone questions.

**Participant:** I am talking about phone questions.

**Interviewer:** Oh, you're saying they don't really feel like they apply?

**Participant:** Sometimes they just not apply because of the way they're worded and let's see--

**Interviewer:** What did you typically do in those situations when you felt like they didn't apply to you in that moment? How did you answer them?

**Participant:** I tried to do a best approximation, but sometimes they felt like I was just answering randomly. I was trying to not answer randomly, but effectively I think it was sort of me answering randomly.

**Interviewer:** In those types of situations?

**Participant:** In those types of situations. Whether I'm feeling fatigued or not energetic or not, it's easier to answer compared to some of the other questions that were sometimes asked. It's a lot of notifications.

**Interviewer:** It's a lot of notifications. Yes.

**Participant:** It's a lot of notifications. That's what I signed up for. That's fine.

[laughter]

If there was some trade-off where I could delay some notifications, I could be like, "Oh, I can't answer this right now because I am having a meeting or busy with something, but I will be able to answer this a few minutes from now." I know you want to get your time data, but if there was some compromise thing you could-- or if you could warn people of notifications upcoming. Like in 15 minutes you'll get a notification, you'll get a survey prompt. I think that would be super useful.

I could time like break with some kind of a break, let's time out with people. I can be like, "I'll need to be away for 15 minutes, or after 15 minutes I'll need to be away." Instead of like, I'm talking to somebody and now there's this notification and I have to answer that notification, but this person will think I'm being rude.

**Interviewer:** Yes. That makes it hard. This is a question I was going to ask later, but we're on the topic. What part was more disturbing for you? Was it the actual either vibration or the buzz from the phone or the sound for a survey, either the phone or the watch, or was it actually taking time aside to do the survey? Like which part was more disruptive for you?

**Participant:** Taking time aside to do the survey wasn't as disruptive as the notification when I'm doing something else. When I'm very deep in concentrating or socializing or I'm trying to answer the surveys, but I'm in the shower and I miss the survey. If it's one of those daily survey questions, those prompts which ask me about my sleep time and they repeat, that's okay. But on a burst day when I'm trying to get as many as possible, I feel like, oh, I missed that. Then I'm like, oh, I should try to not miss others.

**Interviewer:** Yes, definitely. I'm going to ask more specific questions. For this first section though, I want to learn a little bit more about your experience participating in the study. First, I want to learn a little bit about how you first learned about the study. Do you remember where you first learned about it from?

**Participant:** Yes. ResearchMatch.

**Interviewer:** Okay. Do you remember what about the study interested you to want to participate?

**Participant:** I want to help all science, but very tangible compensation.

[chuckling]

That was what attracted me to your study.

**Interviewer:** Absolutely.

**Participant:** I'm physically active.

**Interviewer:** It was important to at least have that compensation part in the study?

**Participant:** Yes.

**Interviewer:** Yes, definitely. Can you describe to me what motivated you to continue answering surveys in the study? I know you mentioned obviously what wanted you to join the study.

**Participant:** I would say that I like to stick to things. The compensation isn't that big of a deal for me, but I thought, "Okay, I signed up for this so I should see it through." It's not that much work. It's annoying at times. It's a consideration at times, but it's not that much work. I should see through.

**Interviewer:** That's good. That's a commitment though for sure. [chuckles] Can you describe the process of answering surveys on a typical burst day? You mentioned, if you were in the shower, you would miss one or something, but can you tell me about how typically, what a burst day was like for you, I guess?

**Participant:** Now is a good time to also-- Now that I remember, one problem with answering the questions on a birthday would be like, I need to answer this now before the survey goes away. It's asking me questions about how I'm feeling. Fatigue, energetic is me not that hard, but some other questions can be more subtle, like "Am I really feeling relaxed or not?"

You know those kind of things and I would not get enough time to really properly answer those because I'm not sure what the answer to those questions are. "Am I tense? Am I not tense?" I don't know at, certain times, especially when I have this deadline to respond right away. What was the worst day like for me? Initially, I had some problems being very responsive with the burst service because I like to keep my phone on vibrate.

I don't have the ringer up, so sometimes I'd often miss them. When I work from home, first isn't so bad. I'm generally just doing something and every hour, I get a notification either on the vibrate or it's like ringing and I hear it and I try to answer as honestly as I can. Sometimes I don't have my phone on me or I'm busy. I don't know. What else would you like to know about my work?

**Interviewer:** Did you have a goal number of surveys that you tried to answer each day? Or did you track your kind of completion of your number of surveys?

**Participant:** Yes. I tried to answer as many as I could. I wouldn't purposefully just not answer a survey if I had hit the 11 mark. If I had only answered 5 out of 10, then I'd feel slightly stressed out about it. Not really stressed out, but I'd be like-

**Interviewer:** It'd be in the back of your mind.

**Participant:** Yes. It would be in the back of my mind. I would try to make an effort to reach that magic number 11 or 8 because 8 was the minimum. Even if I wasn't getting paid, I wanted my data to be useful. Yes, I'd keep track of it.

**Interviewer:** Would anything have made participation in the study more fun or rewarding for you besides paying more? Of course paying more is great, but anything else that would've made participation in the study more fun or rewarding?

**Participant:** If the notification thing was more controlled. Let me tell you about my watch notifications. I turned them all off after a while because-- As in I turned on, Do Not Disturb because there were way too many of those-

**Interviewer:** Questions that came up.

**Participant:** Yes. On a certain day it would be like 30, 40, I don't know exact numbers, but that order of magnitude. If it's like buzzing on my wrist, then it's more entirely grabbing all my attention. That felt like too much for me. I was told that I don't have to answer those, so I chose to not answer those.

**Interviewer:** Yes.

**Participant:** Maybe you could try to have fewer of these that day and maybe that would ensure better compliance and higher-quality feedback because on a burst day if someone is answering up to 15 survey questions, maybe on their 14th response, they're not making as much of an effort to really answer the questions.

**Interviewer:** They're like, "I've reached my limit."

**Participant:** Another thing that would help would be, I don't know if the participants are supposed to know what the survey is for. Are you supposed to keep some things a secret from the participants?

**Interviewer:** Yes, there's stuff that knowing what the surveys are measuring and stuff, that's all kept a secret for data collection, although data collection's finishing up soon, so hopefully, we can share more with you guys.

**Participant:** Some of the prompts towards the end of each survey, whether it's a burst survey or end of this survey, some of the prompts felt a little bit annoying. I intend to not sit or sit, stuff like that. It felt like you were trying to say that I should be engaging in behavior A, B, or C and maybe that's true. Maybe I should be engaging in behavior A, B, or C, but at the time while I'm answering those survey questions, I'm like, "Yes but you don't have to be like that. You don't have to like,"--You know what I mean.

**Interviewer:** Felt sneaky or something maybe.

**Participant:** Changing the wording of the questions could, unless that's what you are explicitly trying to do, if the goal of the study was to remind people to do these things regularly, then I guess what else could you do? Especially your sitting part caught me, I intend to not sit. How am I supposed to work if I'm not sitting?

**Interviewer:** Standing desk, I'm kidding.

**Participant:** Standing desk, not everyone could have them.

**Interviewer:** I know. I can't do that again. The study was looking to see how your health behaviors, like sitting, sleeping, physical activity, how that kind of changes naturally over a year. Most big studies have shown how if you find physical activity rewarding, that's going to affect how much you do but we're interested in the little, day-to-day things to see if that changes your activity, your sitting, your sleeping if that helps answer a question for you.

**Participant:** It does. I'm interested in a lot more of the details, but I'll wait for the paper to come out I guess.

**Interviewer:** We're wrapping up at the end of next month. We're almost done. We'll hopefully provide-- Do you remember getting any of the newsletters or those emails with some of the data? Hopefully, we'll give something like that, telling you what we're doing now that data collection is done and what we're working on. I'm assuming you work in research or you are interested. We'll share that for sure. We'll keep you on the email chain with everything even though you've finished the study.

**Participant:** That'll be fine.

**Interviewer:** For this next section, we've brushed on a little of these topics, but I want to learn a little bit about increased burden. We know obviously the time study was not easy at times and so, we want to learn a little bit about the challenges that you may have faced while in it. First question is what were some specific situations or just general situations in which it was particularly challenging to answer some of the surveys?

**Participant:** When I was travelling, I went back home to India and I was with family and mainly social situations, can you say that's social situations and situations where I would have to-- If I'm traveling, if I'm in a car with other people, then, first of all, I can't hear stuff in the car anyway because of how loud things can get. Even when I have my hanger-on, I generally don't have it all the way up. I prefer quiet environments.

I guess because of that when I was traveling in cars, sometimes I would miss the notifications and if I'm very busy, then I suppose it could be a challenge. No, I guess it's really a social aspect of it. If I'm busy by myself, then I can make some time. Even if it's a little bit disruptive, I can make a minute or two to answer the survey questions, but social situations that can be--

**Interviewer:** Make it difficult, sure.

**Participant:** I'm trying to think if there's any other. My answers to your question may not be the most accurate answers because I haven't thought it through.

**Interviewer:** That's understandable totally. I think if I would've given you these maybe a month before, prep for them, understand but no, don't worry. This is just general feedback of what you experienced and maybe what comes to mind at the most. Don't worry about being super accurate. This is great feedback.

What most frequently led you to be unable to, or to miss answering some surveys? I know you social situations make it kind of hard, but was there anything specific that happened where you specifically were like, "I can't answer surveys during this time?"

**Participant:** Other than social situations, if I'm really stressed out and working, I have some deadline and I really need to focus or if I'm running late, I'm often running late for stuff. I wasn't late today, but I'm often running late for stuff, and if I'm running late, then taking that two minutes to answer the survey question.

**Interviewer:** Makes it hard. Did you ever dismiss a survey? Did you ever prefer to just dismiss it, if you saw it and just dismiss it?

**Participant:** Yes, if I am engaged with the world around me in some way like that if I am running late. I teach sometimes as a TA. I can see the notification when I'm teaching, so it's in a social situation, I can't answer while I'm doing that.

**Interviewer:** That just cause you, of course, to miss it.

**Participant:** Or I'm in a meeting with people and if it's another graduate student or if it's a friend, I can be like, "Okay, hold on," but if it's a supervisor or something very official or it's unprofessional of me to do so I won't do it.

**Interviewer:** That makes sense. Along that line, what did you typically tell friends or family or coworkers about the study if they asked or like, "Are you on your phone?"

**Participant:** Depending on how close I was with them, some friends, I told them what this was about. I didn't tell them all the details, but I told them, "Oh, I'm getting paid to answer these questions, **[inaudible 00:18:52]**. The really close people, I told everything about. I told them about the whole deal, the whole study.

If I didn't know them that well-- Sometimes, like in today's world, it's okay to be on your phone despite talking to someone. I would use that. I don't use my phone excessively, I think but a lot of people do. I would just be on my phone and not explain myself.

**Interviewer:** For this last big section here, I'm curious to talk about accuracy. I know you mentioned earlier sometimes some of the questions didn't really apply, so you just answered them and maybe they weren't super accurate. I'm curious to see how you dealt with other situations along those lines. How did you typically handle distractions when taking a survey?

**Participant:** I try to not be distracted, but I'd often be distracted. Because despite everything, I wouldn't count answering the surveys as something important in my day. Work is something important to me. Physical activity itself is something important to me. I don't know, a number of other things is important to me. This is something I want to do because I consider it as an obligation but it's not important to me to be super accurate. I'd often be distracted. That's the obvious answer.

**Interviewer:** I appreciate that. Thank you. Were there certain specific situations in which your responses were maybe less accurate? Maybe you were like, I was always late in the mornings and so I just wasn't super accurate then, or maybe certain times of the day or if you were around certain types of people, like do you know if there's situations that it repeatedly, you noticed, it was maybe a little less accurate?

**Participant:** Sometimes like the last survey of the day. The last two surveys of the day, maybe not always sometimes when I'm sleepy I would answer them inaccurately. Again, in the vicinity of other social commitments-- Typically, if I see my morning survey on time, I would answer it accurately.

Let's say it's a burst day. It's like 9:00 AM, 10:00 AM. I haven't started interacting with the world. I haven't become busy in tasks as much yet, then I would answer those surveys accurately. If it's 3 o'clock and I have something going on, that I need to get back to, then maybe I would answer those surveys less accurately.

**Interviewer:** A couple more questions here. How do you think your motivation or accuracy may be changed as you're in a study longer?

**Participant:** I had some phone problems that I had. Initially, I kept my phone on vibrate and sometimes I won't hear my notifications and I would miss a lot of these surveys. My motivation was lower back then.

**Interviewer:** That was a while ago, right?

**Participant:** Yes, that was a while ago. My motivation has been, I don't know, more or less steady over the months. Controlling for other factors like travel and how busy I've gotten. Controlling for those things I would say my motivation has been steady. I also remember that a while back when my response rate was low, or whenever my response rate dipped, it's because I'm busier interacting with the world around me. When I control for those things, then I think my motivation has been, they really changed much.

**Interviewer:** Let's see. Last question here. It's kind of off-topic, not about accuracy but what did you think about the questions and messages that were not related to measuring either health behaviors or routines or mood on the phone?

**Participant:** During the last screen that was displayed, as I would finish the survey.

**Interviewer:** Not the one that showed a trivia fact. Not that one. There were actual questions that came up throughout the surveys either, well you would have noticed them on the phone.

**Participant:** Oh, the ones that were checking to make sure that I was paying attention to the survey.

**Interviewer:** What did you think of those ones?

**Participant:** Some of them were stupid [chuckles] but they worked. I remember there were one or two instances where there wasn't a super 100% clear one answer but depending on the meaning of the question, there could be up to two answers.

**Interviewer:** No. Go ahead. Sorry.

**Participant:** I was just going to say that I understand the point and yes, okay, fair enough I don't mind that.

**Interviewer:** Did any stick out to you or any memorable?

**Participant:** Not really, they were all forgettable.

[laughter]

**Interviewer:** Do you have suggestions on how to make them better?

**Participant:** Yes. Did someone actually come up with those questions and the responses?

**Interviewer:** I think it might have been a generator maybe.

**Participant:** Yes, I would suggest using a generator but using a more sophisticated encryption of the generator.

**Interviewer:** You don't have situations where it could be two options?

**Participant:** Yes, generally, it wasn't like that. Generally, it was like, which among them is not a city and George Washington would be one of the options. Maybe keeping the questions simpler would help. There could be people out there who are not aware that Paris is a city. That's a bad example.

**Interviewer:** No, I know what you mean. That's a good point.

**Participant:** Which of these start with a G? Just many variations of that maybe.

**Interviewer:** That's a good idea. Okay, thank you for hanging in there for those questions.

**[00:26:54] [END OF AUDIO]**
