# Supplementary material for: Burden and Inattentive Responding in a 12-Month Intensive Longitudinal Study: Interview Study Among Young Adults
Source: JMIR Form Res. 2024 Aug 2;8:e52165. doi: 10.2196/52165 (PMC11329843; doi:10.2196/52165)
Supplement: Multimedia Appendix 1 [file formative_v8i1e52165_app1.zip › Transcripts/caucuscattlemockup_audio_7.14.22.m4a.docx]

**Ellen:** Yes, no worries. Overall it was fine. It would get at the beginning, especially when it was not as routine, I think I was more on top of it but then it became, oh, this is more of a chore especially during the weekends and stuff like that when I was doing the burst periods and I would be out with people and then be like, Oh, I have to answer my study. Give me two minutes or whatever interrupting dinner was a little bit more of a burden or a hassle. Especially the longer that I was in the study. That was the overall experience of it, but nothing major that I couldn't overcome.

**Interviewer:** Okay. I'm going to ask more specific questions also about your experience. How did you first learn about this study? I know it's been a year. It's been a while.

**Ellen:** I think it was through Research Match. I'm pretty sure it was through Research Match.

**Interviewer:** Do you remember what features of the study interested you to want to join?

**Ellen:** [crosstalk] When I first got the notification for it, it just didn't seem like too bad of a study. It said, "Answer phone surveys and you'll get paid to do it", which seemed just fine, the vagueness of it I guess drew me in a little bit. It was a year ago. I can't really remember.

**Interviewer:** Understandable. It's crazy how it's been year. Can you describe to us what motivated you to continue to answer surveys in the study throughout that year?

**Ellen:** Keeping the watch and money.

**Interviewer:** Totally.

**Ellen:** That's it.

**Interviewer:** That's it. Along money-wise, how important was compensation to you in this study?

**Ellen:** It was more important towards the beginning of it. Answering the surveys on the watch itself, that was fine, that really didn't bother me at all. For the first surveys, money was probably the biggest factor and just having the benchmark of like, oh, y'all want us to answer eight surveys a day. I wanted to reach that kind of benchmark,

**Interviewer:** Was that your goal number then? Was eight when you were--

**Ellen:** Especially towards the end. In the beginning I was like, oh, I just want to answer all of them. It'll help you all with getting your data too. Then towards the end, I was I just want to at least meet the minimum requirements so that I can make sure that I'm getting compensated for the effort that I'm putting into it.

**Interviewer:** Absolutely, yes.

**Ellen:** I guess I want to go back to my previous answer. I think in the beginning, I was also a little bit more motivated to just help with the research because it seemed really exciting. I wanted to just make sure that y'all had good data and I stopped caring as much towards like months 10, 11, 12.

**Interviewer:** Yes. Towards the end it gets a little tiresome. Around month 10, 11, 12, was it like, "I just want to at least be compensated for my time"?

**Ellen:** Basically yes. It's I just, I did want to be done with it, but I want to like at least keep going and get the compensation.

**Interviewer:** Understandable. Can you describe to us the process of answering phone surveys, specifically phone surveys on a typical first day? How did that process work for you? You said your goal was eight, but how was that?

**Ellen:** Mostly on the weekend days, like on Saturday and Sunday, my goal was eight a lot of times because part of this was I also started a romantic relationship within the last five months or so. I was like spending time over at my romantic partner's place and wanting to be with them and not looking at my phone and stuff. Essentially, in the mornings, I would leave my phone charging or maybe it had died the previous night because I forgot to plug it in before going to bed, and wouldn't remember until a few hours after I had woken up. I already would have like lost a few hours of the day.

By the time that my phone was fully charged, it was next to me. It might have already been noon or so, it's okay, [crosstalk] I need to answer the survey questions. I would try to be a little bit more on top of it knowing that, oh it's noon but I'd probably go to sleep at-- I'm realistically not going to be looking at my film that much after 10:00 PM and so I need to answer all of the survey questions in between that timeframe.

**Interviewer:** Yes. I'm short on hours now I got a good date.

**Ellen:** Yes. something that.

**Interviewer:** Did you track your completion then when you were on the app, how it shows you the different things--?

**Ellen:** Yes, I would pull down like the notification at the top which says like 13 surveys and 8 answered or whatever. Something like that. I would look at that.

**Interviewer:** What would have made participation in the study more fun or rewarding besides paying more money because that would obviously before reporting?

**Ellen:** I don't know. Maybe a reminder midway through of what the research was going to be used for because since at the beginning, I was motivated to help y'all with the research and then towards the end I had lost sight of that. I think that that could have made it be a little bit more rewarding. Just the reminder that this is all part of something bigger. I don't think that anything could have made it more like fun. I did really the random questions that y'all would throw in of like, which of these is a city and it would say spaghetti noodle or something like that. Those were really fun.

**Interviewer:** Good. I'm glad. That was actually going to be one of my questions, do you remember those questions?

**Ellen:** I liked them, I would send screenshots of them on my phone to people and look at what my servers asking me.

**Interviewer:** Did you have a favorite of those questions? The spaghetti one. That was pretty good.

**Ellen:** I don't know, let me look at what my screenshots were.

**Interviewer:** Yes.

**Ellen:** I think I might have deleted them as I was like studying them out. I think that they were all-- I just liked a lot of them. I liked what is questions instead of the number related questions. I liked those better than also the select this answer out of this queue or whatever. Are you paying attention questions. I like the more, which one of these are are president and it's like oranges, [crosstalk]

**Interviewer:** Okay, let's see. For this next section, I want to learn more about situations of increased burden that the study may have caused. We know obviously being in the time study for a year is not easy at times. I want to learn a little bit more about the challenges that you've experienced while you were participating. What were situations in which it was particularly challenging to answer the surveys?

**Ellen:** It was inconvenient was more of rather than challenging. There were times where it was very inconvenient to answer the surveys. It's mostly when I'm around people that I don't want to interrupt. If it was during the workday on a Friday or a Monday when I was just at home because I work from home, that was fine. I think I was very on top of answering the survey questions then and didn't really bother me that much. Situations when I'm around people, like at dinner, any social event, made it a little bit more challenging to answer survey questions. Then on the watch, the only time that it was challenging for me to answer questions was when I was biking.

**Interviewer:** Yes.

**Ellen:** Physically trying to like stay focused not fall off and like hit by a car.

**Interviewer:** Yes, for sure. That's a good, like, I'm not even going to look at this right now. I'm just going to keep going. It's a crazy vibration though, you're probably like, "What the hell?"

**Ellen:** Yes. I was stop doing this. I'm just trying to stay on the road.

**Interviewer:** Yes, for sure. What was the most disruptive part of the surveys or the study? Was it the vibration, was it actually taking time to answer the surveys?

**Ellen:** It was the taking time to answer the surveys. It was like, "Oh, I only have five minutes to do this. I need to answer it right now." Something, I don't know if you'll have a question on this later, but sometimes I would start answering a survey, then I waited too long or something. It would close out in the middle. That was really a little bit stress-inducing because I was already towards the end of it. I only had two questions left and then it was gone.

**Interviewer:** That's frustrating for sure. What most frequently led you to be unable to or to just miss answering a survey? I know like when it was disrupted, but what the biggest reason of why you missed one?

**Ellen:** A lot of times, it would be because I didn't see it. Especially if I was out or something, if I had my phone in my bag and it wasn't vibrating against my person, then I just would miss it altogether. Yes that was the biggest reason. If I had it on my table next to me or had it in my pocket, then I would feel it and I would answer the survey.

**Interviewer:** What most frequently led you to dismiss a survey? Can you describe an instance that led you to just dismiss the survey rather than answering it?

**Ellen:** Like if I was aware that I had to answer one?

**Interviewer:** Yes and you're just like, "I am not doing it."

**Ellen:** Sometimes, especially at the end of the day, if I was like, well I've already answered X number of surveys. I've answered seven, I only have the daily survey left, I'm just going to skip a few, but usually, if I was aware of the survey, I would answer them I guess. If I was aware of the survey that I probably was in a spot where I had my phone close enough to me. Those usually were times when I was not with a big group of people.

**Interviewer:** Let's see. What did you typically tell friends or family about the study if they asked about it? I'm sure they all heard [crosstalk] questions of something or saw you answering something.

**Ellen:** Oh, my gosh. Sorry. I'm just trying to remember back what I was telling people. I think I told them I'm answering some survey questions on my phone and on the watch. It'll be like a two-second question on my watch. There's 60 of them a day or something like that. I would always lead with that and they're like, "Oh, it's not like that big of a deal. That's totally cool." I was like, "Also, two times a month, I have these periods where I have to answer questions every hour for four days." They were like, "Okay, cool."

I was like, "Yes, but I got paid money for it and I get to keep my watch to the end, which is pretty cool." I tried to remember what y'all were studying but I don't really remember anymore. So I tried to say that in there. It's like, this is why I'm doing it.

**Interviewer:** If you're curious of the purpose of the study, we're trying to see how young adults health behaviors change naturally over a year. Research has shown big things like if you find physical activity rewarding and that affects how much physical activity you do, but we were interested in how little day-to-day factors influence that or affect that. Hence all of the questions, hence the year long, hence questions every single day. Yes, those in-the-moment questions.

**Ellen:** That's why you guys asked about physical activity a lot. [crosstalk] I don't remember which health behaviors because it was like y'all would ask about like eating healthy, sleep, activity, sitting.

**Interviewer:** Those are the big ones right there.

**Ellen:** Sometimes I would answer the sitting questions, "Well, I work from home, an administrative job, I can't help it."

**Interviewer:** I sit all the time. We're done with data collection at the end of August. So what? In a month? A little bit of a month. We're going to try to put something together or we'll send you guys-- I don't know if you remember receiving any of the newsletter emails or birthday emails?

**Ellen:** I think I saw some of those, yes.

**Interviewer:** We'll try to put one together too that hopefully is able to provide some more insight. Obviously, when we're doing data collection we have to be limited on what we share, but once we're done with that, we can hopefully provide some more or give you some feedback or not feedback, but data from the study as well. Look out for that.

**Ellen:** Yes, I want to see what I'm contributing to.

**Interviewer:** Yes, it's a lot of data for sure. It'd be good to know all those things. For this next section, I want to learn a little bit more about response accuracy. Besides not answering a survey if you were busy or doing something, we're curious if there were any ways that you dealt-- what other ways you dealt with some of these challenges or burdens while you were actually taking a survey. How did you typically handle distractions when taking a survey?

**Ellen:** Distractions-- Can you clarify that a little bit-- distractions?

**Interviewer:** Yes. Like let's say you are with a group of friends and you decide to answer a survey, but everyone's talking, you're at a restaurant, a busy restaurant. Would that affect your answers? Would you just quickly go through a survey? How did that typically--

**Ellen:** If I was answering-- I would still try to answer it decently accurately. The questions themselves were short enough. It was very quick for me to just gauge, am I happy, sad, all of the different feelings and stuff like that. If I was going to answer it then I would do so pretty accurately, I'd say. Me being around people wouldn't affect my responses other than whether or not I did it.

**Interviewer:** Would you tell people like, "Hang on, I'm doing a survey."

**Ellen:** Yes, especially the friends that I saw all the time, they all knew. They just like, "Okay, hold on survey time."

**Interviewer:** Let her do her thing.

**Ellen:** Sometimes they would hear the buzzing and be like, "Oh, Ellen it's time for you to do a survey" I'd be like, "Oh, thanks for telling me."

**Interviewer:** That's so good and funny. That's good. Those are good friends. Were there situations in which your survey responses would have been maybe less accurate?

**Ellen:** I think the ones would have been inaccurate would have been like the, "Were you physically active 20-something minutes ago?" I'd be like, "I don't know." I think so but it was very approximate. I want to remember, "Oh, was I walking around 15 minutes ago or had I finished what I was doing and sat down somewhere?" I couldn't remember those things. Otherwise, I think I was pretty accurate with it.

**Interviewer:** How do you think your motivation changed as you were in the study longer or accuracy? Like you said, you were mostly accurate for most of them. Do you think it changed at all as a study went on?

**Ellen:** Did my accuracy change? I don't know. I actually think that as the study wore on, I was more able to answer the questions and the survey properly because it was easier for me to gauge how I was feeling. I'm thinking about certain things just because I was more practiced at it of like checking, doing the whole mental check-ins every hour of, am I actually stressed? Am I frustrated? Or am I nervous or am I tense? Distinguishing between those things was a nice skill, I guess. [chuckles]

**Interviewer:** For sure you could have felt that over the year and now you're like, "What do I do with this skill that I just built?"

**Ellen:** I'm like, "Dang, do y'all also like a mindfulness study now?"

**Interviewer:** Yes, I mean checking in on your feelings all the time and everything you have to use like calm or I don't know what some of those kind of mind flaps are.

**Ellen:** I don't need them now. I have the time study just living in my brain.

**Interviewer:** Yes. [laughs] That's hilarious. Am I feeling happy right now? What's going on? Okay, I think you answered this one actually. Are there any other points that we didn't cover that you'd like to discuss about the study?

**Ellen:** Let's see. I don't think so. I think I shared most of my thoughts and feelings about the study with y'all already. I told you all about what the frustrations were, mostly of would answer a study and that it wouldn't save.

**Interviewer:** I have these random questions. They're not related to the study more so, just about you. Do you know anyone else who participated in the study?

**Ellen:** No.

**Interviewer:** Okay. We can skip all the other questions after that. This one is talking about exercise. In the past month, have you exercised or performed any type of physical activity such as going for a walk?

**Ellen:** Oh, yes.

**Interviewer:** What types of exercise you typically do?

**Ellen:** I would do yoga. I would go on a walk. I would go like on bike rides. I would go dancing. Those are the main ones. I was moving some things also like lifting and carrying moderate to heavy loads.

**Interviewer:** What types-- I'm sorry, not what types. What days of the week? What days of the week are you typically doing these exercises?

**Ellen:** I'm doing dancing on obviously mostly doing on like Fridays and Saturdays.

**Interviewer:** Yes, for sure.

**Ellen:** Let's see. For like biking around, mostly like Saturday, Sundays. Sometimes during the week like on-- That's not on any like routine. Walking, I think I spent more time walking on probably Tuesdays, Thursdays, Friday, Saturday, Sunday. Didn't do a lot of walking on Mondays and Wednesdays. If I had to like gauge that. That was the other activity that I said I did? Moving and stuff like that wasn't on any regular schedule. Yoga I think was-- I used to do that a lot more regularly, but over these past few months, it's been more irregular. Just like one to two days a week, more randomly. Usually more on Mondays through Thursdays and not so much Fridays through Sunday.

**Interviewer:** Are you doing that like mornings, evenings?

**Ellen:** Mornings.

**Interviewer:** Mornings. Okay.

**Ellen:** Yes.

**Interviewer:** I think that you answered all these other ones. You answered that one. Okay. We are all done.

**Ellen:** Oh, sweet. Okay

**[00:22:14] [END OF AUDIO]**
