# Supplementary material for: Burden and Inattentive Responding in a 12-Month Intensive Longitudinal Study: Interview Study Among Young Adults
Source: JMIR Form Res. 2024 Aug 2;8:e52165. doi: 10.2196/52165 (PMC11329843; doi:10.2196/52165)
Supplement: Multimedia Appendix 1 [file formative_v8i1e52165_app1.zip › Transcripts/pettytransfixedsolubly_audio_8.19.22.m4a.docx]

**Interviewer:** To start, can you provide me with some of your overall general feedback?

**Respondent:** Yes. I guess a little bit less than halfway through, I switched jobs. The first couple months when I was in my office job, I thought it was really easy to go through and answer the questions, do the birth stuff, the questions on the watch and everything. It did get more difficult when I started working in a hospital because I can't have my phone on me while I'm on the floor working with patients. We can only have it during our break. I usually just have it off.

The same thing with the watch because, when it has questions, it goes off so long. I ended up turning on "Do not disturb" just because it would go off at random times when I was working with patients and stuff. It was a little distracting. I thought the questions and stuff I thought were really easy. I feel like it made me think about things a little bit too. Like when I was asked, "Oh, do you have a goal for how much walking you're going to do this week or how much you want to sit," or stuff like that, like the healthy foods, eating stuff like that. I don't know.

It just made me think about it a little bit more when I would do those, and at the end of the day too when you would go through the end of the day questions and it would ask you if you met your goals for the day and the ones on Sunday for the week. I felt like that was kind of nice. It did make me think about it a little bit more because I don't typically set goals.

It was good to think about because then days that I don't work, I could be like, "Okay, yes. Let's go for a walk and let's do this," or something else to keep up with it. I felt bad because, after I started my hospital job, I wasn't able to do the bursts as much or anything. I didn't think it was hard to keep up with it prior to switching jobs.

**Interviewer:** Yes, that's a definite setting where it is a little bit harder to-- Obviously, you can't have your phone. When you're getting prompted all the time, it's a little difficult to do that.

**Respondent:** Yes.

**Interviewer:** I'm going to ask more specific questions. If a question is ever unclear, please feel free to ask me for clarification. First questions I have are going to be a little bit about your motivation for being in this study and your experience participating in it. First question is, how did you learn about this study? I know it's been a year.

**Respondent:** Hold on one second. Hey.

**Interviewer:** [laughs] Don't worry about it. I get it.

**Respondent:** [laughs] I signed up for-- it's called Research Match. It's a website or an app or something. I signed up for a while ago and so I got I think it was just an email about it and I was reading through it, and I thought that it sounded interesting. At the time, before I worked at the hospital, I did clinical research at the Penn State College of Medicine. I was like, "Oh, that'd be so cool to be able to be on the other side of [laughs] the research." I just thought it would be something fun to do and to participate in.

**Interviewer:** Do you remember if there were any aspects about the study that interested you, that stood out to you?

**Respondent:** I don't remember specifically because I feel like the email that I got, they're usually really brief. I don't remember what specifically it said. I just saw that I qualified for it. I don't know, I was just-

**Interviewer:** You're like, yes, let's do it.

**Respondent:** -wanted to do it. [chuckles]

**Interviewer:** Yes. That's awesome. Can you describe what motivated you to continue answer surveys or questions in the study?

**Respondent:** Yes.

**Respondent:** Obviously besides school or work started, the hospital job started.

**Respondent:** Yes. I feel like that a lot of that came from working in research to begin with. I was like, "Okay, I want to continue because I want it to be helpful. I want to try to give them as much information as I can so that you guys have information and can put it towards what you're trying [chuckles] to do."

**Interviewer:** What am I trying to ask now? I'm looking at question and I'm trying to [chuckles] incorporate it together. Compensation in the study, how important was compensation in the study for you when looking at it?

**Respondent:** Not especially. It was cool. It was fun to have the watch and use that. It was nice to know that I would potentially be able to keep it at the end. I don't know. I wasn't really focused on the monetary aspect of it.

**Interviewer:** Can you describe the typical process of answering phone surveys during a burst period, what that was like for you?

**Respondent:** Yes. The question sets would come through about every hour or so. We have multiple chances. For a couple months of the study, I was getting my master's degree too, so I was like working on school. Occasionally, if I got one and I couldn't answer it right away, I would give it a second. If I did get a burst notification and I was doing something quick, I would finish it up and then go and go through and answer the questions.

Usually, it took, I don't know, maybe a minute to get through. After the first couple, you know what the questions are going to be so it's easier to go through faster and, yes, just knock it out essentially and then wait for the next one. There were some times, especially if I was taking an exam for school or when I again started working at the hospital that I would not be able to answer. Otherwise, yes, I could go through them.

**Interviewer:** Do you have like a certain number that you tried to reach each time? A goal number of surveys that you tried to reach during the first periods?

**Respondent:** Not really. If I was available for the whole day, I would try to answer all of them if I could. No, I don't think I really have a goal for-- Just as many as I could.

**Interviewer:** Okay. Besides paying more money, would anything have made participation in the study more fun or rewarding?

**Respondent:** I don't know. I thought the little blurbs, fun facts and stuff at the end of the surveys, I thought were really cute. That was a fun thing. My mom is a travel nurse. When she was home and I would go through the burst periods, I would always read her the little fun facts too. [laughs] I would do it.

I don't know. I thought that there was a lot of stuff that was interactive with the questions. Even on the watch when you'd answer it and it would say do the little things in different languages and say like, "Good job" and cute things like that, I don't know, it was a cute touch. I thought that that was nice. It made me smile whenever I would [chuckles] go through it.

**Interviewer:** Good. I'm glad you noticed those. When someone noticed those, **[unintelligible 00:06:51]** so that's good. For this next section of questions, I want to learn a little bit more about situations of increased burden. Obviously, we know being in the time study wasn't easy, especially with certain jobs. We want to learn a little bit more about those challenges that you experienced. What were some situations-- I know you mentioned when you were taking exams or being at work. What were some situations which was particularly challenging to answer those surveys?

**Respondent:** Definitely if I was in an exam for school or if I was working or really invested, focused on a certain assignment, because I'm driving too. There were a couple times I would be driving with the maps on and will get a notification about the survey and it like covers up the thing.

**Interviewer:** It's a pain.

**Respondent:** Yes. That was hard too because it doesn't like go away on its own. You have to swipe it away. That could be challenging sometimes. Then otherwise, if I'm at work, even when I was in my office job, if I was like on the phone with someone because I did research, if I was talking to them for data collection or something, I couldn't answer at that point either.

Usually, if I got one of the first things-- I know that there's a timeframe. If I knew that I was going to be done with it soon, I would still try to go in and see if I could get it done if I couldn't the first notification that I got. I think it was mostly just schoolwork and in the car where I think would **[unintelligible 00:08:20]**

**Interviewer:** What was more disruptive out of the app? Was it the sound or the vibration letting you know you had a survey or was it actually taking the time to do the survey?

**Respondent:** I think the phone itself, I don't know. Both the phone and the watch just vibrate for a long time. That can be a little bit distracting. No, it was more so just like, especially with schoolwork, if I was focused on something, I didn't want to break the focus to go through the questions. I know it's not super long either, but yes. Otherwise, if I'm in a car, I wouldn't want to go through it because that's hard to answer while I'm trying to drive or something.

**Interviewer:** Yes. I'm glad you [crosstalk]--

**Respondent:** It wasn't even like it was annoying to get it. It was just, "Oh, I have to take a second and I need to mentally be done with what I'm doing to go through it, do it."

**Interviewer:** Can you tell me about an instance where you preferred to dismiss a survey? You saw it on there and you're like, "I am too busy right now," or push it away.

**Respondent:** Yes. I think probably there have been times if I've had friends or family over or I'm at another friend or family member's house and I see it, I'm like, "Okay, I'm not going to do it right now because--" If I'm in the middle of a social situation like that, I don't want to like be rude and be like, "Oh, hold on one second," like especially if I'm in the middle of a conversation. If I was in that setting and I was listening or I was doing something different than I would, but if I was in conversation, then I wouldn't answer it.

**Interviewer:** Absolutely. Speaking of family and friends, what do you typically tell them if they asked you about the study?

**Respondent:** Oh, I would just tell them that I was in a research study then you guys were tracking my activity. I would tell them about the questions and stuff on my phone and just explain it. A lot of people thought it was really cool that that I was doing a study. It was fun to explain it a little bit.

**Interviewer:** Read them the cool facts at the end **[unintelligible 00:10:16]**. For this next section of questions, I want to learn a little bit about response accuracy. Besides not answering, like if you were missed a survey or didn't answer a survey, we're curious if there are other ways that you dealt with some challenges while being in the study. How did you typically handle distractions? Taking a survey?

**Respondent:** While I was answering, you said?

**Interviewer:** Yes.

**Respondent:** I don't know. I feel like you have enough time when you go through the survey that if I was like in the middle of it and got distracted by something else, if it was something like that would take a second, I could look away for a second and then go back and answer the questions. Since they really don't take that long, like I said, if I did get distracted by something or if I was in the middle of it and someone was like, "Oh, can you come do this or see this," I'd be like, "Oh, give me like 20 seconds and let me finish and then I can come and be done," you know?

**Interviewer:** What were some situations in which the survey responses may have been less accurate thinking about like if you're around someone else, maybe time of day?

**Respondent:** That's a good question. I feel like sometimes right in the morning when I would get questions about like if I'm feeling like the productivity one, especially, like if I have been-- or procrastinating is what it was. I was like procrastinating or some of those like how I was feeling, if I was like extra fatigued, I just woke up so like I'm-- you know what I mean?

**Interviewer:** Yes.

**Respondent:** I'm sure that you can see, can tell by the time of day, but yes, I just felt like some of them, I was like, I know that I'm tired right now, but I also just woke up so it's like, I don't know if they're going to know that I just woke up or if they're going to think I'm just extra tired for some reason.

**Interviewer:** Yes, that's a good point. Yes. What do you think or how do you think your motivation or accuracy changed the longer you were in the study?

**Respondent:** I feel like answering the questions probably got a little bit easier because I knew what they were and who gauge better, like in the beginning you think a little bit and you're like, how am I feeling? Then it just becomes like second nature almost to think about it and to go through and you're like, oh yes, I'm a little bit tired or, yes, I have been procrastinating today.

Yes, since you're thinking about it, you know the levels a little bit better, so you're able to-- I was able to, I think, answer more accurately the longer I was in it just because of that.

**Interviewer:** Did that made it easier than being in the study longer? Was there anything that made it harder?

**Respondent:** Oh no, that definitely made it easier. I don't think anything really made it harder to answer the questions or anything because then I felt like the longer I was in it because I was ready for the questions I just was able to answer a little bit better. Like the first couple times you answer, I don't know, it was like relative to I guess how I was feeling at that moment, but then the longer you're in it, I could compare to how I was feeling other times. I was like, oh, well, yesterday I was really tired, so today maybe it's not so much, so yes, things like that.

**Interviewer:** Okay. Let's see. Last big question here, and this is along that point, but not really. What did you think about the questions and messages that were not related to measuring either health behaviors, routines, or mood that came up on the phone?

**Respondent:** Oh. It seemed like maybe they were there to keep people focused and not just like tapping through the questions, which was nice, but I did feel sometimes because I knew what the questions were going to be and like, I was going through them, especially on the burst periods, you get one like that and it breaks my routine going through it and I was like, oh, okay. Then you got to reset.

**Interviewer:** Get back into it.

**Respondent:** Yes, they weren't necessarily like, I don't know, annoying or anything. Some of them actually made me laugh. I got one recently that was like, which of these is like not an outdoor activity and it was like being indoors. I read it out loud to my brother and we cracked up because we just thought it was so funny. Some of them, I think they were good to keep you focused or not focused, but make sure that you're paying attention to the questions.

**Interviewer:** Yes.

**Respondent:** They did slow me down a little bit sometimes, but not to an annoying point or anything, you know.

**Respondent:** Okay. That's good. Okay. Are there any other points that we didn't cover that you'd like to discuss? Maybe something that came up that you want to talk about?

**Interviewer:** I don't think so. I think the biggest thing for me was the Google Maps, having that, if I was in the car and would get a notification. Because a couple times, I tried to swipe it away and then my maps got all messed up and then I had to go through and fix it and I was like, "Ugh."

**Interviewer:** It's frustrating,

**Respondent:** It maybe happened like half a dozen times over the year, so it wasn't that big of a deal. It happened enough times and I was like, oh I probably should say something about it.

**Interviewer:** Yes. You aren't the only one.

**Respondent:** Yes. Otherwise, I don't think so. The questions were really easy to understand and to go through.

**[00:15:29] [END OF AUDIO]**
